# Novel Acylselenourea Derivatives: Dual Molecules with Anticancer and Radical Scavenging Activity

**DOI:** 10.3390/antiox12071331

**Published:** 2023-06-23

**Authors:** Nora Astrain-Redin, Asif Raza, Ignacio Encío, Arun K. Sharma, Daniel Plano, Carmen Sanmartín

**Affiliations:** 1 Departamento de Tecnología y Química Farmacéuticas, Universidad de Navarra, Irunlarrea 1, 31008 Pamplona, Spain; nastrain@alumni.unav.es; 2 Department of Pharmacology, Penn State Cancer Institute, CH72, Penn State College of Medicine, 500 University Drive, Hershey, PA 17033, USA; mraza@pennstatehealth.psu.edu (A.R.); asharma1@pennstatehealth.psu.edu (A.K.S.); 3 Instituto de Investigación Sanitaria de Navarra (IdiSNA), Irunlarrea, 3, 31008 Pamplona, Spain; ignacio.encio@unavarra.es; 4 Departamento de Ciencias de la Salud, Universidad Pública de Navarra, Avda. Barañain s/n, 31008 Pamplona, Spain

**Keywords:** acylselenourea, garlic, antioxidant, natural products, selenium, cancer

## Abstract

Oxidative stress surrounding cancer cells provides them with certain growth and survival advantages necessary for disease progression. In this context, Se-containing molecules have gained attention due to their anticancer and antioxidant activity. In our previous work, we synthesized a library of 39 selenoesters containing functional groups commonly present in natural products (NP), which showed potent anticancer activity, but did not demonstrate high radical scavenger activity. Thus, 20 novel Se derivatives resembling NP have been synthesized presenting acylselenourea functionality in their structures. Radical scavenger activity was tested using DPPH assay and in vitro protective effects against ROS-induced cell death caused by H_2_O_2_. Additionally, antiproliferative activity was evaluated in prostate, colon, lung, and breast cancer cell lines, along with their ability to induce apoptosis. Compounds **1.I** and **5.I** showed potent cytotoxicity against the tested cancer cell lines, along with high selectivity indexes and induction of caspase-mediated apoptosis. These compounds exhibited potent and concentration-dependent radical scavenging activity achieving DPPH inhibition similar to ascorbic acid and trolox. To conclude, we have demonstrated that the introduction of Se in the form of acylselenourea into small molecules provides strong radical scavengers in vitro and antiproliferative activity, which may lead to the development of promising dual compounds.

## 1. Introduction

Cancer continues to be the leading cause of death worldwide, accounting for nearly 10 million deaths in 2020. According to the World Health Organization, the most common types of cancer are breast (2.26 million cases), lung (2.21 million cases), and colon and rectum (1.93 million) [1]. Cancer includes a group of complex and multifactorial diseases characterized by the uncontrolled division of body cells. Chemotherapy is one of the most common treatments, and it is used at different stages of the disease. However, cancer’s heterogenicity and the increase in resistance have underlined the need for the development of new anticancer drugs. 

Selenium-containing molecules have emerged as a promising line of research due to the unique properties of this atom, such as its versatility and its high antioxidant capacity. Selenium (Se) is a trace mineral essential for the proper functioning of the organism and is required in small amounts (RDA 50 μg/day). It is a component of the selenoproteins involved in processes related to reproduction, metabolism, DNA synthesis, and protection against oxidative damage. Its antioxidant function has been the starting point of numerous investigations to develop new active molecules containing Se for the treatment of different diseases including infectious diseases, cancer, Alzheimer’s disease, and cardiovascular diseases [2,3]. 

In cancer, there is an imbalance in the oxidation-reduction reactions, and thus, the environment surrounding the cancer cells is a pro-oxidant environment that provides them with certain growth and survival advantages necessary for disease progression [4,5]. Endogenous antioxidant enzymatic systems include glutathione peroxidase, superoxide dismutase, and thioredoxin reductase, which are selenoproteins. Thus, Se exerts its antioxidant property primarily through its role in selenoproteins [6,7]. Se-containing compounds have exhibited different mechanisms of action depending on the type of tumor, including alterations in carcinogen metabolism, stimulation of DNA repair, modulation of the immune response, regulation of angiogenesis and cell migration, stimulation of programmed cell death (apoptosis, autophagy, necroptosis, entosis…), and modulation of the activity of some kinases as the most representative ones [8,9]. Se can be introduced into the molecule in different chemical forms, such as selenoester, acylselenourea, or diselenides. Among all Se-containing molecules, acylselenoureas and selenoureas represent a group of simple but well-established molecules with anticancer and/or antioxidant properties [10,11,12,13]. 

**Figure 1:**
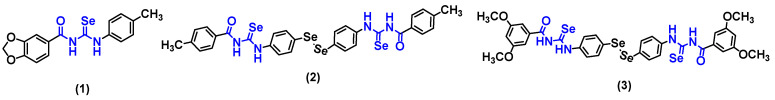
Aromatic acylselenourea derivatives previously synthesized by our research group [10,11].

Our research group previously developed aromatic acylselenoureas showing promising results. We demonstrated that acylselenourea derivatives are excellent radical scavenging agents, which present a fast kinetics behavior and antioxidant effects similar to ascorbic acid and trolox. Compound **1** (Figure 1) stood out as a dual agent showing both antioxidant and selective cytotoxic activity in breast adenocarcinoma cells (MCF-7). Additionally, compounds **2** and **3** (Figure 1), acylselenourea derivatives combined with diselenide motifs, also demonstrated high potency and selectivity against breast cancer MCF-7 cells [10,11]. 

In this work, 20 novel acylselenoureas have been synthesized, purified, and spectroscopically characterized. Moreover, the dose- and time-dependent radical scavenging activity of all the synthesized compounds were assessed in vitro using the colorimetric assays of 2,2-diphenyl-1-picrylhydrazyl (DPPH). Likewise, all the compounds were screened for the cytotoxic activity in vitro at two doses (10 and 50 µM) against a panel of cancer cell lines derived from breast (MDA-MB-231), lung (HTB-54), colon (HT-29), and prostate (DU-145) using the colorimetric assay of 3-(4,5-dimethyl-2-thiazolyl)-2,5-diphenyl-2*H*-tetrazolium bromide (MTT). Compounds exerting the most radical scavenging activity and anticancer capacity were selected for the evaluation of their protective effects on prostate DU-145 cancer cells towards oxidative damage induced by H_2_O_2_ treatment. Their ability to induce apoptosis in DU-145 cells was also determined, along with the measurement of total reactive oxygen species (ROS) levels.

## 2. Materials and Methods

### 2.1. Chemistry 

#### 2.1.1. General Information

All the chemical reagents and solvents were purchased from commercial suppliers, including E. Merck (Darmstadt, Germany), Sigma-Aldrich Química (Alcobendas, Madrid, Spain), Acros Organics (Jassen Pharmaceuticalaan 3a, 2440 Geel, Belgium), and abcr (Karlsruhe, Germany), and were used as received. Reactions were followed by thin-layer chromatography (TLC) on precoated silica gel 60 F_254_ aluminum sheets (Merk, Darmstadt, Germany), and the spots were visualized under UV light. Silica gel 60 (0.040–0.063 mm) 1.09385.2500 (Merk, Darmstadt, Germany) was used for Column Chromatography with hexane/ethyl acetate as the elution solvent. Melting points were determined using a Mettler FP82 + FP80 apparatus (Greifense, Switzerland). ^1^H-, ^13^C-, ^77^Se, COSY, NOESY, HMBC, and HSQC-NMR spectrums were recorded on a Bruker Avance Neo 400 MHz in CDCl_3_ or DMSO-*d*_6_ operating at 400, 100, and 76 MHz, respectively. Chemical shifts were reported in δ values (ppm) and *J* values were reported in hertz (Hz). All the compounds are >95% pure by quantitative NMR (^1^H q-NMR) using dimethyl sulfone as a reference and elemental analysis. For the ^1^H q-NMR, the results are expressed as the percentage of purity and were calculated tracking the signal of the CH_2_ which appears between 5.90 and 6.05 ppm for series **I**, the first alkene hydrogen which appears around 5.9 ppm for series **II**, and the signal of CH_2_ which appears around 3.7 ppm for series **III**.

#### 2.1.2. General Procedure for the Preparation of Acylselenourea Derivatives

Some acyl chlorides were formed by reacting the corresponding carboxylic acid with oxalyl chloride in methylene chloride using *N*,*N*-DMF as a catalyst. The mixture was stirred at room temperature for twelve hours and the reaction solvent was removed by rotatory evaporation in vacuo.

The synthetic procedure was described in previous works of our group [10,11]. Briefly, the corresponding acyl chloride (1 eq.) was reacted with potassium selenocyanate (1 eq.) in anhydrous acetone (30 mL) as a solvent for one hour at room temperature. Then, the corresponding aliphatic amine (1 eq.) was added in situ and allowed to stir for a period of time ranging from 0.5 h to 24 h depending on the heterocycle. The reaction mixture was filtered under vacuum and acetone was removed by rotatory evaporation at reduced pressure. The purification of the compounds was carried out by column chromatography. The eluent used was a gradient of hexane/ethyl acetate, ranging between 15% and 70% of ethyl acetate. The TLCs were performed using a mobile phase of hexane/ethyl acetate with a ratio of 2:8. The Rf range was from 0.18 to 0.75.

***N-(propylcarbamoselenoyl)furan-2-carboxamide* (1.I)**. It was obtained from furan-2-carbonyl chloride as a brown solid in 21% yield; mp: 33–34 °C. ^1^H NMR (400 MHz, CDCl_3_) δ (ppm): 10.98 (s, 1H, NH), 9.37 (s, 1H, NH), 7.66–7.54 (m, 1H, H_furan_), 7.33 (d, 1H, *J* = 3.6 Hz, H_furan_), 6.61 (dd, 1H, *J* = 3.6 Hz, *J* = 1.7 Hz, H_furan_), 3.69 (td, 2H, *J* = 7.1 Hz, *J* = 5.6 Hz, CH_2_), 1.76 (h, 2H, *J* = 7.4 Hz, CH_2_), 1.02 (t, 3H, *J* = 7.4 Hz, CH_3_). ^13^C NMR (100 MHz, CDCl_3_) δ (ppm): 180.1 (C=Se), 156.6 (C=O), 146.5 (C_furan_), 144.8 (C_furan_), 118.9 (C_furan_), 113.4 (C_furan_), 50.7 (CH_2_), 21.5 (CH_2_), 11.4 (CH_3_). ^77^Se NMR (76 MHz, CDCl_3_) δ (ppm): 345. Purity (^1^H q-NMR): 96.3%. Elemental analysis for C_9_H_12_N_2_O_2_Se, calculated/found (percent): C, 41.71/42.03; H, 4.67/4.58; N, 10.81/10.95.

***N-(propylcarbamoselenoyl)thiophene-2-carboxamide* (2.I)**. It was obtained from thiophene-2-carbonyl chloride as a yellow solid in 28% yield; mp: 66–67 °C. ^1^H NMR (400 MHz, CDCl_3_) δ (ppm): 11.06 (s, 1H, NH), 9.14 (s, 1H, NH), 7.71 (dd, 2H, *J* = 9.1 Hz, *J* = 4.4 Hz, H_thiophen_), 7.18 (t, 1H, *J* = 4.4 Hz, H_thiophen_), 3.70 (q, 2H, *J* = 6.9 Hz, CH_2_), 1.83–1.72 (m, 2H, CH_2_), 1.04 (t, 3H, *J* = 7.4 Hz, CH_3_). ^13^C NMR (100 MHz, CDCl_3_) δ (ppm): 180.2 (C=Se), 161.1 (C=O), 135.9 (C_thiophen_), 134.4 (C_thiophen_), 130.8 (C_thiophen_), 128.7 (C_thiophen_), 50.9 (CH_2_), 21.6 (CH_2_), 11.52 (CH_3_). ^77^Se NMR (76 MHz, CDCl_3_) δ (ppm): 340. Purity (^1^H q-NMR): 95.4%. Elemental analysis for C_9_H_12_N_2_OSSe, calculated/found (percent): C, 39.27/38.95; H, 4.39/4.29; N, 10.18/10.09.

***N-(propylcarbamoselenoyl)benzamide* (5.I)**. It was obtained from benzoyl chloride as a white solid in 17% yield; mp: 127–128 °C. ^1^H NMR (400 MHz, CDCl_3_) δ (ppm): 11.25 (s, 1H, NH), 9.29 (s, 1H, NH), 7.87–7.80 (m, 2H, H_aryl_), 7.63 (t, 1H, *J* = 7.5 Hz, H_aryl_), 7.52 (t, 2H, *J* = 7.5 Hz, H_aryl_), 3.71 (td, 2H, *J* = 7.2 Hz, *J* = 5.4 Hz, CH_2_), 1.83–1.73 (m, 2H, CH_2_), 1.09–1.00 (m, 3H, CH_3_). ^13^C NMR (100 MHz, CDCl_3_) δ (ppm): 180.5 (C=Se), 167.0 (C=O), 133.9 (C_aryl_), 131.56 (C_aryl_), 129.33 (C_aryl_), 127.58 (C_aryl_), 50.82 (CH_2_), 21.63 (CH_2_), 11.53 (CH_3_). ^77^Se NMR (76 MHz, CDCl_3_) δ (ppm): 336. Purity (^1^H q-NMR): 99.1%. Elemental analysis for C_11_H_14_N_2_OSe, calculated/found (percent): C, 49.08/49.21; H, 5.24/5.15; N, 10.41/10.25.

***N-(propylcarbamoselenoyl)cinnamamide* (6.I)**. It was obtained from cinnamoyl chloride as a yellow solid in 10% yield; mp: 125–126 °C. ^1^H NMR (400 MHz, CDCl_3_) δ (ppm): 11.29 (s, 1H, NH), 9.38 (s, 1H, NH), 7.79 (d, 1H, *J* = 15.6 Hz, H_alkene_), 7.58–7.53 (m, 2H, H_aryl_), 7.45–7.39 (m, 3H, H_aryl_), 6.55 (d, 1H, *J* = 15.6 Hz, H_alkene_), 3.71 (td, 2H, *J* = 7.2 Hz, *J* = 5.4 Hz, CH_2_), 1.82–1.71 (m, 2H, CH_2_), 1.03 (t, 3H, *J* = 7.4 Hz, CH_3_). ^13^C NMR (101 MHz, CDCl_3_) δ (ppm): 180.4 (C=Se), 165.9 (C=O), 146.7 (C_alkene_), 133.8 (C_aryl_), 131.3 (C_aryl_), 129.3 (C_aryl_), 128.68 (C_aryl_), 118.3 (C_alkene_), 50.61 (CH_2_), 21.68 (CH_2_), 11.54 (CH_3_). ^77^Se NMR (76 MHz, CDCl_3_) δ (ppm): 323. Purity (^1^H q-NMR): 95.0%. Elemental analysis for C_13_H_16_N_2_OSe, calculated/found (percent): C, 52.88/53.03; H, 5.46/5.33; N, 9.49/9.61.

***N-(allylcarbamoselenoyl)furan-2-carboxamide* (1.II)**. It was obtained from furan-2-carbonyl chloride as a yellow crystal in 21% yield; mp: 86–87 °C. ^1^H NMR (400 MHz, CDCl_3_) δ (ppm): 11.02 (s, 1H, NH), 9.41 (s, 1H, NH), 7.61 (dd, 1H, *J* = 1.7 Hz, *J* = 0.8 Hz, H_furan_), 7.35 (dd, 1H, *J* = 3.6 Hz, *J* = 0.8 Hz, H_furan_), 6.62 (dd, 1H, *J* = 3.6 Hz, *J* = 1.7 Hz, H_furan_), 5.97 (ddt, 1H, *J* = 17.3 Hz, *J* = 10.3 Hz, *J* = 5.7 Hz, H_alkene_), 5.34 (dtd, 1H, *J* = 17.3 Hz, *J* = 1.6 Hz, H_alkene_), 5.28 (dq, 1H, *J* = 10.3 Hz, *J* = 1.6 Hz, H_alkene_), 4.39 (tt, 2H, *J* = 5.6 Hz, *J* = 1.6 Hz, CH_2_). ^13^C NMR (100 MHz, CDCl_3_) δ (ppm): 180.9 (C=Se), 156.7 (C=O), 146.6 (C_furan_), 144.9 (C_furan_), 131.5 (C_alkene_), 119.2 (C_furan_), 118.29 (C_alkene_), 113.56 (C_furan_), 51.35 (CH_2_). ^77^Se NMR (76 MHz, CDCl_3_) δ (ppm) 356. Purity (^1^H q-NMR): 98.9%. Elemental analysis for C_9_H_10_N_2_O_2_Se, calculated/found (percent): C, 42.04/41.89; H, 3.92/3.88; N, 10.89/10.81.

***N-(allylcarbamoselenoyl)thiophene-2-carboxamide* (2.II)**. It was obtained from thiophene-2-carbonyl chloride as a yellow solid in 34% yield; mp: 81–82 °C. ^1^H NMR (400 MHz, CDCl_3_) δ (ppm): 11.09 (s, 1H, NH), 9.19 (s, 1H, NH), 7.78— 7.62 (m, 2H, H_thiophen_), 7.17 (dd, 1H, *J* = 4.7 Hz, *J* = 3.8 Hz, H_thiophen_), 5.96 (ddt, 1H, *J* = 17.2 Hz, *J* = 10.3 Hz, *J* = 5.7 Hz, H_alkene_), 5.34 (dq, 1H, *J* = 17.2 Hz, *J* = 1.6 Hz, H_alkene_), 5.27 (dq, 1H, *J* = 10.3 Hz, *J* = 1.6 Hz, H_alkene_), 4.38 (tt, 2H, *J* = 5.6 Hz, *J* = 1.6 Hz, CH_2_). ^13^C NMR (100 MHz, CDCl_3_) δ (ppm): 180.8 (C=Se), 161.1 (C=O), 135.8 (C_thiophen_), 134.5 (C_thiophen_), 131.4 (C_alkene_), 130.9 (C_thiophen_), 128.7 (C_thiophen_), 118.3 (C_alkene_), 51.4 (CH_2_). ^77^Se NMR (76 MHz, CDCl_3_) δ (ppm): 349.65. Purity (^1^H q-NMR): 97.2%. Elemental analysis for C_9_H_10_N_2_OSSe, calculated/found (percent): C, 39.56/39.49; H, 3.69/3.61; N, 10.25/10.03.

***(E)-N-(allylcarbamoselenoyl)-3-(furan-2-yl)acrylamide* (3.II)**. It was obtained from 3-(2-furyl)acrylic acid as an orange solid in 21% yield; m.p: 104–105 °C. ^1^H NMR (400 MHz, CDCl_3_) δ (ppm): 11.29 (s, 1H, NH), 9.17 (s, 1H, NH), 7.58–7.49 (m, 2H, H_furan_ + H_alkene_ ), 6.72 (d, 1H, *J* = 3.5 Hz, H_furan_), 6.50 (dd, 1H, *J* = 3.5 Hz, *J* = 1.8 Hz, H_furan_), 6.33 (d, 1H, *J* = 15.2 Hz, H_alkene_), 5.96 (ddt, 1H, *J* = 17.3 Hz, *J* = 10.4 Hz, *J* = 5.7 Hz, H_alkene_), 5.36–5.23 (m, 2H, H_alkene_), 4.37 (tt, 2H, *J* = 5.6 Hz, *J* = 1.6 Hz, CH_2_). ^13^C NMR (100 MHz, CDCl_3_) δ (ppm): 181.0 (C=Se), 165.7 (C=O), 150.7 (C_furan_), 145.9 (C_furan_), 132.7 (C_alkene_), 131.6 (C_alkene_), 118.2 (C_alkene_), 117.2 (C_furan_), 115.4 (C_alkene_), 112.9 (C_furan_), 51.2 (CH_2_). ^77^Se NMR (76 MHz, CDCl_3_) δ (ppm): 333. Purity (^1^H q-NMR): 96.4%. Elemental analysis for C_11_H_12_N_2_O_2_Se, calculated/found (percent): C, 46.65/46.86; H, 4.27/4.08; N, 9.89/9.94.

***(E)-N-(allylcarbamoselenoyl)-3-(thiophen-2-yl)acrylamide* (4.II)**. It was obtained from 3-(2-thienyl)acrylic acid as an orange solid in 14% yield; m.p: 104–105 °C. ^1^H NMR (400 MHz, CDCl_3_) δ (ppm): 11.28 (s, 1H, NH), 9.24 (s, 1H, NH), 7.89 (d, 1H, *J* = 15.2 Hz, H_alkene_), 7.46 (dt, 1H, *J* = 5.1 Hz, *J* = 1.1 Hz, H_thiophen_), 7.34 (dd, 1H, *J* = 3.7 Hz, *J* = 1.1 Hz, H_thiophen_), 7.09 (dd, 1H, *J* = 5.1 Hz, *J* = 3.7 Hz, H_thiophen_), 6.29 (d, 1H, *J* = 15.2 Hz, H_alkene_), 5.97 (ddt, 1H, *J* = 17.3 Hz, *J* = 10.3 Hz, *J* = 5.6 Hz, H_alkene_), 5.37–5.24 (m, 2H, H_alkene_), 4.38 (tt, 2H, *J* = 5.6 Hz, *J* = 1.6 Hz, CH_2_). ^13^C NMR (100 MHz, CDCl_3_) δ (ppm): 181.0 (C=Se), 165.6 (C=O), 139.2 (C_alkene_), 139.1 (C_thiophen_), 132.6 (C_thiophen_), 131.6 (C_alkene_), 130.2 (C_thiophen_), 128.6 (C_thiophen_), 118.2 (C_alkene_), 116.6 (C_alkene_), 51.2 (CH_2_). ^77^Se NMR (76 MHz, CDCl_3_) δ (ppm): 334. Purity (^1^H q-NMR): 96.3%. Elemental analysis for C_11_H_12_N_2_OSSe, calculated/found (percent): C, 44.15/44.23; H, 4.04/4.12; N, 9.36/9.26.

***N-(allylcarbamoselenoyl)benzamide* (5.II)**. It was obtained from benzoyl chloride as a brown solid in 24% yield; mp: 62–63 °C. ^1^H NMR (400 MHz, CDCl_3_) δ (ppm): 11.30 (s, 1H, NH), 9.35 (s, 1H, NH), 7.89–7.82 (m, 2H, H_aryl_), 7.67–7.61 (m, 1H, H_aryl_), 7.57–7.48 (m, 2H, H_aryl_), 5.99 (ddt, 1H, *J* = 17.2 Hz, *J* = 10.3 Hz, *J* = 5.6 Hz, H_alkene_), 5.36 (dtd, 1H, *J* = 17.2 Hz, *J* = 1.7 Hz, *J* = 1.2 Hz, H_alkene_), 5.29 (dq, 1H, *J* = 10.4 Hz, *J* = 1.2 Hz, H_alkene_), 4.41 (tt, 2H, *J* = 5.6 Hz, *J* = 1.7 Hz, CH_2_). ^13^C NMR (100 MHz, CDCl_3_) δ (ppm): 181.2 (C=Se), 167.0 (C=O), 133.9 (C_aryl_), 131.5 (C_alkene_), 131.5 (C_aryl_), 129.4 (C_aryl_), 127.6 (C_aryl_), 118.4 (C_alkene_), 51.4 (CH_2_). ^77^Se NMR (76 MHz, CDCl_3_) δ (ppm): 346. Purity (^1^H q-NMR): 97.0%. Elemental analysis for C_11_H_12_N_2_OSe, calculated/found (percent): C, 49.45/49.29; H, 4.53/4.59; N, 10.48/10.35.

***N-(allylcarbamoselenoyl)cinnamamide* (6.II)**. It was obtained from cinnamoyl chloride as an orange solid in 17% yield; mp: 114–115 °C. ^1^H NMR (400 MHz, CDCl_3_) δ (ppm): 11.32 (s, 1H, NH), 9.40 (s, 1H, NH), 7.79 (d, 1H, *J* = 15.6 Hz, H_alkene_), 7.55 (dd, 2H, *J* = 7.5 Hz, *J* = 2.1 Hz, H_aryl_), 7.45–7.39 (m, 3H, H_aryl_), 6.53 (d, 1H, *J* = 15.5 Hz, H_alkene_), 5.97 (ddt, 1H, *J* = 17.3 Hz, *J* = 10.4 Hz, *J* = 5.7 Hz, H_alkene_), 5.38–5.25 (m, 2H, H_alkene_), 4.40 (tt, 2H, *J* = 5.5 Hz, *J* = 1.6 Hz, CH_2_). ^13^C NMR (100 MHz, CDCl_3_) δ (ppm): 181.0 (C=Se), 165.8 (C=O), 146.9 (C_alkene_), 133.8 (C_aryl_), 131.5 (C_alkene_), 131.4 (C_aryl_), 129.3 (C_aryl_), 128.7 (C_aryl_), 118.2 (C_alkene_), 118.1 (C_alkene_), 51.2 (CH_2_). ^77^Se NMR (76 MHz, CDCl_3_) δ (ppm): 332. Purity (^1^H q-NMR): 95.7%. Elemental analysis for C_13_H_14_N_2_OSe, calculated/found (percent): C, 53.25/52.99; H, 4.81/4.73; N, 9.55/9.42.

***N-(allylcarbamoselenoyl)nicotinamide* (7.II)**. It was obtained from nicotinic acid as an orange solid in 10% yield; mp: 103–104 °C. ^1^H NMR (400 MHz, CDCl_3_) δ (ppm): 11.16 (s, NH), 9.47 (s, NH), 9.10 (dd, 1H, *J* = 2.4 Hz, *J* = 0.9 Hz, H_pyridine_), 8.86 (dd, 1H, *J* = 4.8 Hz, *J* = 1.7 Hz, H_pyridine_), 8.14 (ddd, 1H, *J* = 8.0 Hz, *J* = 2.4 Hz, *J* = 1.7 Hz, H_pyridine_), 7.47 (ddd, 1H, *J* = 8.0 Hz, *J* = 4.9 Hz, *J* = 0.9 Hz, H_pyridine_), 5.98 (ddt, 1H, *J* = 17.3 Hz, *J* = 10.3 Hz, *J* = 5.7 Hz, H_alkene_), 5.36 (dq, 1H, *J* = 17.3 Hz, *J* = 1.6 Hz, H_alkene_), 5.30 (dq, 1H, *J* = 10.3 Hz, *J* = 1.3 Hz, H_alkene_), 4.40 (tt, 2H, *J* = 5.6 Hz, *J* = 1.5 Hz, CH_2_). ^13^C NMR (100 MHz, CDCl_3_) δ (ppm): 181.0 (C=Se), 165.4 (C=O), 154.3 (C_pyridine_), 148.9 (C_pyridine_), 135.3 (C_pyridine_), 131.3 (C_alkene_), 127.6 (C_pyridine_), 123.9 (C_pyridine_), 118.6 (C_alkene_), 51.5 (CH_2_). ^77^Se NMR (76 MHz, CDCl_3_) δ (ppm): 364. Purity (^1^H q-NMR): 96.2%. Elemental analysis for C_10_H_11_N_3_OSe, calculated/found (percent): C, 44.79/44.85; H, 4.13/4.19; N, 15.67/15.78.

***N-(allylcarbamoselenoyl)benzo[d][1,3]dioxole-5-carboxamide* (8.II)**. It was obtained from piperonylic acid as a light orange solid in 15% yield; m.p: 105–106 °C. ^1^H NMR (400 MHz, CDCl_3_) δ (ppm): 11.27 (s, 1H, NH), 9.21 (s, 1H, NH), 7.40 (dd, 1H, *J* = 8.2 Hz, *J* = 1.9 Hz, H_aryl_), 7.31 (d, 1H, *J* = 1.9 Hz, H_aryl_), 6.89 (d, 1H, *J* = 8.2 Hz, H_aryl_), 6.09 (s, 2H, CH_2-benzodioxole_), 5.98 (ddt, 1H, *J* = 17.3 Hz, *J* = 10.4 Hz, *J* = 5.6 Hz, H_alkene_), 5.35 (dq, 1H, *J* = 17.3 Hz, *J* = 1.5 Hz, H_alkene_), 5.28 (dq, 1H, *J* = 10.4 Hz, *J* = 1.5 Hz, H_alkene_), 4.39 (tt, 2H, *J* = 5.6 Hz, *J* = 1.5 Hz, CH_2_). ^13^C NMR (100 MHz, CDCl_3_) δ (ppm): 181.1 (C=Se), 166.1 (C=O), 152.6 (C_aryl_), 148.8 (C_aryl_), 131.5 (C_alkene_), 125.3 (C_aryl_), 123.2 (C_aryl_), 118.3 (C_alkene_), 108.6 (C_aryl_), 107.9 (C_aryl_), 102.5 (CH_2-benzodioxole_), 51.4 (CH_2_). ^77^Se NMR (76 MHz, CDCl_3_) δ (ppm): 340. Purity (^1^H q-NMR): 98.8%. Elemental analysis for C_12_H_12_N_2_O_3_Se, calculated/found (percent): C, 46.31/46.15; H, 3.89/3.78; N, 9.00/8.88.

***(E)-N-(allylcarbamoselenoyl)-3-(benzo[d][1,3]dioxol-5-yl)acrylamide* (9.II)**. It was obtained from 3,4-(methylenedioxy)cinnamic acid as a brown solid in 29% yield; m.p: 187–188 °C. ^1^H NMR (400 MHz, DMSO-*d*_6_) δ (ppm): 11.48 (s, 1H, NH), 11.41 (t, 1H, *J* = 5.7 Hz, NH), 7.64 (d, 1H, *J* = 15.6 Hz, H_alkene_), 7.19–7.11 (m, 2H, H_aryl_), 7.01 (d, 1H, *J* = 8.0 Hz, H_aryl_), 6.88 (d, 1H, *J* = 15.7 Hz, H_alkene_), 6.10 (s, 2H, CH_2-benzodioxole_), 6.01–5.90 (m, 1H, H_alkene_), 5.28–5.17 (m, 2H, H_alkene_), 4.32 (tt, 2H, *J* = 5.5 Hz, *J* = 1.7 Hz, CH_2_). ^13^C NMR (100 MHz, DMSO-*d*_6_) δ (ppm): 181.1 (C=Se), 166.7 (C=O), 150.2 (C_aryl_), 148.6 (C_aryl_), 144.9 (C_alkene_), 133.3 (C_alkene_), 129.0 (C_aryl_), 125.2 (C_aryl_), 118.1 (C_alkene_), 117.2 (C_alkene_), 109.3 (C_aryl_), 107.0 (C_aryl_), 102.3 (CH_2-benzodioxole_), 50.1 (CH_2_). ^77^Se NMR (76 MHz, DMSO) δ (ppm): 334. Purity (^1^H q-NMR): 96.0%. Elemental analysis for C_14_H_14_N_2_O_3_Se, calculated/found (percent): C, 49.86/50.02; H, 4.18/4.26; N, 8.31/8.19.

***N^1^,N^3^-bis(allylcarbamoselenoyl)isophthalamide* (10.II)**. It was obtained from isophthaloyl chloride as a light-yellow solid in 10% yield; m.p: 184–185 °C. ^1^H NMR (400 MHz, CDCl_3_) δ (ppm): 11.25 (s, 2H, NH), 9.99 (s, 2H, NH), 8.41 (d, 1H, *J* = 1.9 Hz, H_aryl_), 8.12 (dd, 2H, *J* = 7.8 Hz, *J* = 1.9 Hz, H_aryl_), 7.70 (t, 1H, *J* = 7.8 Hz, H_aryl_), 5.98 (ddt, 2H, *J* = 16.4 Hz, *J* = 10.9 Hz, *J* = 5.7 Hz, H_alkene_), 5.41–5.23 (m, 4H, H_alkene_), 4.39 (t, 4H, *J* = 5.5 Hz, CH_2_). ^13^C NMR (100 MHz, CDCl_3_) δ (ppm): 181.0 (C=Se), 166.0 (C=O), 133.0 (C_aryl_), 132.4 (C_aryl_), 131.3 (C_alkene_), 130.3 (C_aryl_), 127.2 (C_aryl_), 118.6 (C_alkene_), 51.4 (CH_2_). ^77^Se NMR (76 MHz, CDCl_3_) δ (ppm): 350. Purity (^1^H q-NMR): 97.6%. Elemental analysis for C_16_H_18_N_4_O_2_Se_2_, calculated/found (percent): C, 42.12/42.28; H, 3.98/4.08; N, 12.28/12.35.

***N^1^-allyl-N^3^-(allylcarbamoselenoyl)isophthalamide* (11.II)**. It was obtained from isophthaloyl chloride as a by-product in 20% yield, and it was a white solid; m.p: 152–153 °C. ^1^H NMR (400 MHz, CDCl_3_) δ (ppm): 11.24 (s, 1H, NH_acylselenourea_), 9.47 (s, 1H, NH_acylselenourea_), 8.27 (t, 1H, *J* = 1.8 Hz, H_aryl_), 8.07 (dt, 1H, *J* = 7.8 Hz, *J* = 1.2 Hz, H_aryl_), 7.98 (dt, 1H, *J* = 7.8 Hz, *J* = 1.3 Hz, H_aryl_), 7.61 (t, 1H, *J* = 7.8 Hz, H_aryl_), 6.43 (s, 1H, NH_amide_), 5.96 (dddt, 2H, *J* = 19.5 Hz, *J* = 17.2 Hz, *J* = 10.2 Hz, *J* = 5.7 Hz, H_alkene_), 5.40–5.17 (m, 4H, H_alkene_), 4.40 (tt, 2H, *J* = 5.6 Hz, *J* = 1.6 Hz, CH_2-acylselenourea_), 4.11 (tt, 2H, *J* = 5.8 Hz, *J* = 1.5 Hz, CH_2-amide_). ^13^C NMR (100 MHz, CDCl_3_) δ (ppm): 181.1 (C=Se), 166.2 (C=O_acylselenourea_), 165.9 (C=O_amide_), 135.8 (C_aryl_), 133.8 (C_aryl_), 132.4 (C_aryl_), 131.96 (C_aryl_), 131.36 (C_alkene_), 130.45 (C_alkene_), 129.80 (C_aryl_), 126.16 (C_aryl_), 118.47 (C_alkene_), 117.45 (C_alkene_), 51.42 (CH_2-acylselenourea_), 42.85 (CH_2-amide_). ^77^Se NMR (76 MHz, CDCl_3_) δ (ppm): 354. Purity (^1^H q-NMR): 99.9%. Elemental analysis for C_15_H_17_N_3_O_2_Se, calculated/found (percent): C, 51.43/51.29; H, 4.89/4.95; N, 12.00/11.87.

***N-(prop-2-in-1-ylcarbamoselenoyl)furan-2-carboxamide* (1.III)**. It was obtained from furan-2-carbonyl chloride as a yellow solid in 28% yield; mp: 113–114 °C. ^1^H NMR (400 MHz, CDCl_3_) δ (ppm): 11.07 (s, 1H, NH), 9.45 (s, 1H, NH), 7.61 (dd, 1H, *J* = 1.7 Hz, *J* = 0.8 Hz, H_furan_), 7.37 (dd, 2H, *J* = 3.6 Hz, *J* = 0.8 Hz, H_furan_), 6.62 (dd, 1H, *J* = 3.6 Hz, *J* = 1.7 Hz, H_furan_), 4.52 (dd, 2H, *J* = 5.0 Hz, *J* = 2.6 Hz, CH_2_), 2.37 (t, 1H, *J* = 2.6 Hz, CH). ^13^C NMR (100 MHz, CDCl_3_) δ (ppm): 181.4 (C=O), 156.6 (C=Se), 146.7 (C_furan_), 144.7 (C_furan_), 119.4 (C_furan_), 113.6 (C_furan_), 77.1 (C), 73.5 (CH), 38.7 (CH_2_). ^77^Se NMR (76 MHz, CDCl_3_) δ (ppm): 379. Purity (^1^H q-NMR): 96.7%. Elemental analysis for C_9_H_8_N_2_O_2_Se, calculated/found (percent): C, 42.37/42.31; H, 3.16/3.02; N, 10.98/10.89.

***N-(prop-2-in-1-ylcarbamoselenoyl)thiophene-2-carboxamide* (2.III)**. It was obtained from thiophene-2-carbonyl chloride as a yellow solid in 94% yield; mp: 105–106 °C. ^1^H NMR (400 MHz, CDCl_3_) δ (ppm): 11.15 (s, 1H, NH), 9.22 (s, 1H, NH), 7.73 (dd, 1H, *J* = 5.0 Hz, *J* = 1.1 Hz, H_thiophen_), 7.71 (dd, 1H, *J* = 3.9 Hz, *J* = 1.1 Hz, H_thiophen_), 7.18 (dd, 1H, *J* = 5.0 Hz, *J* = 3.9 Hz, H_thiophen_), 4.52 (dd, 2H, *J* = 5.0 Hz, *J* = 2.6 Hz, CH_2_), 2.37 (t, 1H, *J* = 2.6 Hz, CH). ^13^C NMR (100 MHz, CDCl_3_) δ (ppm): 181.4 (C=Se), 161.1 (C=O), 135.6 (C_thiophen_), 134.8 (C_thiophen_), 131.1 (C_thiophen_), 128.8 (C_thiophen_), 77.0 (C), 73.5 (CH), 38.8 (CH_2_). ^77^Se NMR (76 MHz, CDCl_3_) δ (ppm): 372. Purity (^1^H q-NMR): 99.8%. Elemental analysis for C_9_H_8_N_2_OSSe, calculated/found (percent): C, 39.86/39.99; H, 2.97/3.06; N, 10.33/10.41.

***(E)-3-(furan-2-yl)-N-(prop-2-in-1-ylcarbamoselenoyl)acrylamide* (3.III)**. It was obtained from cinnamoyl chloride as an orange solid in 15% yield; mp: 118–119 °C. ^1^H NMR (400 MHz, CDCl_3_) δ (ppm): 11.33 (s, 1H, NH), 9.18 (s, 1H, NH), 7.55 (d, 1H, *J* = 15.3 Hz, H_alkene_), 7.54 (d, 1H, *J* = 1.8 Hz, H_furan_), 6.73 (d, 1H, *J* = 3.4 Hz, H_furan_), 6.51 (dd, 1H, *J* = 3.4, *J* = 1.8 Hz, H_furan_), 6.31 (d, 1H, *J* = 15.3 Hz, H_alkene_), 4.51 (dd, 2H, *J* = 5.0 Hz, *J* = 2.6 Hz, CH_2_), 2.36 (t, 1H, *J* = 2.6 Hz, CH). ^13^C NMR (100 MHz, CDCl_3_) δ (ppm): 181.6 (C=Se), 165.7 (C=O), 150.7 (C_furan_), 146.0 (C_furan_), 132.9 (C_alkene_), 117.4 (C_furan_), 115.2 (C_furan_), 113.0 (C_alkene_), 73.3 (CH), 38.5 (CH_2_). ^77^Se NMR (76 MHz, CDCl3) δ (ppm): 356. Purity (^1^H q-NMR): 95.0%. Elemental analysis for C_11_H_10_N_2_O_2_Se, calculated/found (percent): C, 46.99/46.84; H, 3.58/3.66; N, 9.96/9.86.

***N-(prop-2-in-1-ylcarbamoselenoyl)benzamide* (5.III)**. It was obtained from benzoyl chloride as a yellow solid in 26% yield; mp: 98–99 °C. ^1^H NMR (400 MHz, CDCl_3_) δ (ppm): 11.34 (s, 1H, NH), 9.38 (s, 1H, NH), 7.88–7.82 (m, 2H, H_aryl_), 7.64 (d, 1H, *J* = 7.3 Hz, H_aryl_), 7.53 (dd, 2H, *J* = 8.4 Hz, *J* = 7.3 Hz, H_aryl_), 4.54 (dd, 2H, *J* = 5.0 Hz, *J* = 2.6 Hz, CH_2_), 2.39 (t, 1H, *J* = 2.6 Hz, CH). ^13^C NMR (100 MHz, CDCl_3_) δ (ppm) 181.8 (C=Se), 167 (C=O), 134.0 (C_aryl_), 131.3 (C_aryl_), 129.4 (C_aryl_), 127.7 (C_aryl_), 77.1 (C) 73.5 (CH), 38.7 (CH_2_). ^77^Se NMR (76 MHz, CDCl_3_) δ (ppm): 369. Purity (^1^H q-NMR): 95.6%. Elemental analysis for C_11_H_10_N_2_OSe, calculated/found (percent): C, 49.82/50.03; H, 3.80/3.69; N, 10.56/10.65.

***N-(prop-2-in-1-ylcarbamoselenoyl)benzo[d]*[1,3]*dioxole-5-carboxamide* (8.III)**. It was obtained from piperonylic acid as an orange solid in 46% yield; m.p: 140–141 °C. ^1^H NMR (400 MHz, CDCl_3_) δ (ppm): 11.32 (s, 1H, NH), 9.24 (s, 1H, NH), 7.40 (dd, 1H, *J* = 8.2 Hz, *J* = 1.9 Hz, H_aryl_), 7.31 (d, 1H, *J* = 1.9 Hz, H_aryl_), 6.90 (d, 1H, *J* = 8.2 Hz, H_aryl_), 6.09 (s, 2H, CH_2-benzodioxole_), 4.53 (dd, 2H, *J* = 5.0 Hz, *J* = 2.6 Hz, CH_2_), 2.38 (t, 1H, *J* = 2.6 Hz, CH). ^13^C NMR (100 MHz, CDCl_3_) δ (ppm): 181.7 (C=O), 166.0 (C=Se), 152.7 (C_aryl_), 148.9 (C_aryl_), 125.2 (C_aryl_), 123.2 (C_aryl_), 108.7 (C_aryl_), 108.0 (C_aryl_), 102.5 (CH_2-benzodioxole_), 73.4 (C), 77.2 (CH), 38.7 (CH_2_). ^77^Se NMR (76 MHz, CDCl_3_) δ (ppm): 363. Purity (^1^H q-NMR): 95.0%. Elemental analysis for C_12_H_10_N_2_O_3_Se, calculated/found (percent): C, 46.62/46.81; H, 3.26/3.33; N, 9.06/9.14.

### 2.2. Radical Scavenging Activity

Different methods are available to quantify the antioxidant capacity of natural or novel molecules. Notable among these are the ABTS^•+^ and DPPH^•^ methods, which measure the quenching efficiency in vitro for known concentrations of preformed radicals. In this work, the DPPH^•^ colorimetric assay was carried out. Measurements were recorded on a BioTeck PowerWave XS spectrophotometer (BioTeck Instruments, Winooski, VT, USA) and data were collected using KCJunior v.1.41. software (BioTeck Instruments, Winooski, VT, USA). Compounds with potent activity were selected to further determine their protective effect against ROS-induced cell death caused by H_2_O_2_. 

#### DPPH Radical Scavenging Assay 

DPPH colorimetric assay was performed to determine the in vitro radical scavenging activity of the acylselenourea derivatives. DPPH assay was performed as described by Svinyarov [14]. For each acylselenourea, a stock solution of 1 mg/mL in dimethyl sulfoxide (DMSO) was made, and these solutions were used to prepare the different concentrations employed in the assay. The compounds were tested at two different concentrations (0.003 and 0.03 mg/mL). Intermediate solutions were prepared in methanol and a volume of 100 µL of each was added to each well of the plate. Then, 100 µL of DPPH^•^ (previously prepared at a concentration of 0.04 mg/mL in methanol and preserved in the dark) was added to the previous methanolic acylselenourea solutions. The discoloration of the purple radical to the yellow reduced form was followed at 517 nm at different time points (0, 5, 15, 30, 60, 90, and 120 min). Ascorbic acid and trolox were used as positive controls. All measurements were carried out in triplicate. Results are expressed as the percentage of the radicals scavenged, calculated using the following formula:

(1) $$\;\%\;\mathrm{DPPH}\;\mathrm{radical}\;\mathrm{scavenging}=\frac{A_{\mathrm{control}}{\;-\;A}_{\mathrm{sample}}}{A_{\mathrm{control}}}\;\times \;100\;$$

where $A_{\mathrm{control}}$ refers to the absorbance of the negative control and $A_{\mathrm{sample}}\;$ refers to the absorbance of the tested compounds. Results are expressed as percentage of DPPH radical scavenging ± SD. 

### 2.3. Biological Evaluation 

#### 2.3.1. Cell Culture Conditions 

Cell lines were purchased from the American Type Culture Collection (ATCC). Cell lines MDA-MB-231, HTB-54, DU-145, and BEAS-2B were cultured in RPMI 1640 medium (Gibco, Thermo Fisher Scientific, Gaithersburg, MD, USA) supplemented with 10% fetal bovine serum (FBS; Gibco), 100 units/mL penicillin, and 100 mg/mL streptomycin (Gibco). HT-29 cell line was grown in McCoy’s 5A (Gibco), 10% FBS, 100 units/mL penicillin, and 100 μg/mL streptomycin. Cells were maintained at 37 °C and 5% CO_2_. The culture medium was changed every three days. 

#### 2.3.2. Cell Viability Assay 

The cell viability of each compound was measured by MTT colorimetric assay [15]. Each compound was dissolved in DMSO at a concentration of 0.01 M. Each compound was tested at two different concentrations (10 and 50 µM) in MDA-MB-231 (breast), HTB-54 (lung), DU-145 (prostate), and HT-29 (colon) human cancer cell lines. Serial dilutions were prepared with supplemented culture medium. Additionally, the most active compounds (those that showed less than 50% cell growth at 10 µM in two of the four lines evaluated) were further evaluated at seven different concentrations (1, 2.5, 5, 10, 25, 50 and 100 µM) in HTB-54, DU-145, and MDA-MB-231 cancer cell lines and non-malignant BEAS-2B lung cell line. IC_50_ and selectivity indexes (SI) were determined. 

Briefly, 1 × 10^4^ tumor cells per well were grown in 96-well plates for 24 h. Then, these cells were incubated for 48 h with either DMSO (control) or a different concentration of each of the tested compounds. The cell viability was determined by adding 20 μL of MTT two hours and a half before the termination point. After this time, the culture medium was removed. The resulting formazan crystals were dissolved in 50 μL of DMSO, and the absorbance was measured at 570 nm. Results are expressed as % cell growth. The data were obtained from at least three independent experiments performed in quadruplicate. 

#### 2.3.3. Protective Effects against H_2_O_2_-Induced Oxidative Stress in DU-145 Cells 

Cells were treated with the selected compounds and then oxidative damage to DU-145 cells was induced using different concentrations of H_2_O_2_ (200, 250 and 300 µM), and cell viability was assessed by the MTT method. Briefly, 2.5 × 10^4^ DU-145 cells per well were added in 96-well plates. After 24 h of incubation, DMSO, **1.I**, **2.I**, **5.I**, **7.II**, **10.II**, and ascorbic acid were added to each well at two different concentrations (0.0015 mg/mL and 0.0003 mg/mL) and incubated for 1 h. After that, H_2_O_2_ was added at the concentrations of 200, 250, and 300 µM, and the cells were incubated for 12 h to induce cell damage. MTT assay was used to measure cell viability. For each treatment, the mean cell viability was calculated from three independent experiments. The DMSO-treated controls were assigned with a cell viability value of 100% (V_control_). The results are expressed as a cell growth inhibition % caused by H_2_O_2_ and the increased fold of cell survival, as follows:

- Cells without compounds, only treated with H_2_O_2_:

(2) $$\%\;\mathrm{Cell}\;\mathrm{growth}\;\mathrm{inhibition}=\frac{A_{\mathrm{control}}{\;-\;A}_{\mathrm{sample}}}{A_{\mathrm{control}}}\;\times \;100\;$$

- Cells treated with compounds and H_2_O_2_:

(3) $$\%\;\mathrm{Cell}\;\mathrm{growth}\;\mathrm{inhibition}=\frac{A_{\mathrm{sample}\;\left(\mathrm{compound}\right)}{\;-\;A}_{\mathrm{sample}\;\left(\mathrm{compound}+H2O2\right)}}{A_{\mathrm{control}}}\;\times \;100\;$$

(4) $$\mathrm{Increase}-\mathrm{fold}\;\mathrm{of}\;\mathrm{cell}\;\mathrm{survival}=\frac{{\%\;}_{\mathrm{cell}\;\mathrm{growth}\;\mathrm{inhibition}\;\left(H2O2\right)}}{{\%\;}_{\mathrm{cell}\;\mathrm{growth}\;\mathrm{inhibition}\;\left(\mathrm{compound}+\;H2O2\right)}}$$

The standard error of the mean (SEM) was calculated in at least three independent experiments performed in quadruplicates.

#### 2.3.4. Apoptosis Assays 

Induction of apoptosis was assayed using Annexin V & Dead Cell assay kit and Caspase 3/7 assay kit (EMD Millipore, Darmstadt, Germany). DU-145 cells were seeded in 6-well plates at a density of 6 × 10^5^ cells/well and treated either with DMSO (control) or 10 µM of compounds **1.I** and **5.I** and incubated for 24 h. At the end of treatment, cells were harvested and processed with appropriate staining according to the manufacturer’s protocol. For the Caspase 3/7 assay, cells were stained with 5 μL of Muse™ Caspase 3/7 working solution and incubated at 37 °C for 30 min. After incubation, 150 mL of Muse™ Caspase 7-AAD working solution was added and the samples were incubated for another 5 min at room temperature in the dark. For Annexin V assay, cells were stained with 100 μL pf Muse™ Annexin V & Dead Cell Reagent and incubated for 20 min at room temperature in the dark prior to analysis. Samples were further analyzed on a Muse™ Cell Analyzer (Merck Millipore, Darmstadt, Germany) using Muse™ 1.4 software. Data were obtained from at least two independent experiments.

#### 2.3.5. ROS Measurement

Total ROS levels were measured in DU-145 cells treated with **1.I** and **5.I** using the Muse Oxidative Stress Kit (EMD Millipore, Billerica, MA, USA) according to the manufacturer’s protocol. This kit uses Muse oxidative stress reagent to detect ROS inside cells. This kit identifies two different cell populations: ROS(−) cells and ROS(+) cells. In the graph, the ROS(−) population is represented by the M1 peak and the ROS(+) cells by the M2 peak. DU-145 cells treated with H_2_O_2_ (0.9 µM) were used as positive controls to identify M1 and M2 peaks. Percentages of ROS(−) and ROS(+) cells were measured using the Muse Cell Analyzer.

#### 2.3.6. Statistical Analysis

Data were expressed as the mean ± SD (standard deviation) and experiments were performed three times in triplicates unless otherwise specified. IC_50_ values were determined using a non-linear curve regression analysis calculated by OriginPro 9.0.0 software (OriginLab Corporation; Northampton, MA, USA). Two-way analysis of variance (ANOVA) was used to calculate the statistical significance of differences when comparing control and compounds or treatments alone. Data were analyzed using GraphPad Prism version 8.0.2 (GraphPad Software; San Diego, CA, USA), and the statistically significant values (pvalue) for ANOVA analysis were taken as **** *p* < 0.0001, *** *p* < 0.001, ** *p* < 0.01 and * *p* < 0.05.

### 2.4. X-ray Christallography of 1.II

Evaporation of ethyl acetate from the test tube during the chromatography column gave a single crystal of **1.II**. Appropriate crystals were selected for data collection on a Bruker SMART APEX CCD0 diffractometer and mounted on a nylon loop using paratone oil. The crystal was kept at room temperature (~25 °C) during data collection. Using Olex2 [16], the structure was solved with the XS [17] structure solution program using Direct Methods, and refined with the XL [17] refinement package using Least Squares minimization.

## 3. Results

### 3.1. Design

**Figure 2:**
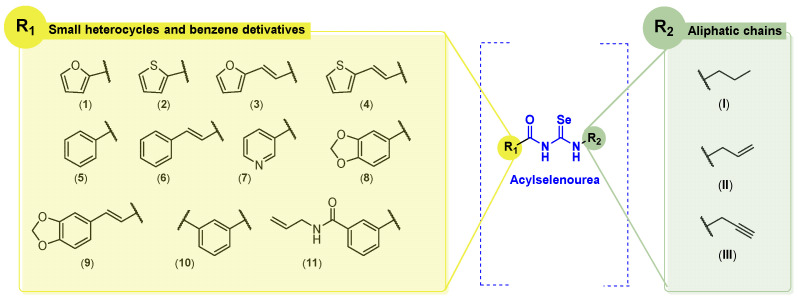
General structures of synthesized compounds.

In our previous work, we developed 39 novel Se-containing compounds combining carbo- and hetero-cycles with active fragments from natural products [18]. The Se atom was introduced as a selenoester functional group in the novel molecules. The results obtained demonstrated low radical scavenger activity. Thus, taking into account all these previous observations, we have synthesized 20 novel derivatives replacing the selenoester functional group by acylselenourea to obtain more potent radical scavengers (Figure 2). 

In the acyl moiety, we envisioned the introduction of different small aromatic cycles, including heterocycles such as thiophene, pyridine, furan, and benzodioxole. According to the FDA, many of chemotherapeutic agents contain heterocycles in their structure [19,20]. In the opposite location of the molecules, aliphatic amines derived from natural products (allyl and propargyl fragments) were included. The allylic motif is present in many bioactive molecules, such as allylic sulfides or allicin in garlic, or eugenol and myristicin in parsley and cloves. These natural allyl derivatives have demonstrated potent anticancer activity in a variety of cancers through different mechanisms of action [21]. On the other hand, the propargyl motif can be found in marine sources, including viridamide, a bioactive compound from marine cyanobacteria, and duryne, a sponge metabolite [22,23]. Strategies to change both sides of the molecule aim to find synergies between the fragment and the acylselenourea itself. In addition to the allylic and propargylic fragments (terminal double and triple bonds, respectively) the synthesized molecules will include the propyl motif (saturated chain). We consider it important to study how chain unsaturation will affect anticancer and antioxidant activity. This work represents the first approach in which aliphatic amines were used for the synthesis of acylselenourea derivatives following similar protocols reported previously for analogous compounds [24,25].

### 3.2. Chemistry

**Scheme 1:**
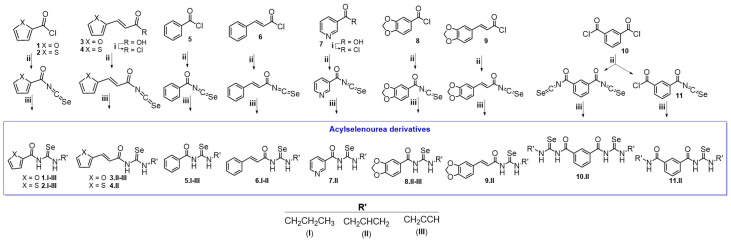
Synthesis of the acylselenourea derivatives. Reagents and conditions: (**i**) ClCOCOCl, *N*,*N*-DMF, CH_2_Cl_2_, 12 h, room temperature; (**ii**) KSeCN, acetone (dry), 1 h, room temperature; (**iii**) NH_2_CH_2_CH_2_CH_3_, NH_2_CH_2_CH_2_ or NH_2_CH_2_CCH, 0.5–24 h, room temperature.

The synthetic procedure of acylselenourea is depicted in Scheme 1. First, the corresponding acyl isoselenocyanate was formed by the reaction of the acyl chloride and potassium selenocyanate for 1 h in dry acetone at room temperature. Then, propylamine (**I**), allylamine (**II**), or propargylamine (**III**) were added to the reaction mixture and stirred for 0.5–24 h at room temperature to obtain the corresponding acylselenourea, with yields ranging from 10 to 94%. The compounds presenting a double bond between the aromatic ring and the carbonyl group were obtained in lower yields compared to those directly linking the aromatic ring and the carbonyl group. This decrease in the yield could be explained by the different stabilization of the reaction intermediate by resonance effect comparing the allylic and benzylic positions. To the best of our knowledge, no acylselenourea derivatives synthesized from aliphatic amine had been reported, all of the reports thus far having used aromatic amines [10,11,26]. All the new compounds were characterized by spectroscopic analysis, including ^1^H-NMR, ^13^C-NMR, ^77^Se-NMR, and 2D-NMR experiments. All spectra recorded for each compound are included in the Supplementary Material (Figures S1–S99).

### 3.3. Antioxidant Activity

Oxidative stress is associated with cancer progression by promoting tumor cell transformation, proliferation, and survival [27]. For this reason, molecules capable of radical scavenging might be developed to treat cancer. Se compounds, such as ebselen, have shown significant antioxidants activity [28,29,30], the latter possessing glutathione peroxidase-like activity. We therefore decided to evaluate the radical scavenging activity of the compounds using the DPPH assay. Compounds exhibiting interesting radical scavenging and anticancer activity were also tested in a prostate cancer cell line (DU-145) to confirm their ability to prevent ROS-induced cell death.

#### DPPH Radical Scavenging Assay

**Figure 3:**
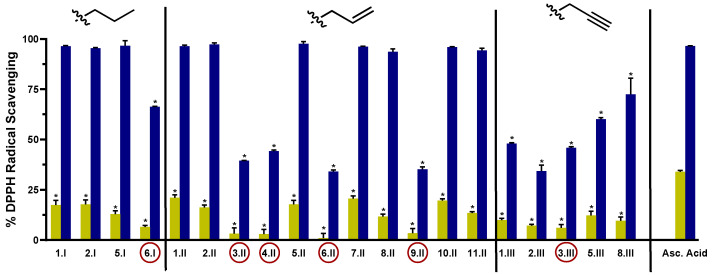
DPPH radical scavenging activity of the novel compounds at 0.003 mg/mL (light green) and 0.03 mg/mL (blue) at 120 min. Compounds with the red circle contain in their structure the double bond between the hetero- or carbo-cycle and the carbonyl of the acylselenourea. Significance: * *p* < 0.0001 compared to the ascorbic acid at the same concentration.

**Figure 4:**
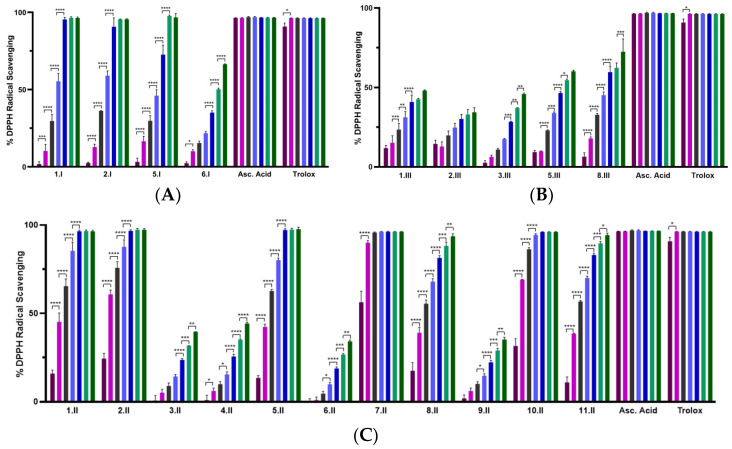
DPPH radical scavenging activity of the novel compounds at 0.03 mg/mL recorded at different points: 0 min (purple), 5 min (pink), 15 min (grey), 30 min (light blue), 60 min (dark blue), 90 min (light green), and 120 min (dark green). (**A**) Percentage of DPPH^•^ scavenging of compounds from series **I** (propyl); (**B**) percentage of DPPH^•^ scavenging of compounds from series **III** (propargyl); (**C**) percentage of DPPH^•^ scavenging of compounds from series **II** (allyl). Significance: * *p* < 0.05, ** *p* ≤ 0.01, *** *p* ≤ 0.001, **** *p* ≤ 0.0001 compared to the previous time point. Results obtained for the 0.003 mg/mL are included in the Supplementary Material (Table S3 and Figure S100).

The DPPH assay was used as a first experimental approach to determine the radical scavenging activity of the synthesized acylselenourea derivatives. Measurements were performed at two different concentrations (0.003 and 0.03 mg/mL) and collected at different time points (0, 5, 15, 30, 60, 90, and 120 min). The results obtained at 120 min for both concentrations are depicted in Figure 3, and the results obtained at the highest concentration at the different time points are presented in Figure 4. The results for the concentration of 0.003 mg/mL are depicted in Table S3. 

As shown in Figure 3, the radical scavenging activity of the compounds was dose-dependent. Moreover, at the highest concentration evaluated (0.03 mg/mL) most of them exhibited time-dependent and similar activity to the positive controls (Figure 4). Comparing between the three series, **I** and **II** showed potent DPPH radical scavenging activity, unlike series **III**. It seems that the propargyl chain (series **III**) caused a loss of inhibitory activity. As shown in Figure 3, all the molecules from series **I** and **II** that do not have a double bond between the carbo- or hetero-cycle and the carbonyl group of the acylselenourea (**6.I**, **3.II**, **4.II**, **6.II**, **9.II** and **3.III**) showed DPPH inhibitory activity similar to ascorbic acid at 120 min. These results seem to be contrary to the study published by Estarad et al. in 2016, where they developed hybrids of cinnamic-benzylpiperidine and cinnamic dibenzyl(*N*-methyl)amine with high antioxidant activity (similar to trolox), despite possessing a cinnamoyl group. However, it appears that this antioxidant capacity depended more on the hydroxyl groups of the benzene ring (phenol groups), since the antioxidant capacity was lost when they were replaced by methoxy groups [31]. These results might be related to the weak acid character of the phenol group, which can yield the hydrogen from the –OH under suitable conditions and scavenge free radicals. In the same way, the propargyl group (series **III**) also possesses an acidic hydrogen (≡C**H**) that could be released and scavenge the DPPH radical. However, the results we have obtained go in the opposite direction, since the propargyl derivatives have shown a lower inhibitory activity. Therefore, it seems that the presence of the acylselenourea moiety may generate a competitive reaction between the hydrogen (–N**H**) from the acylselenourea and the acidic hydrogen (≡C**H**) from the propargyl chain, and slow down the free radical scavenging process.

Compound **7.II**, containing allyl and pyridine groups, demonstrated the fastest kinetics, reaching the highest DPPH inhibition after 5 min of treatment. Likewise, **1.II**, **2.II**, **5.II**, **10.II**, **1.I**, and **2.I** also exhibited a fast kinetic reaching the highest DPPH inhibition value after 60 min. Interestingly, as in our previously reported study [11], acylselenourea derivative containing benzodioxole moiety showed potent radical scavenging activity, but there were other derivatives that exhibited faster kinetics. 

As shown in Figure 3, the novel molecules also exhibited concentration-dependent radical scavenging activity in vitro. At the lowest concentration (0.003 mg/mL), all the compounds showed considerably decreased DPPH inhibition at 120 min, showing lower potency than the positive control (*p* < 0.0001). 

### 3.4. Biological Evaluation

#### 3.4.1. Cytotoxic Activity

**Figure 5:**
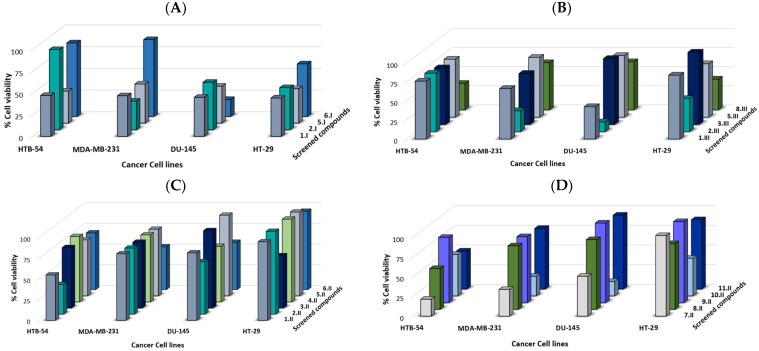
Synthesized compounds reduced cell viability in several human cancer cell lines. Cell growth % at 10 µM after 48 h of treatment of synthesized compounds: (**A**) series I, (**B**) series III, and (**C**), and (**D**) series II.

All the compounds were evaluated against a panel of breast (MDA-MB-231), colon (HT-29), lung (HTB-54), and prostate (DU-145) human tumor cell lines at two different concentrations (10 and 50 µM). The cell viability was determined after 48 h of treatment using MTT assay. The data are expressed as the percentage of cell growth ± SD in at least three independent experiments performed in quadruplicates (Figure 5 and Table S1). 

In general, the synthesized compounds demonstrated a moderate anticancer activity against the panel of four cancer cell lines (Figure 5). Prostate cell line DU-145 was the most sensitive one, since 8 out of 20 compounds exhibited a cell growth < 60% at 10 µM, highlighting compounds **6.I**, **10.II**, and **2.III**, which showed a cell growth < 20%. Overall, molecules from series **I** (propyl) exhibited the most potent anticancer activity against the panel of cancer cell lines. 

The following preliminary structure activity relationship (SAR) could be determined:

- Compounds **5.I** and **6.I** differ only in the presence of a double bond (**6.I**) between the benzene ring and the carbonyl group. This structural modification led to a loss of activity against MDA-MB-231, HT-29, and HTB-54 cell lines, whereas it improved the activity against the prostate DU-145 cell line (cell growth at 10 µM of 42.4% and 19.47% for **5.I** and **6.I**, respectively). For these same derivatives, but of the series **II** (allyl), the introduction of this double bond was also observed to slightly improve the activity in the MDA-MB-231 cell line (cell growth at 10 µM of 80.4% and 51.8% for **5.II** and **6.II**, respectively). Compound **6.II** showed better activity than **5.II** against the DU-145 cell line (cell growth at 10 µM of 57.30% and 97.5%, respectively);
- On the other hand, comparing compounds **5.I**, **5.II**, and **5.III**, which only differ in the aliphatic chain, it was observed that **5.I** (propyl) showed a more potent anticancer activity against the panel of the four cell lines, **5.II** and **5.III** being hardly active;
- Compounds **1.II** and **3.II** contain a furan ring in their structure and their only difference is the double bond (**3.II**) between the heterocycle and carbonyl. However, on this occasion, both showed low activity with cell growth % between 54.8–95.0 at 10 µM. Their series **III** analogues (**1.III** and **3.III**) also showed low-moderate activity for the MDA-MB-231, HT-29, and HTB-54 cell lines. However, the DU-145 cell line proved to be more sensitive to **1.III**, showing a cell growth of 42.2 at 10 µM;
- Compound **1.I** (propyl) showed more potent anticancer activity than its analogues (**1.II** and **1.III**) against the same four cancer cell lines panel. On the other hand, compounds **3.II** and **3.III** did not exhibit great anticancer activity;
- For the thiophene derivatives (**2.II** and **4.II**), the presence of this double bond led to a loss of activity in the lung HTB-54 cell line (cell growth at 10 µM of 35.8% and 79.2% for **2.II** and **4.II**, respectively). For the rest of the cell lines, **2.II** and **4.II** presented moderate-low activity. The presence of the allyl chain in the thiophene analogues (**2.II**) led to a loss of activity compared to propyl and propargyl derivatives (**2.I** and **2.III**);
- Compounds **8.II** and **9.II** are allylic derivatives of benzodioxole that differ in the presence of the double bond between the core and the carbonyl. However, this small structural modification does not seem to affect the anticancer activity against MDA-MB-231, HT-29, and DU-145 cancer cell lines. Molecule **8.II**, the derivative presenting the benzodioxole core directly attached to the carbonyl, was shown to be more potent against the HTB-54 cell line (cell growth at 10 µM of 51.8% and 82.8% for **8.II** and **9.II**, respectively).

Compound **10.II** differs from compound **5.II** in the presence of two acylselenourea functionalities substituted with the allylic chain. Molecule **5.II** showed moderate-low anticancer activity, whereas the addition of another allylic acylselenourea moiety (**10.II**) led to a more potent derivative. Compound **10.II** showed cell growth of 24.8, 47.7, 52.6, and 18.1% against MDA-MB-231, HT-29, HTB-54, and DU-145 cancer cell lines. On the other hand, compound **11.II** differs from compound **10.II** in that instead of presenting two acylselenourea groups, it presents only one together with an amide group. This compound, at 10 µM, showed cell growth higher than 77.0%, evidencing the crucial role played by the acylselenourea functional group in the anticancer activity.

Compounds **1.I**, **2.I**, **5.I**, **7.II**, **10.II**, **2.III**, and **8.III** were further tested at seven different concentrations (1, 2.5, 5, 10, 25, 50 and 100 µM), after 48 h of treatment, in order to establish their dose–response curve in lung HTB-54 cancer cells. The potential selectivity of these compounds was further investigated in a cell line derived from normal lung tissue (BEAS-2B). Results are expressed as IC_50_ and the SI, calculated as the ratio of the IC_50_ values determined for the non-malignant and the tumor cells, respectively (IC_50_ (BEAS-2B)/IC_50_ (HTB-54)). The results are shown in Table 1.

**Figure 6:**
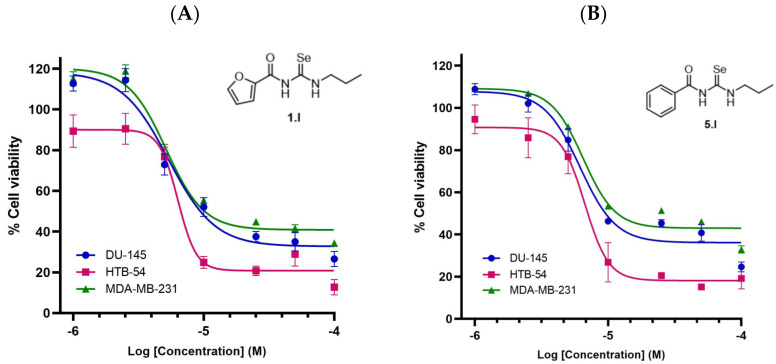
Cytotoxicity of compounds **1.I** and **5.I** in several human cancer cell lines. (**A**) Dose-response curve of compound **1.I**. (**B**) Dose-response curve of compound **5.I**. Calculated IC_50_ values are shown in Table 1.

Compounds **1.I** and **5.I** exhibited the most potent activity with IC_50_ values in lung cancer cells (HTB-54) < 10 µM, along with high SI towards malignant lung cancer cells (SI of >12.7 and >11.9, respectively). On the other hand, compound **2.I**, which contains a thiophene ring with propyl chain, showed high cytotoxicity against normal lung tissue. Derivatives **2.III** and **8.III** from series **III** (propargyl) showed anticancer activity with IC_50_ values of 11.6 and 21.7, respectively, against HTB-54 cells, with **8.III** being more selective towards malignant lung cancer cells (SI of 2.3 and >4.6, respectively). On the other hand, the introduction of the allyl chain (compounds **7.II** and **10.II**) led to potent cytotoxicity against normal lung tissue cells (BEAS-2B) with IC_50_ values of 6.3 and 29.9 µM. Thus, the SI for these compounds was low (0.5 and 0.7, respectively). Additionally, compounds **1.I** and **5.I**, which exhibited the highest SI towards malignant lung cancer cells, were also evaluated against MDA-MB-231 (breast) and DU-145 (prostate) cancer cell lines (Figure 6). Both compounds showed similar IC_50_ values of 8.8 µM and 9.5 µM for compound **1.I**, and 15.5 µM and 11.5 µM for compound **5.I**, against MDA-MB-231 and DU-145, respectively. As shown in Table 1, these two derivatives showed similar IC_50_ values to cisplatin, even better for HTB-54 cells, along with SI > 20 times higher than cisplatin towards normal lung tissue. Cisplatin was chosen as a reference because it is one of the most widely used chemotherapeutic agents in the clinic.

#### 3.4.2. Protective Effects against H_2_O_2_-Induced Cell Damage in DU-145 Cells

**Figure 7:**
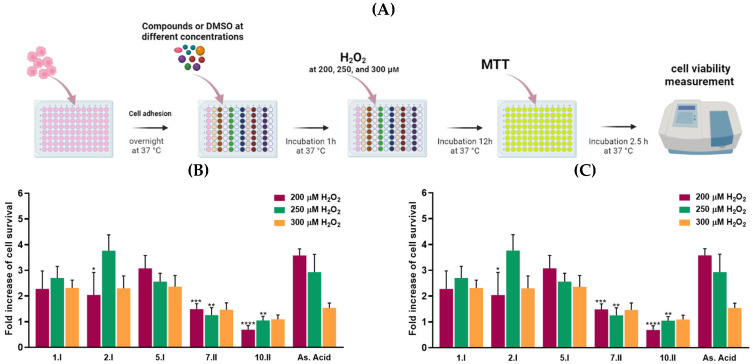
H_2_O_2_ -induced DU-145 cells oxidative damage model. (**A**) Protocol of the assay. (**B**) Radical scavenging activity of hit compounds at 0.0015 mg/mL and (**C**) 0.0003 mg/mL with 200 (purple), 250 (green), and 300 µM (orange) of H_2_O_2_. The results are expressed as a fold increase of cell survival. **** *p* < 0.0001, *** *p* < 0.001, ** *p* < 0.01, * *p* < 0.05 when comparing As. Acid and compounds. Results obtained are included in the Supplementary Material (Tables S4 and S5). Figure 7A was created with BioRender software, ©biorender.com (accessed on 26 March 2023).

Compounds **1.I**, **2.I**, **5.I**, **7.II**, and **10.II** demonstrated the most potent DPPH inhibitory activity, along with cell growth below 55% in at least three of the four cancer cell lines tested. Therefore, they were selected to be evaluated in H_2_O_2_-damaged DU-145 cells at the concentrations of 0.0015 mg/mL and 0.0003 mg/mL using MTT assay (Figure 7). The latter concentrations were chosen because they maintain the antioxidant capacity of the compounds, but they were not toxic to the cells. Likewise, the DU-145 cancer cell line was chosen as it was the most sensitive to the compounds. 

Oxidative damage was induced by three different concentrations of H_2_O_2_: 300, 250, and 200 µM. Whilst these hydrogen peroxide concentrations might not be relevant for mimicking the intracellular ROS levels on in vivo models, they were chosen to induce ample oxidative stress for inducing reasonable levels of cell death.

Propyl derivatives including **1.I**, **2.I**, and **5.I** doubled cell viability at the concentration of 0.0015 mg/mL in DU-145 cells treated with 300, 250, and 200 µM of H_2_O_2_. Compound **2.I** showed the most protective activity against H_2_O_2_-induced oxidative cell damage at the concentration of 0.0015 mg/mL, reaching fold increase values above 3.5. Likewise, compounds **5.I** and **1.I** showed no significant differences at the three different H_2_O_2_ concentrations compared to ascorbic acid. On the other hand, at the lowest concentration (0.0003 mg/mL), none of the compounds exhibited superior activity to ascorbic acid. 

#### 3.4.3. Compounds 1.I and 5.I Induce Apoptosis in DU-145 Cells 

The cell viability assays performed showed that compounds **1.I** and **5.I** exhibited antiproliferative activity and high SI, together with radical scavenging activity. To further confirm if these compounds could induce cell death through apoptosis, we performed the Annexin V Live & Dead Cell assay. During the induction of apoptosis, a progressive series of cellular biochemical and morphological changes occur, including the translocation of phosphatidylserine (PS) from the inside to the outside of the plasma membrane and its exposure to the external surface of the cell. This is a cell-type-independent phenomenon that appears at the beginning of the apoptotic process. Annexin V has a high affinity for PS residues exposed on the cell surface, and is, therefore, routinely used to detect early apoptosis [34]. On the other hand, late-stage apoptotic cells, in addition to translocation of PS to the outer membrane, also show a loss of membrane integrity. The dye 7-AAD, with high affinity for DNA, is used to distinguish dead cells from early apoptotic cells [35]. Furthermore, apoptosis-induced cell death can be either caspase-dependent or caspase-independent. To determine whether the apoptosis observed in the annexin V assay was caused by the activation of caspases, the caspase 3/7 assay was performed. Executioner caspases such as caspase-3 and caspase-7 trigger the cleavage of many structural and regulatory proteins that arrest cellular functions, thus becoming hallmarks of apoptosis [36]. The caspase 3/7 assay uses a DNA-binding dye that binds to a DEVD peptide substrate. This dye is unable to bind DNA when bound to the DEVD peptide. The cleavage of this bond by caspase 3/7 in apoptotic cells results in the release of the dye, its translocation to the nucleus, and its binding to DNA. Likewise, this assay also included the dead cell marker 7-AAD.

**Figure 8:**
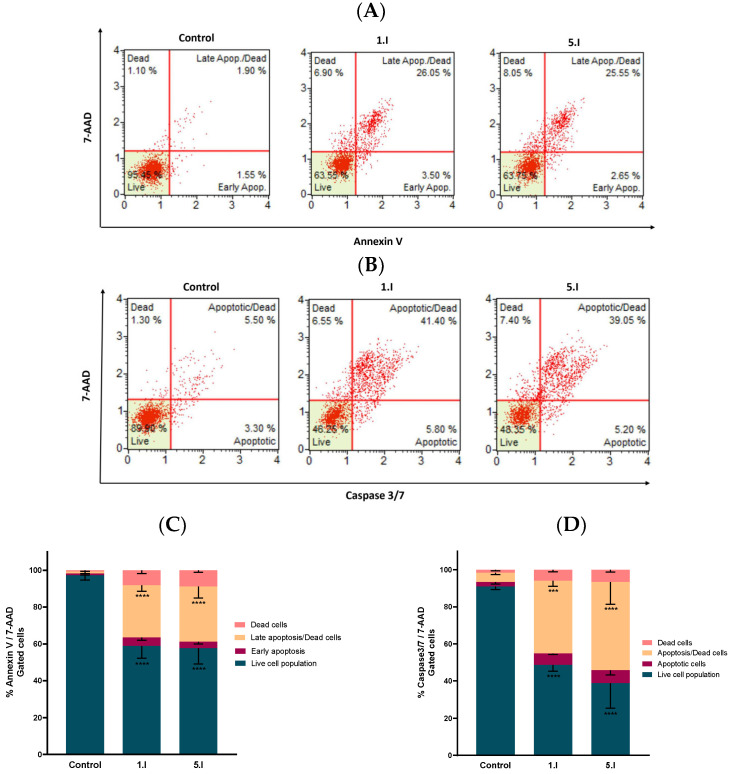
Compounds **1.I** and **5.I** induced apoptotic cell death in DU-145 cells. (**A**) Cells were treated with the concentration of 10 µM for 24 h and examined on a Muse™ automated cell analyzer with the Annexin V Live & Dead Cell apoptosis assay. (**B**) Analogous independent experiment performed with the Caspase 3/7 apoptosis assay. (**C**,**D**) Quantification of the cell population with the Annexin V & Dead Cell and Caspase 3/7 assays, respectively. Data are presented as the mean ± SEM of two independent experiments. **** *p* < 0.0001, *** *p* < 0.001 when comparing control and compounds.

Therefore, DU-145 cells were treated with compounds **1.I** and **5.I** for 24 h at the concentration of 10 µM, and the results were obtained following the manufacturer’s protocol. Four different cell populations were obtained in both assays: healthy cells (Annexin V, Caspase 3/7 and 7-AAD negative (lower left quadrant)); early apoptotic cells (both Annexin V and Caspase 3/7 positive and 7-AAD negative (lower right quadrant)); late apoptotic or dead cells (Annexin V, Caspase 3/7 and 7-AAD positive (upper right quadrant)); and necrotic cells (both Annexin V and Caspase 3/7 negative and 7-AAD positive (upper left quadrant)). Results obtained are shown in Figure 8, with a quantitative comparison of the difference in the cell population induced by each compound (Figure 8C,D). 

The cells treated with DMSO (control) were mainly located in the lower left quadrant, whereas treatment with compounds **1.I** or **5.I** induced a shift of healthy cells towards an apoptotic state. This shift was similar for both compounds at 10 µM in the Annexin V assay (Figure 8A), where viable population was reduced by 37%, while more than 40% of the cells were found to be apoptotic. The population of early apoptotic cells was about 3%, and the population of late/dead apoptotic cells increased with respect to the control by about 25% for both compounds (Figure 8A,C). Considering the Caspase 3/7 assay, both compounds exhibited similar behavior with a considerable increase in the population of apoptotic/dead cells (~40%) compared to the control (Figure 8B,D). Moreover, only a slight percentage of cells were necrotic, as there were almost no cells located in the upper left quadrant. 

Based on these results, it is demonstrated that both compounds, **1.I** and **5.I**, induce apoptosis in the prostate DU-145 human cancer cell line at 10 µM, with a clear involvement of the caspase 3/7.

#### 3.4.4. Compounds 1.I and 5.I Slightly Induce ROS Production during 24 h of Treatment

**Figure 9:**
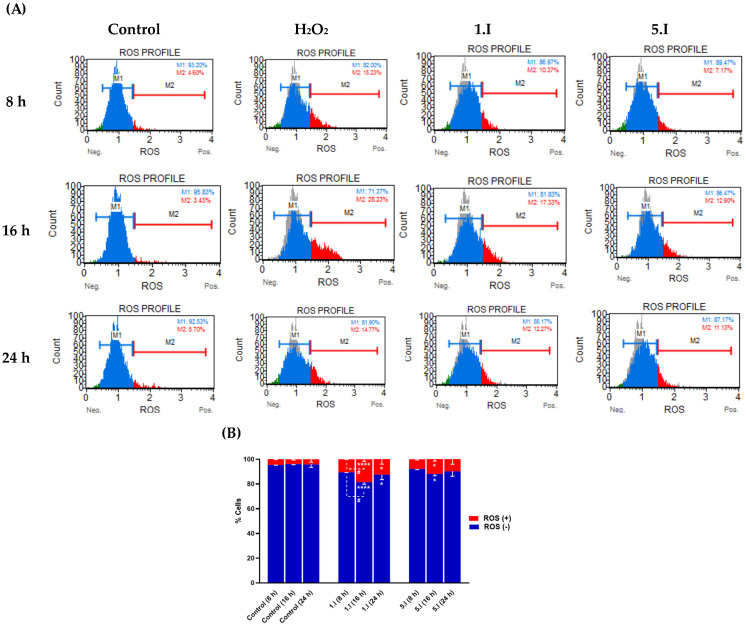
Effect of compound **1.I** and **5.I** on ROS levels. (**A**) DU-145 cells were treated with **1.I** and **5.I** at the concentration of 10 µM for 8, 16, and 24 h, and subjected to Muse flow cytometry-based oxidative stress analysis for total ROS levels measurement. (**B**) Quantification of the cell population with the oxidative stress assay. Data are presented as the mean ± SEM of two independent experiments and have been normalized. **** *p* < 0.0001, * *p* < 0.05 when comparing control and compounds, and ^#^ *p* < 0.05 when comparing compounds at different time points.

Acylselenourea derivatives have shown to exhibit good radical scavenging activity, as previously reported by our research group [11]. On the other hand, it has been described that the anticancer effect of certain Se-containing molecules is associated with increased total ROS levels [30]. Thus, to investigate whether the induced cytotoxic effect of **1.I** and **5.I** is related to ROS production, the total ROS level in DU-145 cells treated with **1.I** and **5.I** was performed. DU-145 cells were treated with DMSO, H_2_O_2_ (0.9 µM), **1.I** (10 µM), and **5.I** (10 µM) for 8, 16, and 24 h, and total ROS levels were measured. As shown in Figure 9, total ROS levels in cells treated with **5.I** did not change significantly between the three time points, reaching at 8 h about 7%, and at 24 h about 11% of ROS(+) cells. Likewise, cells treated with **1.I** exhibited a slight increase at 8 h in the ROS(+) cell population, reaching almost 20% compared to cells treated with **5.I**. However, at 24 h, the percentage of ROS(+) cells decreased to about 12%. Therefore, no increase in ROS levels was observed that could be associated with the cytotoxic effect of **1.I** and **5.I**, supporting their possible role as radical scavengers. Previously, we evaluated the protective effect against H_2_O_2_-induced cell damage in DU-145 cells treated with **1.I** and **5.I**. Both the compounds showed protective activity against H_2_O_2_-induced oxidative cell damage at the concentration of 0.0015 mg/mL (equals to 5.78 µM and 5.57 µM for **1.I** and **5.I**, respectively), reaching a greater two-fold increase of cell survival. Therefore, a significant increase in total ROS levels may not be observed in cells treated with **1.I** and **5.I** at 10 µM, as they may be exerting radical scavenging activity.

### 3.5. X-ray Crystallography of N-(allylcarbamoselenoyl)furan-2-carboxamide (Compound **1.II**)

**Figure 10:**
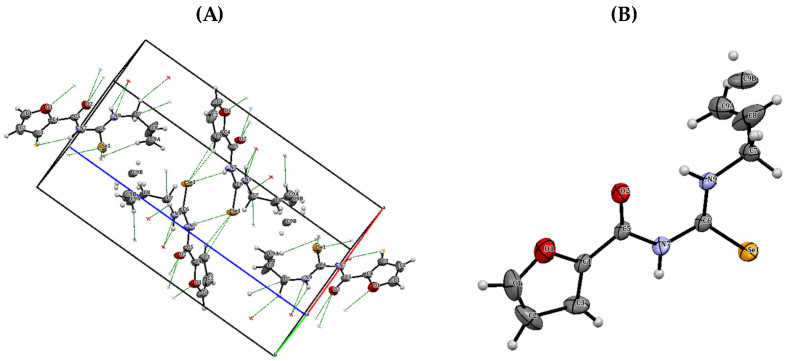
**(A)** Crystal packing of compound **1.II. (B)** ORTEP diagram of compound **1.II** with displacement ellipsoids drawn at 50% probability level.

The structure of the synthesized derivative was further confirmed by X-ray diffraction analysis of compound **1.II**, as an example, after growing a single crystal. The crystal data and structural refinements are listed in Table S6. The structure and crystal packing of the compound are shown in Figure 10, and the interatomic distances and angles selected for this structure are compiled in Table S7 of the Supplementary Material.

## 4. Discussion

The design of these new derivatives is the second approach to our previous work, in which we synthesized similar molecules where the Se atom was part of a selenoester group [18]. These molecules did not show a potent radical scavenging effect. Therefore, we decided to introduce the Se atom in the form of acylselenourea, since this functional group had shown a high free radical scavenging capacity [11]. The main aim of this structural modification was to develop dual molecules with anticancer and radical scavenging activity. Admittedly, the development of molecules with this dual activity may seem counterproductive since many chemotherapeutic drugs often damage DNA by producing free radicals that interfere with the cell cycle, leading to cell death by apoptosis or necrosis [37]. However, there are situations in which antioxidants can act synergistically with many of the anticancer drugs [38,39]. Quantitatively, these antioxidants will allow a longer and more prolonged duration of intake of antineoplastic agents, thus increasing the efficacy of therapy [40]. Moreover, it has been reported that conditions of elevated oxidative stress may slow down cell division, resulting in the ineffectiveness of many antineoplastic drugs, and could lead to the oxidation of cellular proteins into carbonyls capable of inhibiting caspases and consequently apoptosis [41]. On the other hand, molecules that exert both antioxidant and antiproliferative activity have been reported, such as 2,6-diaminopyridine derivatives [42], 1,3,5-trisubstituted aryl-5-hydroxypyrazoline analogues [43], 3,5-bis(arylidene)-4-piperidones [44], and several phosphoramidates containing selenium [45]. Most of them display moderate anticancer activity together with excellent antioxidant capacity [11,46,47].

The first approach to determine the radical scavenging activity of the novel acylselenoureas was the DPPH assay. They demonstrated dose-dependent DPPH activity showing a similar effect to the positive controls (ascorbic acid and trolox) at 0.03 mg/mL. It was also observed that the incorporation of the triple terminal bond (series **III**) led to a loss of radical scavenging activity. Likewise, the presence of a double bond between the hetero- or carbo-cycle and the carbonyl group also resulted in a loss of DPPH inhibitory activity. However, the introduction of propyl and allyl chains (series **I** and **II**, respectively) increased the radical scavenger activity. It is noteworthy that the selenoester analogs of this acylselenourea derivative did not appear to exert DPPH inhibitory activity in our previous work [18] (Table 2). Thus, the incorporation of the acylselenourea functional group has proven to be key for the design of free radical scavenger molecules.

On the other hand, we also evaluated the antiproliferative activity of the synthesized acylselenoureas against a panel of four cancer cell lines: HTB-54 (lung), MDA-MB-231 (breast), HT-29 (colon), and DU-145 (prostate). The latter cancer cell line was shown to be the most sensitive to the new compounds with eight of the 20 exhibiting less than 60% cell growth at 10 µM. In addition, the propyl derivatives (series **I**) showed higher cytotoxic activity. Among them, **1.I** and **5.I** also exhibited the highest SI in lung tissue, with values around 20-fold superior to cisplatin. These two compounds showed moderate-low IC_50_ values between 7.8 and 15.5 µM. Moreover, **1.I** and **5.I** demonstrated activity similar to ascorbic acid in the H_2_O_2_ -induced DU-145 cells oxidative damage model at 0.0015 mg/mL (equals to 5.78 µM and 5.57 µM for **1.I** and **5.I**, respectively). Hence, these new derivatives seem to be capable of exhibiting radical scavenging activity in cell culture. 

To further study the antiproliferative capacity of **1.I** and **5.I**, we evaluated whether they were able to induce apoptosis in the DU-145 cancer cell line at 10 µM. Both the compounds induced apoptosis with a clear involvement of caspase 3/7. Thus, their antiproliferative activity in DU-145 cells may be triggered by the caspase-mediated induction of apoptosis.

Finally, to determine whether apoptosis could be induced by increased total ROS levels, they were measured by flow cytometry at different time points after exposure to 10 µM of **1.I** and **5.I** in DU-145 cells. Both the compounds showed a slight increase in total ROS levels that peaked at 16 h, with levels around 17% and 13%. However, they did not reach sufficient levels to likely be responsible for the induction of apoptosis.

## 5. Conclusions

In this work, we synthesized 20 novel acylselenoureas that combine in their structure hetero- and carbo-cycles with three-carbon aliphatic chains bearing a double- (allylic), triple- (propargylic), or single-terminal (propyl) bond. We demonstrated how the introduction of the acylselenourea functional group should render new radical scavenger derivatives. In addition, **1.I** and **5.I** stood out for their potent DPPH inhibitory activity and their protective effect against free radicals in cell culture. Moreover, they have shown high-moderate antiproliferative activity, together with high SI and apoptosis induction. Therefore, we have developed two molecules with dual activity as anticancer agents and radical scavengers.

## Figures and Tables

**Table 1:** IC_50_ values (in µM) and SI for compounds, **1.I**, **2.I**, **5.I**, **7.II, 10.II**, **2.III**, **8.III, and cisplatin** in MDA-MB-231, DU-145, HTB-54, and BEAS-2B cell lines.

	Cell Lines				
Compounds	Breast	Prostate	Lung	Lung	
	MDA-MB-231	DU-145	HTB-54	BEAS-2B	SI ^a^
1.I	8.8 ± 4.8	9.5 ± 2.5	7.8 ± 12.7	>100	>12.7
2.I	n.d ^b^	n.d ^b^	8.6 ± 1.1	5.3 ± 0.6	0.6
5.I	15.5 ± 6.3	11.5 ± 3.4	8.4 ± 3.9	>100	>11.9
7.II	n.d ^b^	n.d ^b^	12.0 ± 2.7	6.3 ± 1.5	0.5
10.II	n.d ^b^	n.d ^b^	43. 0 ± 4.1	29.9 ± 5.0	0.7
2.III	n.d ^b^	n.d ^b^	11.6 ± 1.6	26.6 ± 4.0	2.3
8.III	n.d ^b^	n.d ^b^	21.7 ± 4.1	>100	>4.6
Cisplatin	5.5 ± 0.5	6.2 ± 0.1	13.68 [32]	8.63 [33]	0.63

IC_50_ values are presented as the mean ± SD of at least three independent experiments determined by the MTT assay. ^a^ SI calculates in lung cells as IC_50_ (BEAS-2B)/IC_50_ (HTB-54). ^b^ Not determined.

**Table 2:** DPPH inhibitory activity at 0.03 mg/mL and at 90 min for selenoester and acylselenourea analogs.

	DPPH Inhibitory%			
R_1_	Allyl Chain		Propargyl Chain	
	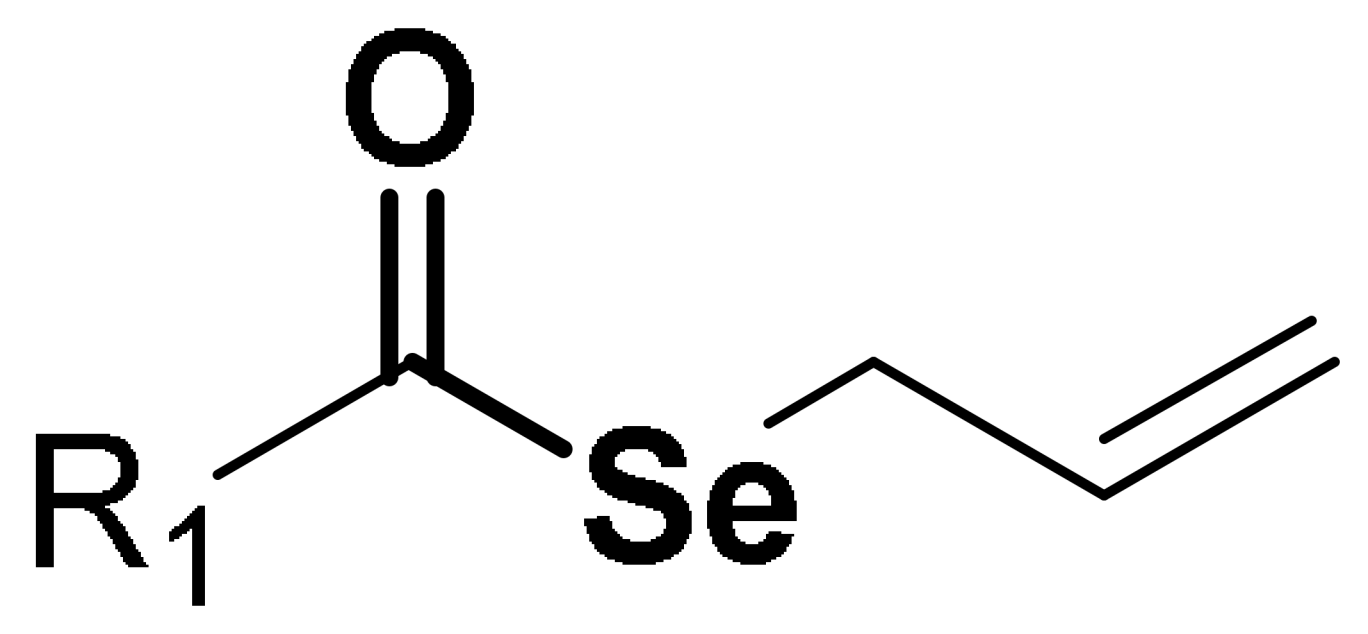	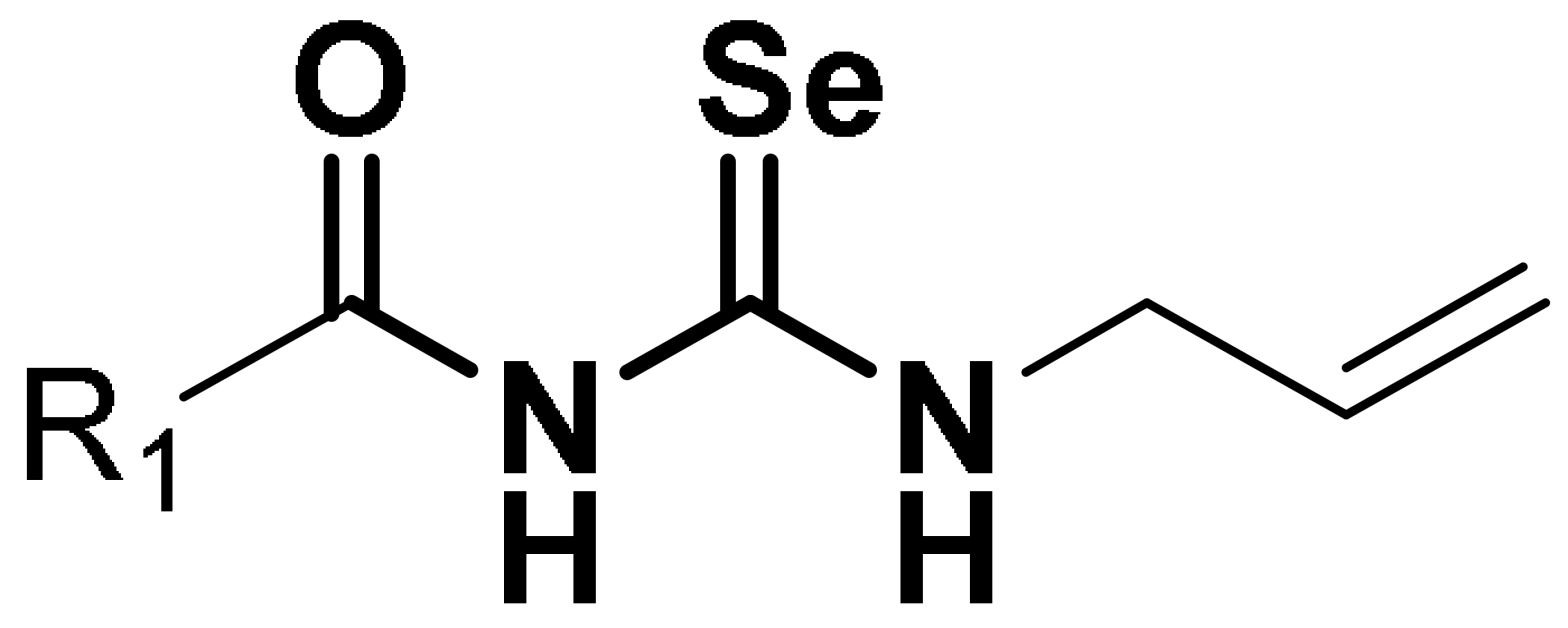	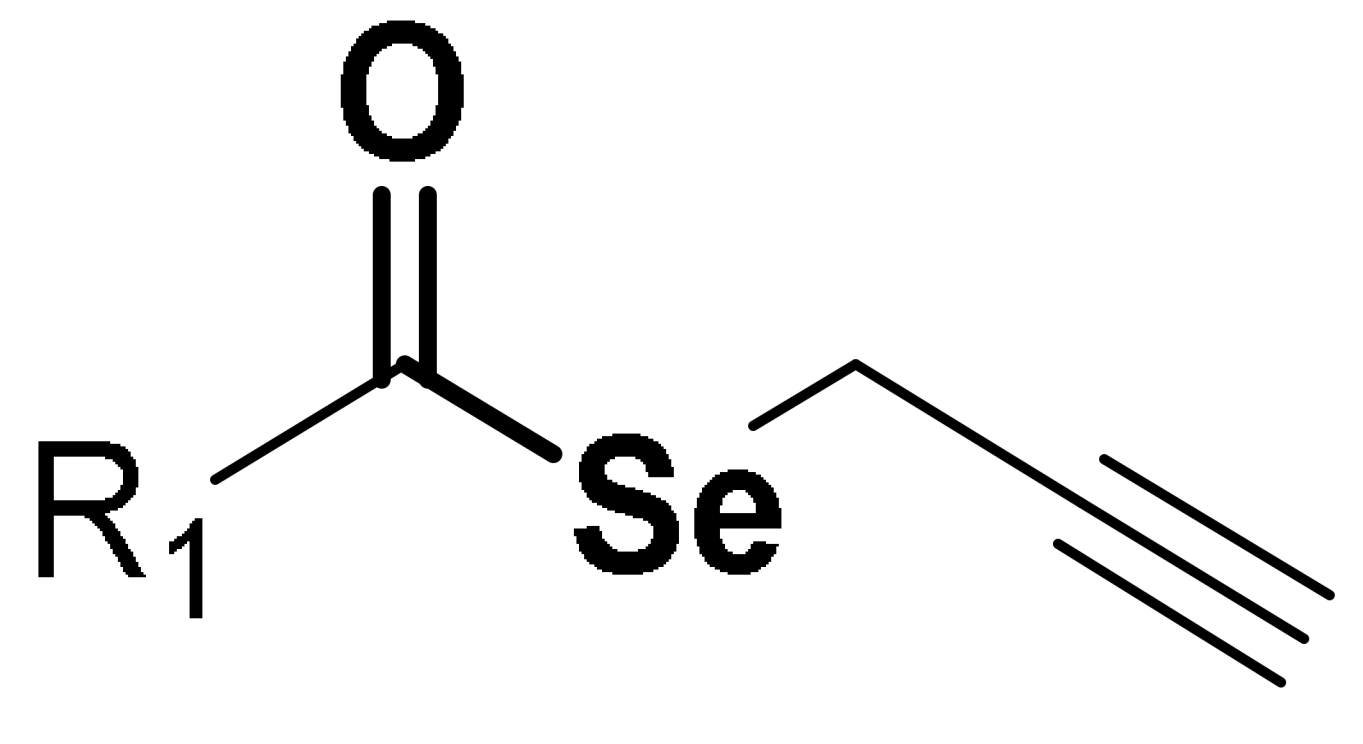	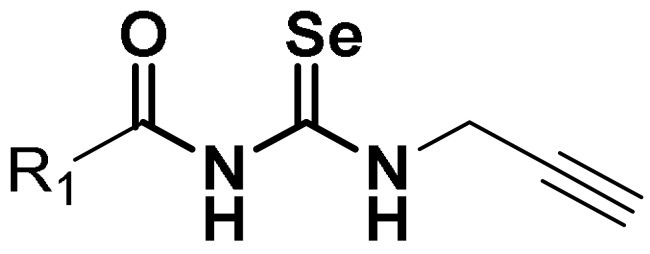
	*Selenoester* ^a^	*Acylselenourea*	*Selenoester* ^a^	*Acylselenourea*
phenyl	9.0 ± 1.2	97.4 ± 0.8	3.5 ± 0.9	54.6 ± 0.8
5-benzo[d][1,3]dioxyl	0.0 ± 2.3	88.2 ± 1.3	4.2 ± 8.0	62.4 ± 3.1
2-phenylvinyl	3.6 ± 5.3	26.7 ± 0.6	2.4 ± 2.0	
3-pyridyl	6.3 ± 2.9	96.2 ± 0.2	7.4 ± 0.7	
2-thiophenyl	7.8 ± 10.4	97.3 ± 0.8	0.0 ± 2.1	33.0 ± 3.1
2-furyl	4.6 ± 7.5	96.6 ± 0.6	5.4 ± 2.3	42.6 ± 0.6

^a^ Data obtained from [18].

## Data Availability

The data presented in this study are available in the article and Supplementary Materials.

## Supplementary Materials

The following supporting information can be downloaded at: https://www.mdpi.com/article/10.3390/antiox12071331/s1, Figures S1–S99: Spectroscopic characterization; Figure S100: DPPH radical scavenging activity at 0.003 mg/mL; Table S1: Cytotoxicity screening; Tables S2 and S3: DPPH radical scavenging assay; Tables S4 and S5: H_2_O_2_-induced oxidative damage in DU-145; Tables S6 and S7: X-ray crystallography data.

## Abbreviations

ATCC	American Type Culture Collection
DMSO	Dimethylsulfoxide
MTT	3-(4,5-dimethyl-2-thiazolyl)-2,5-diphenyl-2*H*-tetrazolium bromide
*N,N*-DMF	*N,N*-dimethylformamide
NMR	Nuclear Magnetic Resonance
NP	Natural Products
PS	Phosphotidylserine
RDA	Recommended Dietary Allowances
ROS	Reactive Oxygen Species
Se	Selenium
SI	Selectivity Index
TLC	thin-layer chromatography
