# Supplementary material for: Novel Acylselenourea Derivatives: Dual Molecules with Anticancer and Radical Scavenging Activity
Source: Antioxidants (Basel). 2023 Jun 23;12(7):1331. doi: 10.3390/antiox12071331 (PMC10376326; doi:10.3390/antiox12071331)
Supplement: Supplementary file 1 [file antioxidants-12-01331-s001.zip › antioxidants-2455574-supplementary.pdf]

Novel Acylselenourea Derivatives: Dual Molecules with Anticancer and Radical Scavenging Activity

Nora Astrain-Redin [1,2], Asif Raza [2], Ignacio Encío [3,4], Arun K. Sharma [2], Daniel Plano [1,3,*] and Carmen Sanmartín [1,3,*]

1 Departamento de Tecnología y Química Farmacéuticas, Universidad de Navarra, Irunlarrea 1, E-31008 Pamplona, Spain; nastrain@alumni.unav.es (N.A.-R.); dplano@unav.es (D.P.)

2 Department of Pharmacology, Penn State Cancer Institute, CH72, Penn State College of Medicine, 500 University Drive, Hershey, PA 17033, USA; asharma1@pennstatehealth.psu.edu (A.K.S.); mraza@pennstatehealth.psu.edu (A.R.)

3 Instituto de Investigación Sanitaria de Navarra (IdiSNA), Irunlarrea, 3, 31008, Pamplona, Spain; ignacio.encio@unavarra.es

4 Departamento de Ciencias de la Salud, Universidad Pública de Navarra, Avda. Barañain s/n, 31008, Pamplona, Spain

* Correspondence: dplano@unav.es (D.P.); sanmartin@unav.es (C.S.)

Table of Contents

**Biological evaluation**	
**Cytotoxicity screening**	**Table S1**
**DPPH radical scavenging assay**	**Tables S2-S3**<br>**Figure S100**
**H_2O_2-induced oxidative damage in DU-145 cells**	**Tables S4-S5**
**X-ray crystallography data**	**Tables S6-S7**
**Spectroscopic characterization**	
**^{1}H, ^{13}C and ^{77}Se NMR spectra**	**Figures S1-S99**

Table S1. Cell growth % for the compounds **1.I-6.I**, **1.II-11.II**, and **1.III-8.III** in HTB-54, DU-145, HT-29, and MDA-MB-231.

	10 µM				50 µM			
	Cancer cell lines				Cancer cell lines			
	HTB-54	MDA-MB-231	DU-145	HT-29	HTB-54	MDA-MB-231	DU-145	HT-29
**1.I**	47.09 ± 14.8	46.68 ± 5.6	44.88 ± 4.8	44.17 ± 7.1	31.65 ± 8.2	37.96 ± 7.8	48.73 ± 3.1	25.53 ± 4.2
**2.I**	92.29 ± 6.2	32.83 ± 7.2	54.53 ± 8.4	48.43 ± 13.8	71.81 ± 4.4	15.23 ± 8.8	50.20 ± 2.0	32.69 ± 6.9
**5.I**	36.79 ± 8.6	44.99 ± 12.5	42.43 ± 4.9	39.59 ± 14.3	27.54 ± 6.5	21.29 ± 11.2	47.87 ± 3.9	21.39 ± 1.5
**6.I**	84.63 ± 5.9	88.36 ± 16.3	19.47 ± 2.0	60.43 ± 9.7	36.21 ± 5.7	23.48 ± 12.9	12.57 ± 1.0	25.13 ± 5.0
**1.II**	54.85 ± 11.2	80.43 ± 8.4	81.50 ± 13.5	94.99 ± 15.7	11.10 ± 5.2	14.52 ± 7.9	10.56 ± 5.7	16.65 ± 5.3
**2.II**	35.85 ± 12.1	79.94 ± 9.6	63.36 ± 5.6	107.24 ± 16.2	8.52 ± 4.8	11.07 ± 5.9	7.33 ± 4.6	26.20 ± 5.1
**3.II**	73.12 ± 3.0	79.59 ± 7.7	93.62 ± 14.7	63.26 ± 15.0	35.35 ± 8.9	14.06 ± 5.8	10.43 ± 4.1	33.21 ± 6.1
**4.II**	79.19 ± 9.2	81.12 ± 6.7	67.23 ± 7.1	100.1 ± 7.1	44.16 ± 6.2	17.63 ± 6.6	13.79 ± 1.2	32.90 ± 5.0
**5.II**	67.61 ± 14.9	80.43 ± 8.4	97.48 ± 18.5	100.18 ± 16.6	16.85 ± 4.1	15.62 ± 6.0	10.34 ± 4.9	23.85 ± 5.0
**6.II**	68.67 ± 8.2	51.81 ± 6.8	57.30 ± 3.6	94.72 ± 5.3	44.07 ± 10.6	30.48 ± 7.1	14.56 ± 4.5	56.07 ± 11.0
**7.II**	21.44 ± 8.2	33.91 ± 6.7	50.65 ± 2.4	110.11 ± 19.4	10.17 ± 5.8	10.52 ± 7.0	8.95 ± 4.9	19.04 ± 3.6
**8.II**	51.86 ± 15.0	80.71 ± 6.4	88.61 ± 10.1	83.51 ± 16.4	20.03 ± 6.6	15.52 ± 7.6	10.28 ± 4.2	26.64 ± 3.8
**9.II**	82.80 ± 2.8	83.75 ± 7.9	110.12 ± 9.0	112.94 ± 7.8	60.04 ± 4.5	71.33 ± 5.3	69.57 ± 6.1	102.91 ± 7.1
**10.II**	52.59 ± 10.5	24.81 ± 6.1	18.15 ± 2.7	47.75 ± 8.7	43.50 ± 17.6	21.00 ± 9.4	18.79 ± 3.1	46.40 ± 4.8
**11.II**	48.26 ± 6.9	77.12 ± 10.1	93.85 ± 8.0	88.11 ± 4.8	5.94 ± 4.7	14.69 ± 7.8	22.01 ± 5.8	30.82 ± 4.0
**1.III**	76.00 ± 4.8	66.33 ± 16.7	42.41 ± 4.8	83.98 ± 16.8	48.02 ± 4.2	17.56 ± 9.3	15.17 ± 1.2	17.23 ± 4.2
**2.III**	76.75 ± 6.8	27.43 ± 5.4	12.75 ± 2.8	43.42 ± 4.2	44.74 ± 3.5	9.48 ± 7.8	14.30 ± 6.7	20.61 ± 4.0
**3.III**	74.23 ± 4.0	66.84 ± 7.2	86.45 ± 9.3	94.82 ± 17. 6	35.29 ± 5.7	20.11 ± 8.0	24.70 ± 4.9	51.37 ± 6.0
**5.III**	76.00 ± 4.8	78.45 ± 12.0	80.81 ± 7.4	70.03 ± 8.7	33.41 ± 8.6	33.28 ± 6.5	26.27 ± 6.5	33.88 ± 4.5
**8.III**	34.54 ± 9.1	61.89 ± 13.5	63.05 ± 4.8	40.26 ± 8.3	31.63 ± 6.1	19.15 ± 9.6	12.27 ± 7.8	18.08 ± 5.3

The cell growth % is presented as the mean ± SD of three independent experiments performed in triplicates

Table S2. DPPH radical scavenging activity (%) for the compounds **1.I-6.I**, **1.II-11.II**, and **1.III-8.III** at 0.03 mg/mL in the time range of 0, 5, 15, 30, 60, 90, and 120 minutes.

**0.03 mg/mL**	**DPPH scavenging activity (%)**						
**Compd.**	0 min	5 min	15 min	30 min	60 min	90 min	120 min
**Asc. Acid**	96.4 ± 0.2	96.4 ± 0.1	96.9 ± 0.5	96.9 ± 0.5	96.6 ± 0.1	96.6 ± 0.1	96.6 ± 0.1
**Trolox**	90.8 ± 2.3	96.3 ± 0.1	96.3 ± 0.2	96.3 ± 0.2	96.2 ± 0.2	96.2 ± 0.2	96.2 ± 0.2
**1.I**	1.8 ± 1.5	10.3 ± 4.3	29.7 ± 4.1	55.4 ± 5.1	95.4 ± 4.1	96.5 ± 0.7	96.4 ± 0.6
**2.I**	2.5 ± 0.6	12.8 ± 1.8	36.2 ± 0.1	59.0 ± 3.0	90.7 ± 5.7	95.5 ± 0.2	95.5 ± 0.3
**5.I**	3.2 ± 2.4	16.6 ± 3.1	29.8 ± 3.4	46.1 ± 3.9	72.6 ± 6.2	97.7 ± 0.6	96.7 ± 2.5
**6.I**	2.3 ± 1.0	10.1 ± 1.1	15.4 ± 1.3	21.8 ± 1.1	35.1 ± 1.2	50.2 ± 0.8	66.4 ± 0.2
**1.II**	16.0 ± 1.9	45.2 ± 5.1	65.4 ± 4.1	85.4 ± 4.9	96.5 ± 0.6	96.6 ± 0.6	96.5 ± 0.5
**2.II**	24.4 ± 3.0	60.7 ± 2.5	75.8 ± 3.5	87.6 ± 4.0	96.7 ± 0.7	97.3 ± 0.8	97.3 ± 0.8
**3.II**	0.7 ± 2.7	5.2 ± 1.8	9.0 ± 1.6	14.3 ± 1.1	23.7 ± 0.8	31.7 ± 0.1	39.6 ± 0.1
**4.II**	0.9 ± 2.8	6.1 ± 1.7	9.9 ± 1.5	15.5 ± 1.4	25.6 ± 1.3	35.0 ± 0.6	44.3 ± 0.6
**5.II**	13.5 ± 1.3	42.4 ± 1.5	62.7 ± 0.8	80.2 ± 1.1	97.2 ± 0.7	97.4 ± 0.8	97.7 ± 1.0
**6.II**	0.0 ± 1.7	1.0 ± 1.7	4.6 ± 1.3	9.8 ± 1.1	18.8 ± 0.9	26.7 ± 0.6	34.2 ± 0.7
**7.II**	56.3 ± 6.2	90.0 ± 1.3	95.7 ± 0.4	96.2 ± 0.2	96.2 ± 0.2	96.2 ± 0.2	96.2 ± 0.2
**8.II**	17.6 ± 4.6	39.0 ± 3.0	55.5 ± 2.1	68.0 ± 1.7	81.4 ± 1.3	88.2 ± 1.3	93.7 ± 1.4
**9.II**	1.9 ± 1.9	6.1 ± 1.7	10.1 ± 1.2	14.7 ± 1.1	22.3 ± 1.2	29.0 ± 1.2	35.2 ± 1.3
**10.II**	31.6 ± 4.1	69.1 ± 0.1	86.3 ± 0.9	94.4 ± 0.7	96.1 ± 0.1	96.0 ± 0.2	96.0 ± 0.3
**11.II**	11.0 ± 3.0	38.5 ± 0.3	56.8 ± 0.6	70.1 ± 1.0	83.0 ± 1.0	89.6 ± 1.2	94.3 ± 1.2
**1.III**	11.8 ± 1.7	15.2 ± 4.4	23.5 ± 3.9	31.2 ± 3.7	40.9 ± 4.3	42.6 ± 0.6	48.1 ± 0.4
**2.III**	14.5 ± 2.3	12.8 ± 3.0	19.8 ± 2.8	24.8 ± 2.6	30.2 ± 2.9	33.0 ± 3.1	34.4 ± 2.9
**3.III**	2.6 ± 1.3	6.3 ± 1.1	10.9 ± 0.9	17.5 ± 0.4	28.4 ± 0.4	37.1 ± 0.3	45.9 ± 0.6
**5.III**	9.3 ± 1.0	9.8 ± 0.3	23.0 ± 0.3	34.0 ± 0.6	46.6 ± 0.9	54.6 ± 0.8	60.2 ± 0.7
**8.III**	6.4 ± 2.5	17.9 ± 0.9	32.8 ± 0.9	45.2 ± 2.0	59.7 ± 3.8	62.4 ± 3.1	72.5 ± 8.1

The percentage of inhibition of DPPH is presented as the mean ± SEM of three independent experiments performed in triplicates.

Table S3. DPPH radical scavenging activity (%) for the compounds **1.I-6.I**, **1.II-11.II**, and **1.III-8.III** at 0.003 mg/mL in the time range of 0, 5, 15, 30, 60, 90, and 120 minutes.

**0.003 mg/mL**	**DPPH scavenging activity (%)**						
**Compd.**	0 min	5 min	15 min	30 min	60 min	90 min	120 min
**Asc. Acid**	33.5 ± 0.5	33.6 ± 0.4	34.5 ± 1.6	34.1 ± 1.1	34.0 ± 0.9	34.0 ± 0.8	34.0 ± 0.7
**Trolox**	28.4 ± 1.0	29.2 ± 1.0	29.2 ± 0.9	29.2 ± 0.8	29.2 ± 0.8	29.2	29.1
**1.I**	0.0 ± 1.7	1.1 ± 2.0	3.5 ± 2.0	6.6 ± 2.0	11.7 ± 2.3	15.5 ± 2.5	17.5 ± 2.3
**2.I**	0.0 ± 1.5	4.9 ± 3.6	8.2 ± 3.3	11.4 ± 2.7	16.0 ± 3.4	17.0 ± 2.2	17.8 ± 2.2
**5.I**	1.7 ± 1.1	2.9 ± 1.1	4.6 ± 1.2	6.4 ± 1.3	9.3 ± 1.4	11.4 ± 1.5	13.0 ± 1.6
**6.I**	1.5 ± 0.5	2.0 ± 0.8	2.5 ± 0.8	3.1 ± 0.6	4.4 ± 0.6	5.6 ± 0.7	6.6 ± 0.8
**1.II**	1.7 ± 0.3	5.4 ± 0.5	8.8 ± 0.9	12.1 ± 1.2	16.5 ± 1.5	19.5 ± 1.7	21.1 ± 1.4
**2.II**	0.0 ± 1.0	6.4 ± 1.1	10.4 ± 1.2	13.0 ± 1.2	15.2 ± 1.2	16.0 ± 1.2	16.3 ± 1.2
**3.II**	0.0 ± 3.3	0.0 ± 3.2	0.0 ± 3.1	0.5 ± 2.9	1.7 ± 2.9	2.5 ± 2.9	3.3 ± 2.9
**4.II**	0.0 ± 2.9	0.0 ± 2.7	0.0 ± 2.6	0.5 ± 2.5	1.7 ± 2.4	2.5 ± 2.4	3.1 ± 2.3
**5.II**	1.3 ± 1.7	4.5 ± 1.7	7.5 ± 1.7	10.4 ± 1.8	13.9 ± 1.9	16.2 ± 2.0	17.8 ± 2.1
**6.II**	0.0 ± 2.9	0.0 ± 2.7	0.0 ± 2.6	0.0 ± 2.6	0.0 ± 2.4	0.5 ± 2.3	1.1 ± 2.3
**7.II**	3.6 ± 2.3	12.0 ± 2.2	16.8 ± 1.3	18.9 ± 1.3	20.3 ± 1.2	20.7 ± 1.3	20.7 ± 1.3
**8.II**	1.2 ± 1.4	2.8 ± 1.3	5.0 ± 1.2	7.1 ± 1.1	9.7 ± 1.1	10.9 ± 1.2	11.7 ± 1.2
**9.II**	0.1 ± 2.9	0.3 ± 2.7	0.8 ± 2.5	1.5 ± 2.4	2.3 ± 2.3	3.0 ± 2.3	3.5 ± 2.3
**10.II**	0.9 ± 1.8	6.7 ± 1.7	11.5 ± 1.5	14.7 ± 1.2	17.7 ± 1.1	19.0 ± 0.9	19.7 ± 0.9
**11.II**	0.0 ± 1.7	2.5 ± 1.4	5.8 ± 1.2	8.6 ± 0.9	11.6 ± 1.0	12.8 ± 0.8	13.5 ± 0.7
**1.III**	1.5 ± 0.7	3.4 ± 0.6	4.9 ± 0.7	6.5 ± 0.7	8.5 ± 1.0	9.6 ± 0.9	10.0 ± 0.8
**2.III**	2.4 ± 0.6	4.3 ± 0.4	5.4 ± 0.3	6.5 ± 0.5	7.1 ± 0.5	7.3 ± 0.6	7.3 ± 0.6
**3.III**	0.9 ± 1.9	1.2 ± 1.8	1.9 ± 1.7	2.7 ± 1.7	4.1 ± 1.7	5.3 ± 1.7	6.2 ± 1.6
**5.III**	0.6 ± 1.2	2.9 ± 1.0	5.1 ± 1.3	7.2 ± 1.6	9.8 ± 1.9	11.3 ± 2.0	12.3 ± 2.1
**8.III**	1.1 ± 2.3	2.4 ± 2.2	4.4 ± 2.0	6.2 ± 1.9	8.3 ± 1.8	9.2 ± 1.8	9.7 ± 1.8

The percentage of inhibition of DPPH is presented as the mean ± SEM of three independent experiments performed in triplicates.

Table S4. Antioxidant activity of hit compounds at 0.0015 mg/mL was evaluated using the H_2O_2-induced DU-145 cells oxidative damage model at different concentrations of H_2O_2.

	**200 µM H_2O_2**		**250 µM H_2O_2**		**300 µM H_2O_2**	
**Compound**	**Cell growth inhibition (%)**	**Increase-fold**	**Cell growth inhibition (%)**	**Increase-fold**	**Cell growth inhibition (%)**	**Increase-fold**
**H_2O_2**	36.9 ± 6.9		53.4 ± 6.1		66.8 ± 6.3	
**1.I**	14.7 ± 2.3	2.3 ± 0.7	17.8 ± 2.1	2.7 ± 0.4	24.6 ± 3.0	2.3 ± 0.3
**2.I**	6.8 ± 4.9	2.9 ± 1.5	13.5 ± 0.7	3.1 ± 1.3	18.4 ± 7.5	2.9 ± 1.2
**5.I**	10.6 ± 6.1	3.1 ± 0.5	18.6 ± 1.4	2.7 ± 0.3	24.4 ± 4.7	2.4 ± 0.4
**7.II**	24.9 ± 3.4	1.5 ± 0.2	43.5 ± 6.8	1.2 ± 0.3	46.4 ± 7.4	1.4 ± 0.3
**10.II**	31.4 ± 8.9	1.0 ± 0.6	48.3 ± 9.8	1.1 ± 0.2	58.5 ± 8.6	1.1 ± 0.2
**Asc. Acid**	9.1 ± 0.8	3.6 ± 0.3	16.6 ± 3.1	2.9 ± 0.7	37.0 ± 4.1	1.5 ± 0.2

The percentage of cell growth inhibition is presented as the mean ± SEM of three independent experiments performed in triplicates.

Table S5. Antioxidant activity of hit compounds at 0.0003 mg/mL evaluated using the H_2O_2-induced DU-145 cells oxidative damage model at different concentrations of H_2O_2.

	**200 µM H_2O_2**		**250 µM H_2O_2**		**300 µM H_2O_2**	
**Compound**	**Cell growth inhibition (%)**	**Increase-fold**	**Cell growth inhibition (%)**	**Increase-fold**	**Cell growth inhibition (%)**	**Increase-fold**
**H_2O_2**	36.9 ± 6.9		53.4 ± 6.1		66.8 ± 6.3	
**1.I**	16.3 ± 2.7	2.0 ± 0.0	26.3 ± 8.1	2.0 ± 0.7	32.1 ± 7.7	1.8 ± 0.4
**2.1**	25.3 ± 4.1	0.8 ± 0.2	52.5 ± 8.4	0.9 ± 0.2	37.5 ± 3.7	1.0 ± 0.3
**5.I**	17.97 ± 5.4	1.9 ± 0.3	24.8 ± 4.3	1.9 ± 0.4	33.9 ± 8.5	1.7 ± 0.4
**7.II**	21.0 ± 6.6	2.3 ± 1.2	33.8 ± 3.6	1.6 ± 0.2	37.3 ± 9.3	1.8 ± 0.4
**10.II**	45.6 ± 4.1	0.8 ± 0.4	51.3 ± 7.5	1.0 ± 0.3	73.1 ± 9.6	1.0 ± 0.2
**Asc. Acid**	7.9 ± 1.9	4.2 ± 0.4	15.5 ± 2.8	3.1 ± 0.7	26.8 ± 1.1	2.2 ± 0.1

The percentage of cell growth inhibition is presented as the mean ± SEM of three independent experiments performed in triplicates.

Table S6. X-ray Crystallographic Data and Refinement Parameters for compound **1.II**.

Crystal data

$C_9H_{10}N_2O_2Se$	?
M_r = 254.13	D_x = 1.680 Mg m^{-3}
Monoclinic, $P2_1/n$	Melting point: ? K
Hall symbol: ?	Cu *K*α radiation, λ = 1.54184 Å
a = 11.2263 (1) Å	Cell parameters from 5122 reflections
b = 4.8613 (1) Å	θ = 2.4–73.9°
c = 18.4256 (2) Å	μ = 4.88 mm^{-1}
β = 92.093 (1)°	*T* = 173 K
V = 1004.90 (3) Å^3	Block, colourless
Z = 4	0.18 × 0.15 × 0.15 mm
F(000) = 500	

Data collection

ROD, Synergy Custom system, HyPix-Arc 150 diffractometer	1901 independent reflections
Radiation source: Rotating-anode X-ray tube, Rigaku (Cu) X-ray Source	1869 reflections with $I > 2\sigma(I)$
Mirror monochromator	R_{int} = 0.032
Detector resolution: 10.0000 pixels mm^{-1}	θ_{max} = 74.2°, θ_{min} = 4.5°
ω scans	*h* = −13→12
Absorption correction: multi-scan *CrysAlis PRO* 1.171.41.109a (Rigaku Oxford Diffraction, 2021) Empirical absorption correction using spherical harmonics, implemented in SCALE3 ABSPACK scaling algorithm.	*k* = −5→6
T_{min} = 0.260, T_{max} = 1.000	*l* = −21→22
4970 measured reflections	

Refinement

Refinement on F^2	Secondary atom site location: ?
Least-squares matrix: full	Hydrogen site location: inferred from neighbouring sites
$R[F^2 > 2\sigma(F^2)]$ = 0.047	H-atom parameters constrained
$wR(F^2)$ = 0.124	$w = 1/[\sigma^2(F_o^2) + (0.0712P)^2 + 2.384P]$ where $P = (F_o^2 + 2F_c^2)/3$
S = 1.04	$(\Delta/\sigma)_{max}$ = 0.001
1901 reflections	$\Delta\rho_{max}$ = 0.96 e Å^{-3}
138 parameters	$\Delta\rho_{min}$ = −0.63 e Å^{-3}
0 restraints	Extinction correction: *SHELXL2018*/3 (Sheldrick 2018), $Fc^*=kFc[1+0.001xFc^2\lambda^3/\sin(2\theta)]^{-1/4}$
? constraints	Extinction coefficient: 0.0174 (12)
Primary atom site location: dual	

Table S7. Selected Interatomic Distances (Å) and Angles (°) for compound **1.II**

Geometric parameters (Å, °)

Se1—C6	1.832 (3)	C1—C2	1.284 (8)
O1—C1	1.350 (6)	C2—C3	1.469 (7)
O1—C4	1.353 (4)	C3—C4	1.339 (5)
O2—C5	1.215 (4)	C4—C5	1.466 (5)
N7—C5	1.380 (4)	C7—C8	1.481 (6)
N7—C6	1.390 (5)	C8—C9A	1.138 (11)
N9—C6	1.312 (4)	C8—C9B	1.160 (15)
N9—C7	1.457 (5)		
C1—O1—C4	107.0 (4)	O2—C5—N7	123.4 (3)
C5—N7—C6	127.1 (3)	O2—C5—C4	121.7 (3)
C6—N9—C7	124.0 (3)	N7—C5—C4	114.8 (3)
C2—C1—O1	110.4 (4)	N7—C6—Se1	117.2 (2)
C1—C2—C3	108.4 (4)	N9—C6—Se1	125.0 (3)
C4—C3—C2	102.8 (4)	N9—C6—N7	117.9 (3)
O1—C4—C5	117.1 (3)	N9—C7—C8	114.8 (5)
C3—C4—O1	111.3 (3)	C9A—C8—C7	133.0 (8)
C3—C4—C5	131.6 (3)	C9B—C8—C7	154.1 (9)

Spectroscopic characterization-NMR spectra

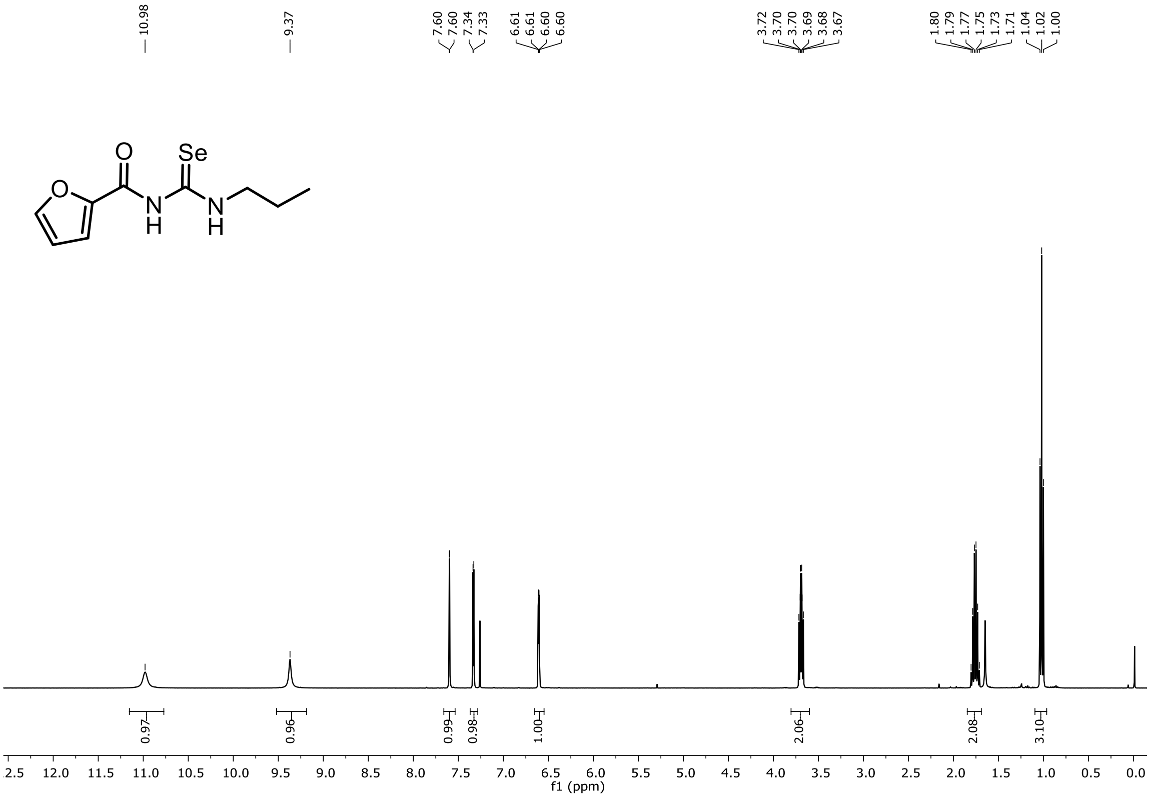

Figure S1. ^{1}H-NMR spectrum of compound **1.I**.

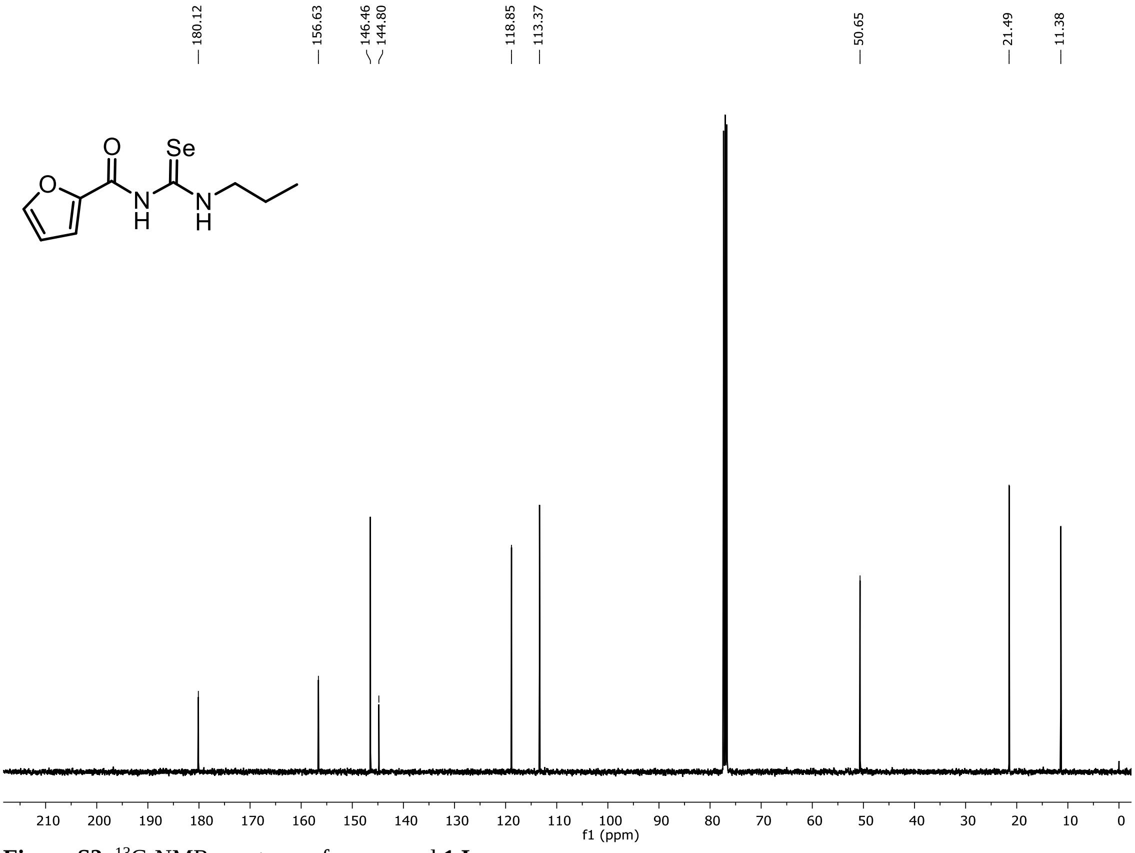

Figure S2. ^{13}C-NMR spectrum of compound **1.I**.

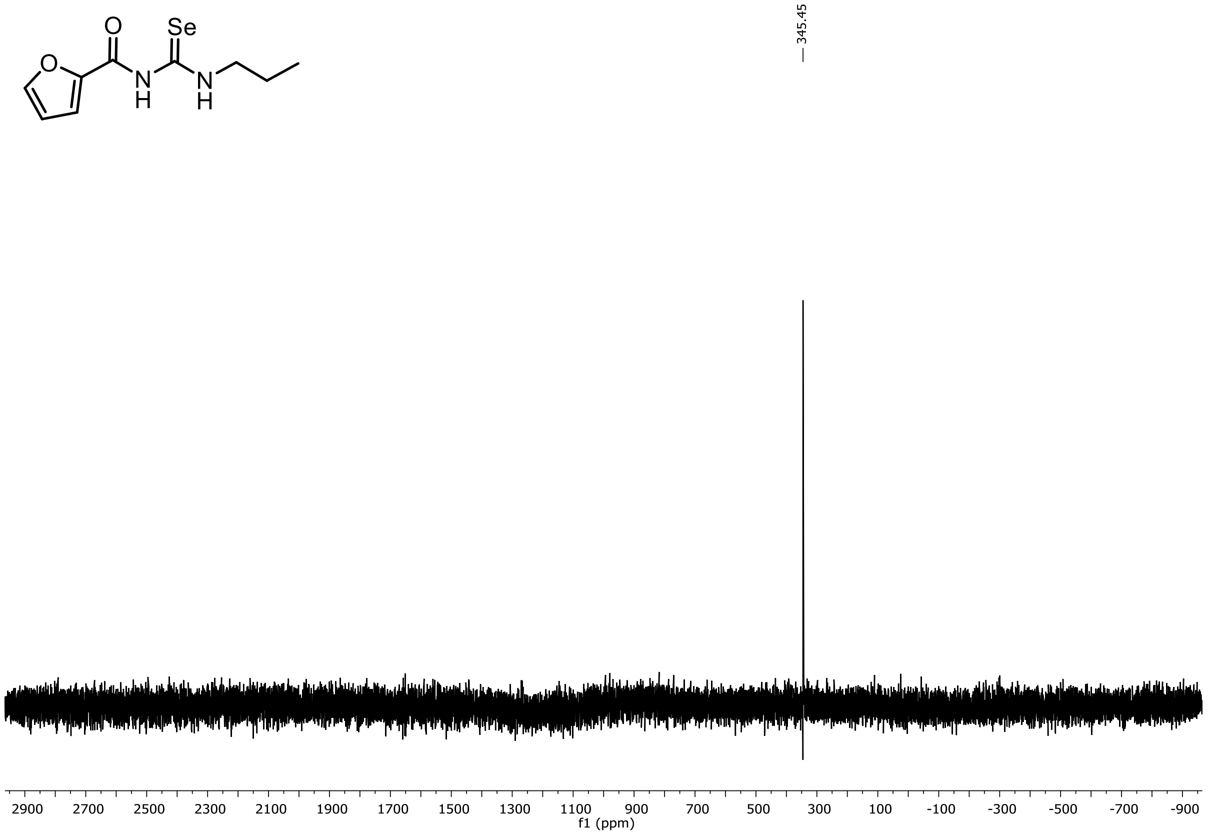

Figure S3. ^{77}Se-NMR spectrum of compound **1.I**.

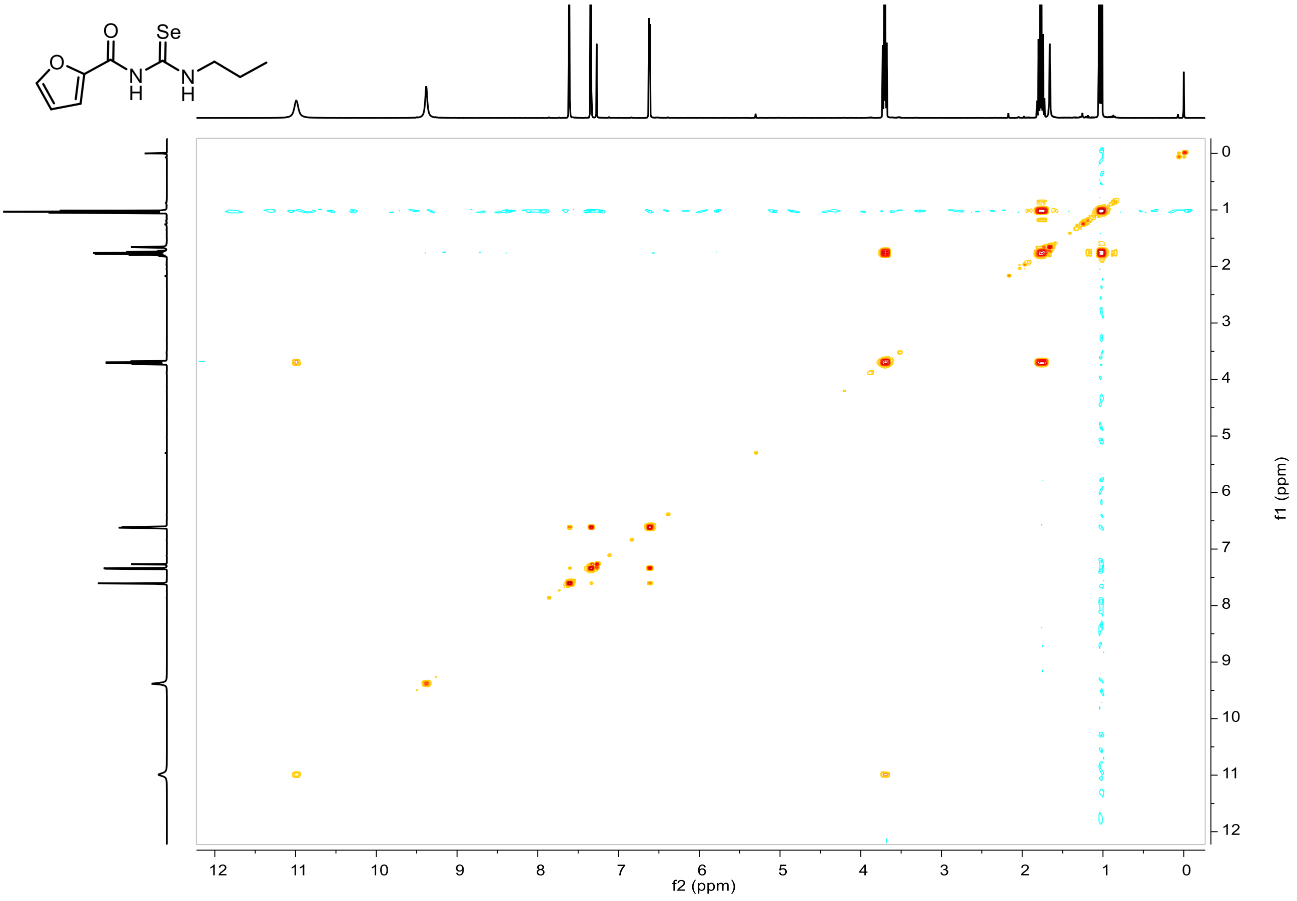

Figure S4. COSY-NMR spectrum of compound **1.I**.

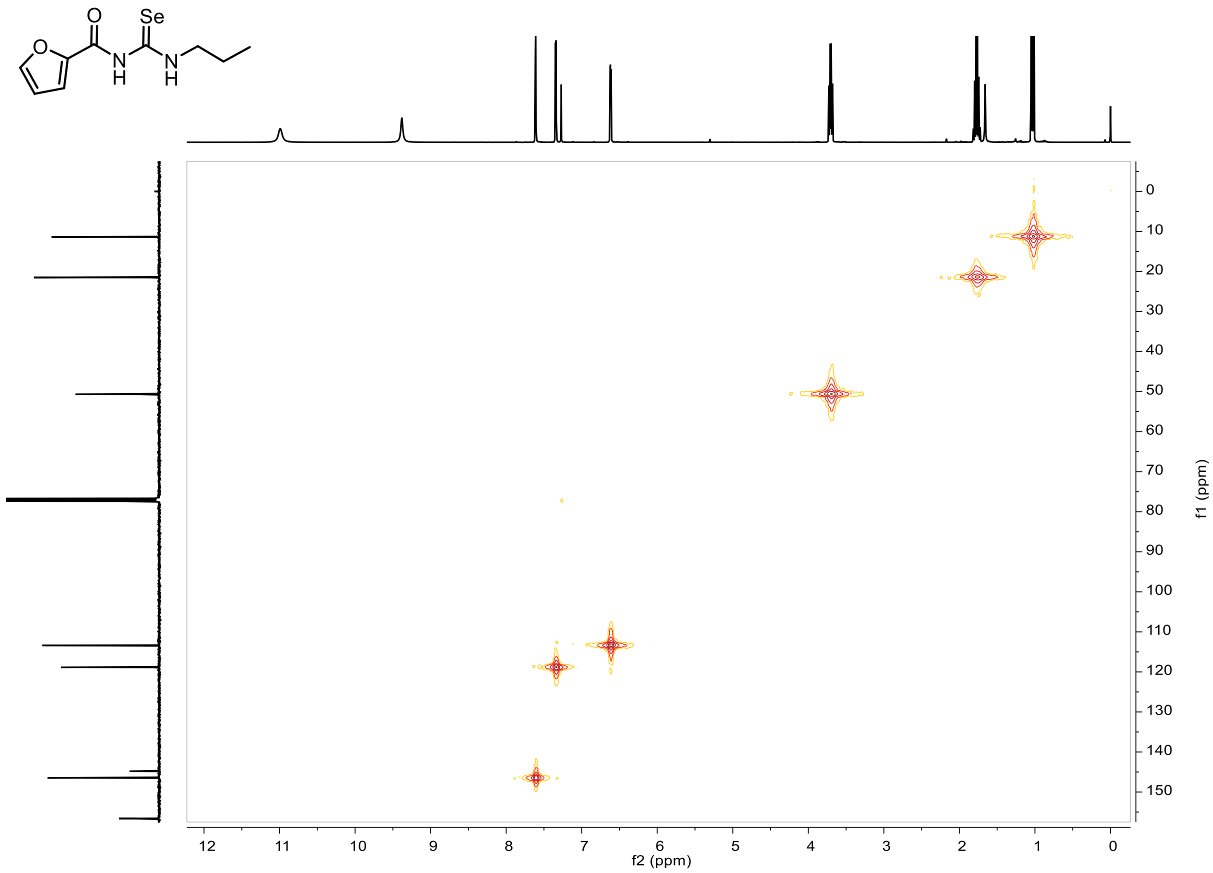

Figure S5. HMQC-NMR spectrum of compound **1.I**.

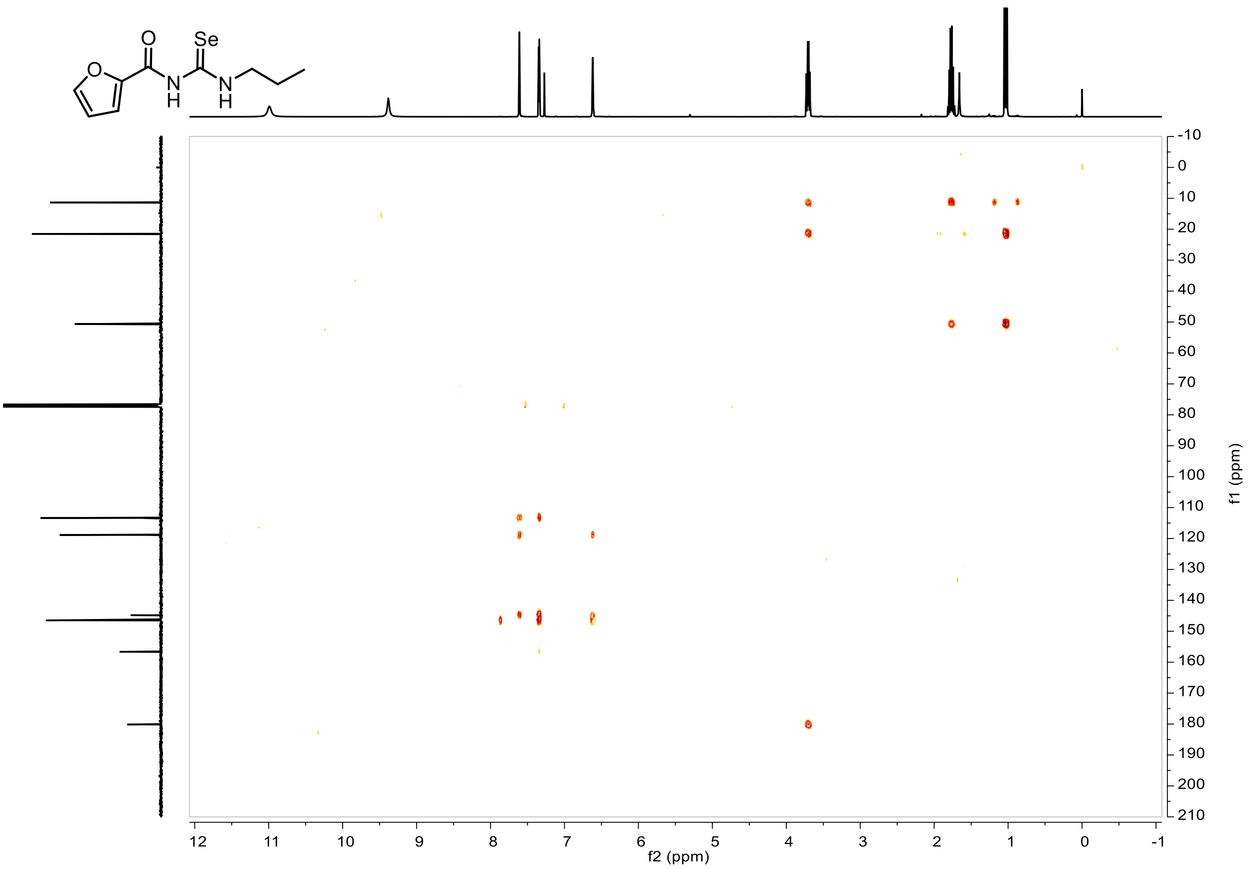

Figure S6. HMBC-NMR spectrum of compound **1.I**.

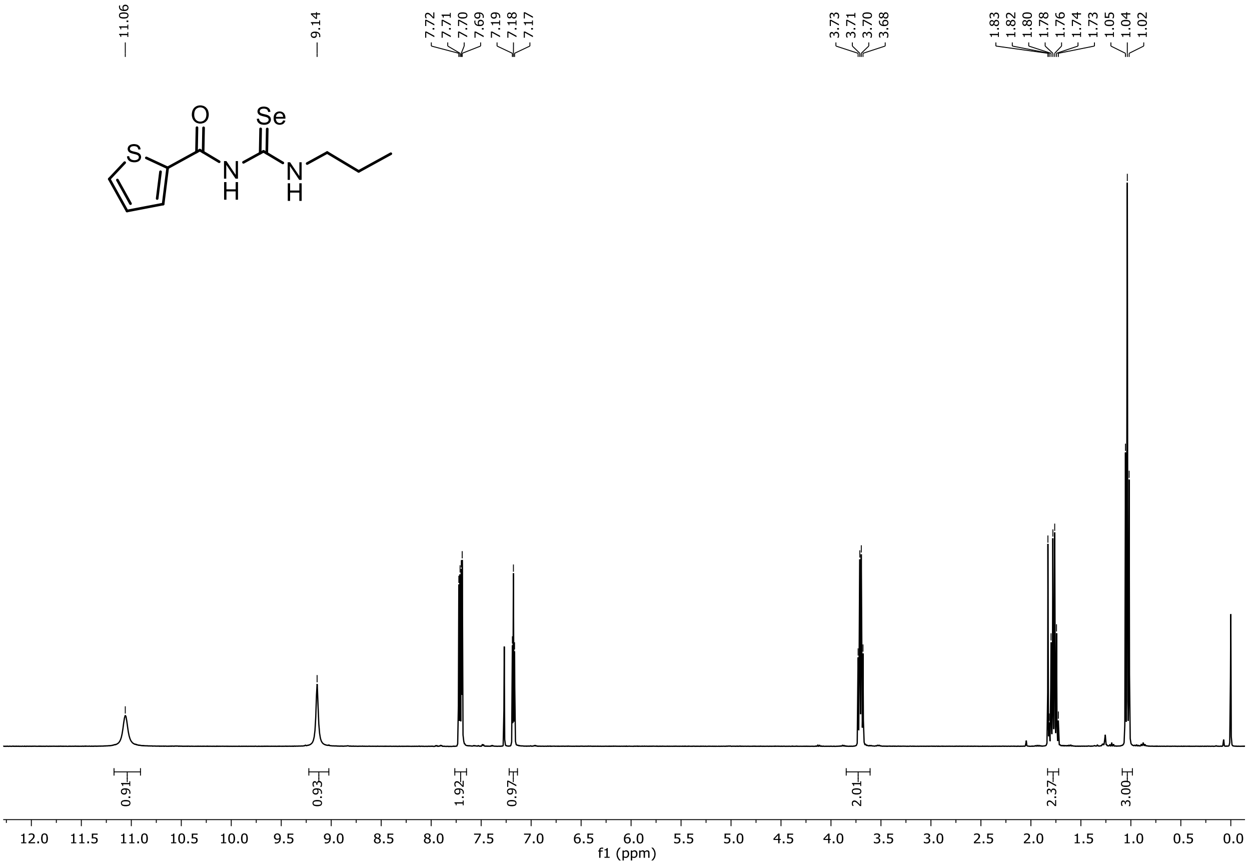

Figure S7. ^{1}H-NMR spectrum of compound **2.I**.

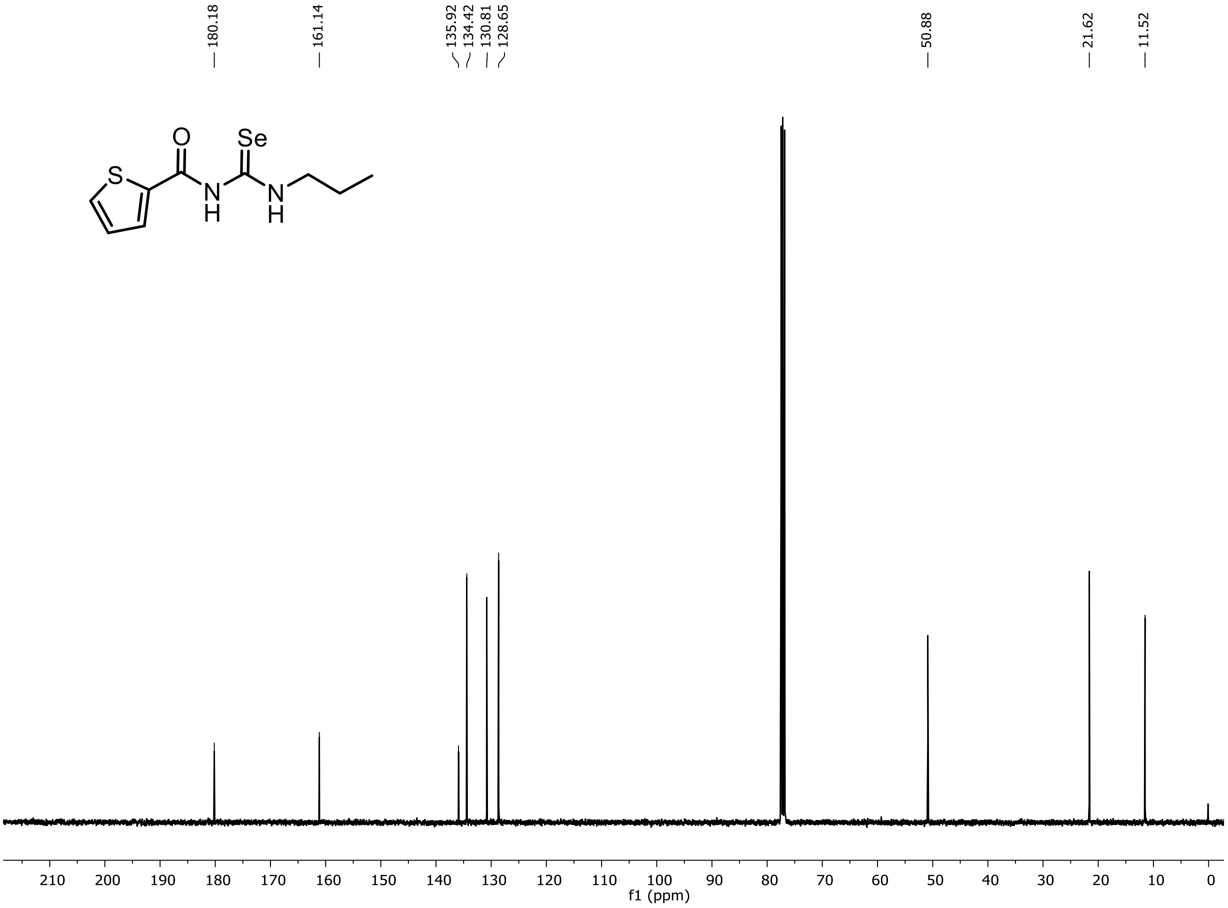

Figure S8. ^{13}C-NMR spectrum of compound **2.I.**

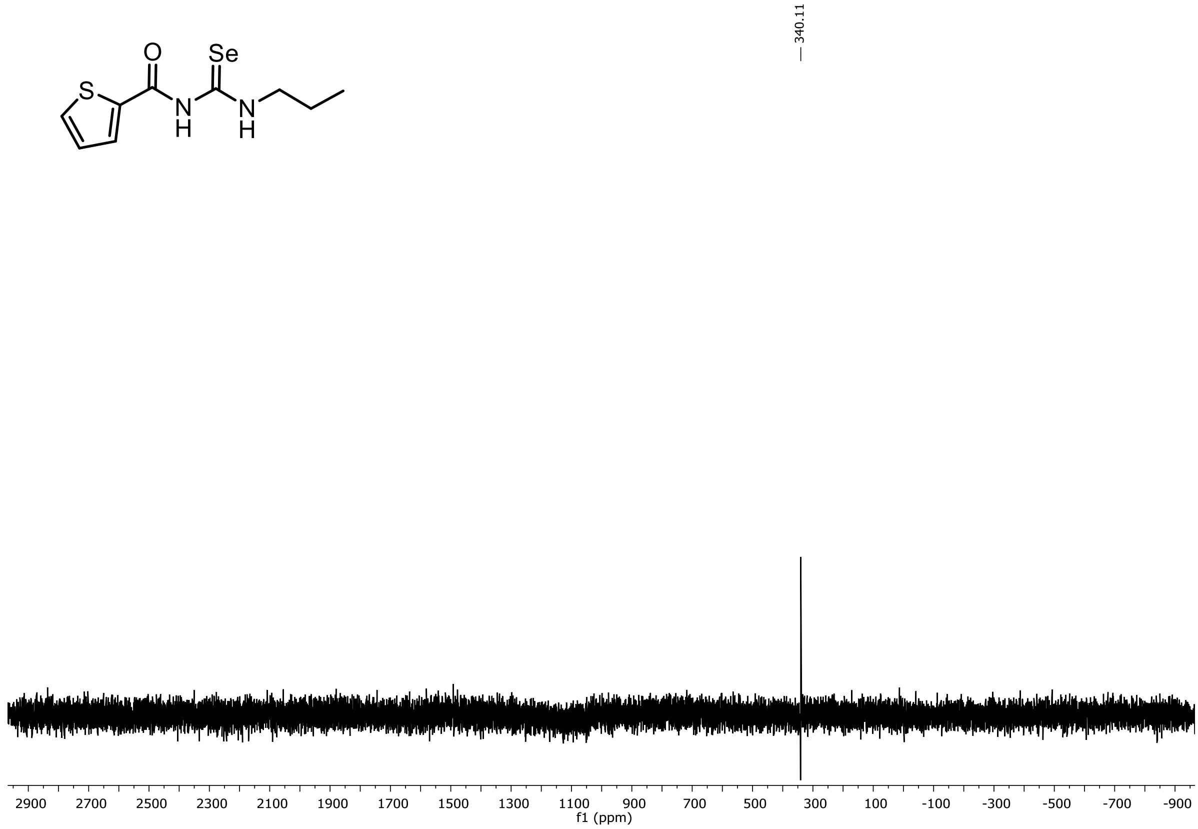

Figure S9. ^{77}Se-NMR spectrum of compound **2.I**.

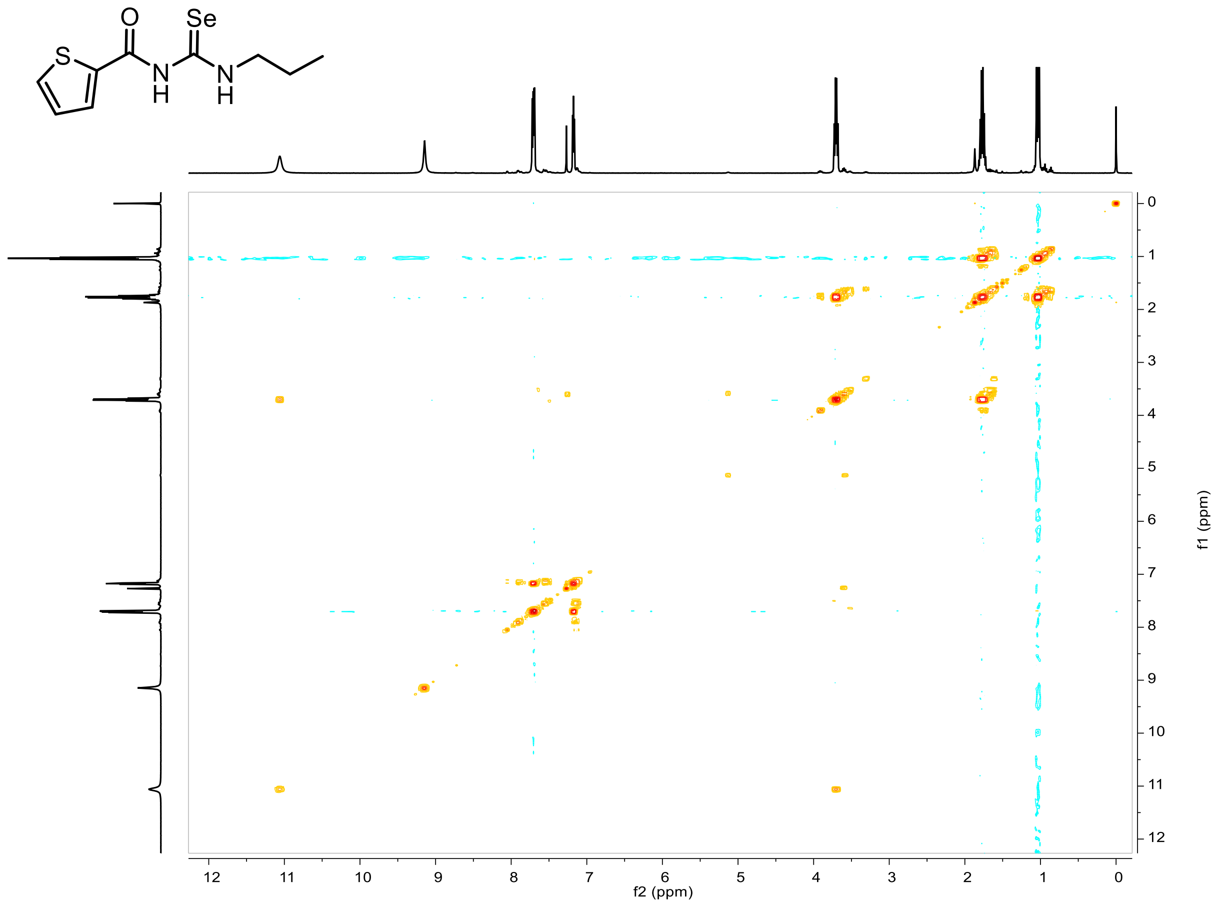

Figure S10. COSY-NMR spectrum of compound **2.I**.

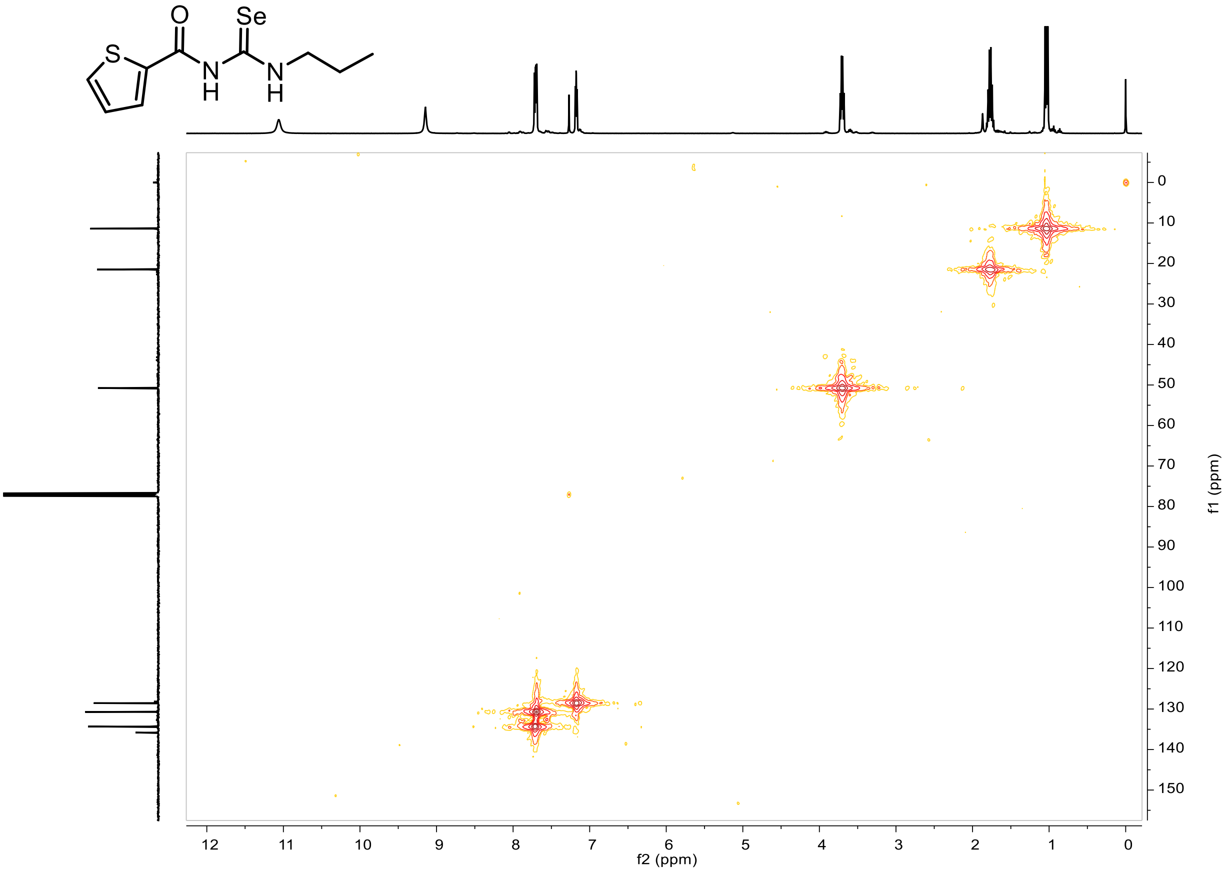

Figure S11. HMQC-NMR spectrum of compound **2.I**.

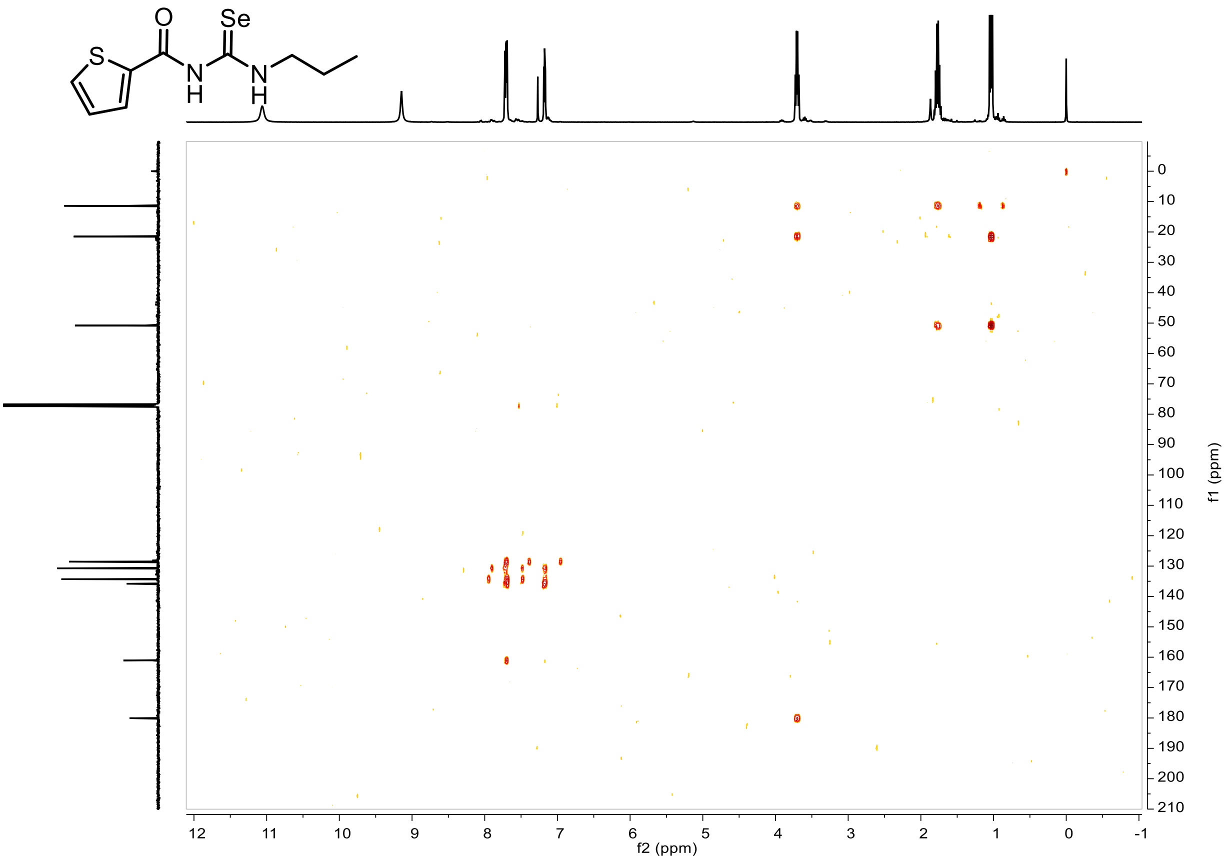

Figure S12. HMBC-NMR spectrum of compound **2.I**.

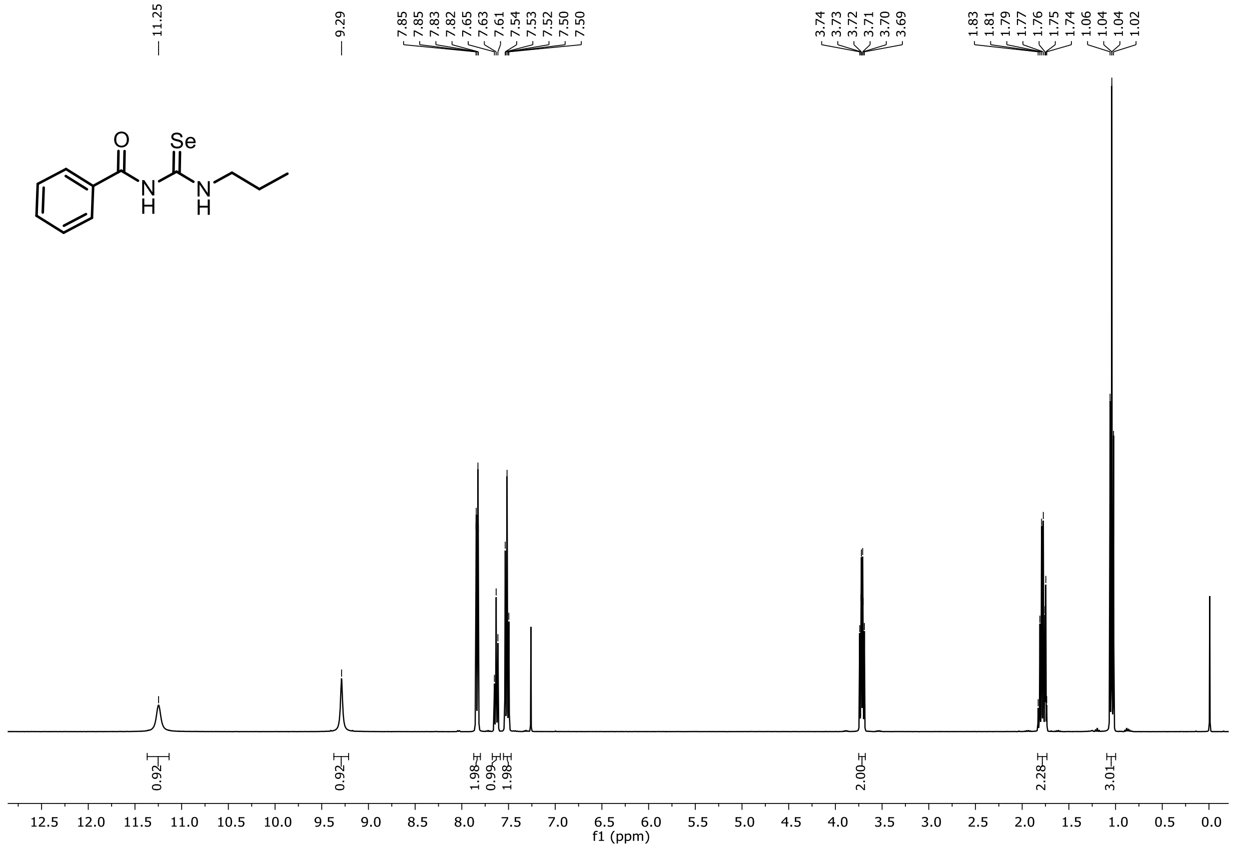

Figure S13. ^{1}H-NMR spectrum of compound **5.I**.

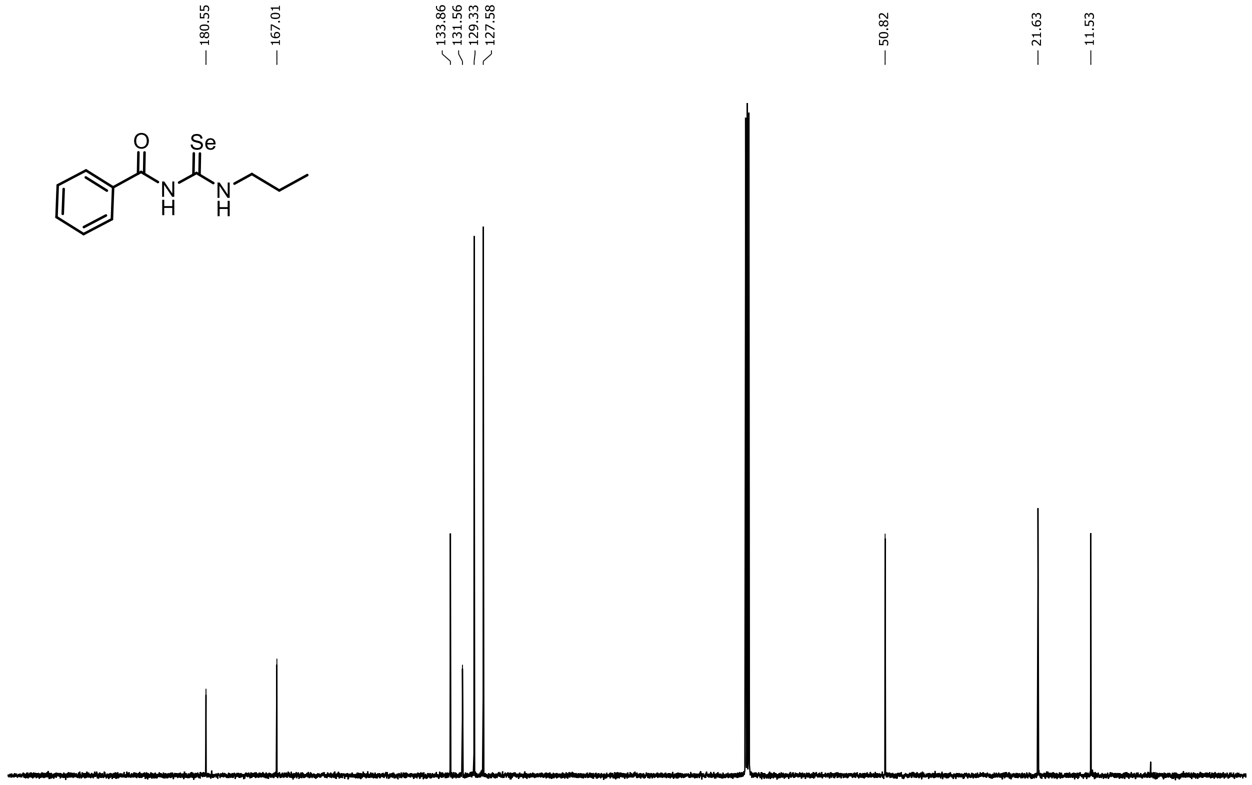

Figure S14. ^{13}C-NMR spectrum of compound **5.I.**

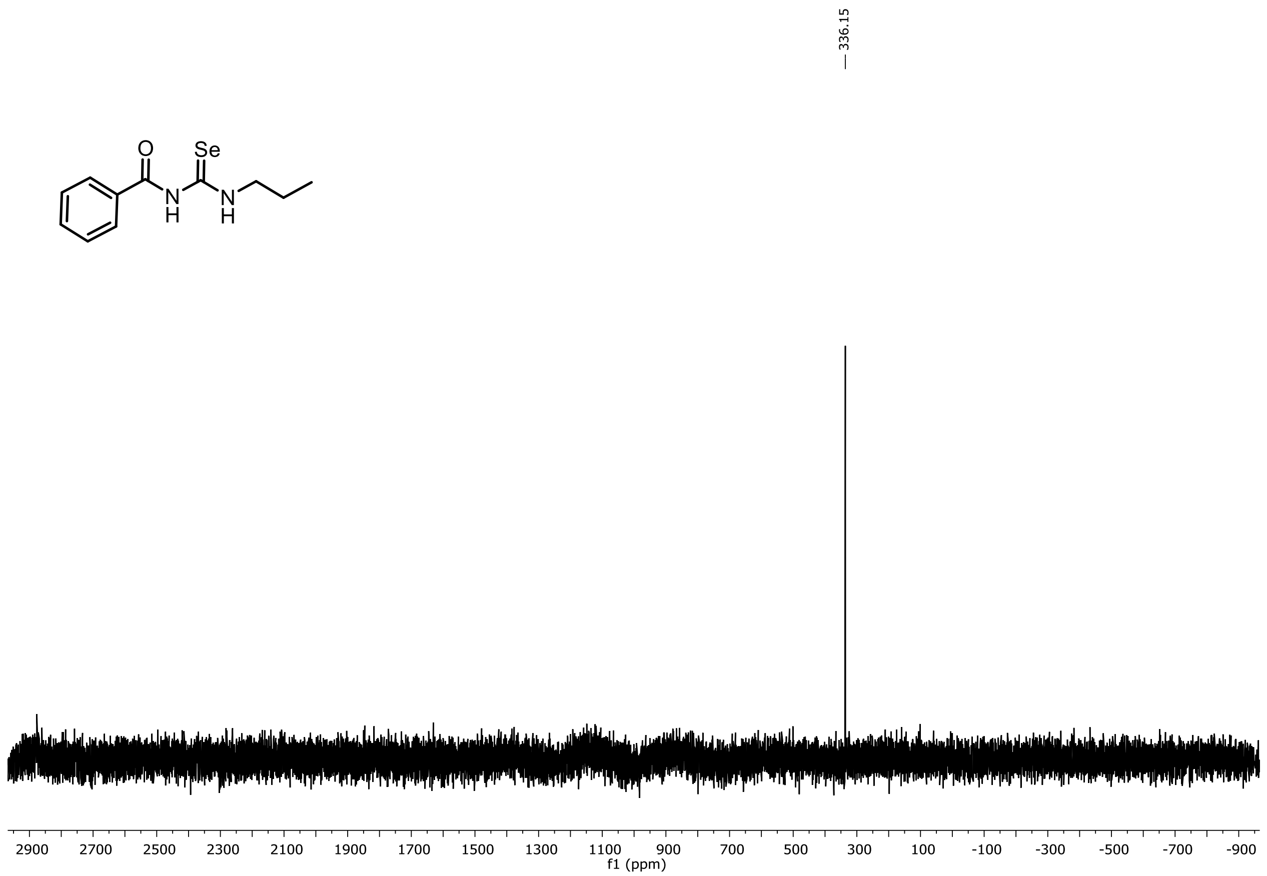

Figure S15. ^{77}Se-NMR spectrum of compound **5.I**.

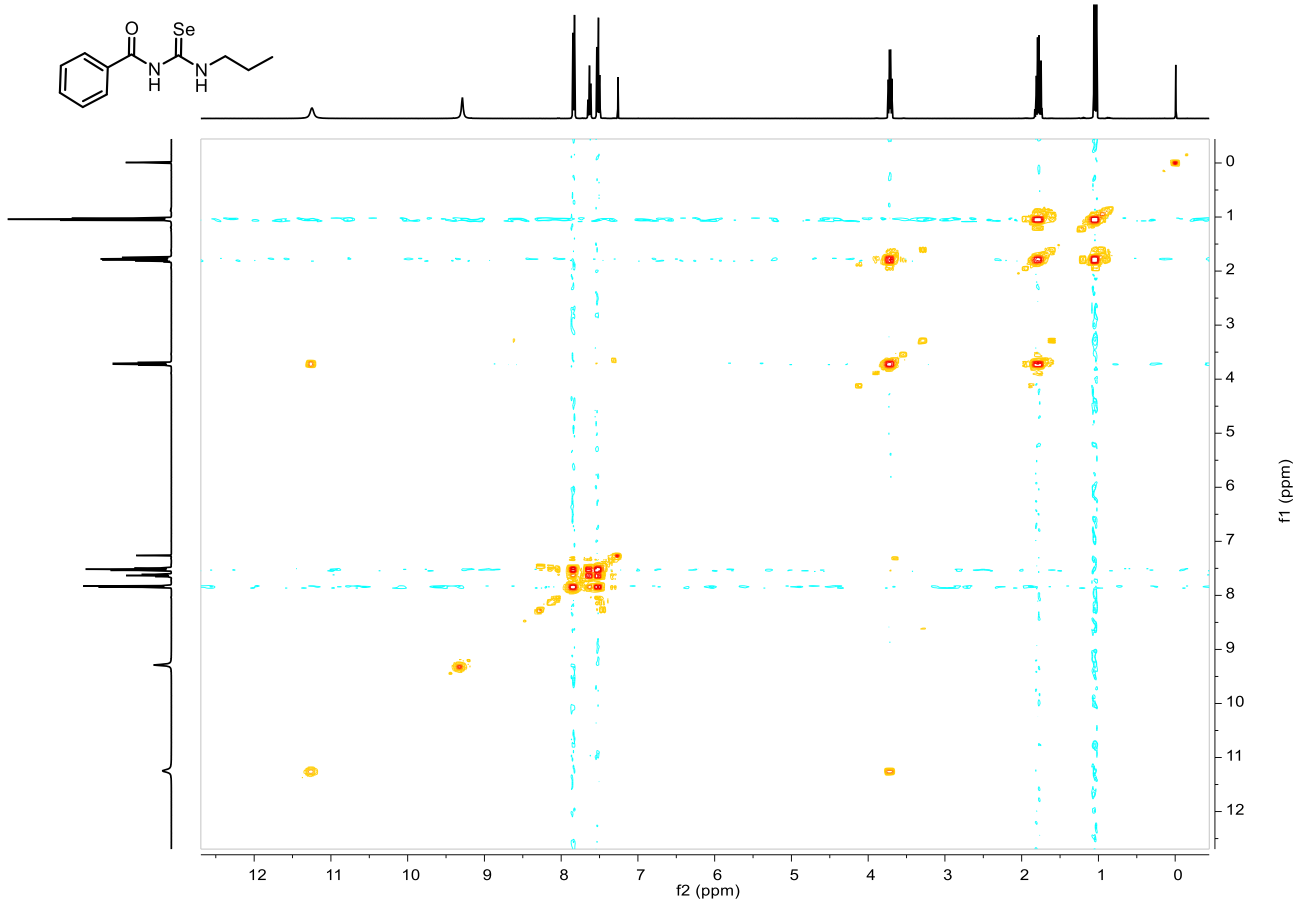

Figure S16. COSY-NMR spectrum of compound **5.I**.

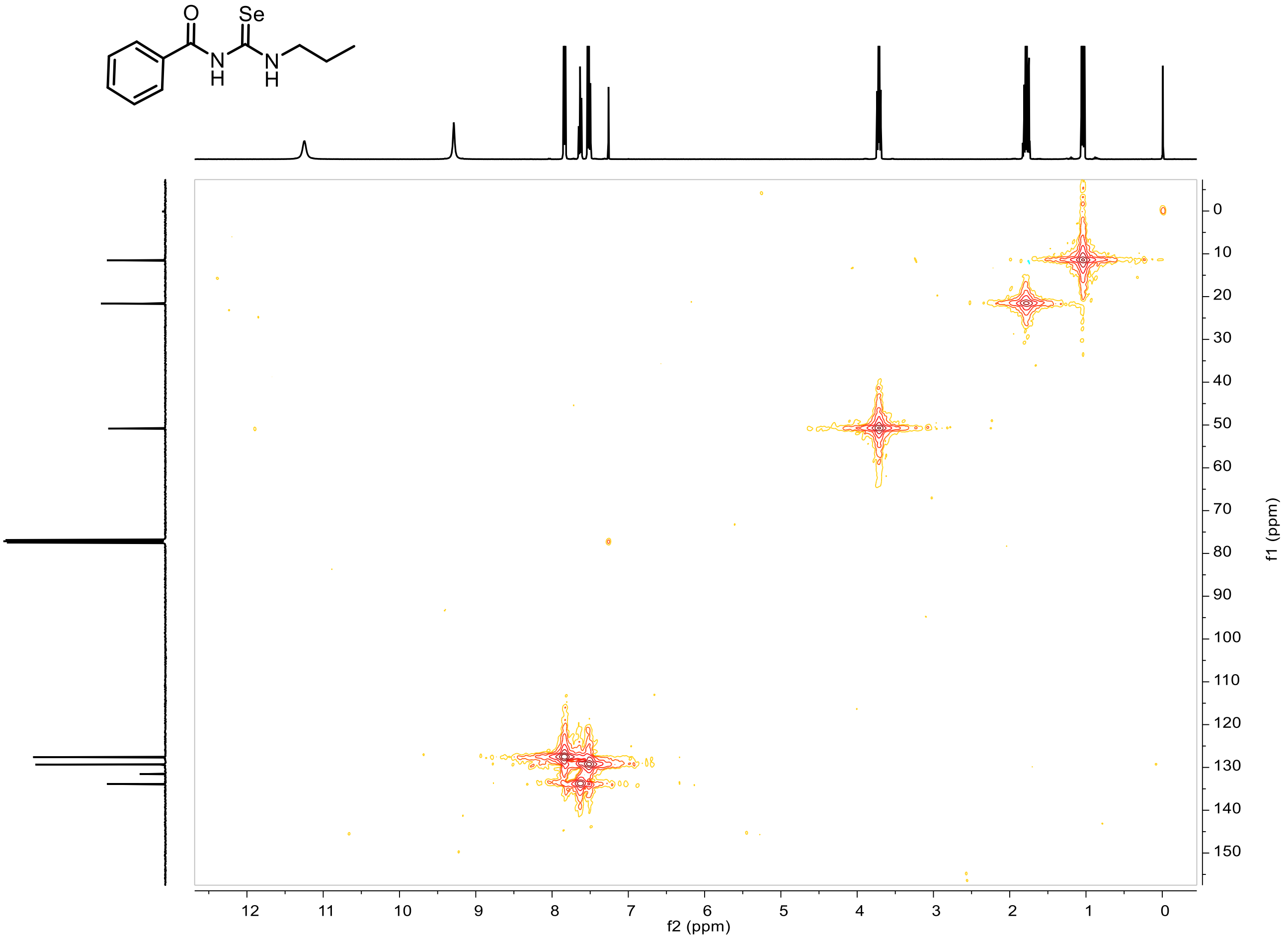

Figure S17. HMQC-NMR spectrum of compound **5.I**.

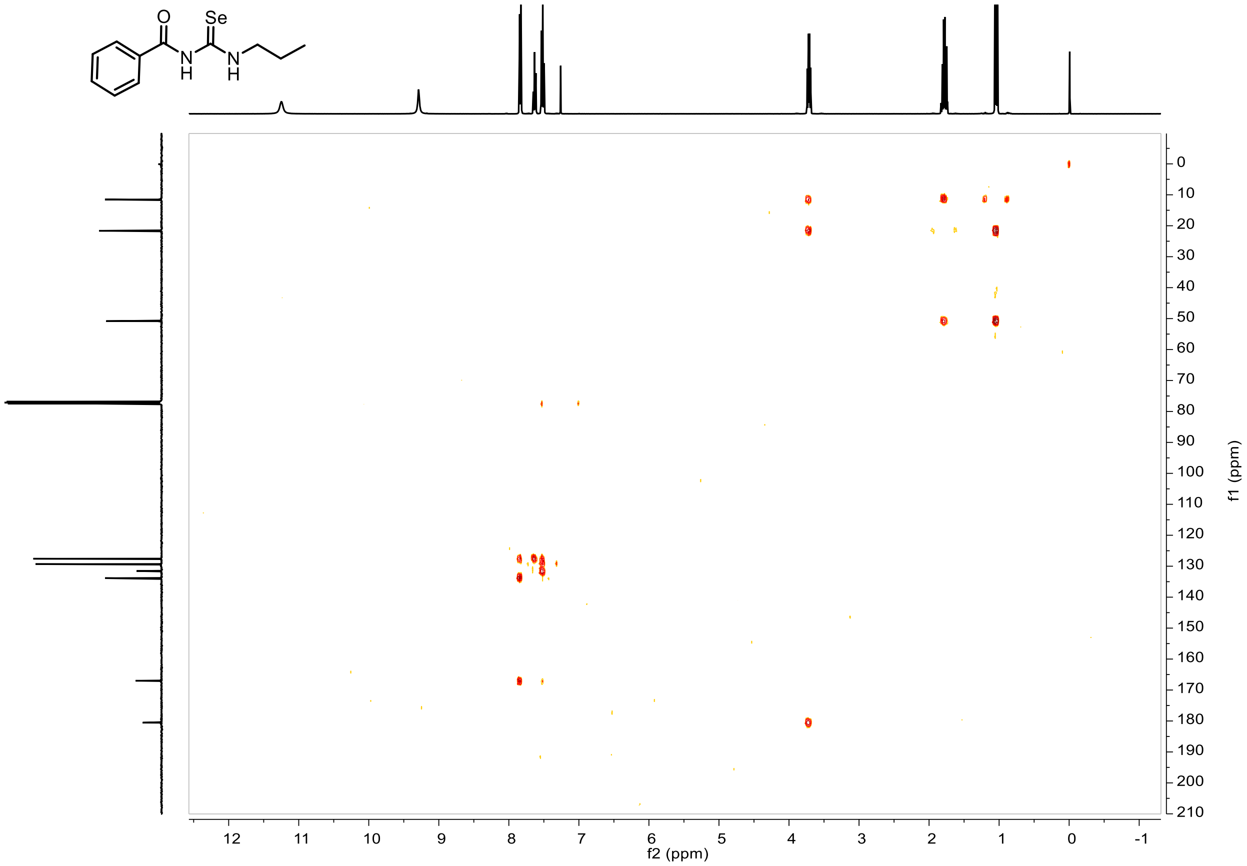

Figure S18. HMBC-NMR spectrum of compound **5.I**.

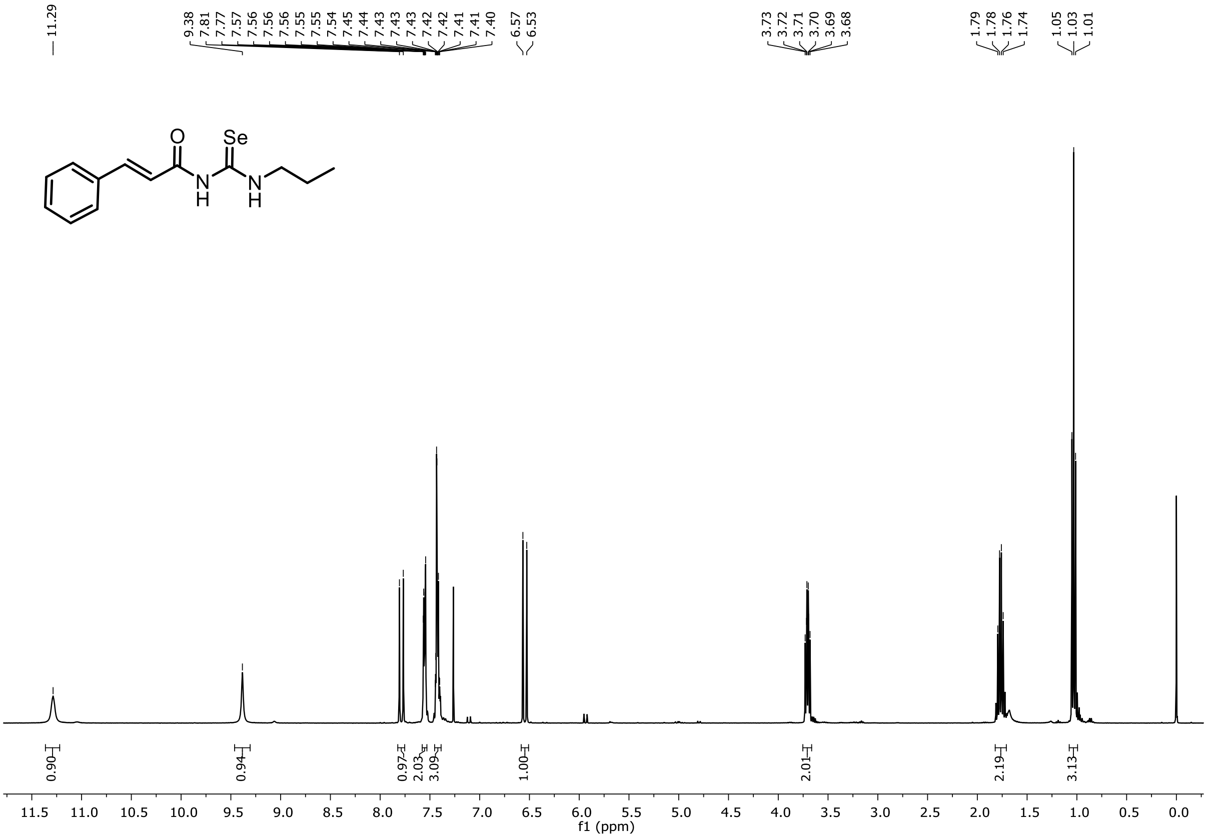

Figure S19. ^{1}H-NMR spectrum of compound **6.I**.

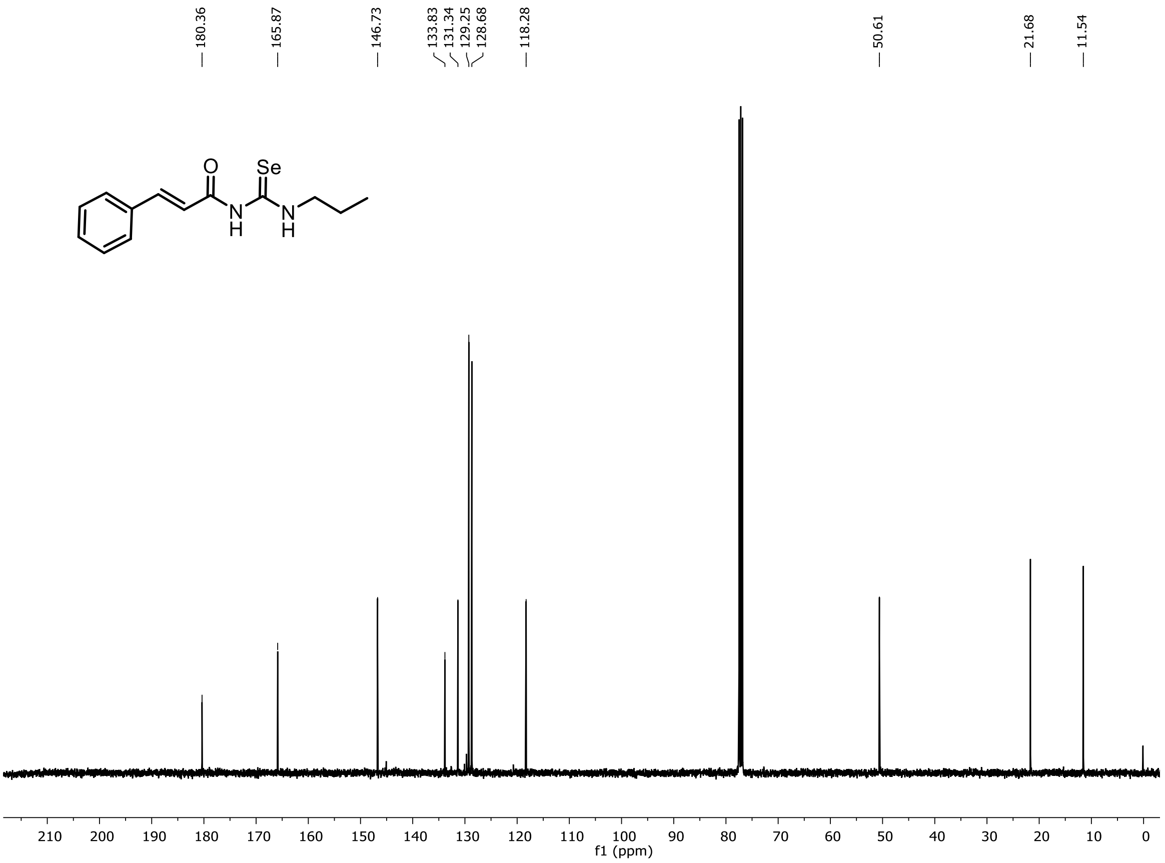

Figure S20. ^{13}C-NMR spectrum of compound **6.I.**

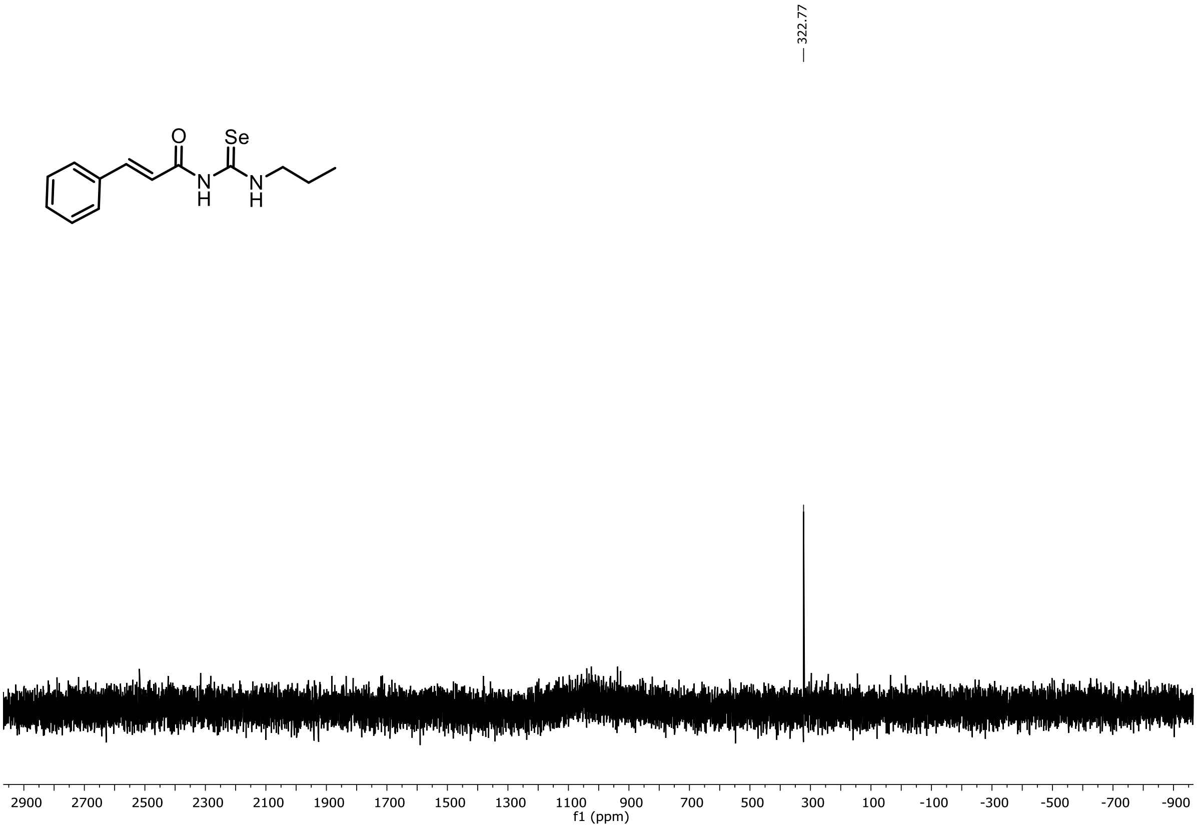

Figure S21. ^{77}Se-NMR spectrum of compound **6.I.**

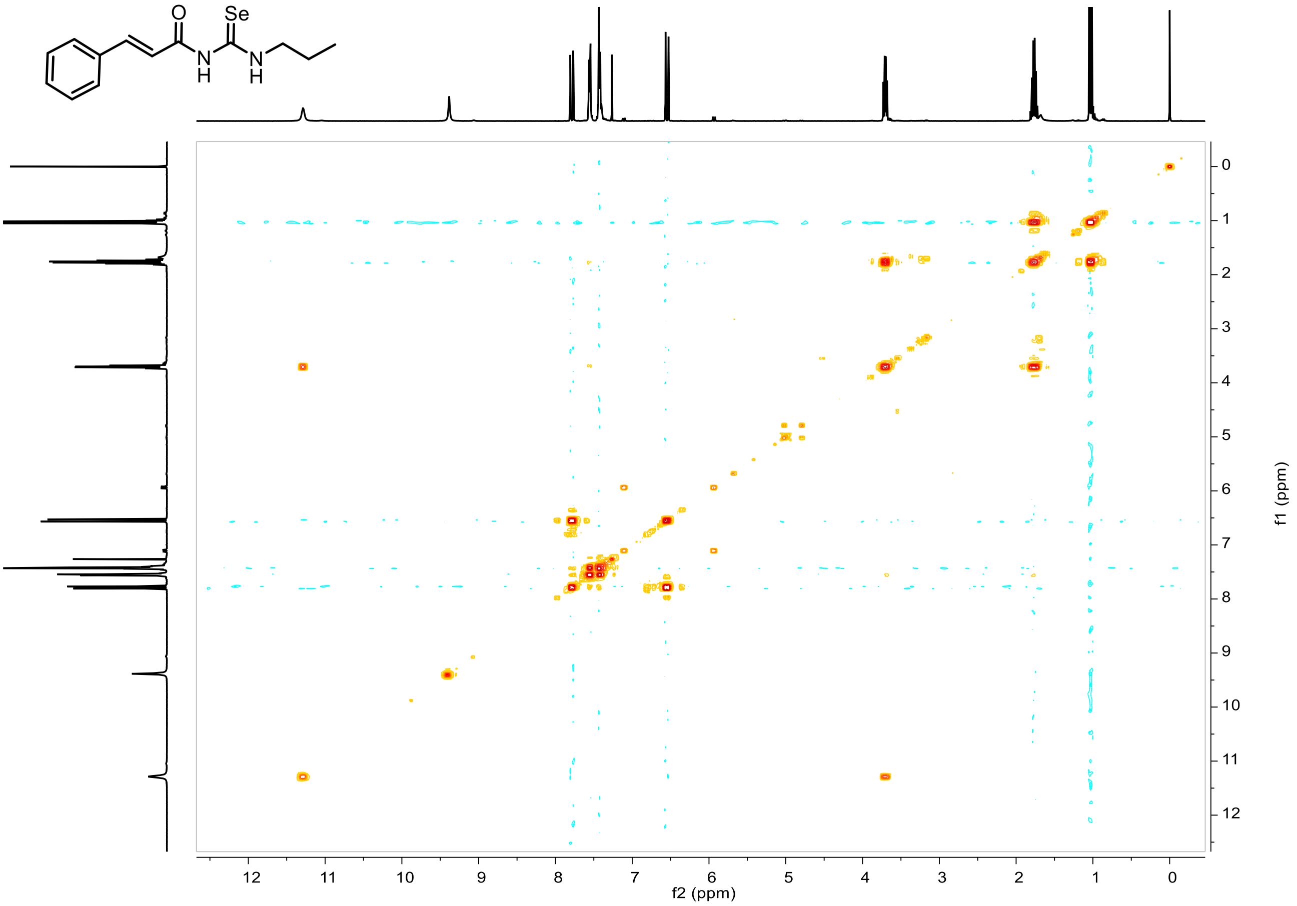

Figure S22. COSY-NMR spectrum of compound **6.I.**

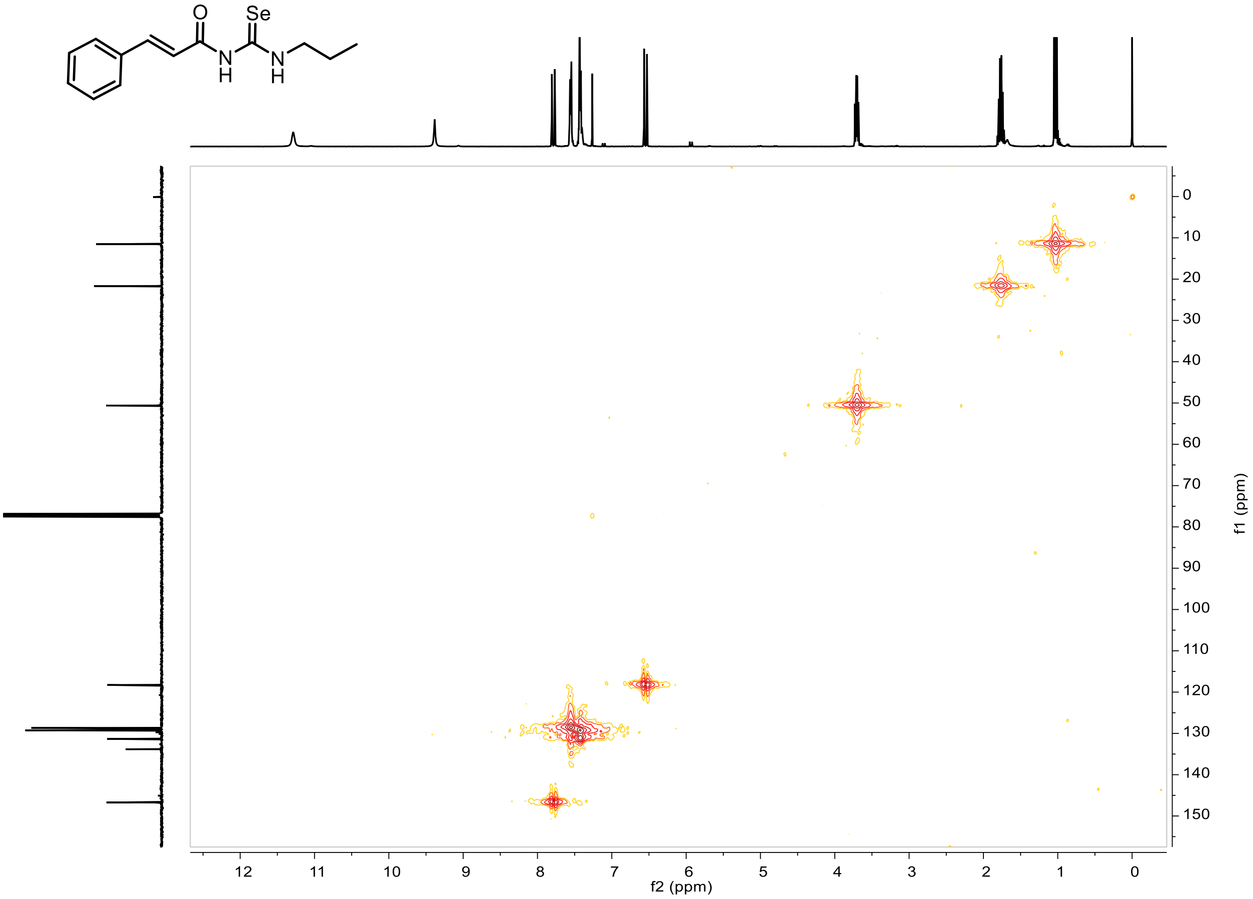

Figure S23. HMQC-NMR spectrum of compound **6.I.**

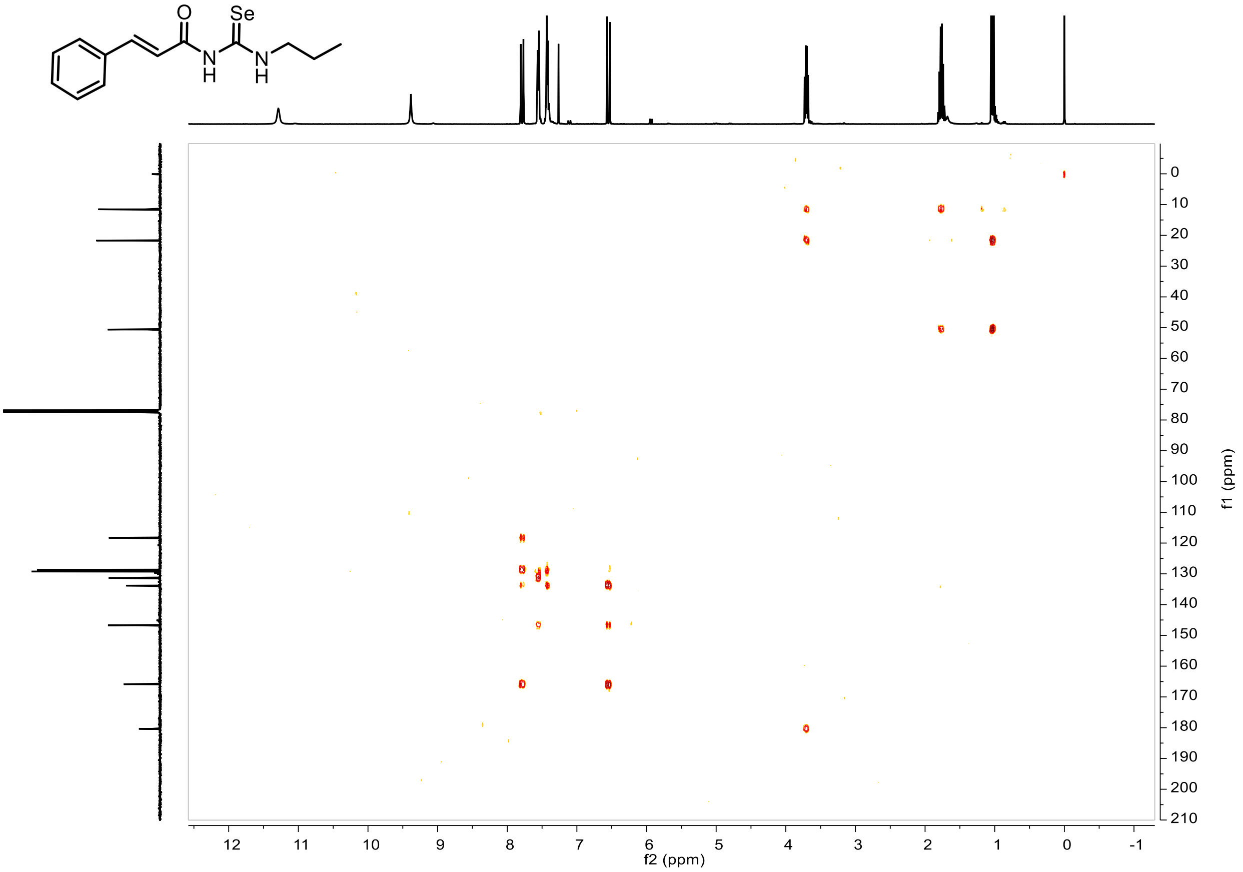

Figure S24. HMBC-NMR spectrum of compound **6.I.**

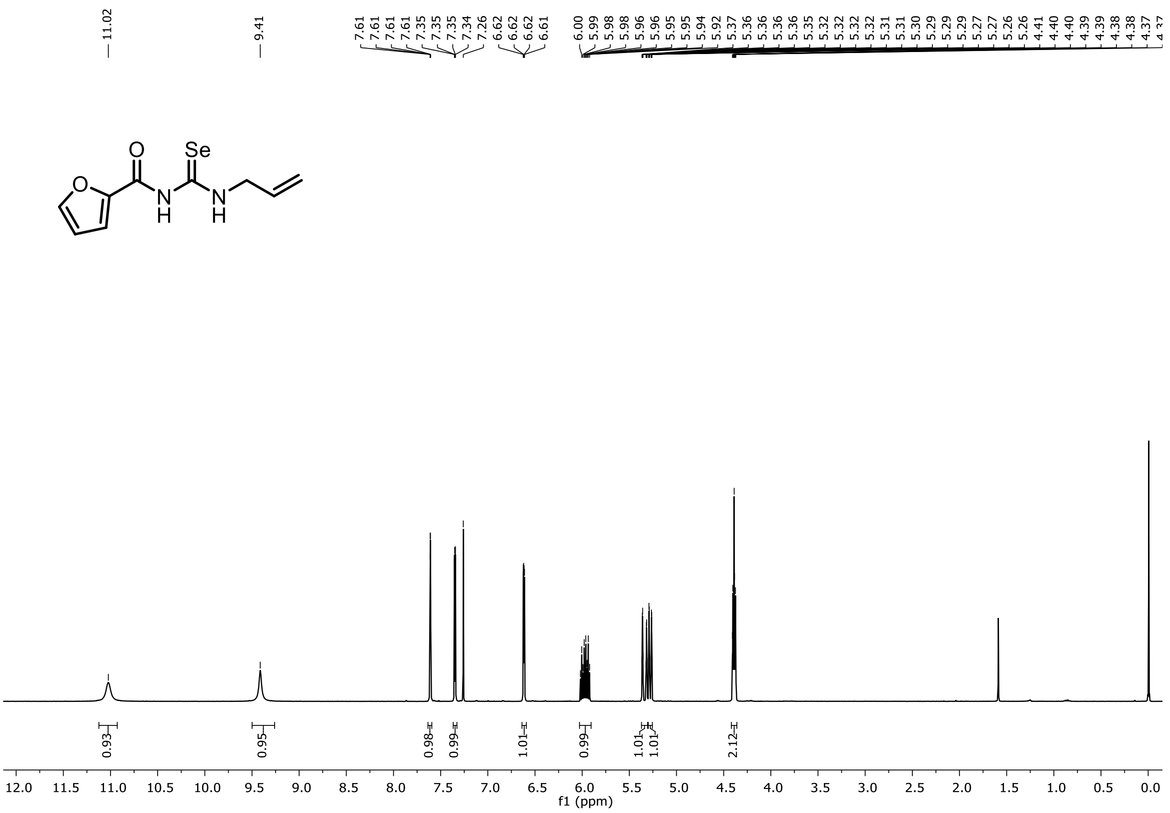

Figure S25. ^{1}H-NMR spectrum of compound **1.II**.

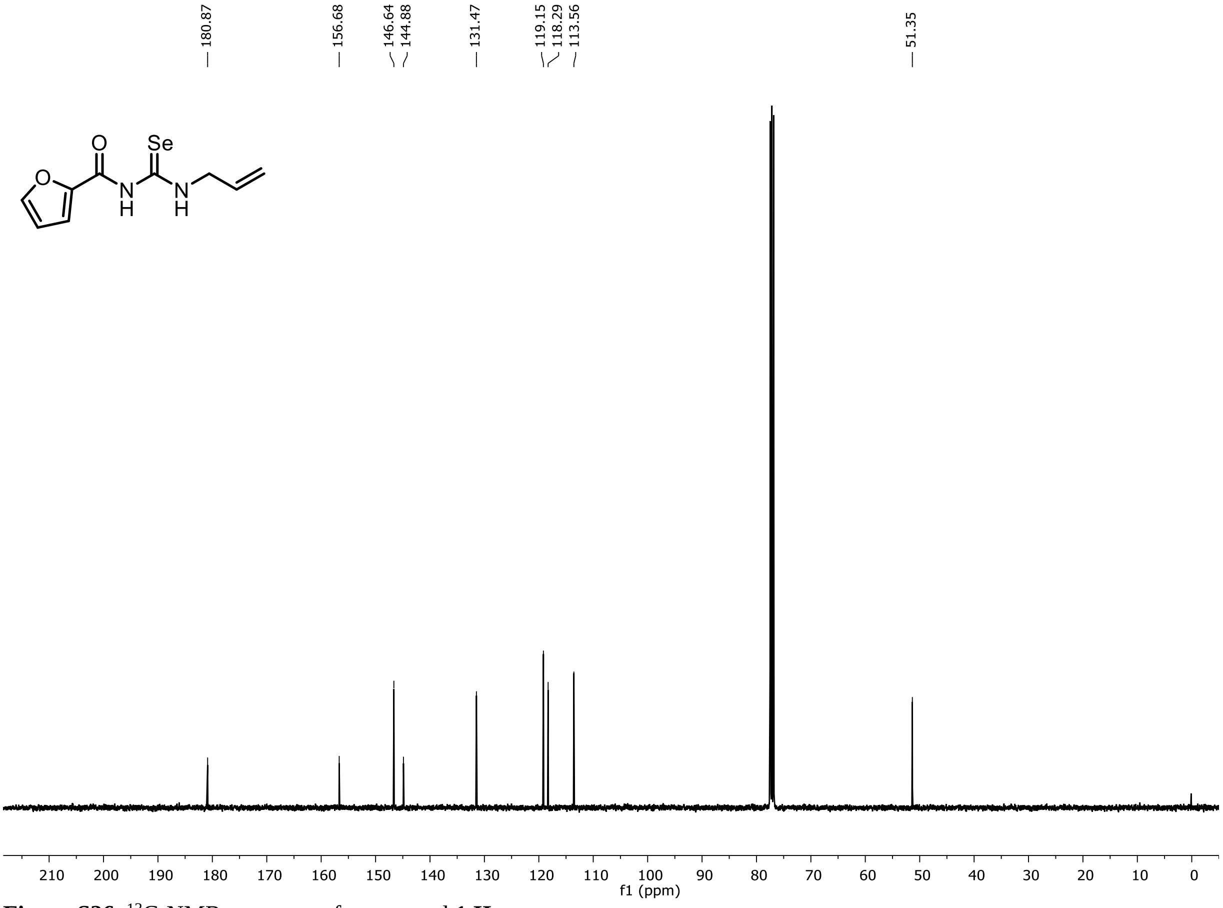

Figure S26. ^{13}C-NMR spectrum of compound **1.II**.

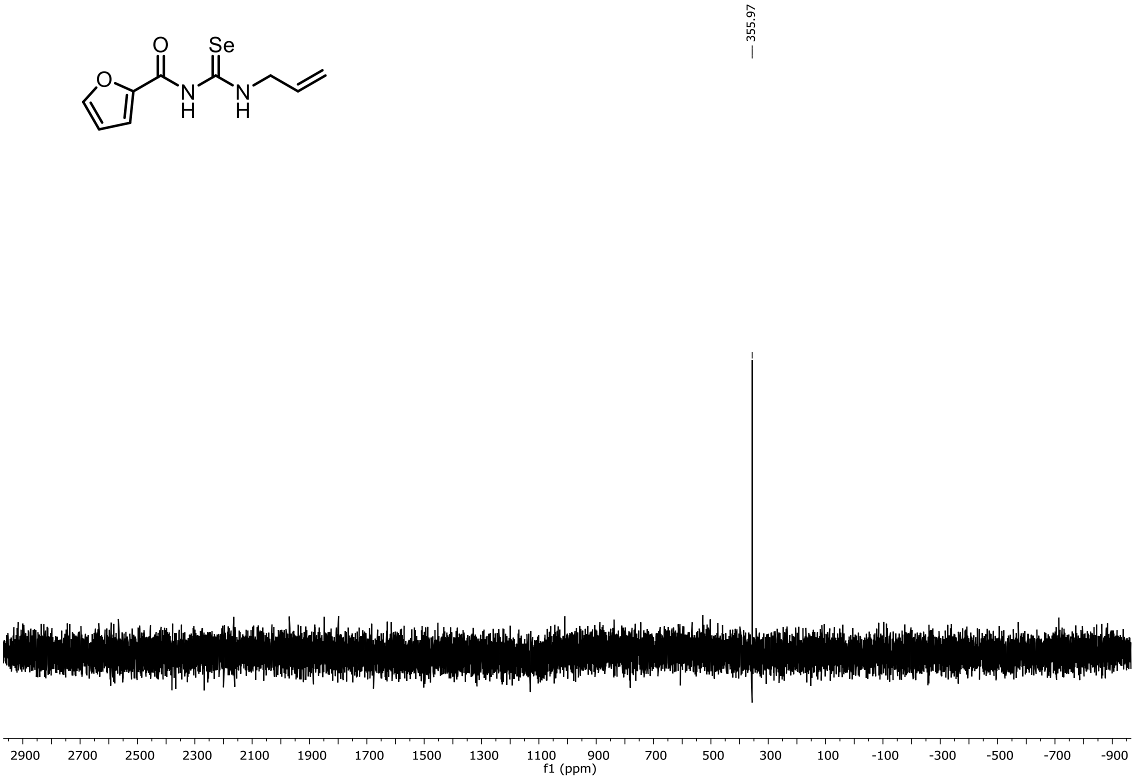

Figure S27. ^{77}Se-NMR spectrum of compound **1.II**.

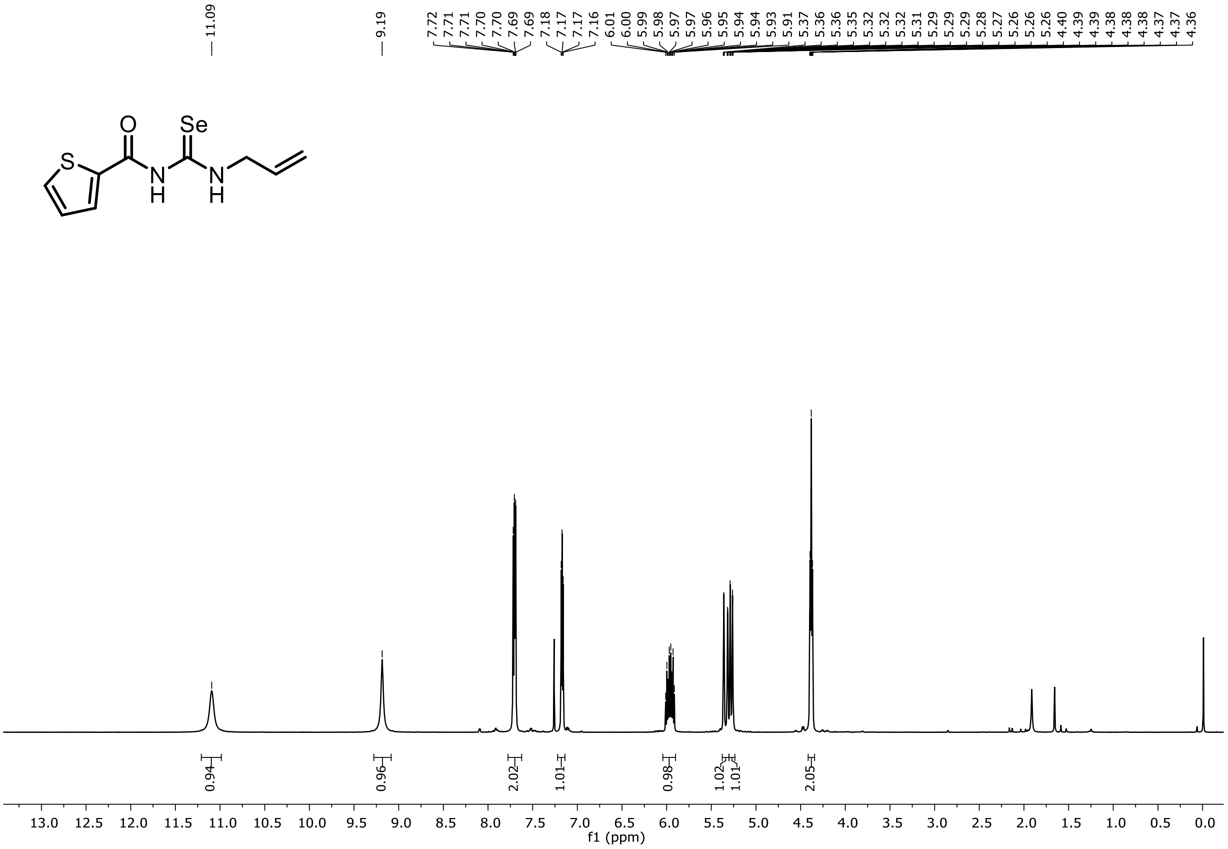

Figure S28. ^{1}H-NMR spectrum of compound **2.II**.

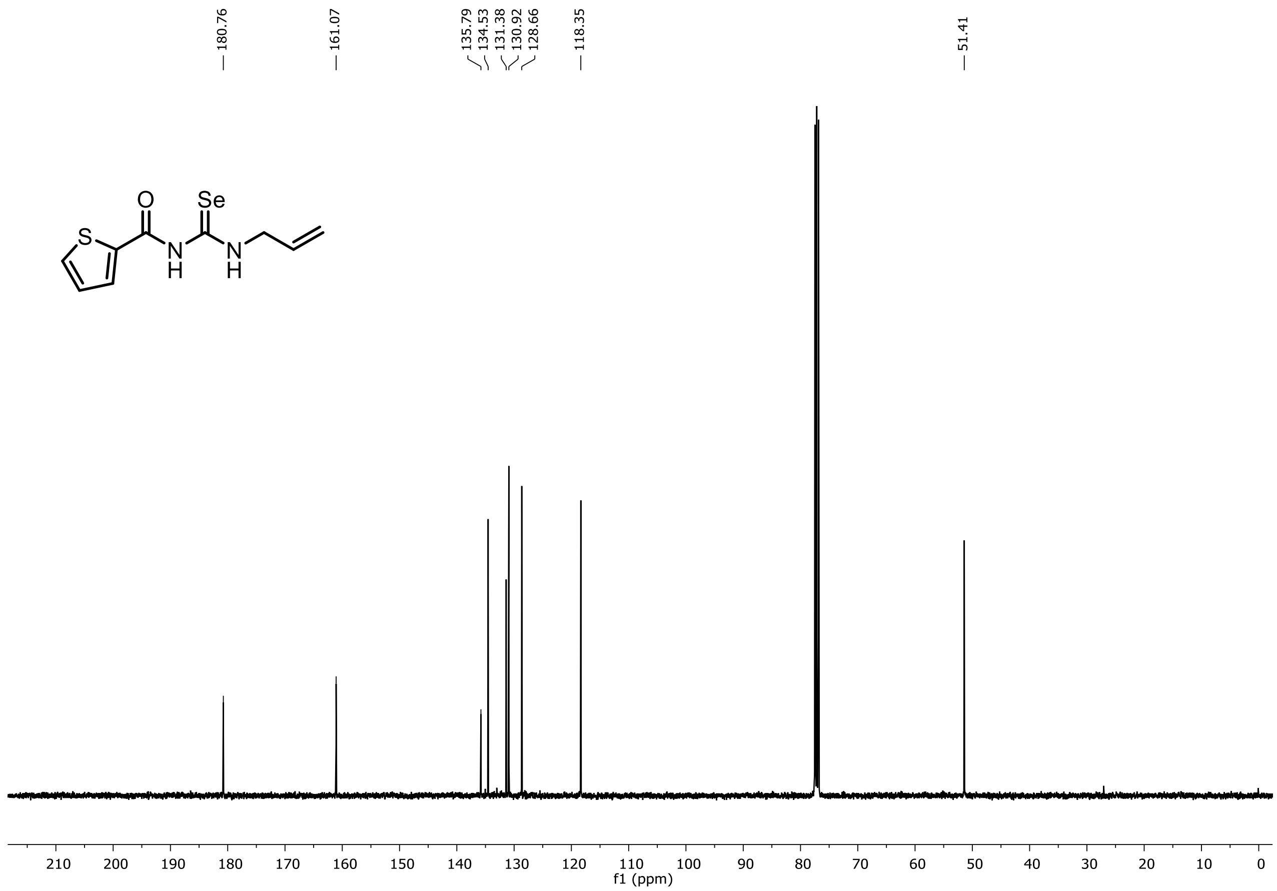

Figure S29. ^{13}C-NMR spectrum of compound **2.II**.

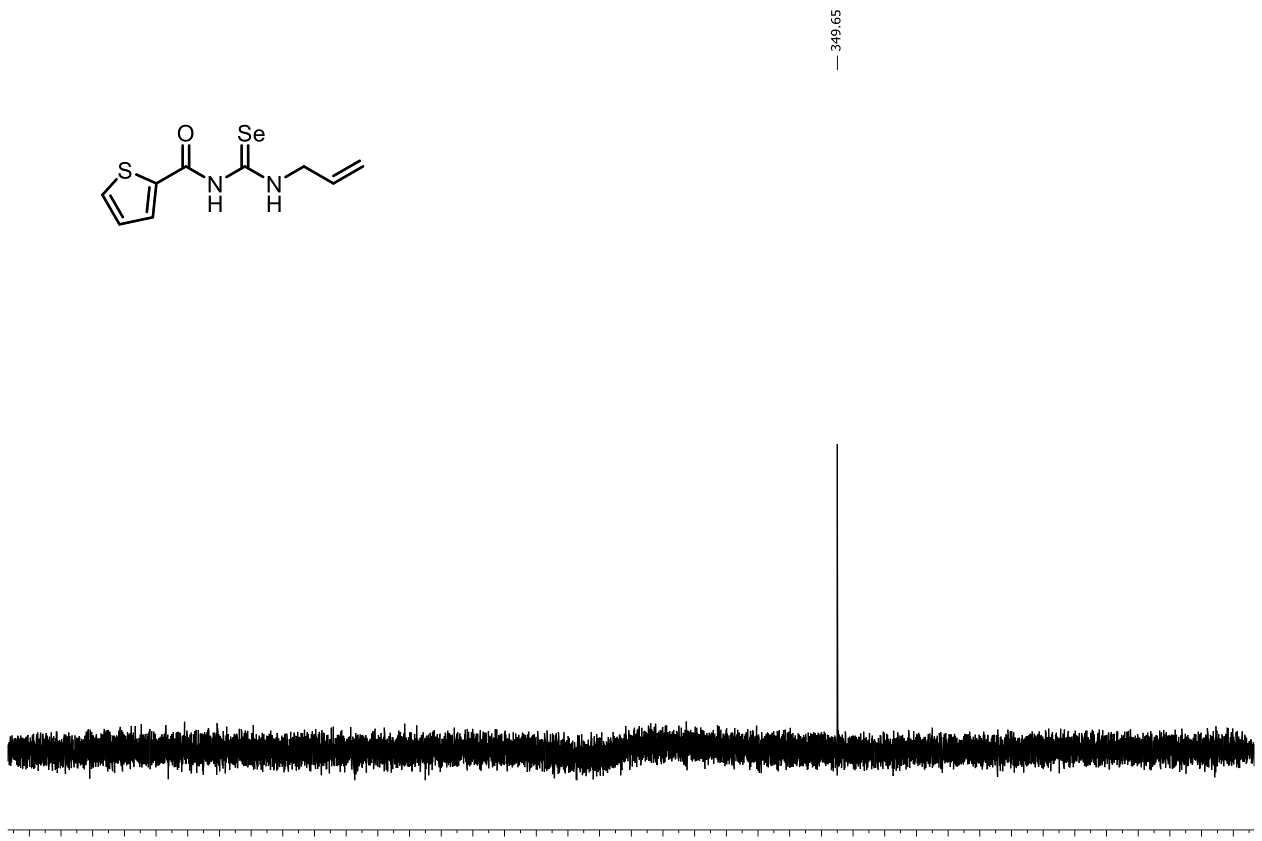

Figure S30. ^{77}Se-NMR spectrum of compound **2.II**.

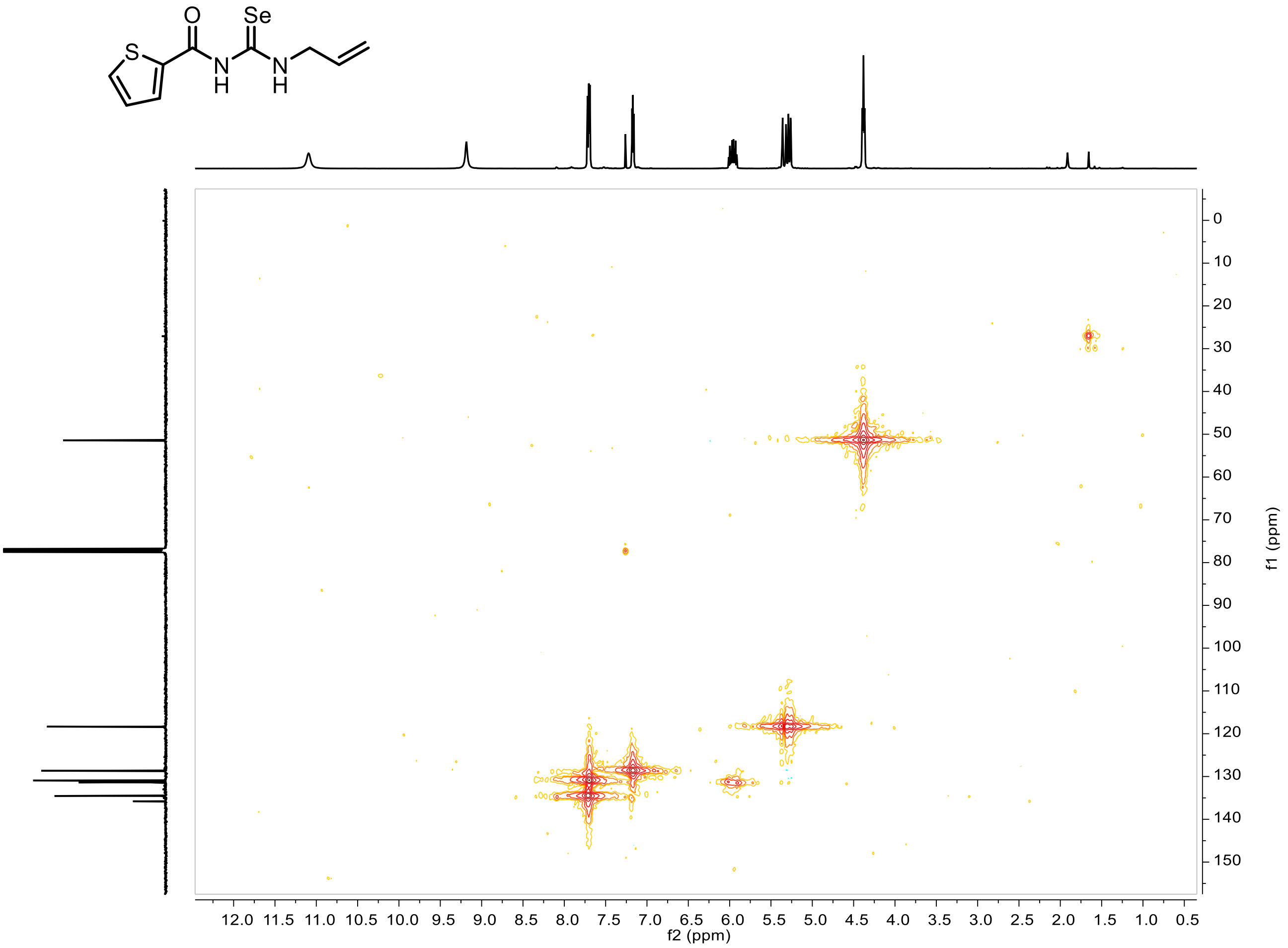

Figure S31. HMQC-NMR spectrum of compound **2.II**.

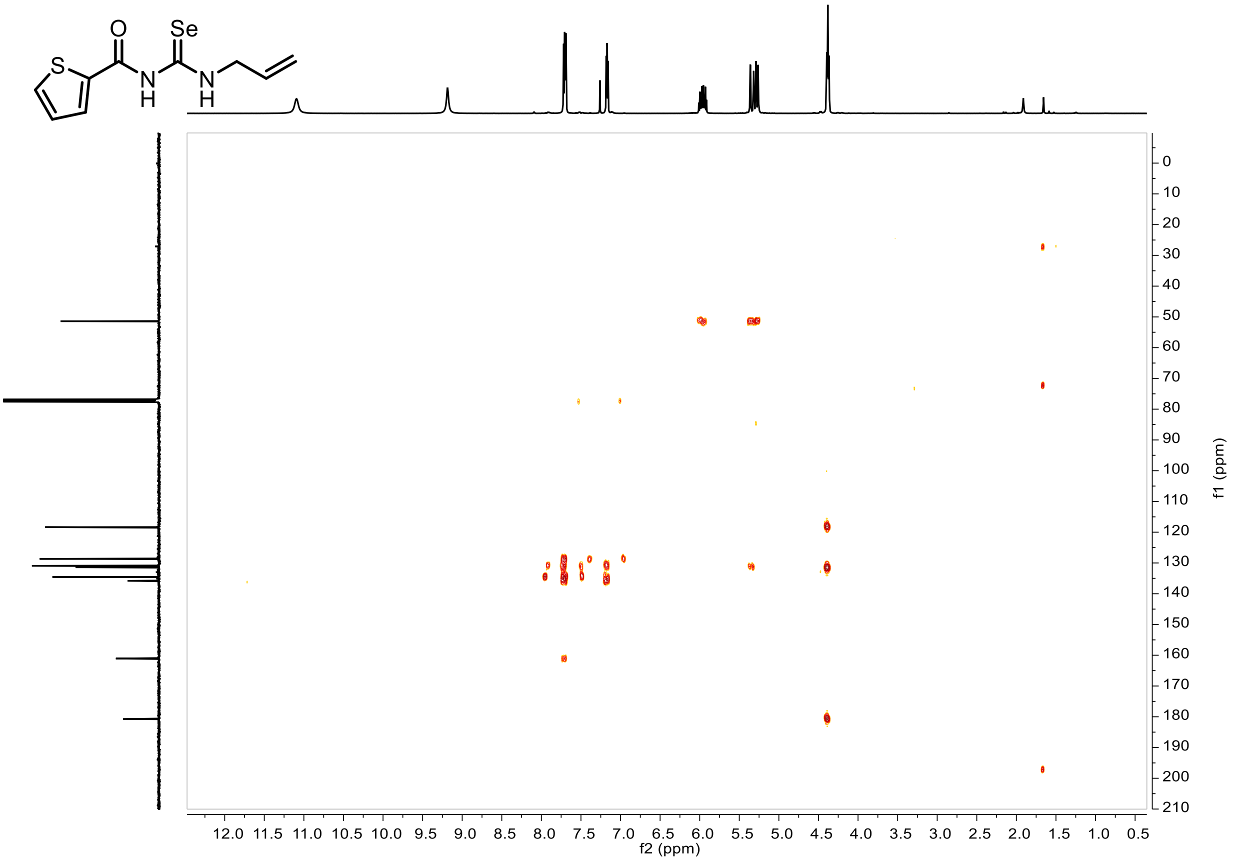

Figure S32. HMBC-NMR spectrum of compound **2.II**

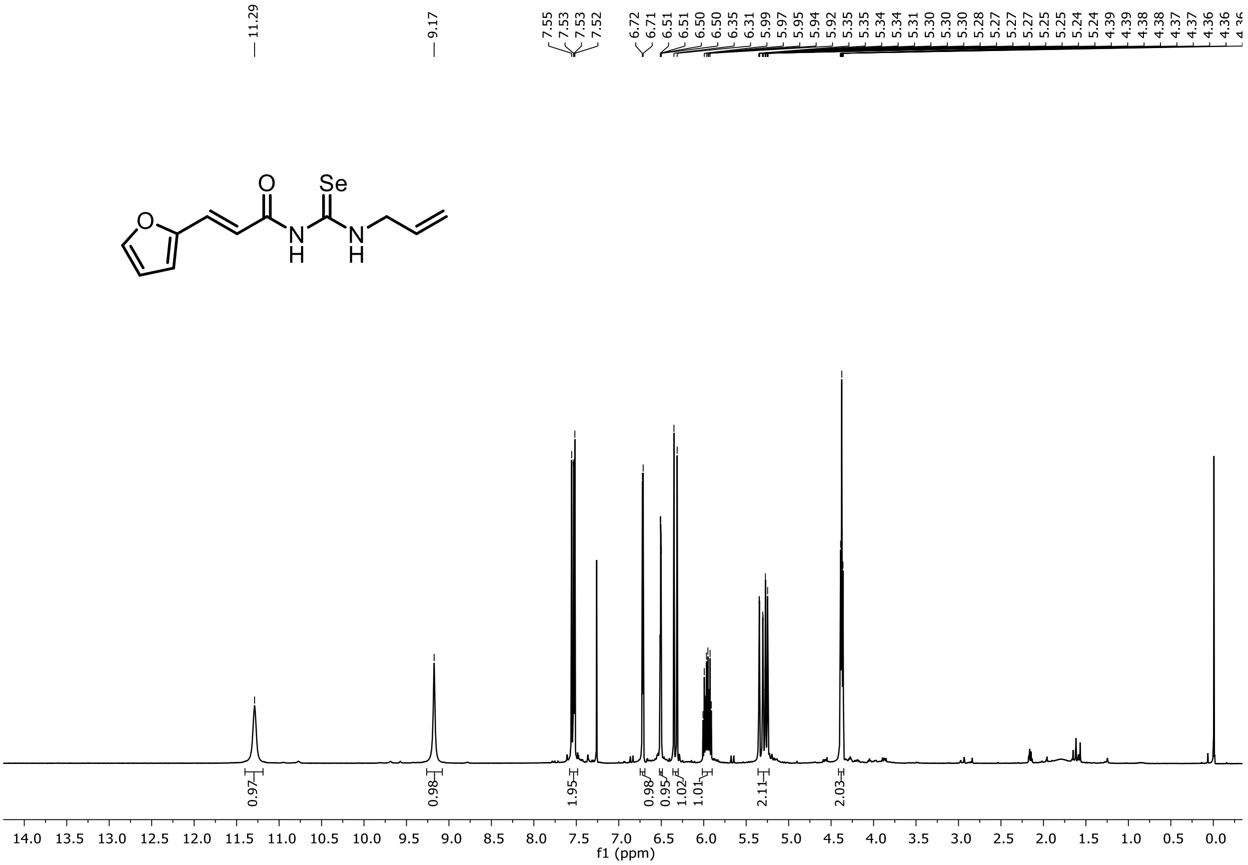

Figure S33. ^{1}H-NMR spectrum of compound **3.II**.

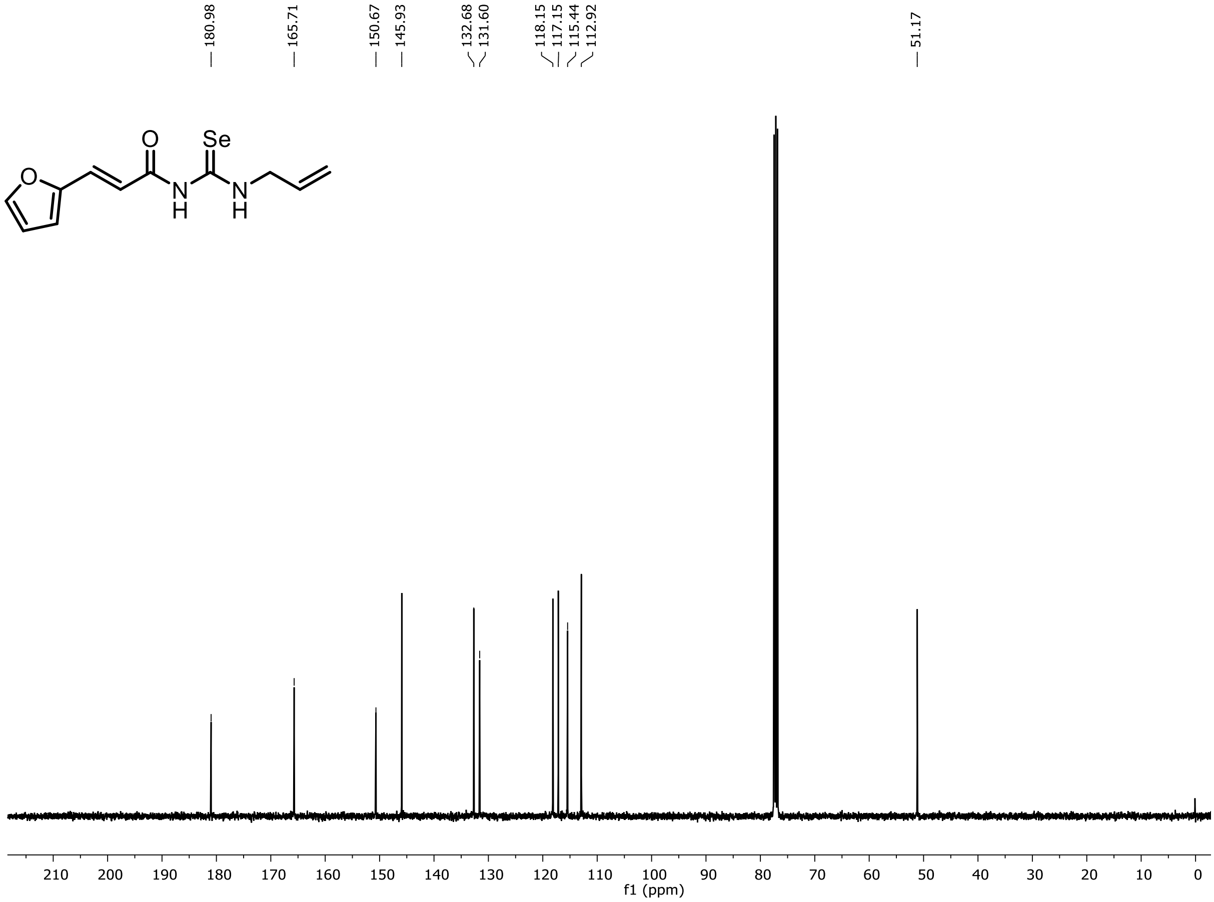

Figure S34. ^{13}C-NMR spectrum of compound **3.II**.

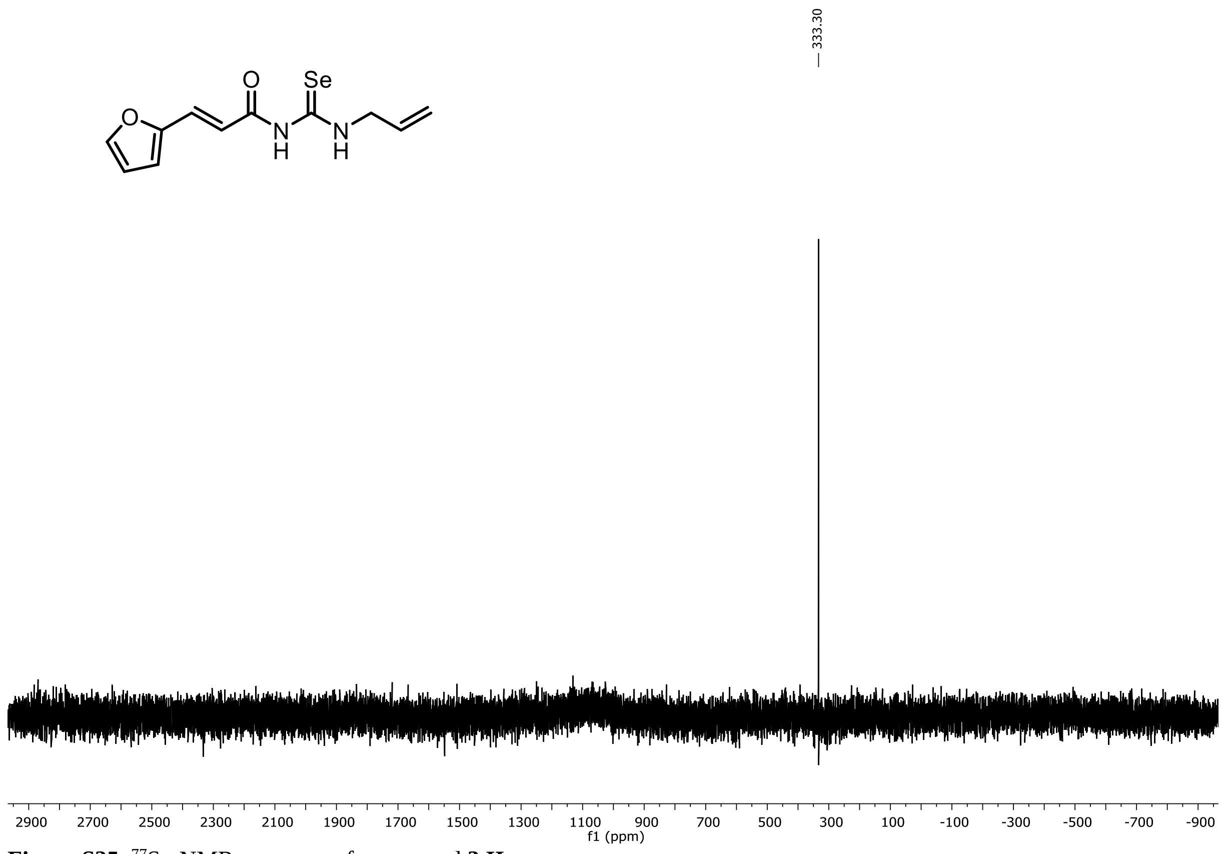

Figure S35. ^{77}Se-NMR spectrum of compound **3.II**.

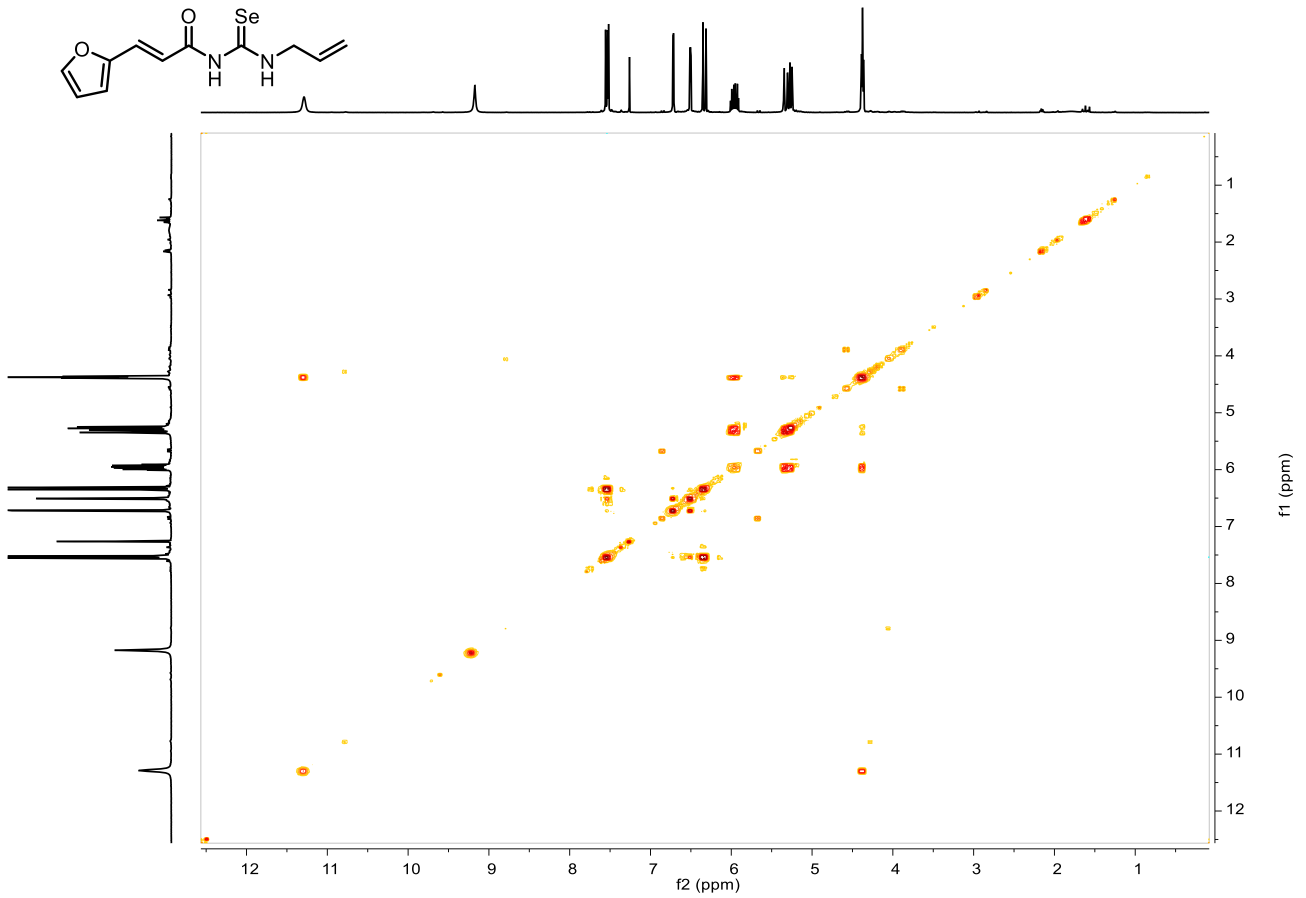

Figure S36. COSY-NMR spectrum of compound **3.II**.

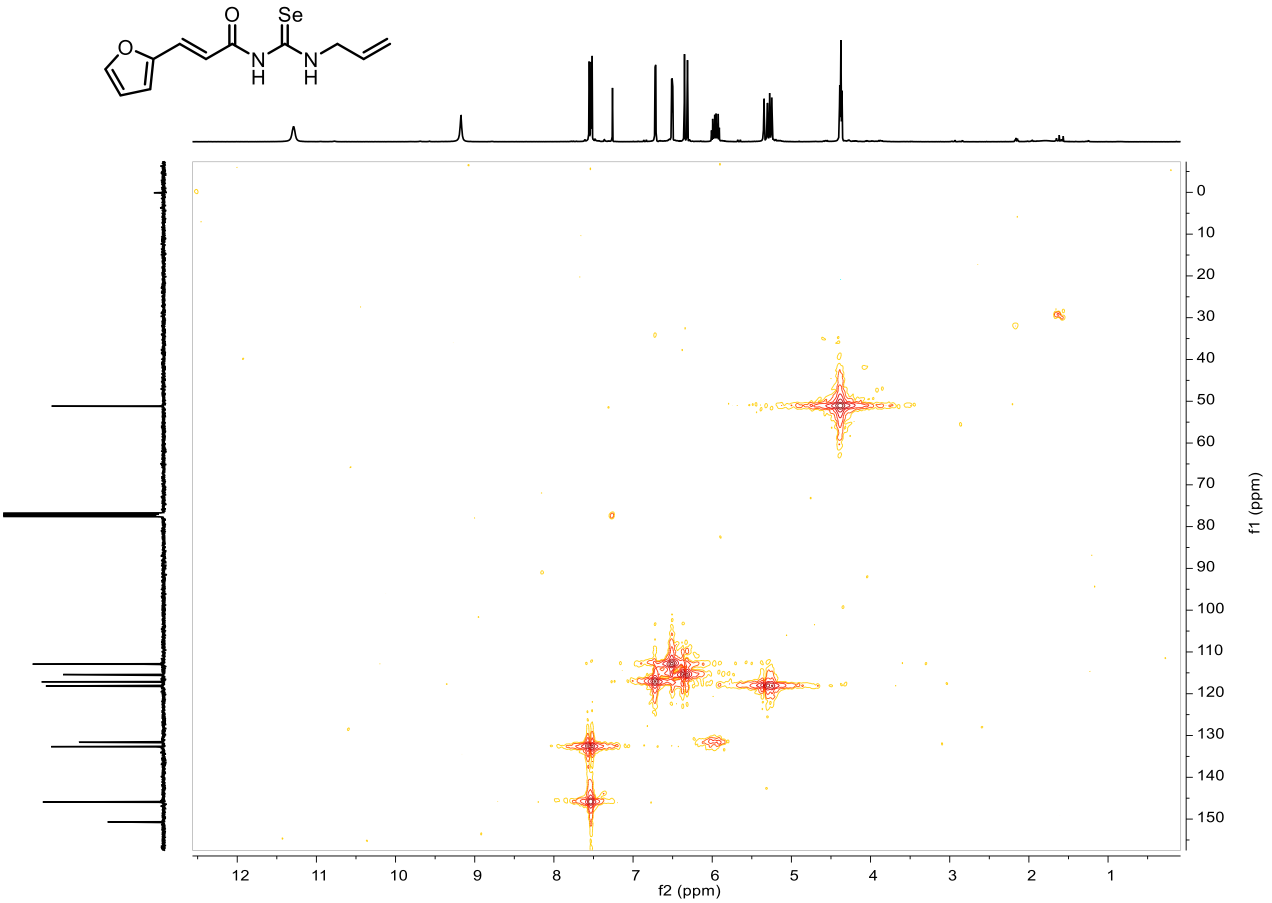

Figure S37. HMQC-NMR spectrum of compound **3.II**.

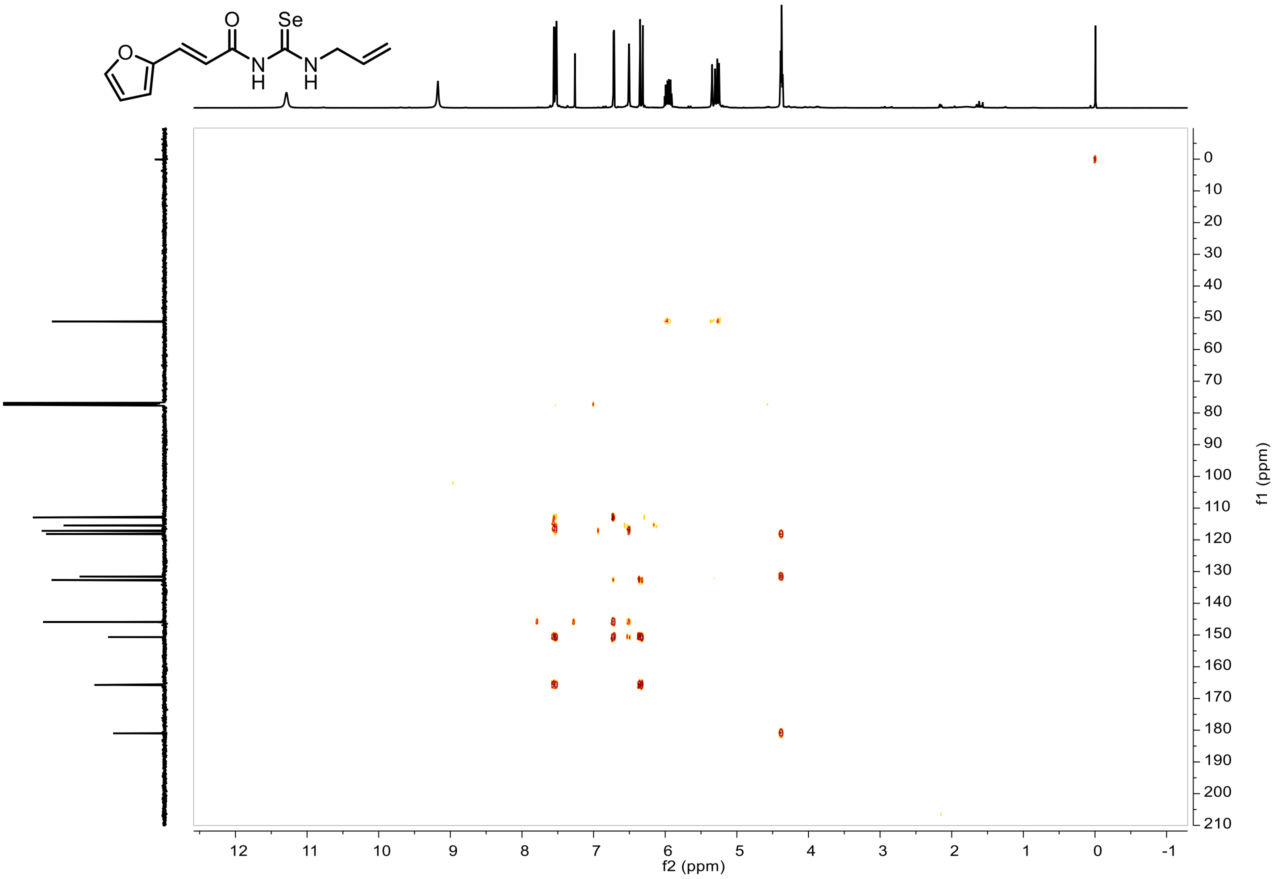

Figure S38. HMBC-NMR spectrum of compound **3.II**.

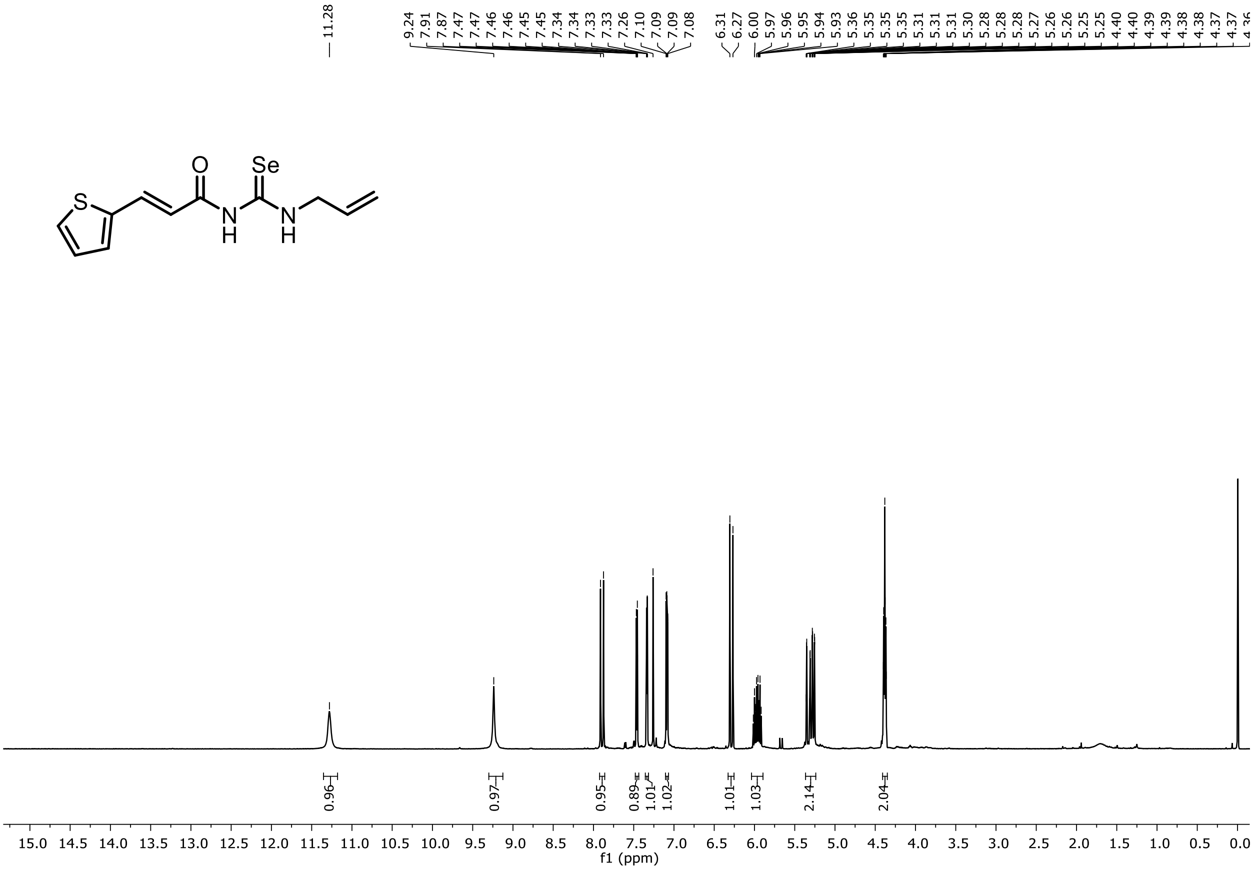

Figure S39. ^{1}H-NMR spectrum of compound **4.II**.

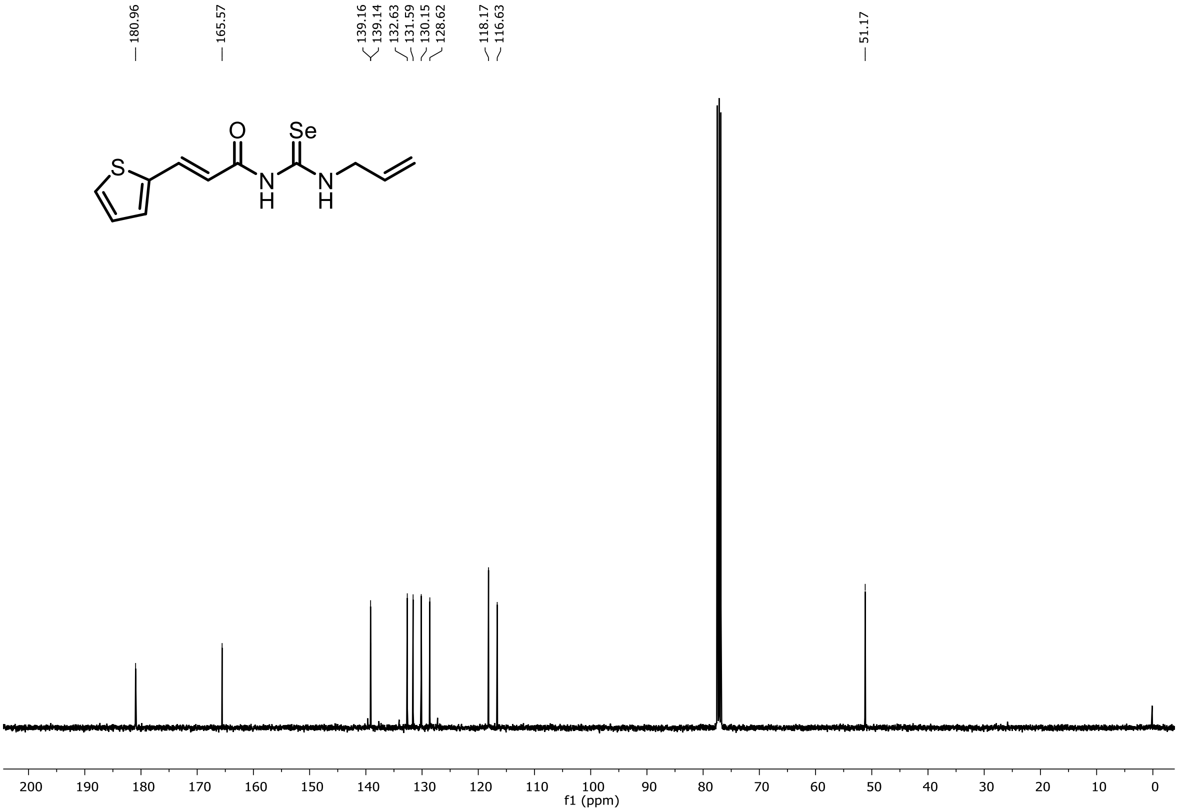

Figure S40. ^{13}C-NMR spectrum of compound **4.II**.

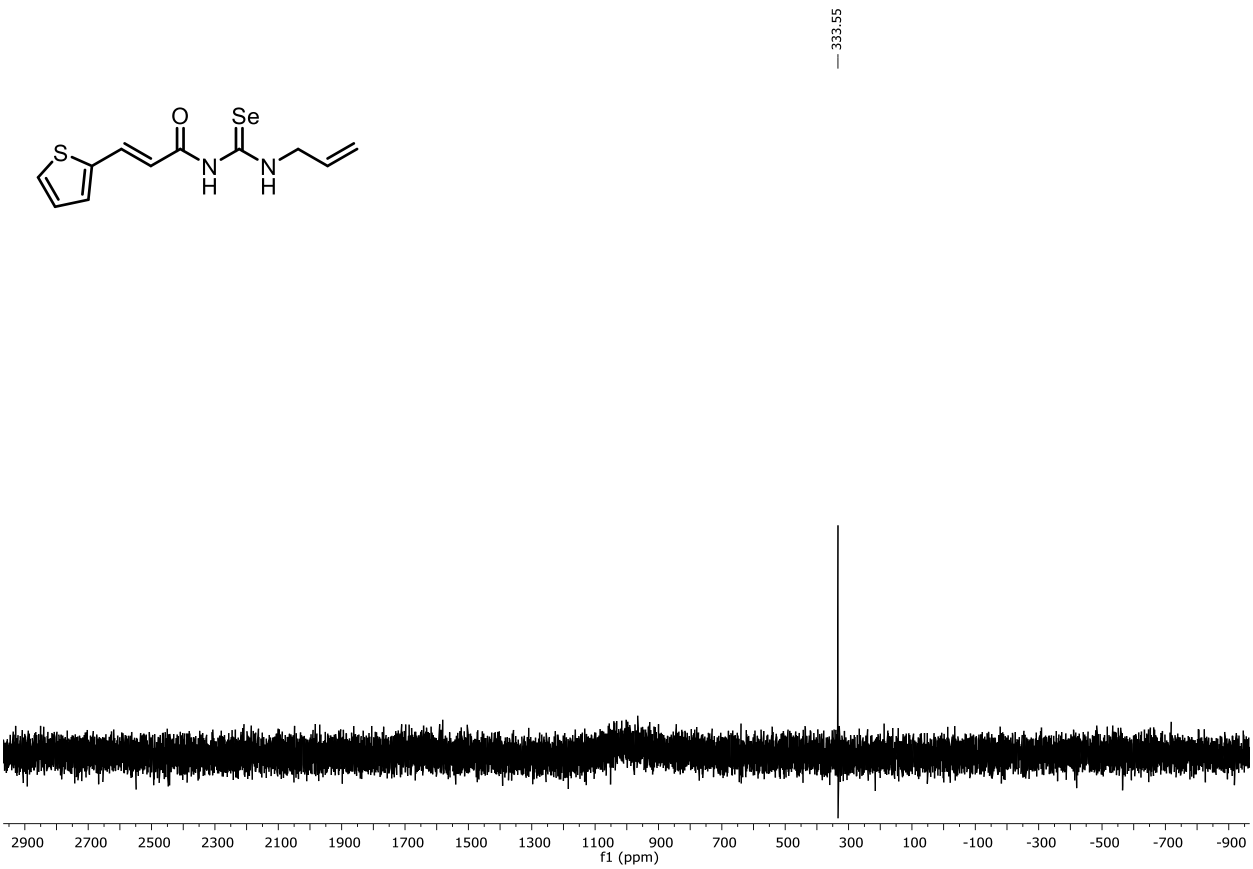

Figure S41. ^{77}Se-NMR spectrum of compound **4.II**.

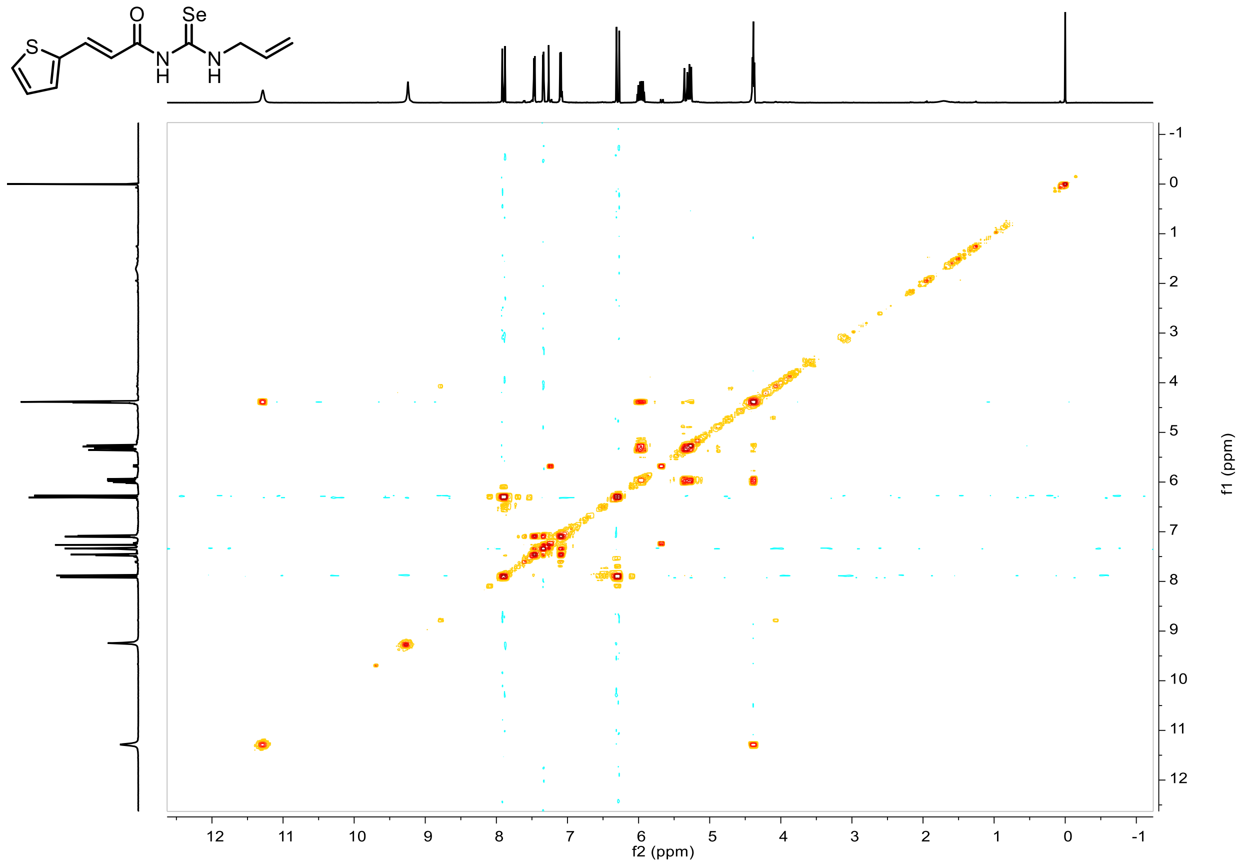

Figure S42. COSY-NMR spectrum of compound **4.II**

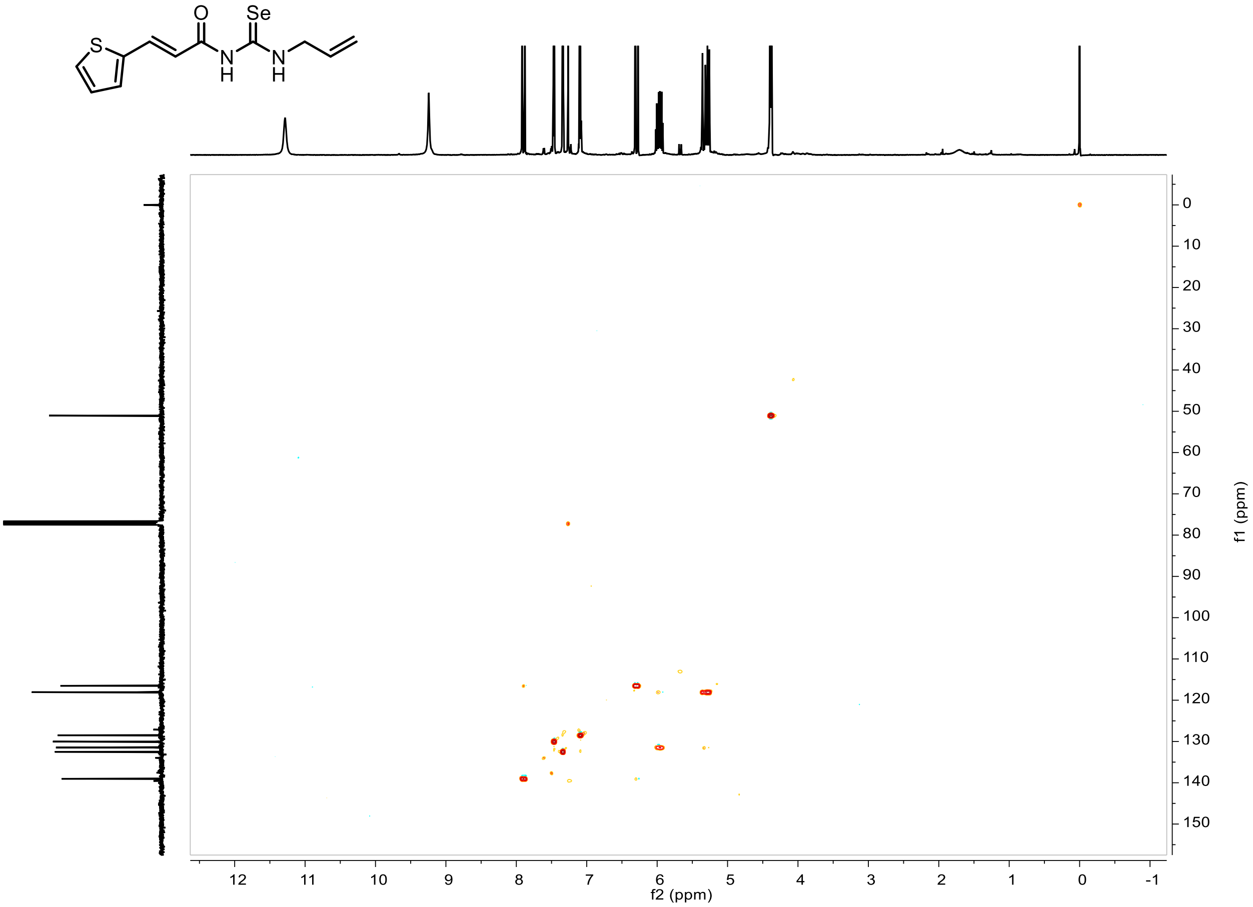

Figure S43. HSQC-NMR spectrum of compound **4.II**

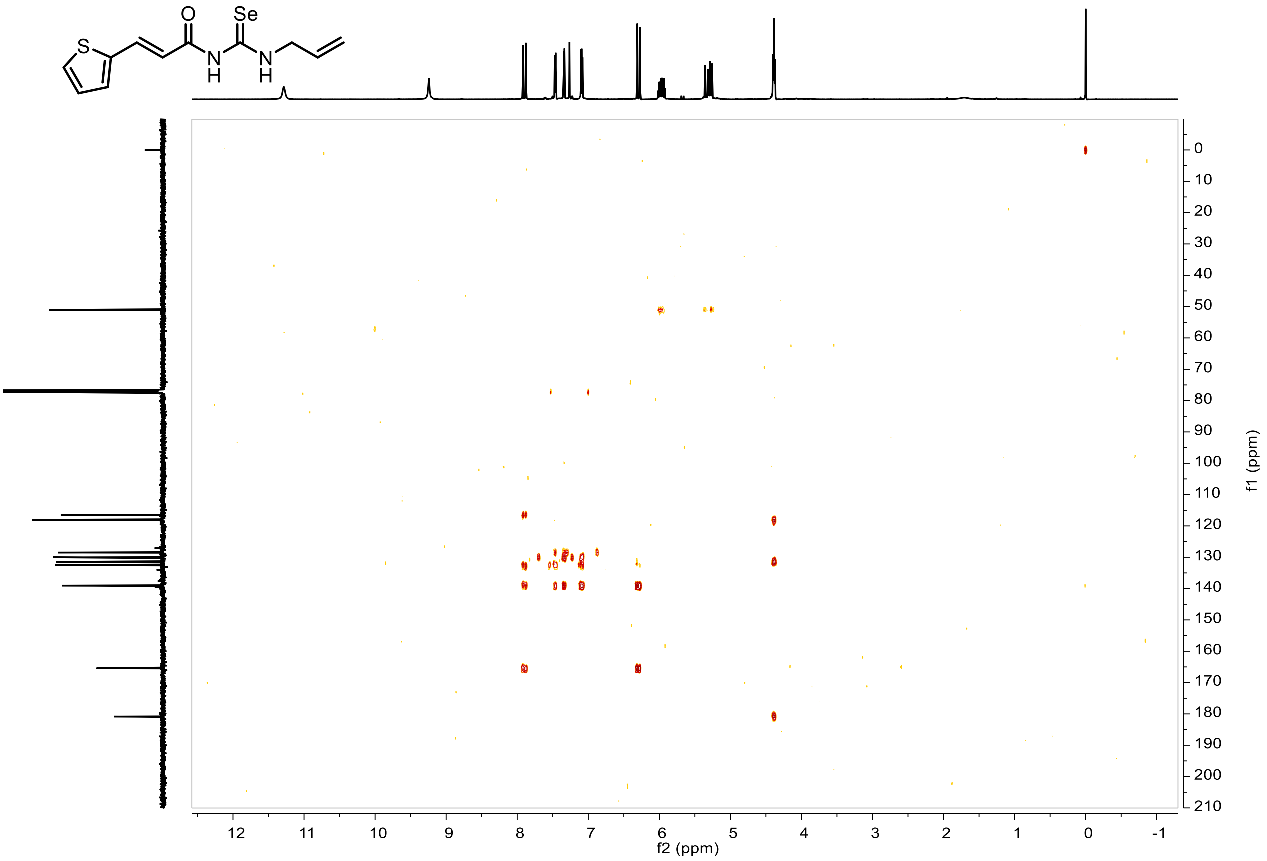

Figure S44. HMBC-NMR spectrum of compound **4.II**

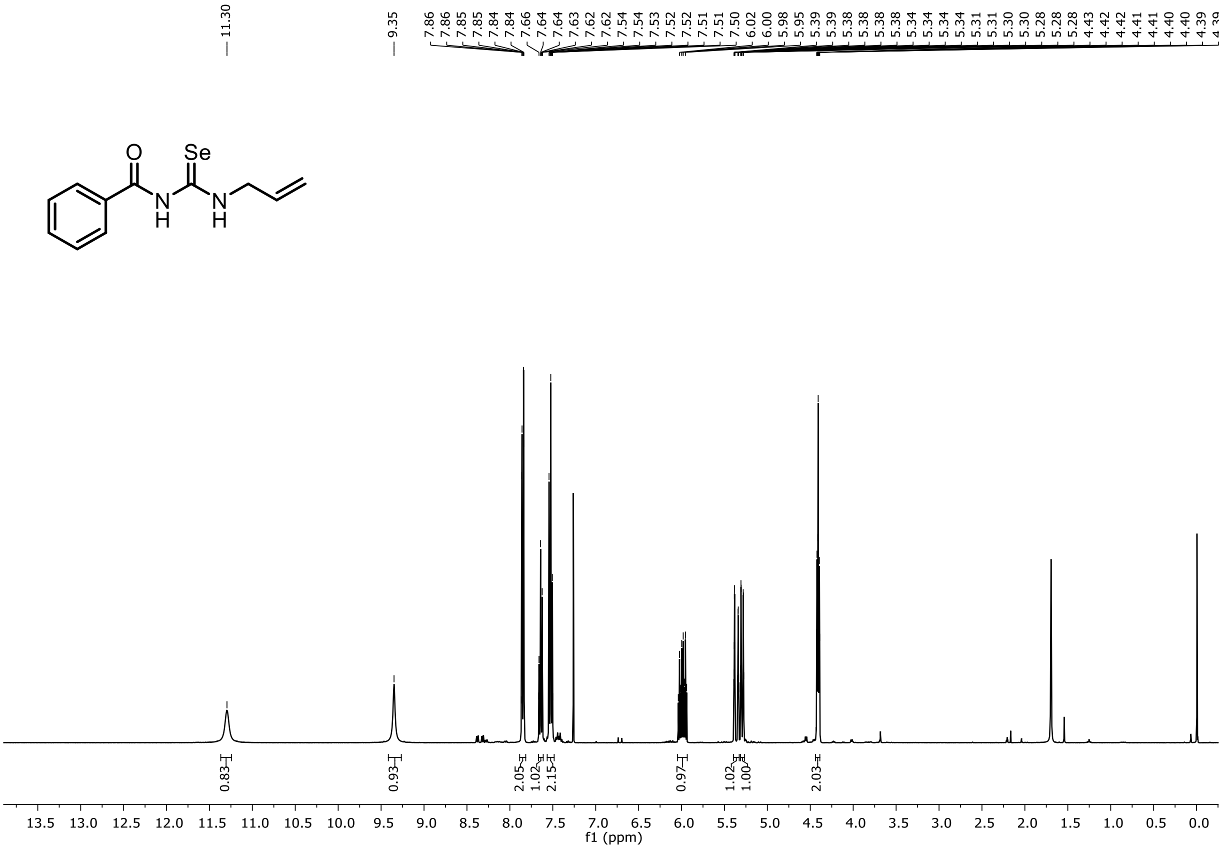

Figure S45. ^{1}H-NMR spectrum of compound **5.II**.

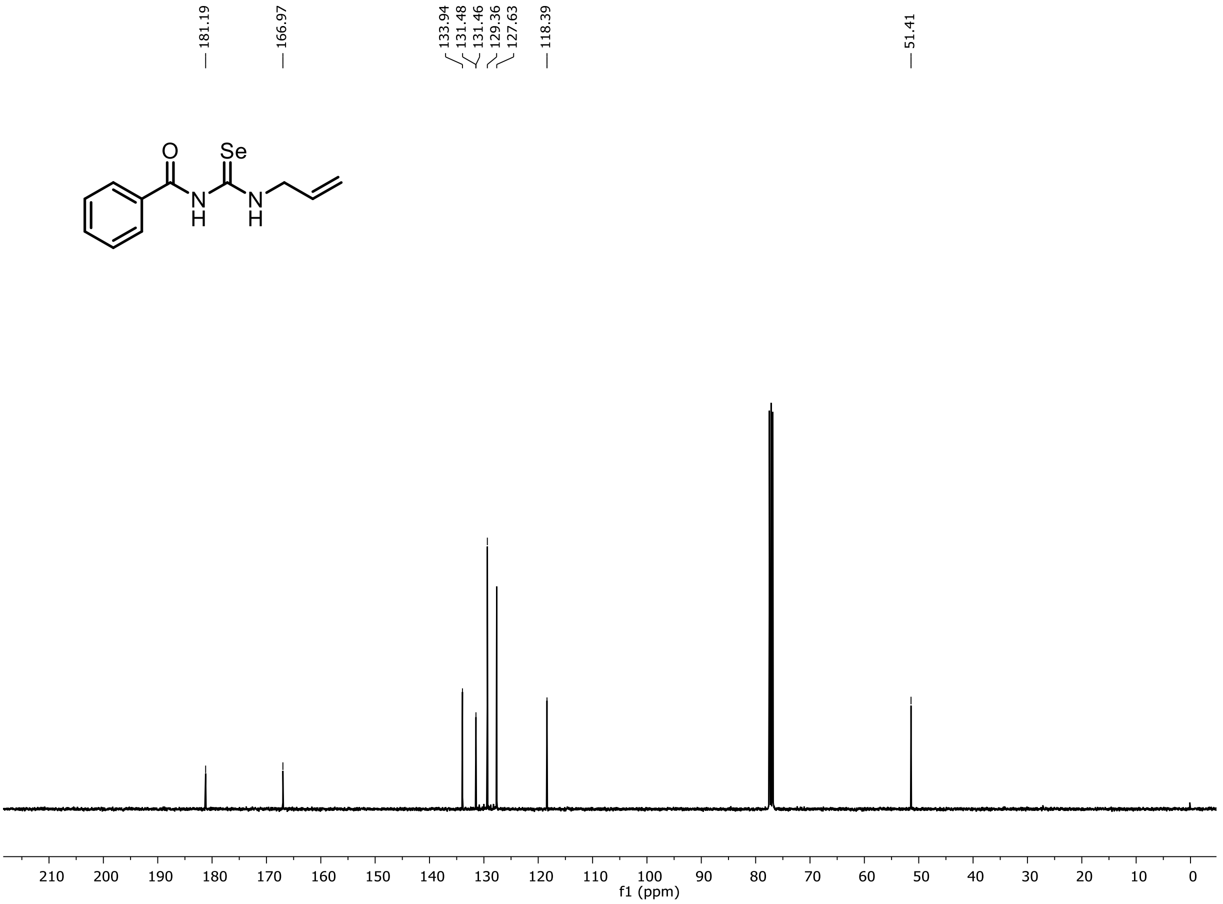

Figure S46. ^{13}C-NMR spectrum of compound **5.II.**

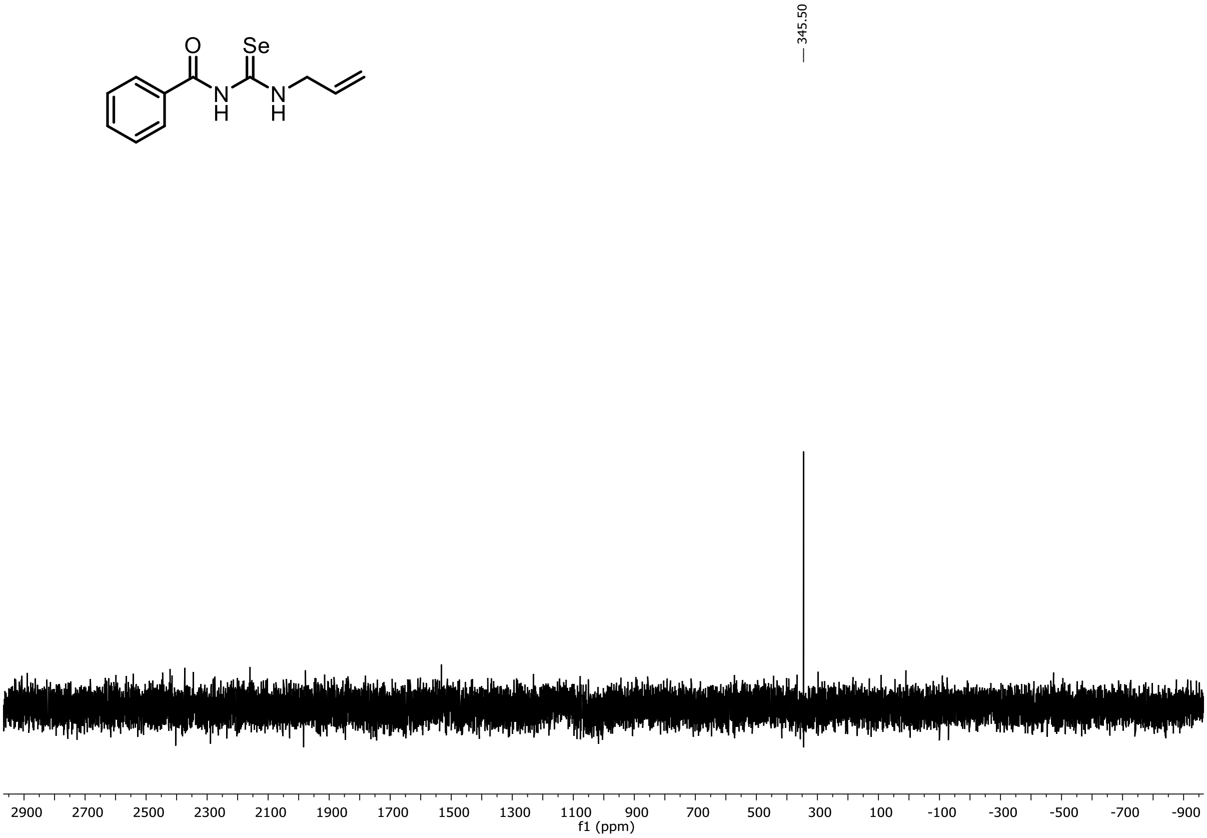

Figure S47. ^{77}Se-NMR spectrum of compound **5.II**.

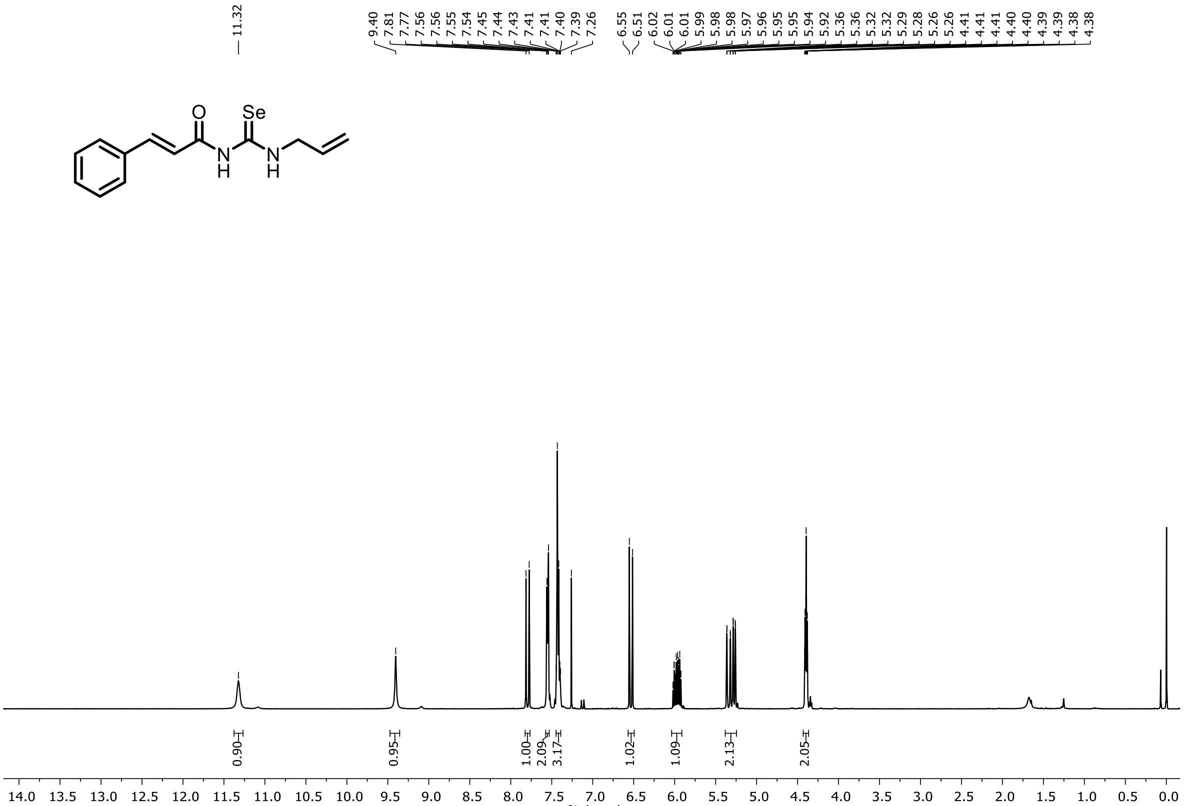

Figure S48. ^{1}H-NMR spectrum of compound **6.II**.

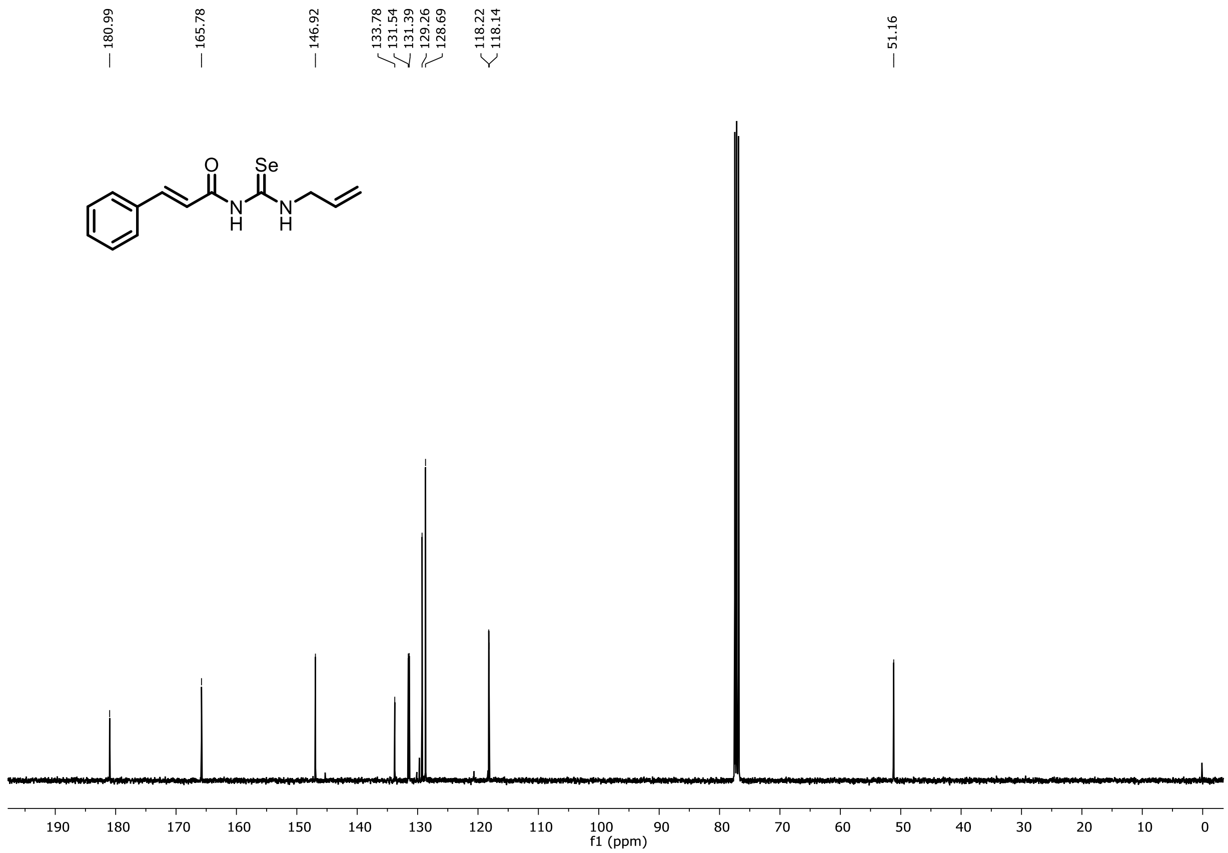

Figure S49. ^{13}C-NMR spectrum of compound **6.II**.

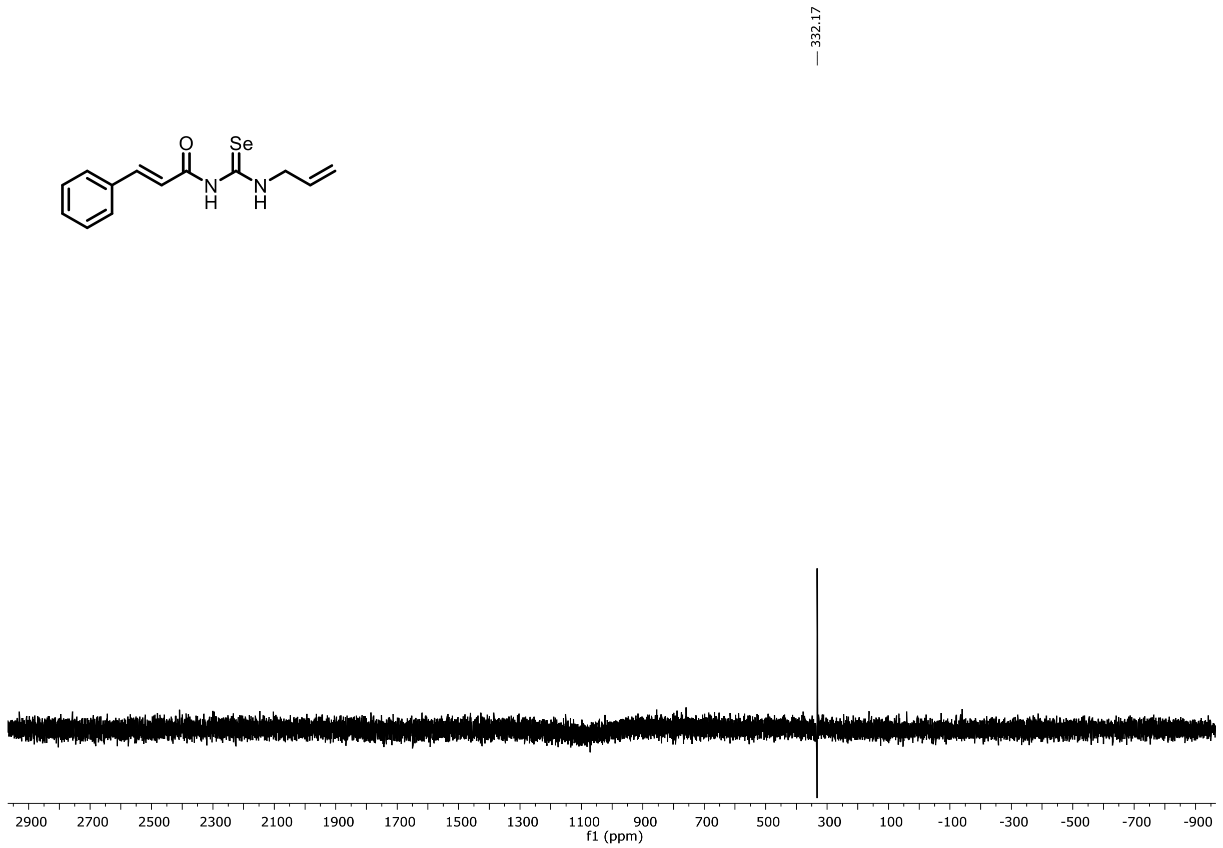

Figure S50. ^{77}Se-NMR spectrum of compound **6.II**.

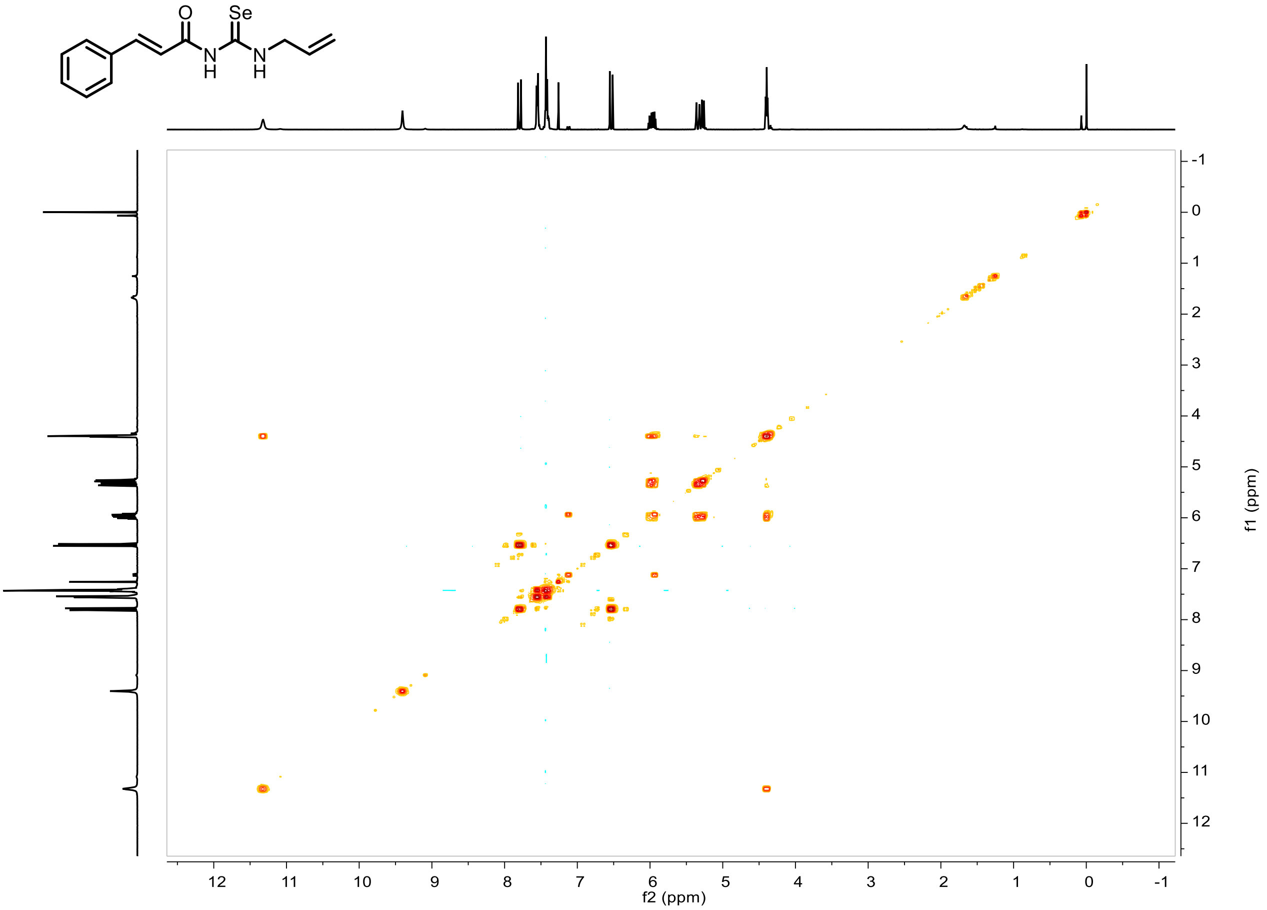

Figure S51. COSY-NMR spectrum of compound **6.II**.

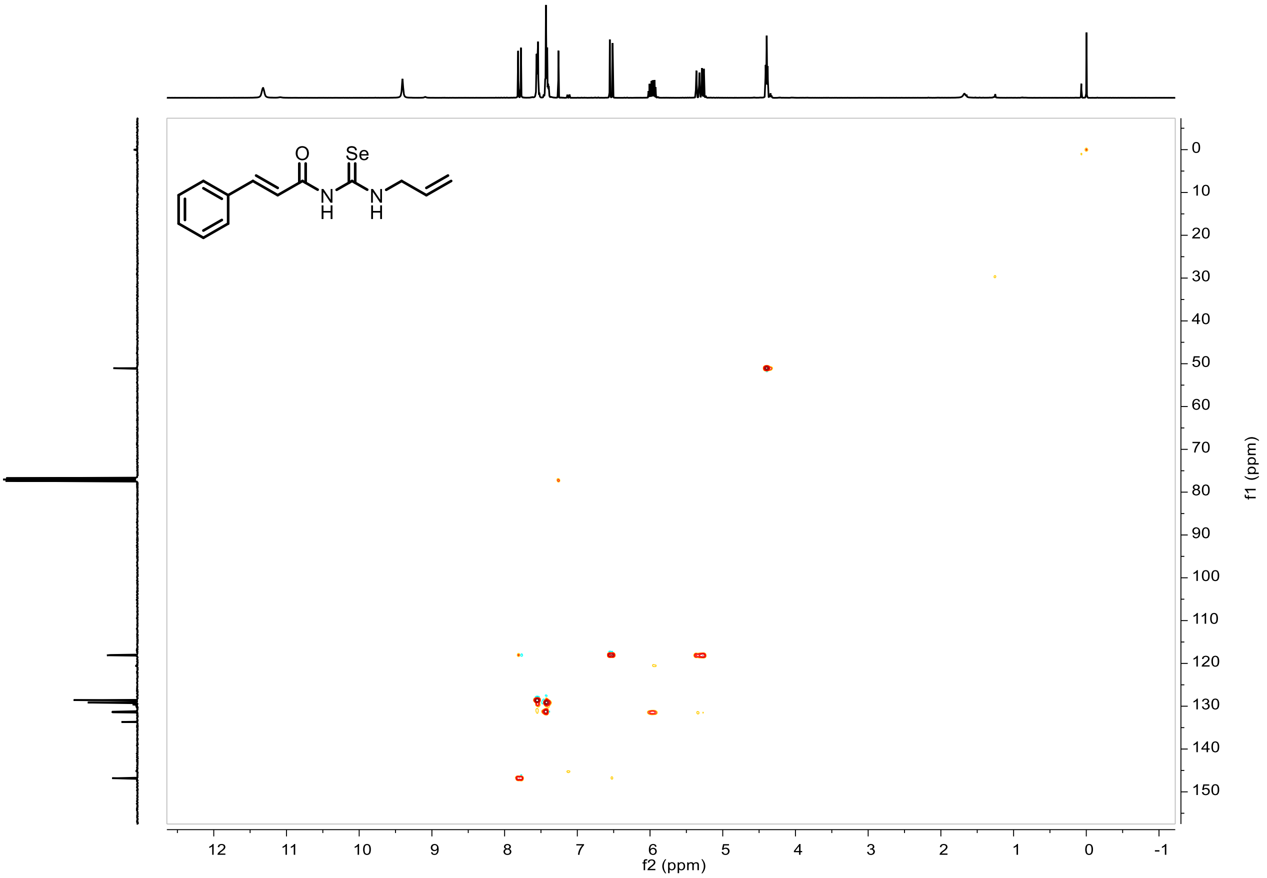

Figure S52. HSQC-NMR spectrum of compound **6.II**.

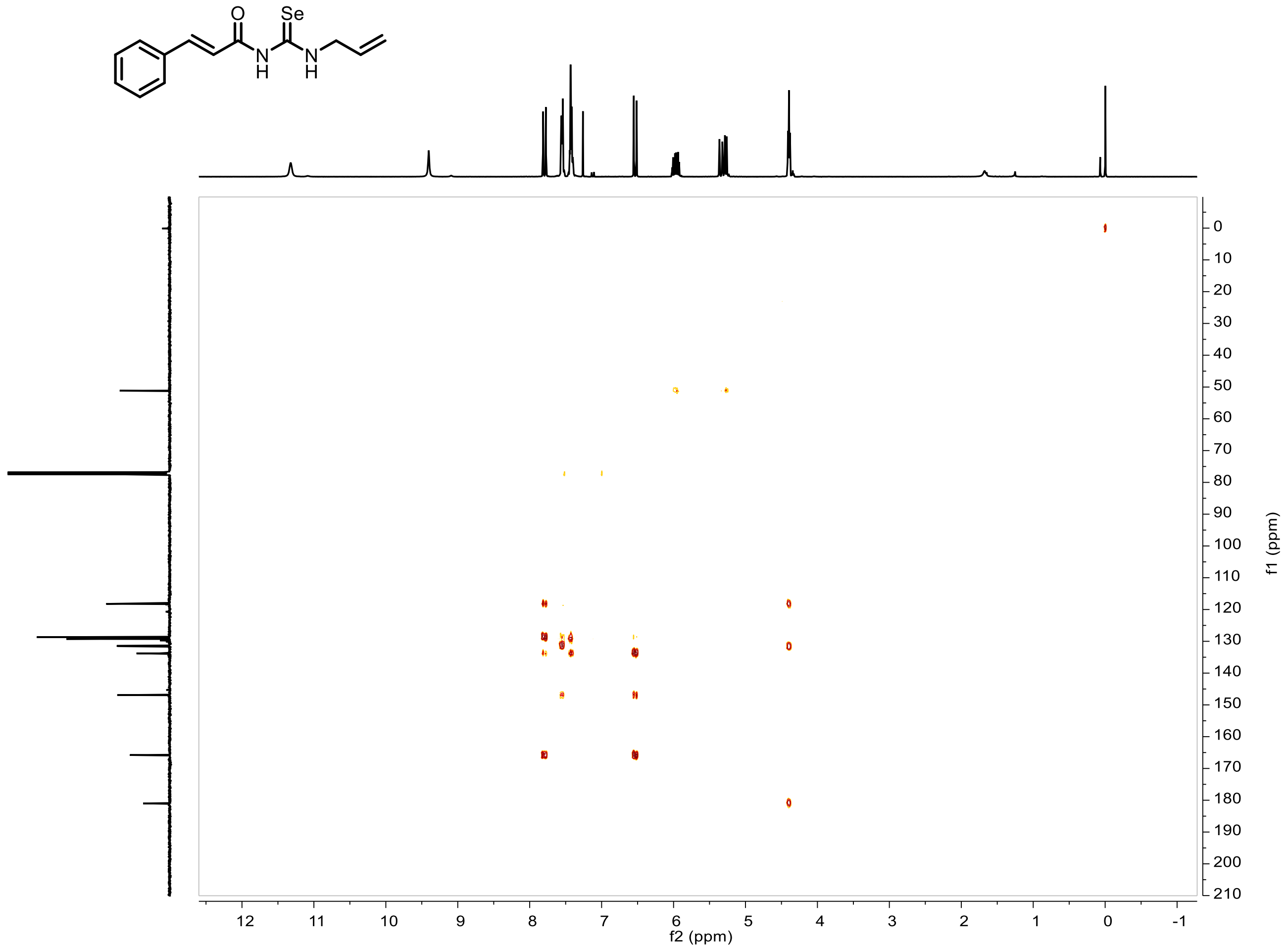

Figure S53. HMBC-NMR spectrum of compound **6.II**.

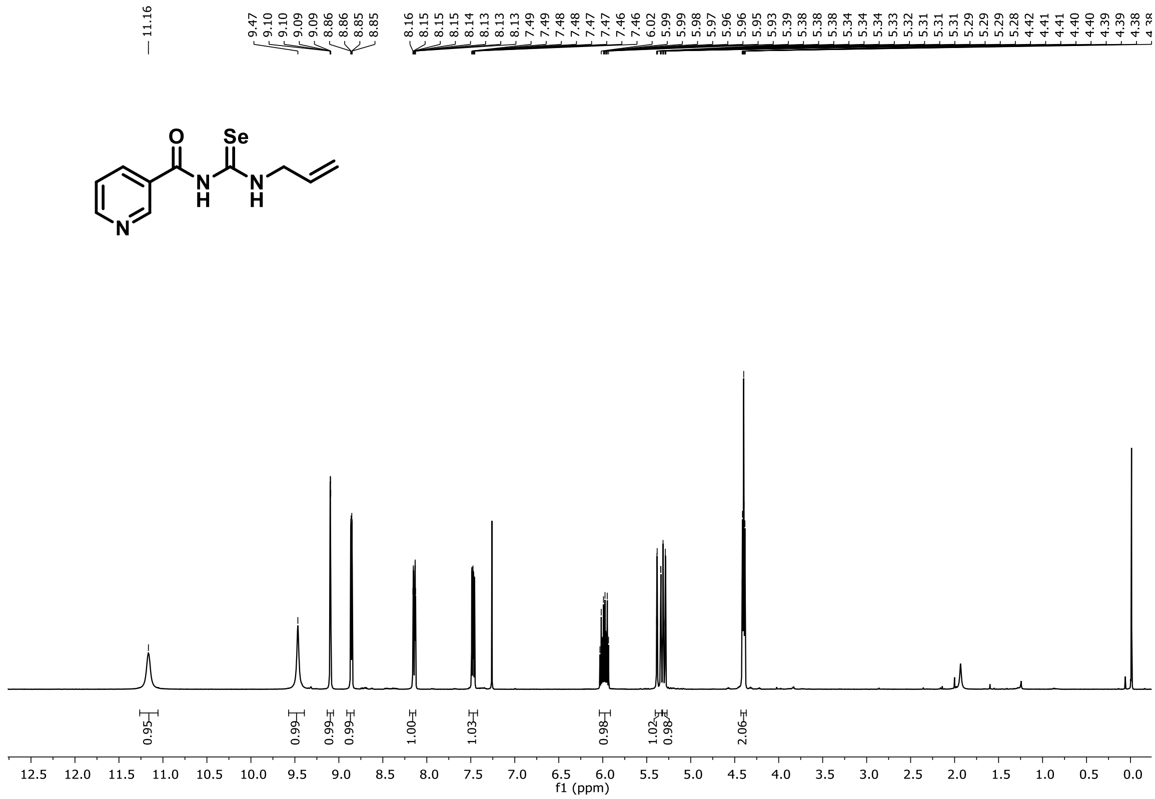

Figure S54. ^{1}H-NMR spectrum of compound **7.II**.

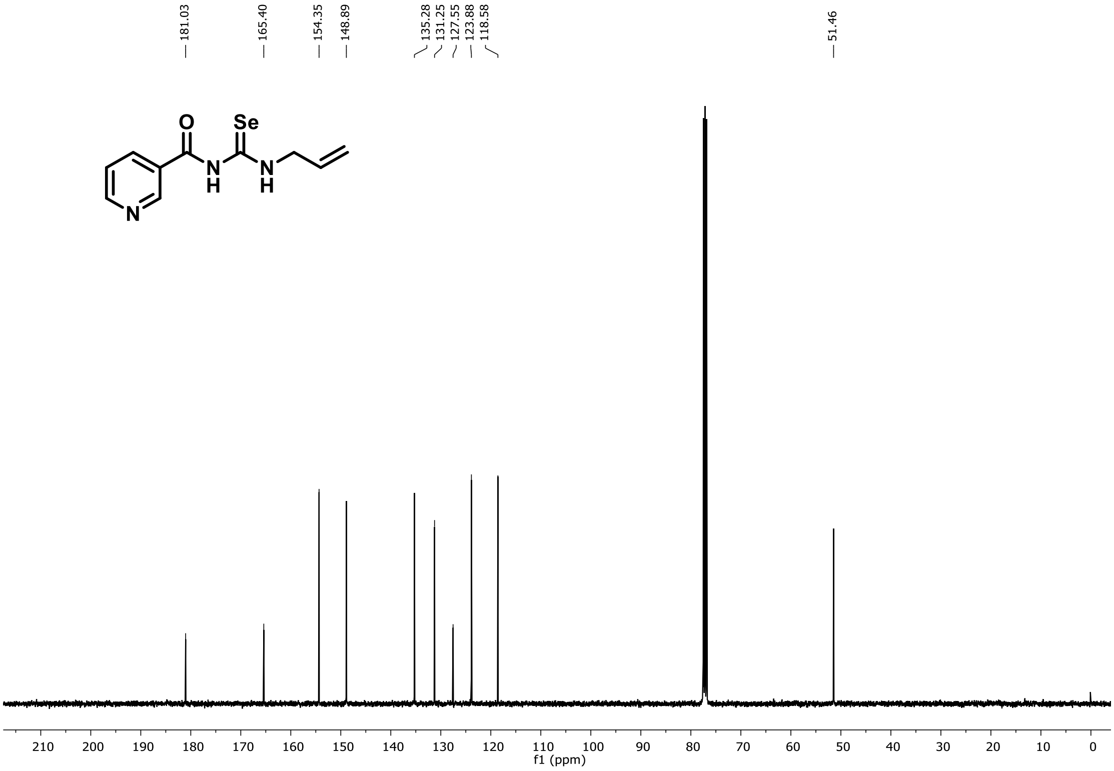

Figure S55. ^{13}C-NMR spectrum of compound **7.II**.

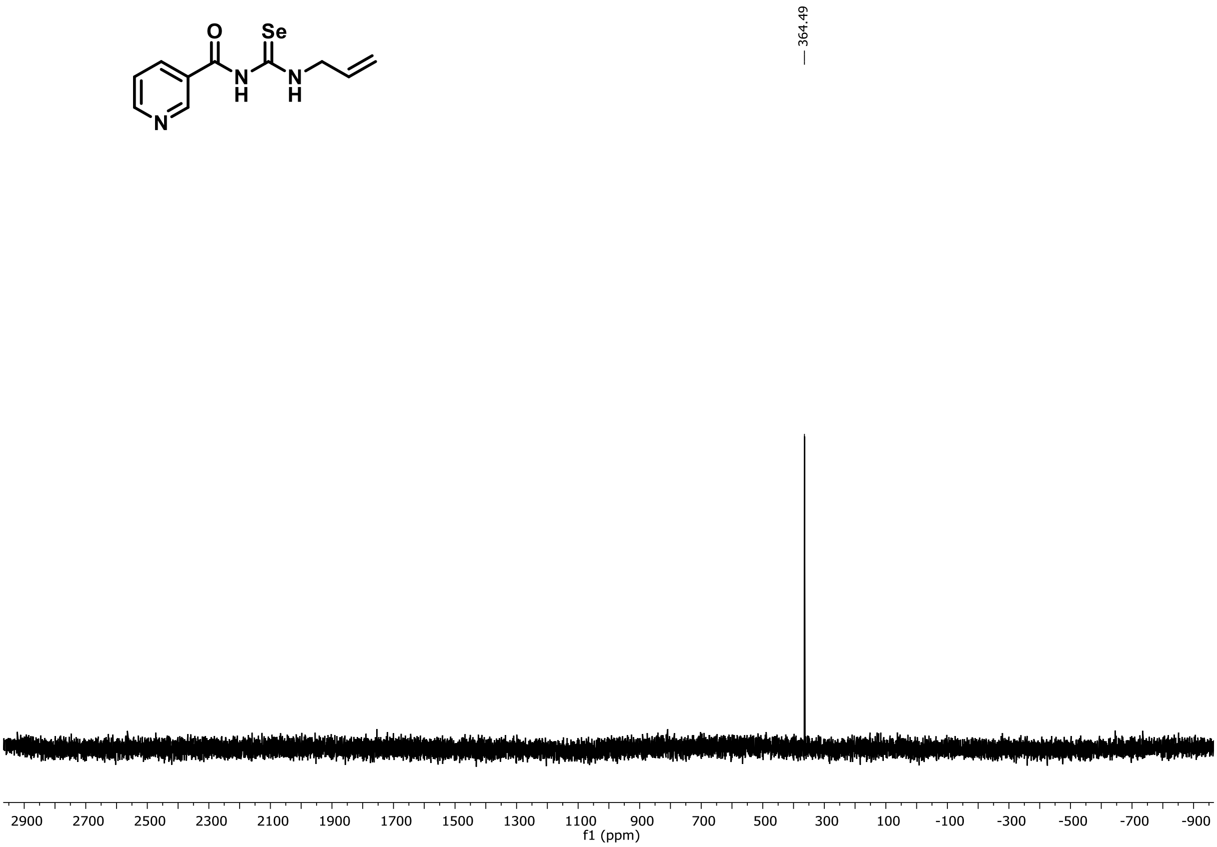

Figure S56. ^{77}Se-NMR spectrum of compound **7.II**.

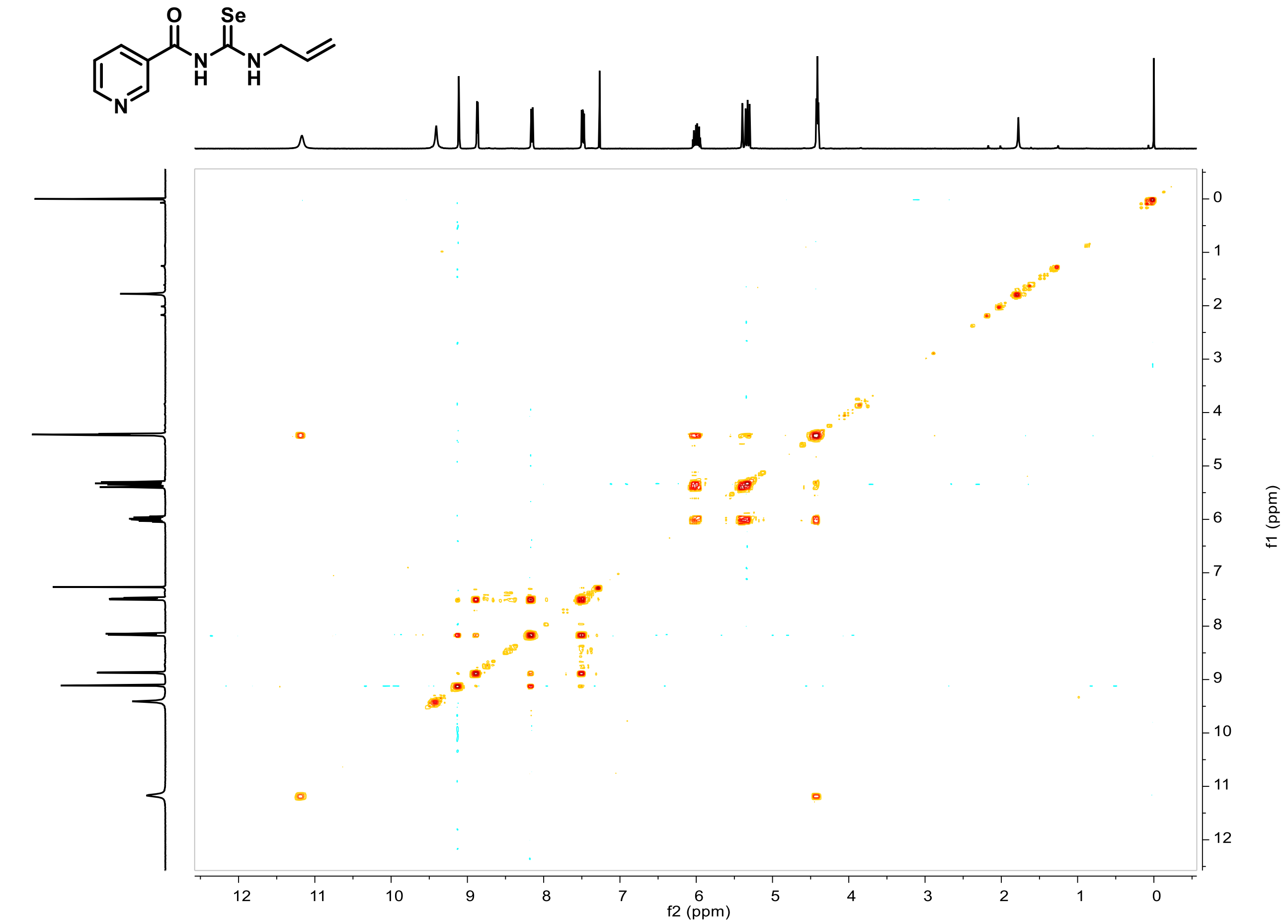

Figure S57. COSY-NMR spectrum of compound **7.II**.

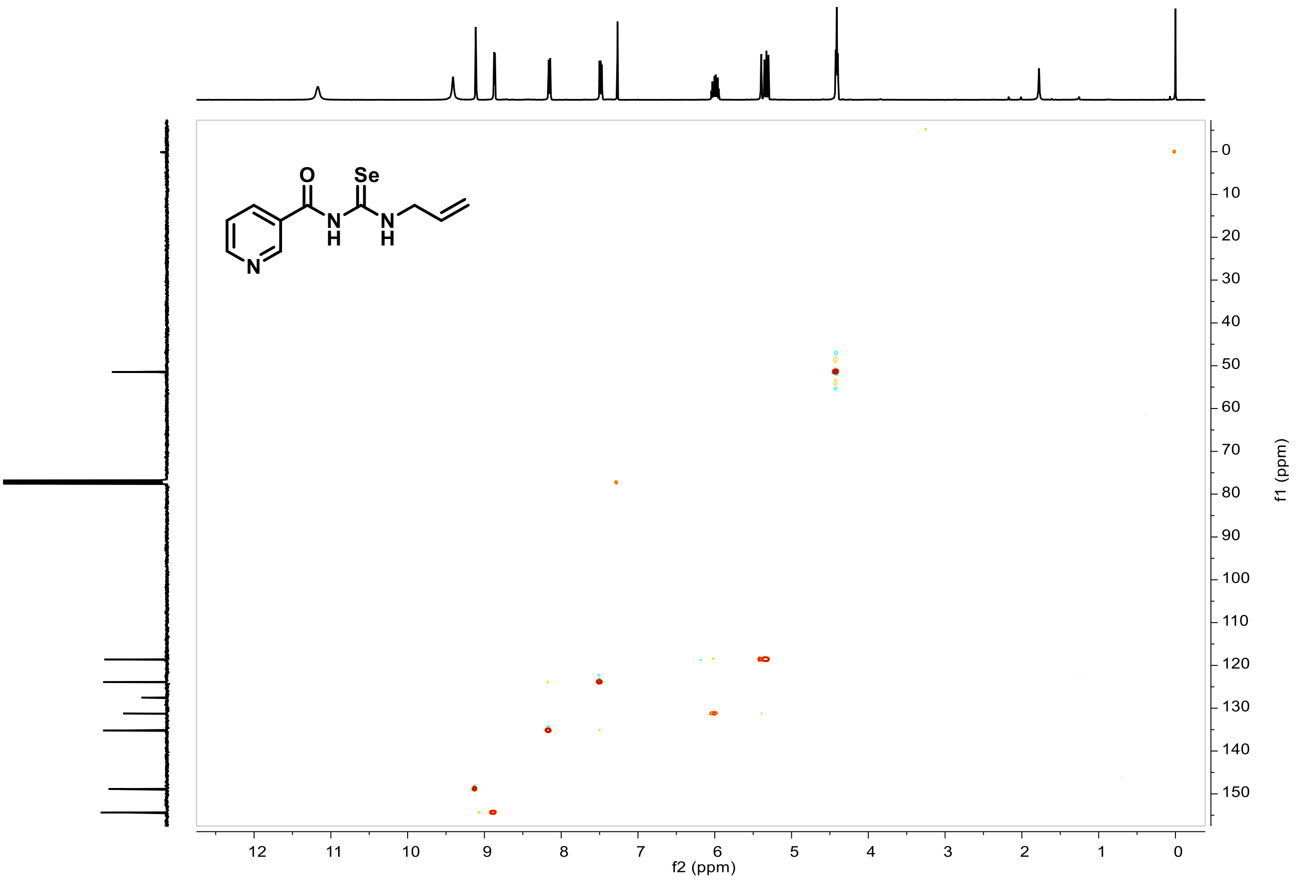

Figure S58. HSQC-NMR spectrum of compound **7.II**.

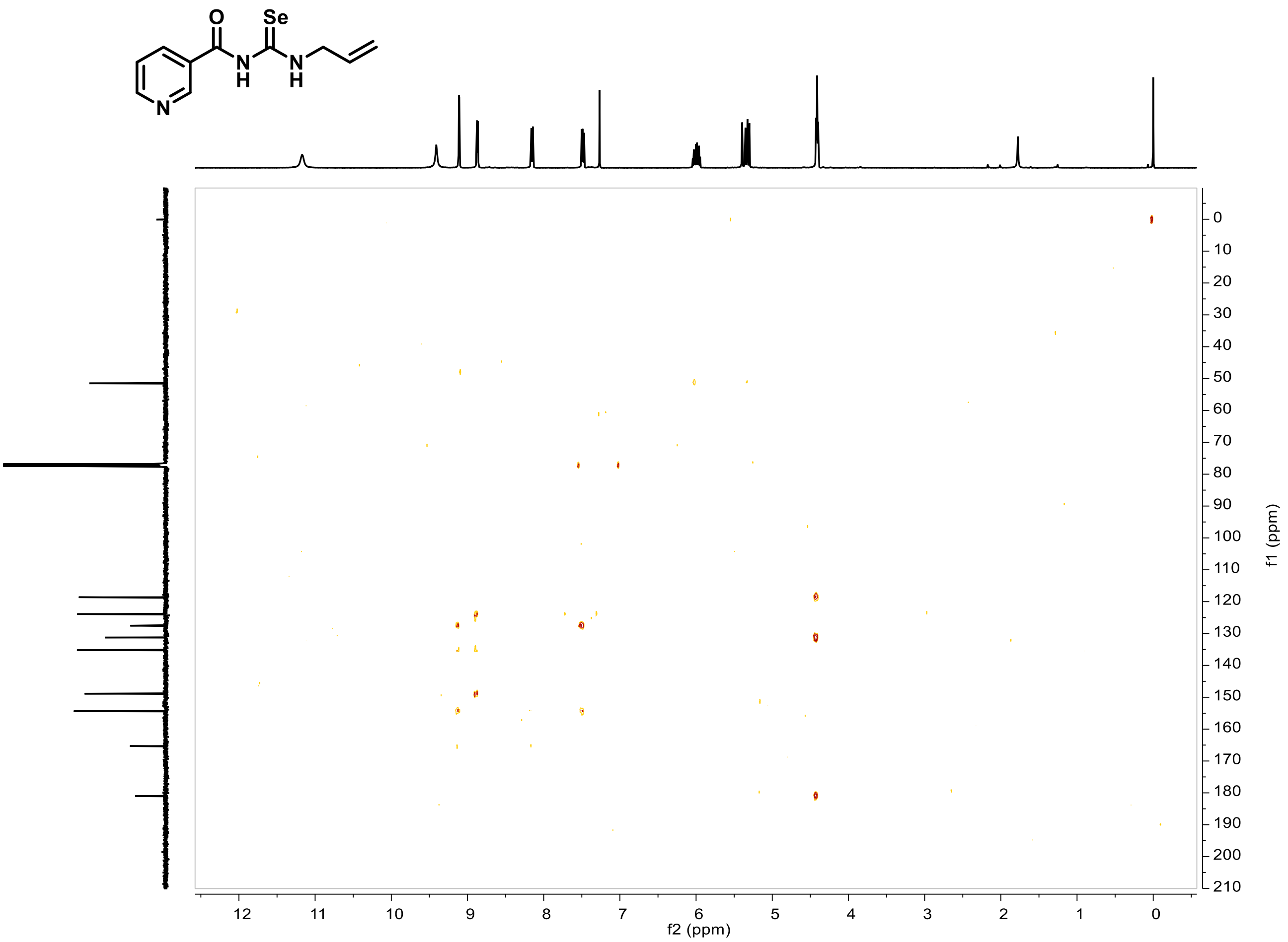

Figure S59. HMBC-NMR spectrum of compound **7.II**.

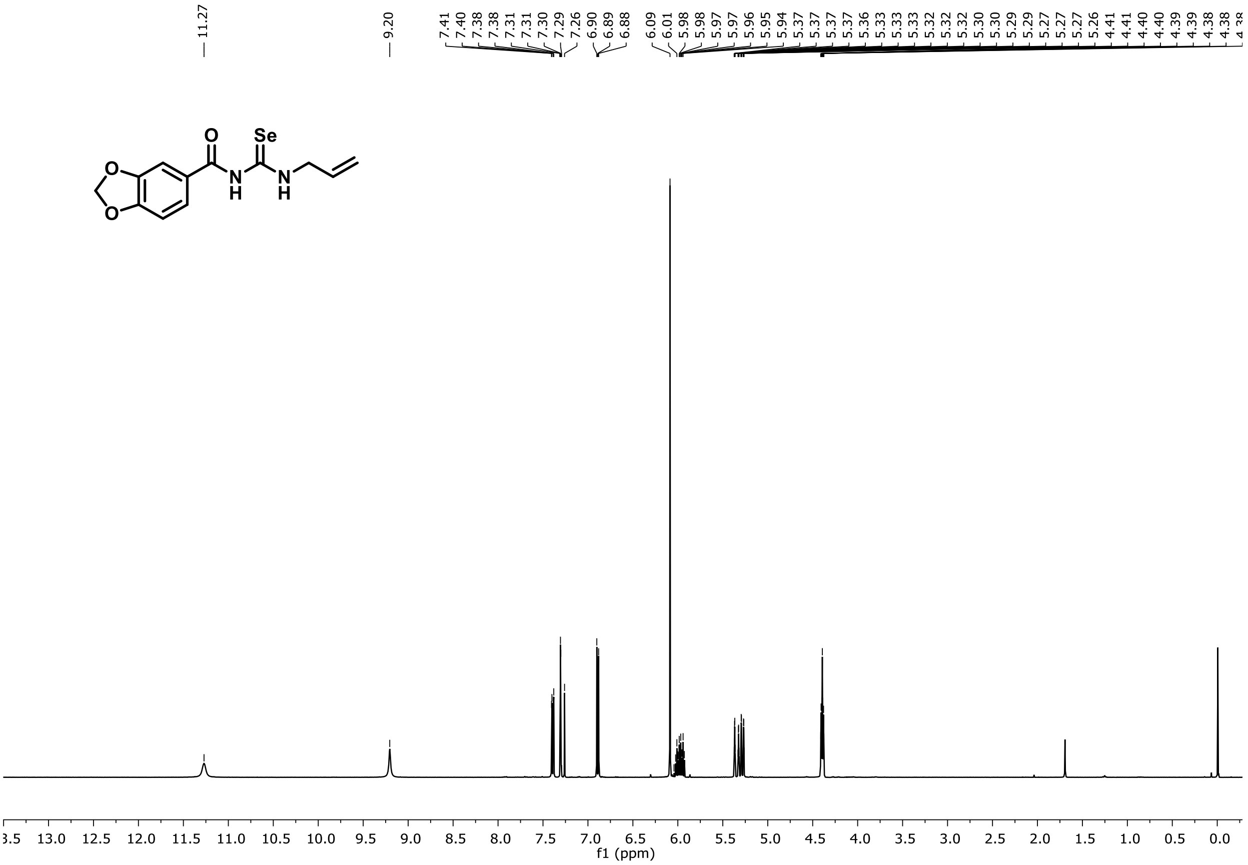

Figure S60. ^{1}H-NMR spectrum of compound **8.II**.

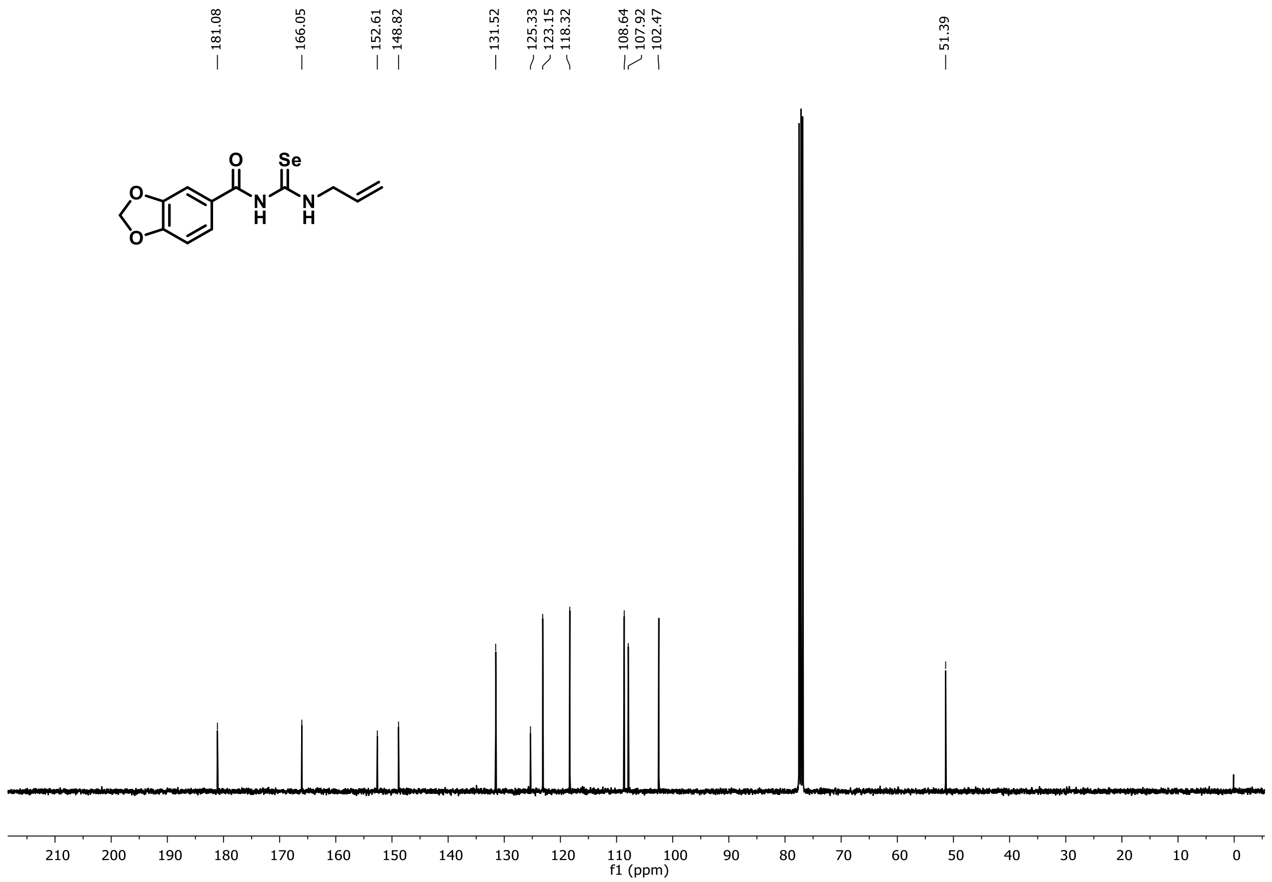

Figure S61. ^{13}C-NMR spectrum of compound **8.II**.

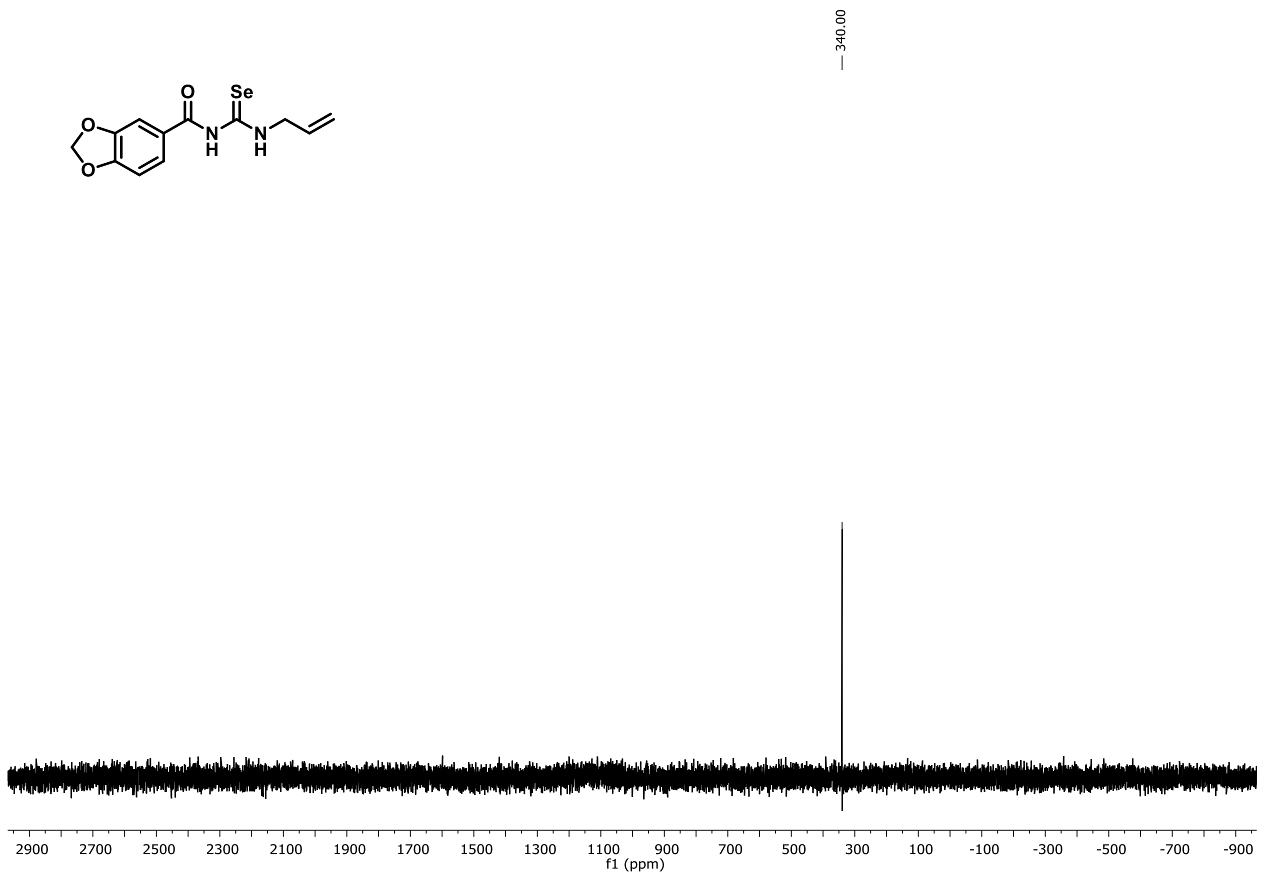

Figure S62. ^{77}Se-NMR spectrum of compound **8.II**.

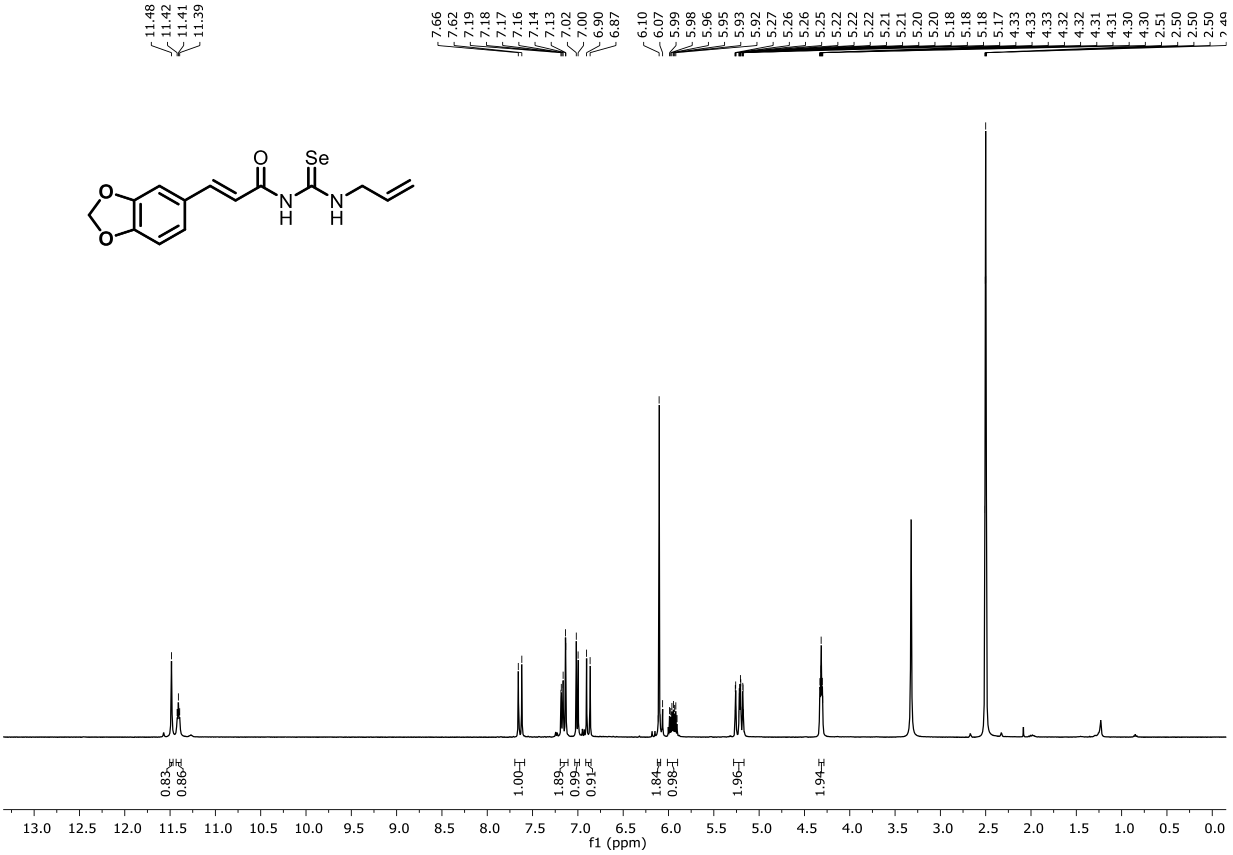

Figure S63. ^{1}H-NMR spectrum of compound **9.II**.

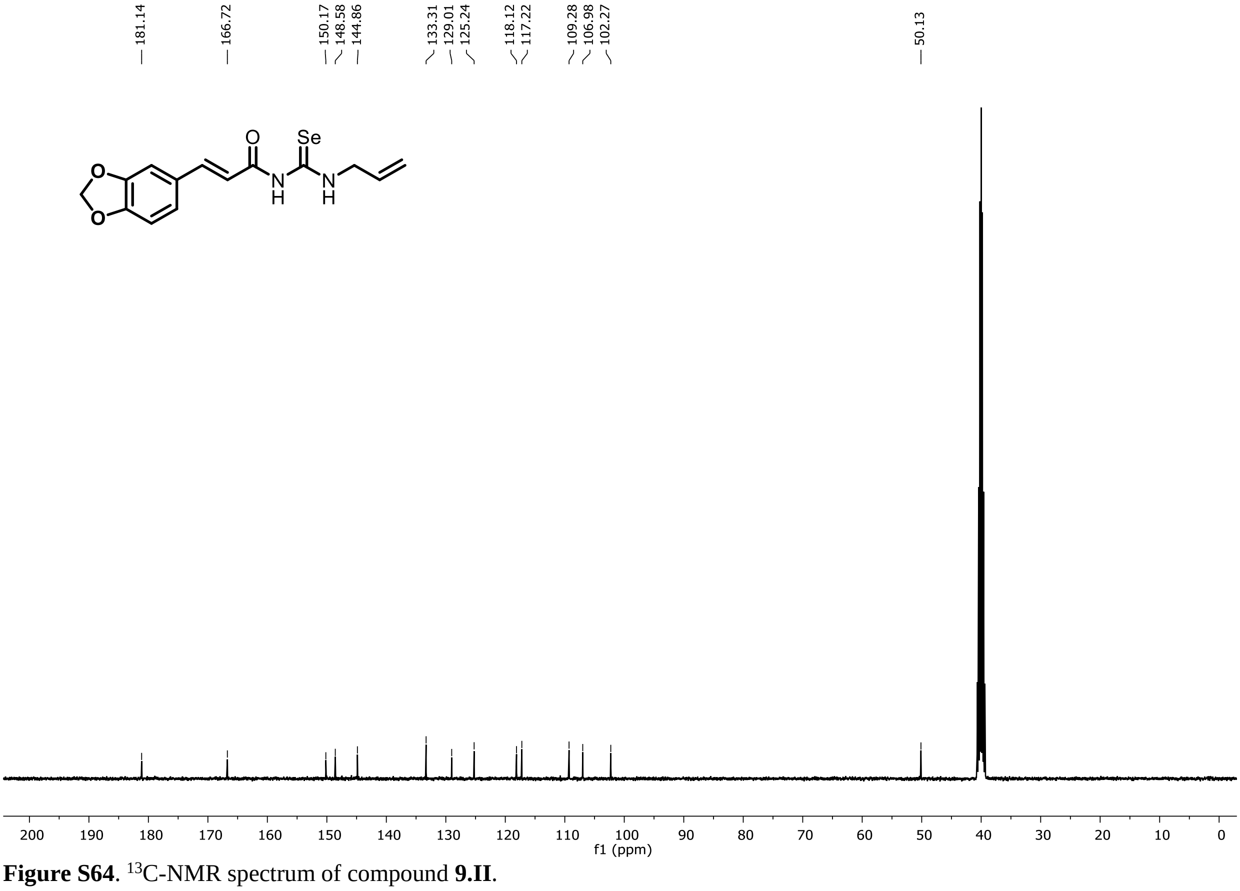

Figure S64. ^{13}C-NMR spectrum of compound **9.II**.

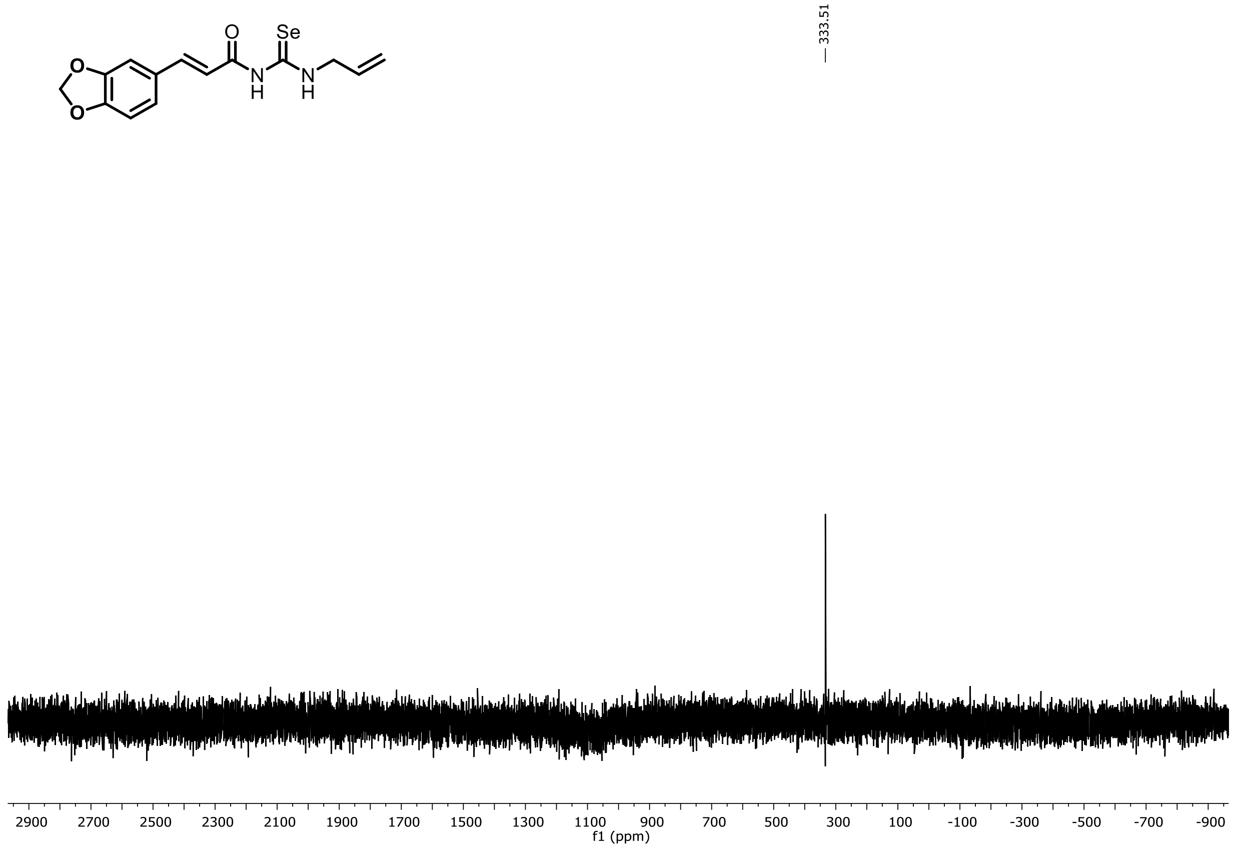

Figure S65. ^{77}Se-NMR spectrum of compound **9.II**.

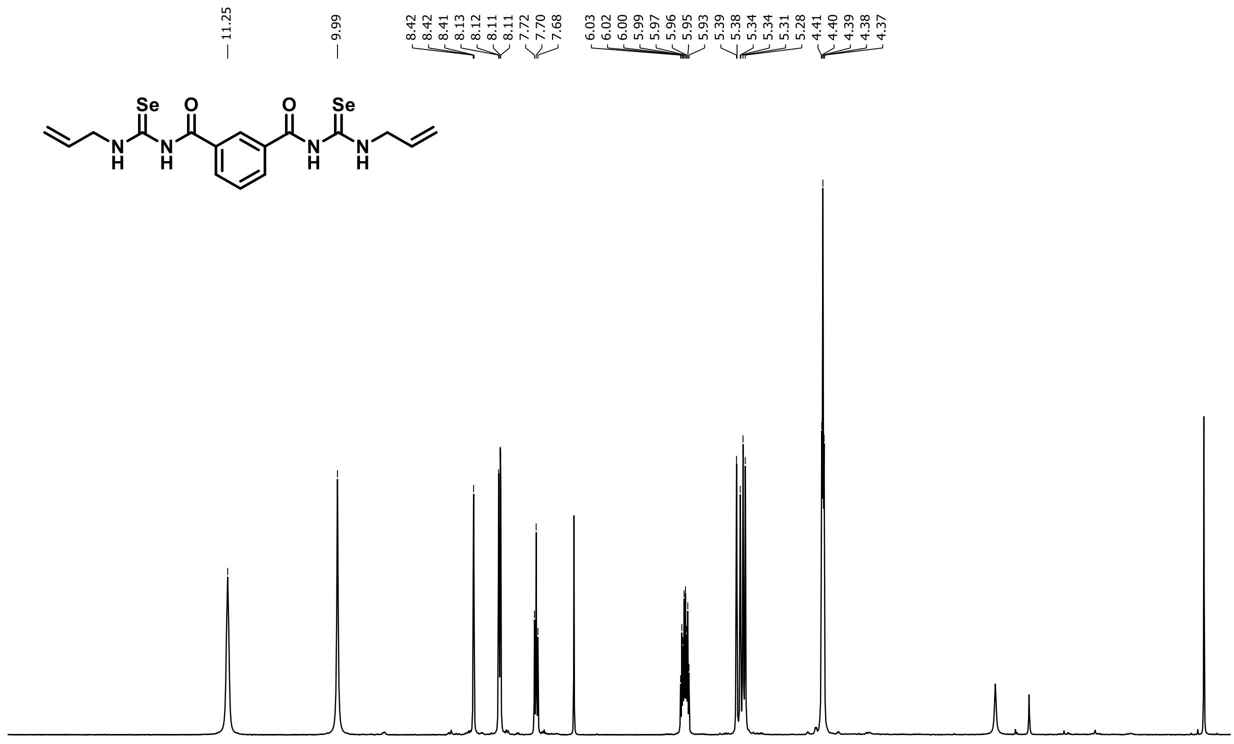

Figure S66. ^{1}H-NMR spectrum of compound **10.II**.

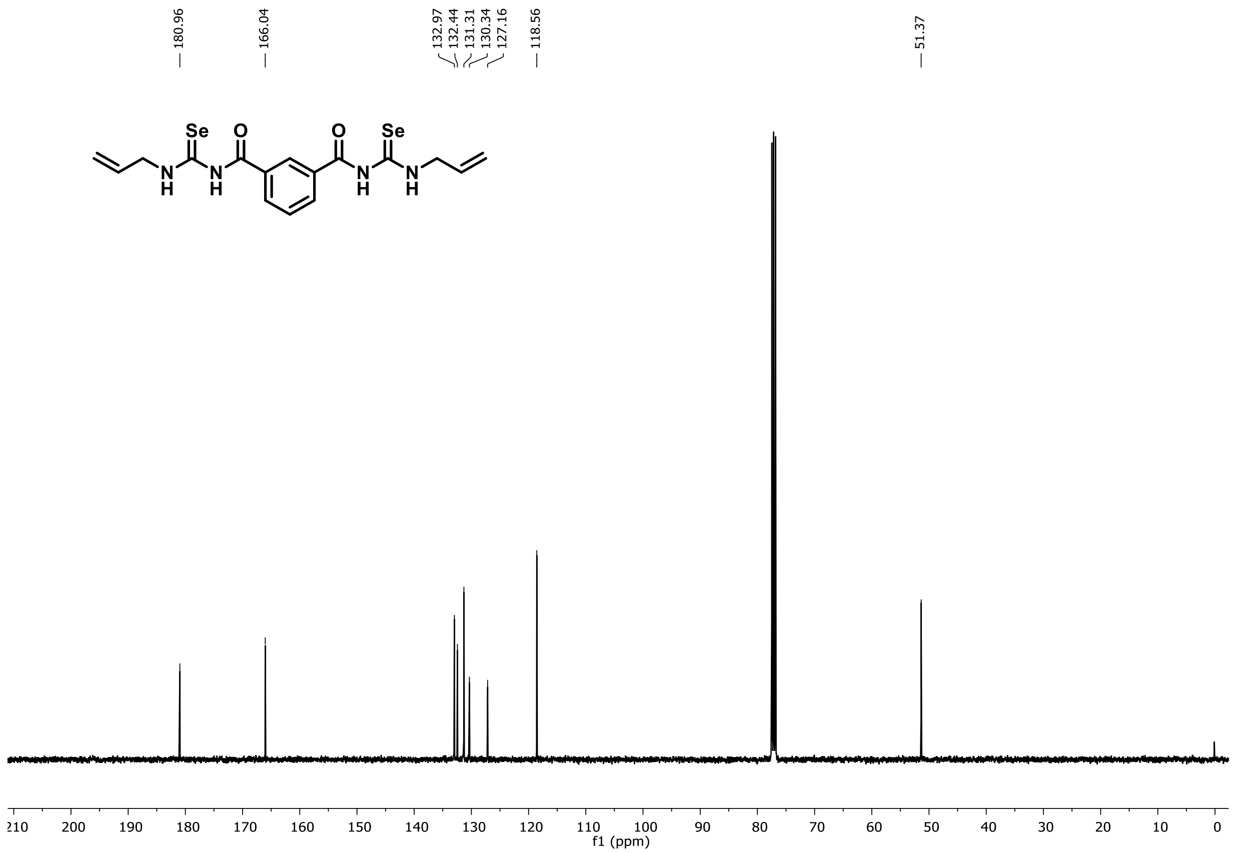

Figure S67. ^{13}C-NMR spectrum of compound **10.II**.

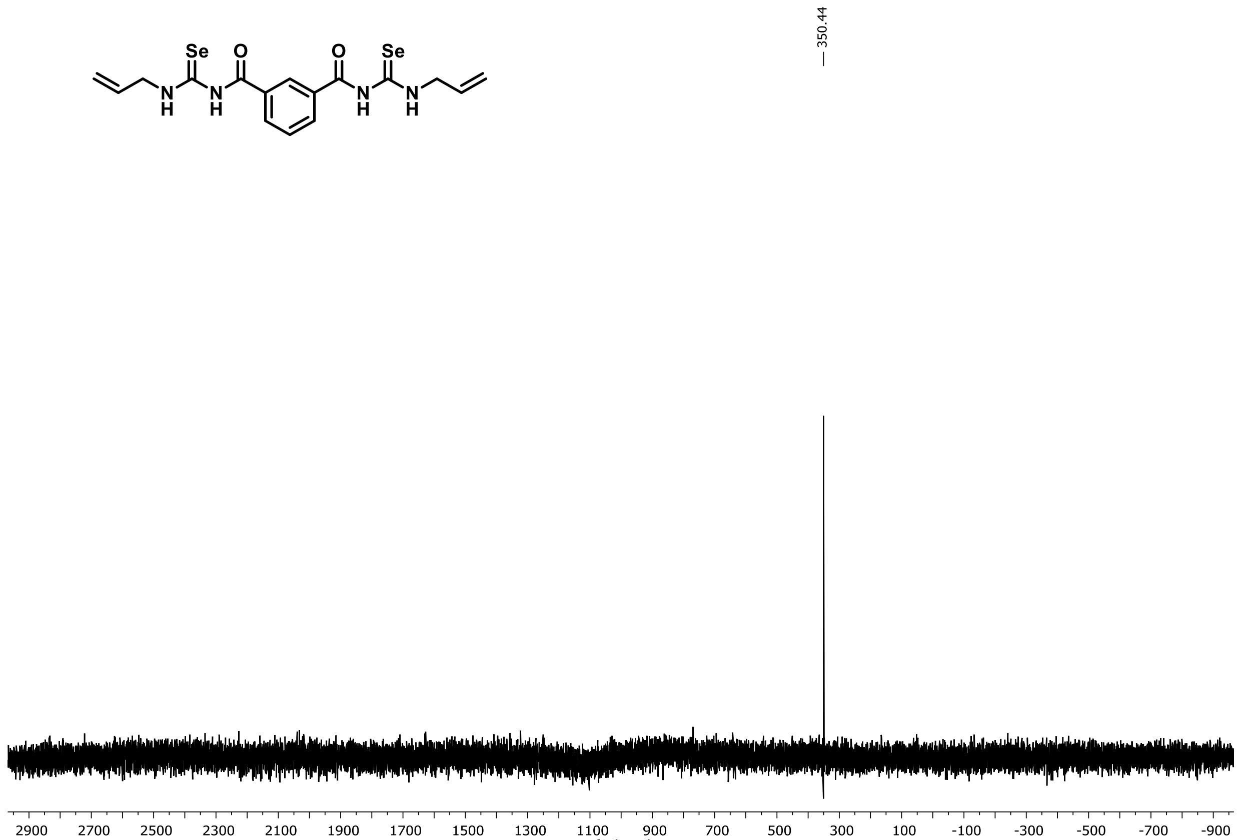

Figure S68. ^{77}Se-NMR spectrum of compound **10.II**.

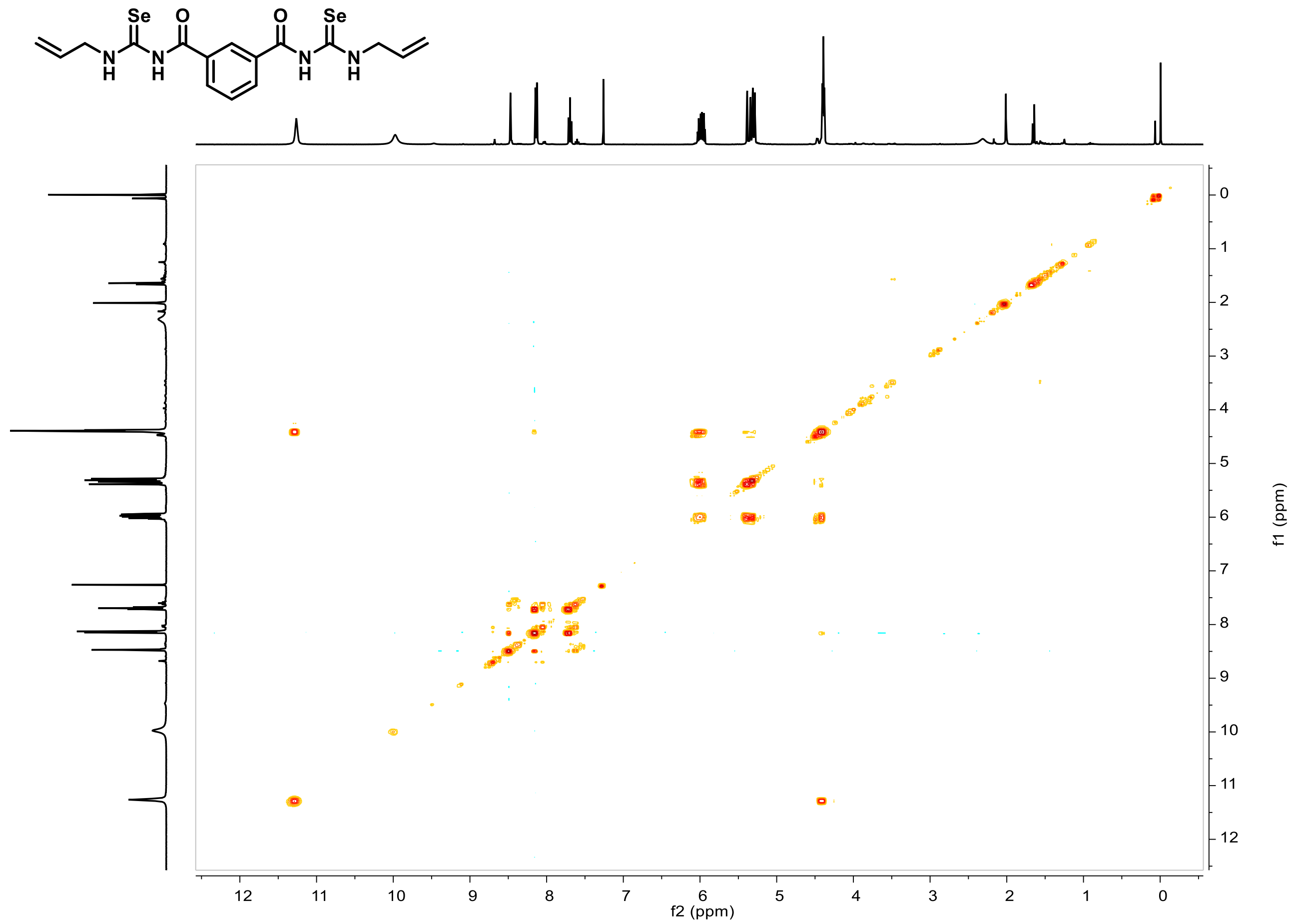

Figure S69. COSY-NMR spectrum of compound **10.II**.

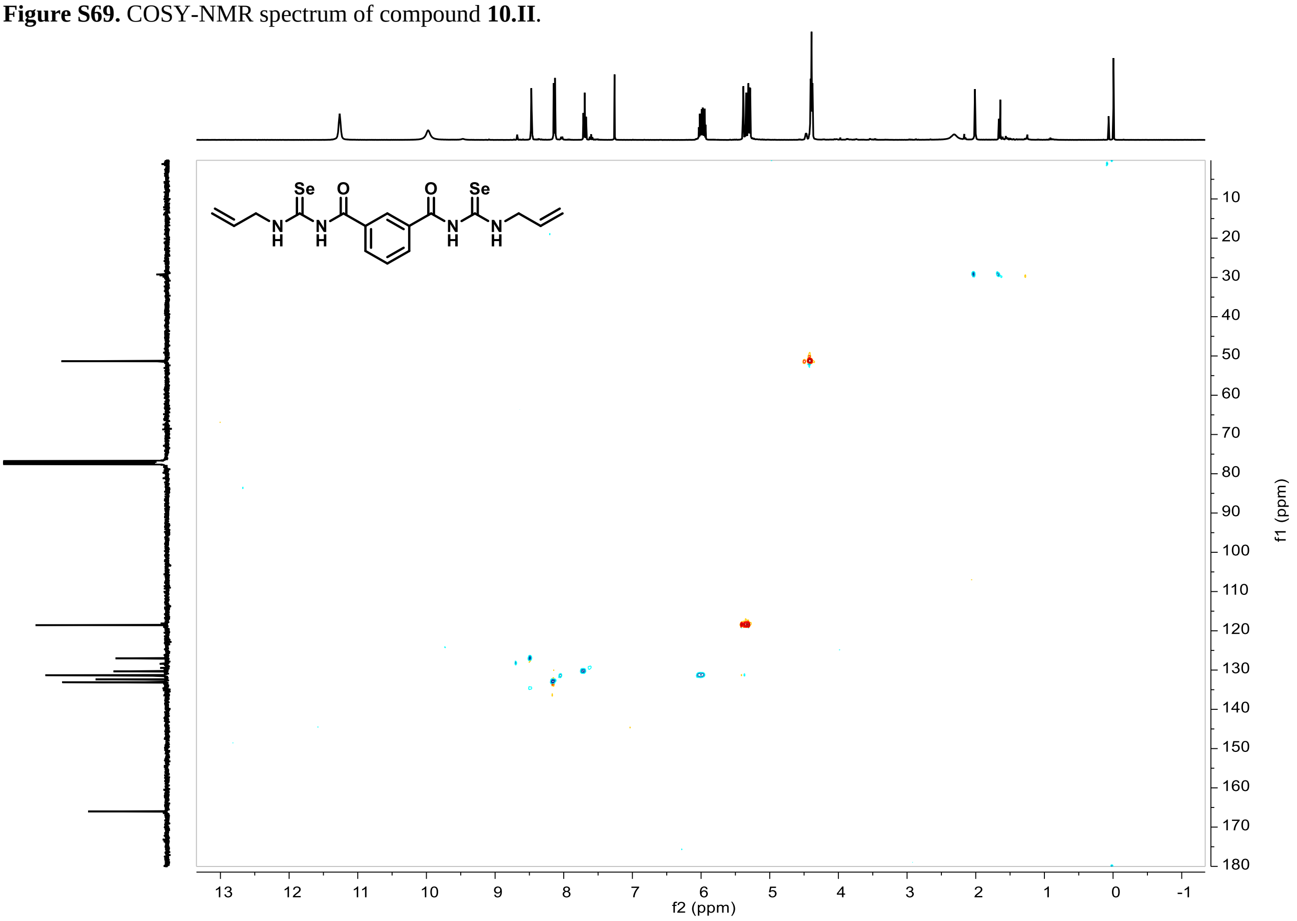

Figure S70. HSQC-NMR spectrum of compound **10.II**.

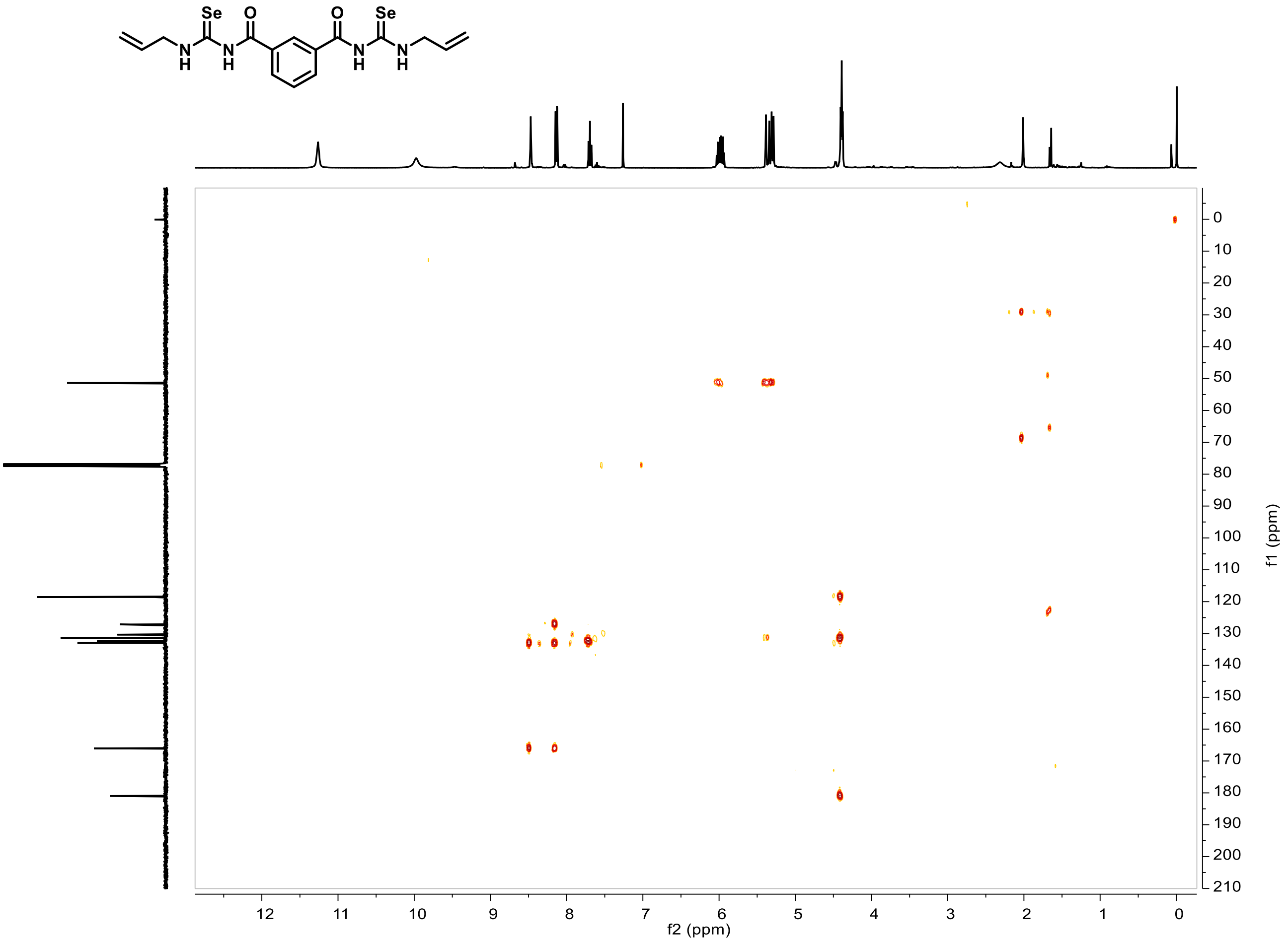

Figure S71. HMBC-NMR spectrum of compound **10.II**.

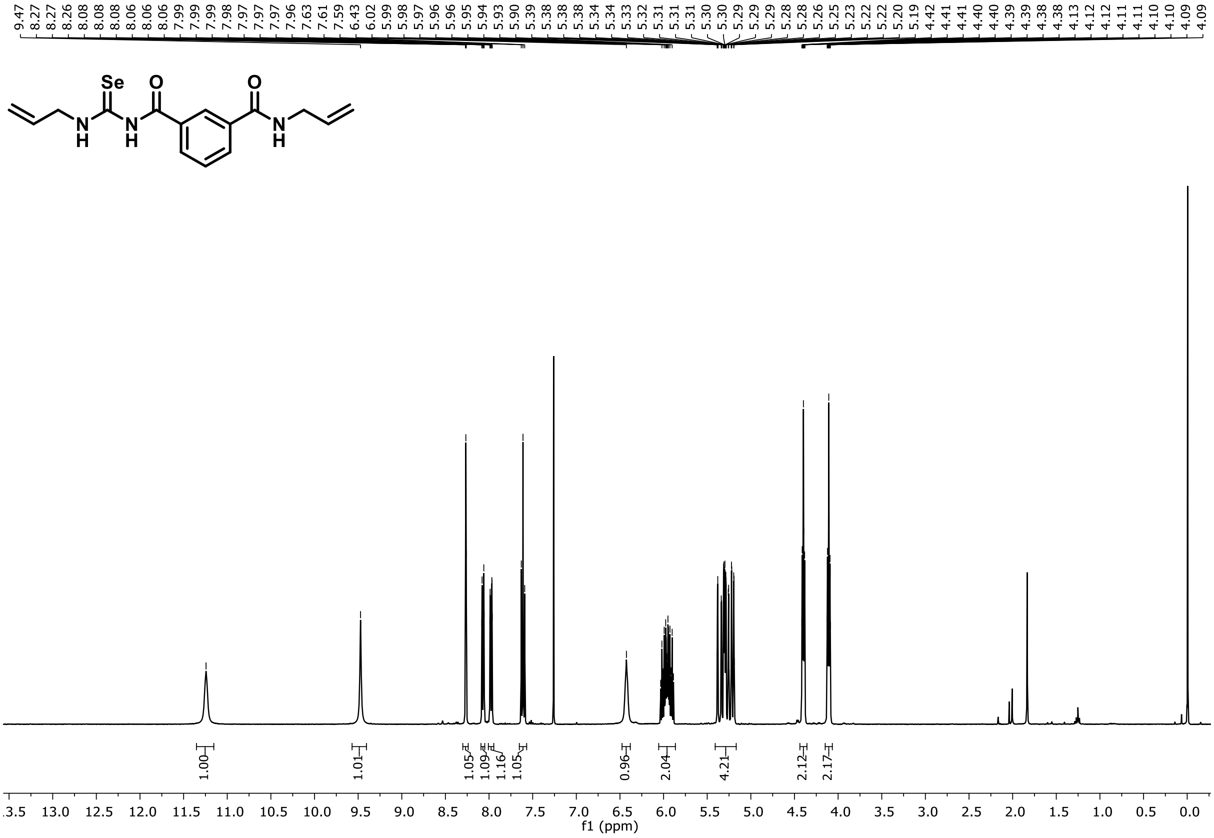

Figure S72. ^{1}H-NMR spectrum of compound **11.II**.

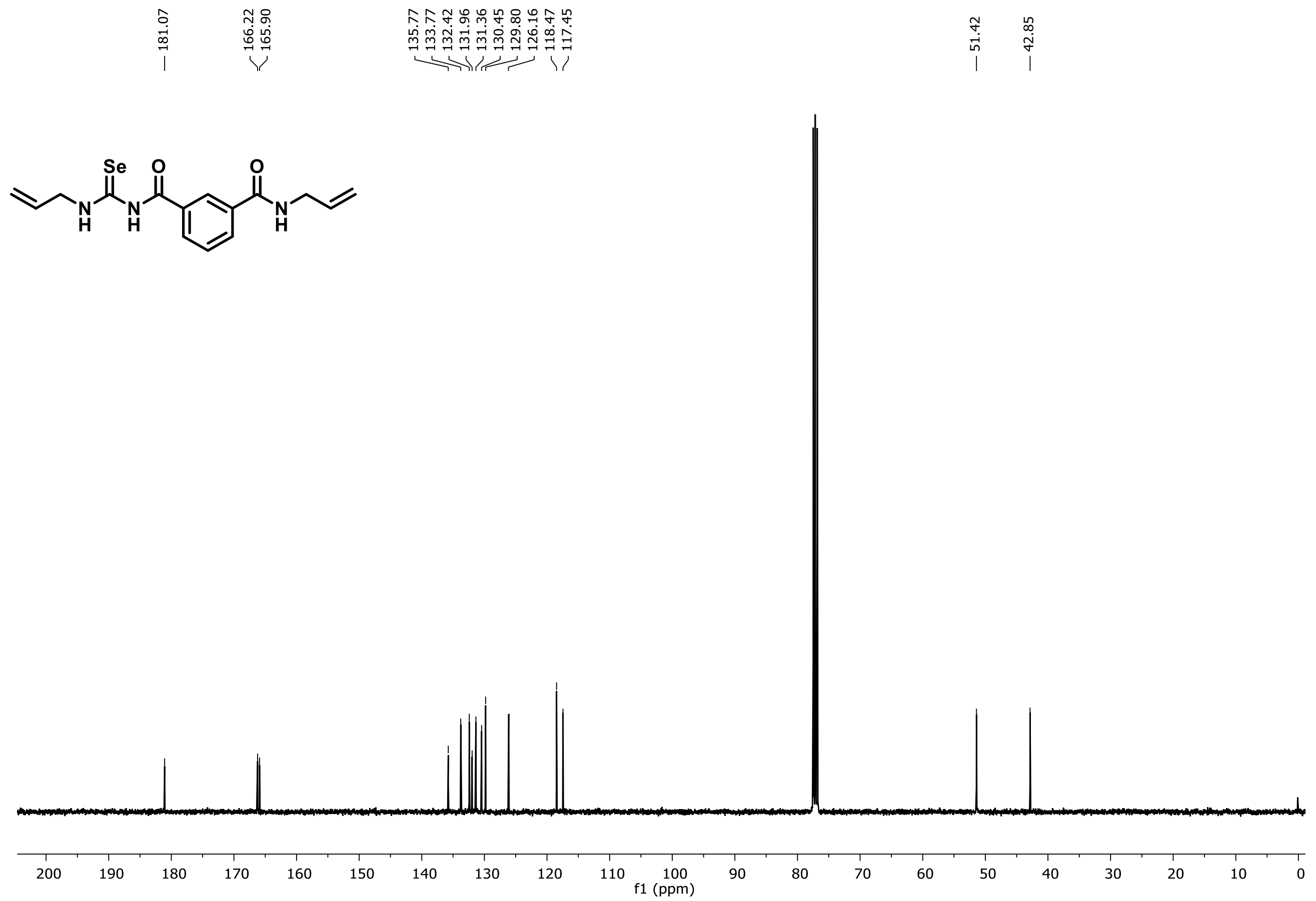

Figure S73. ^{13}C-NMR spectrum of compound **11.II**.

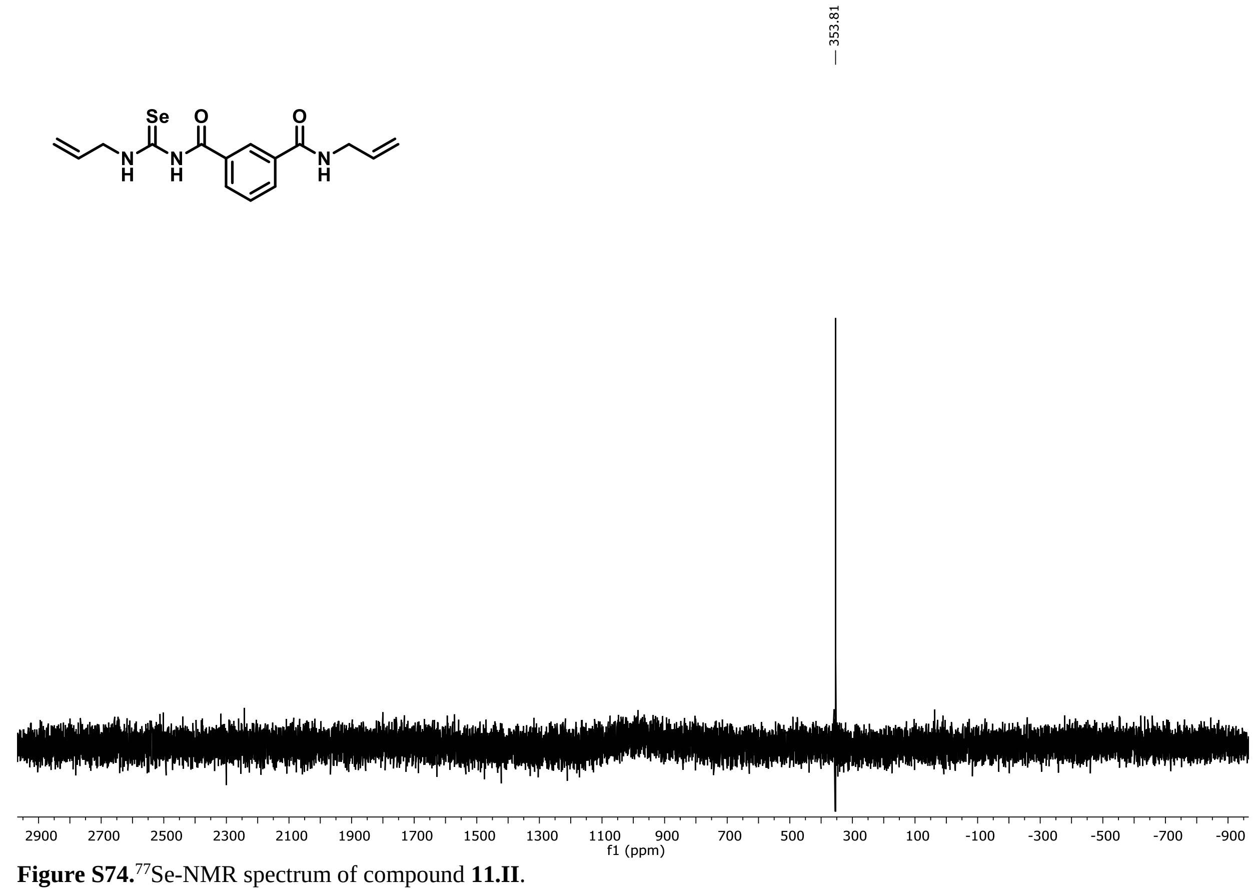

Figure S74.^{77}Se-NMR spectrum of compound **11.II**.

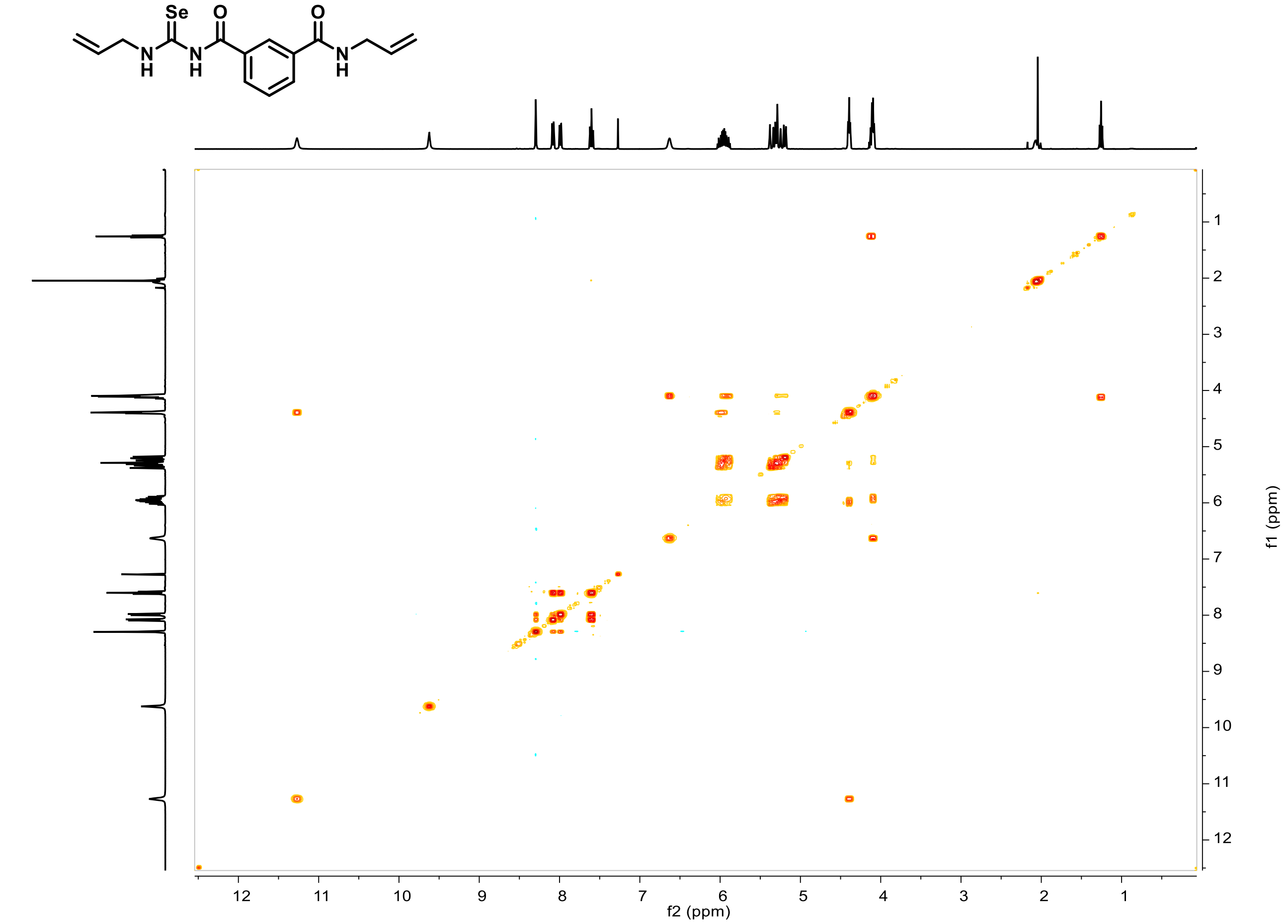

Figure S75. COSY-NMR spectrum of compound **11.II**.

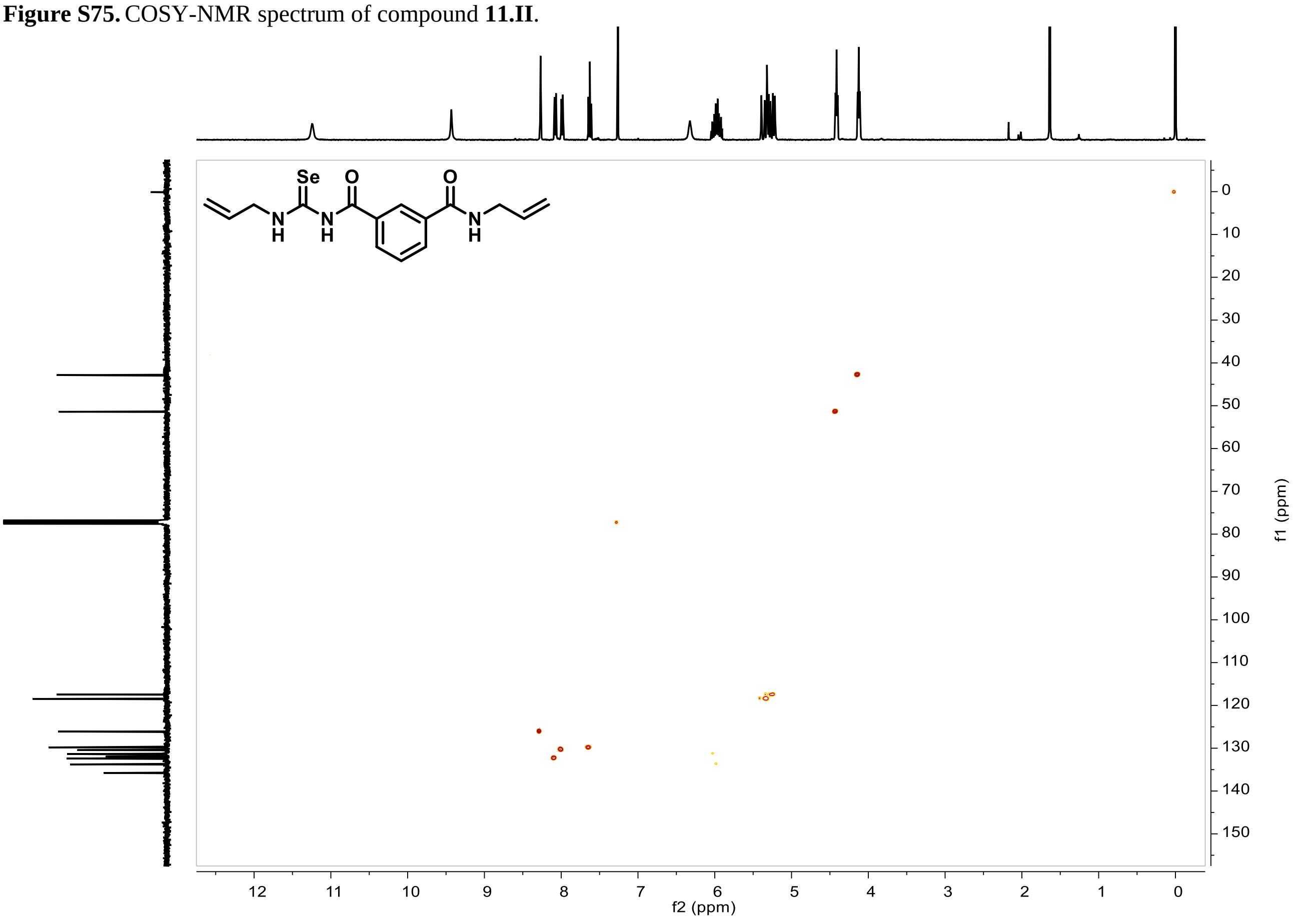

Figure S76. HSQC-NMR spectrum of compound **11.II**.

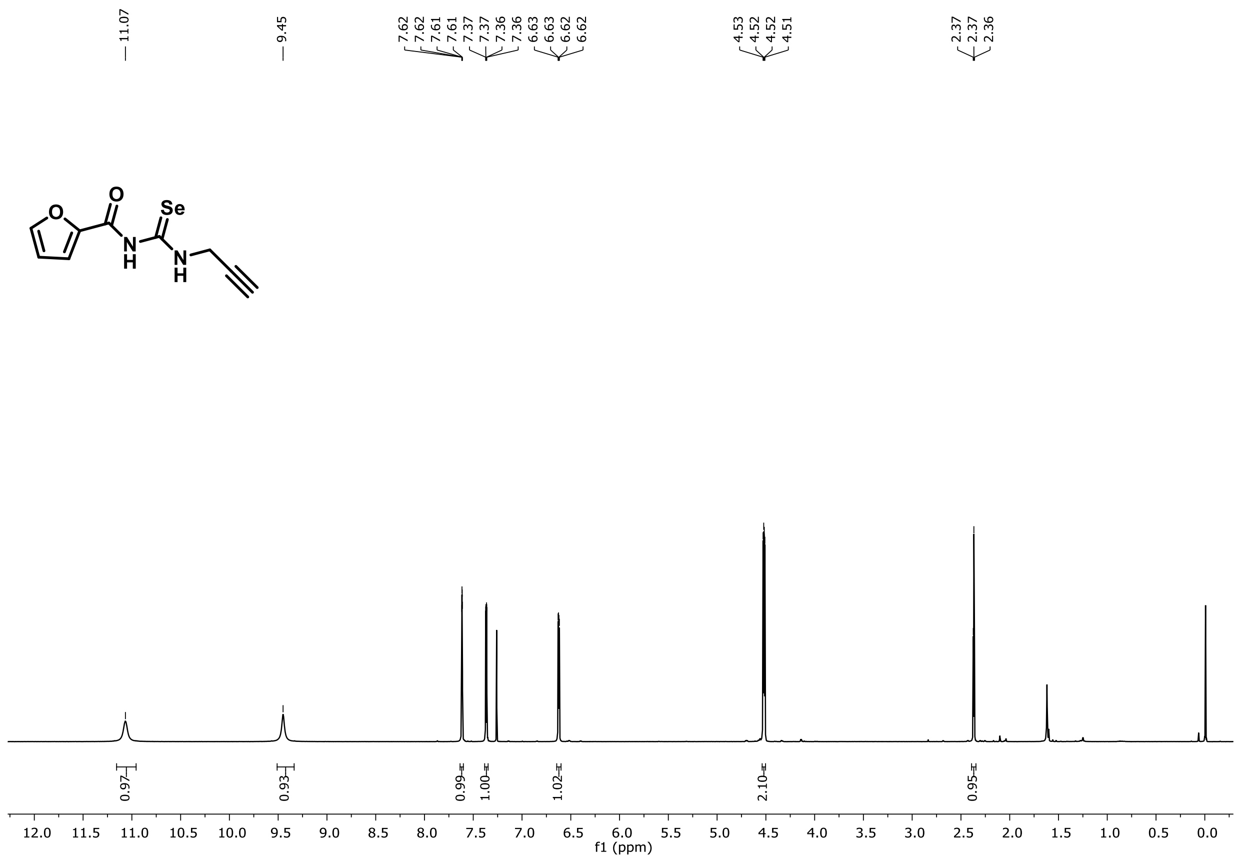

Figure S77. ^{1}H-NMR spectrum of compound **1.III**.

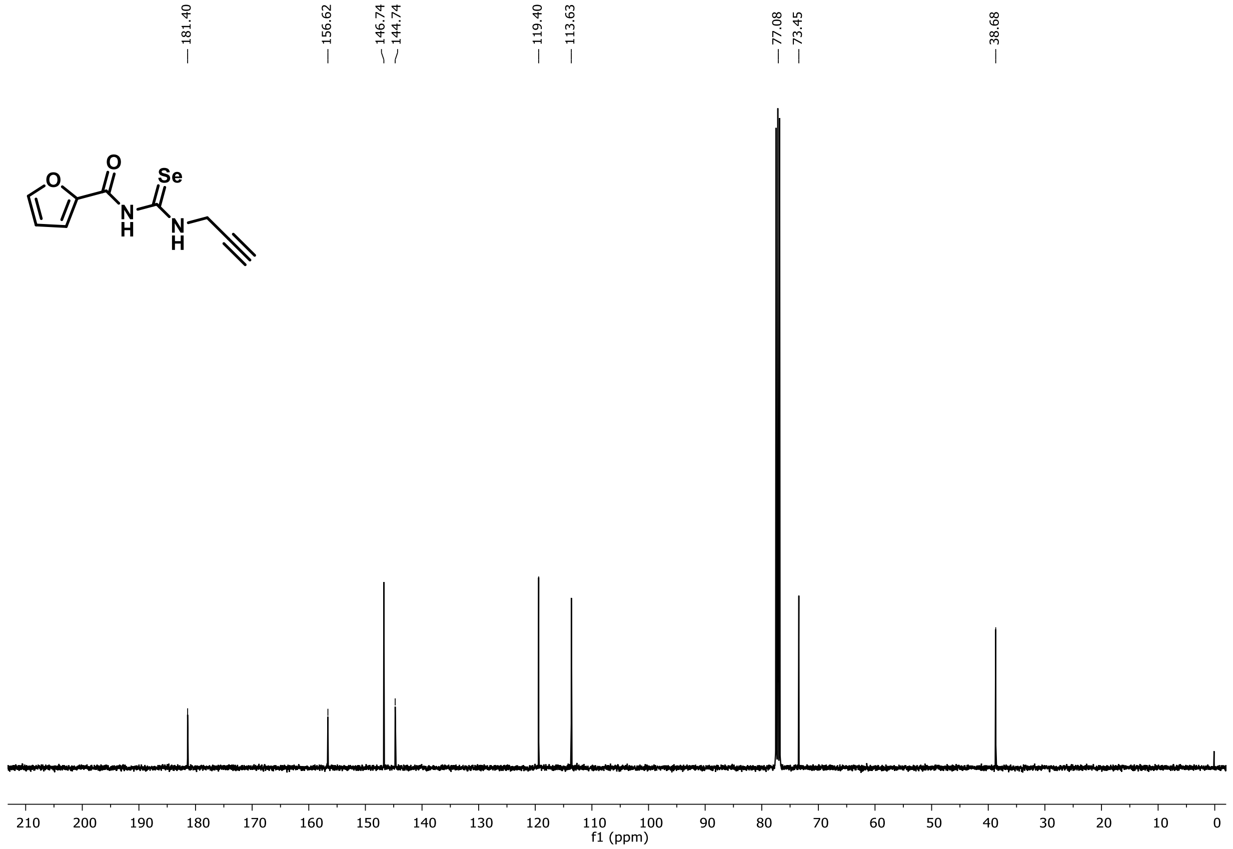

Figure S78. ^{13}C-NMR spectrum of compound **1.III**.

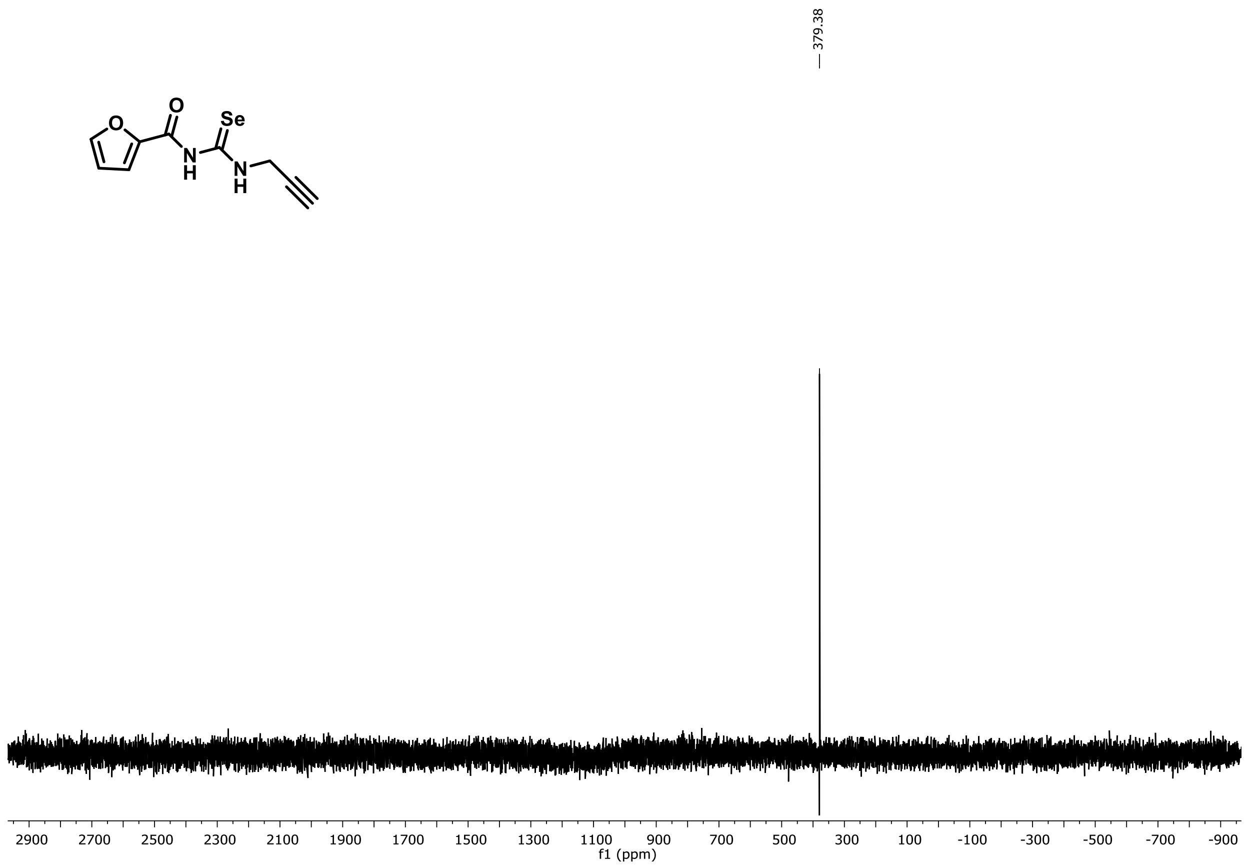

Figure S79.^{77}Se-NMR spectrum of compound **1.III**.

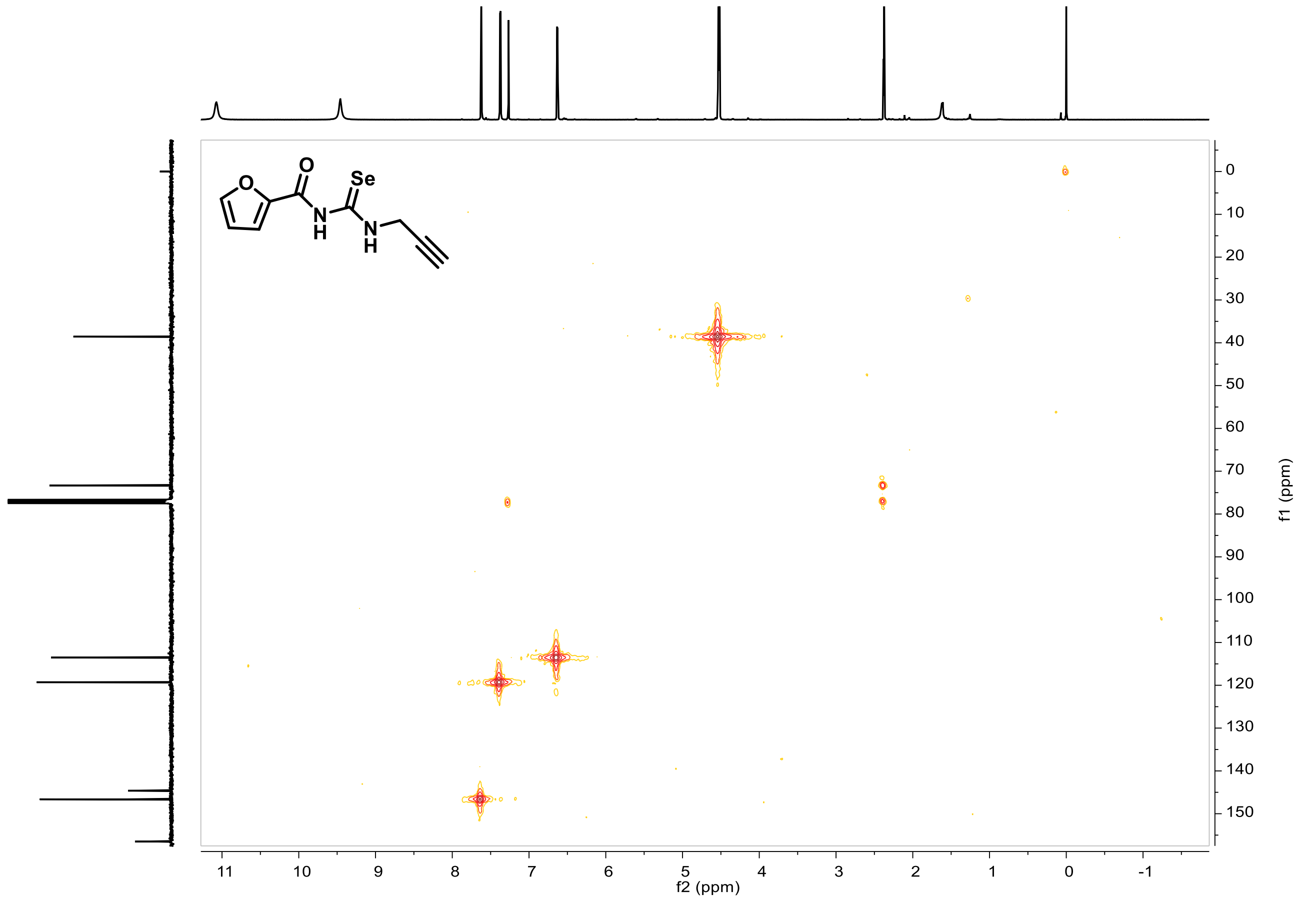

Figure S80. HMQC-NMR spectrum of compound **1.III**.

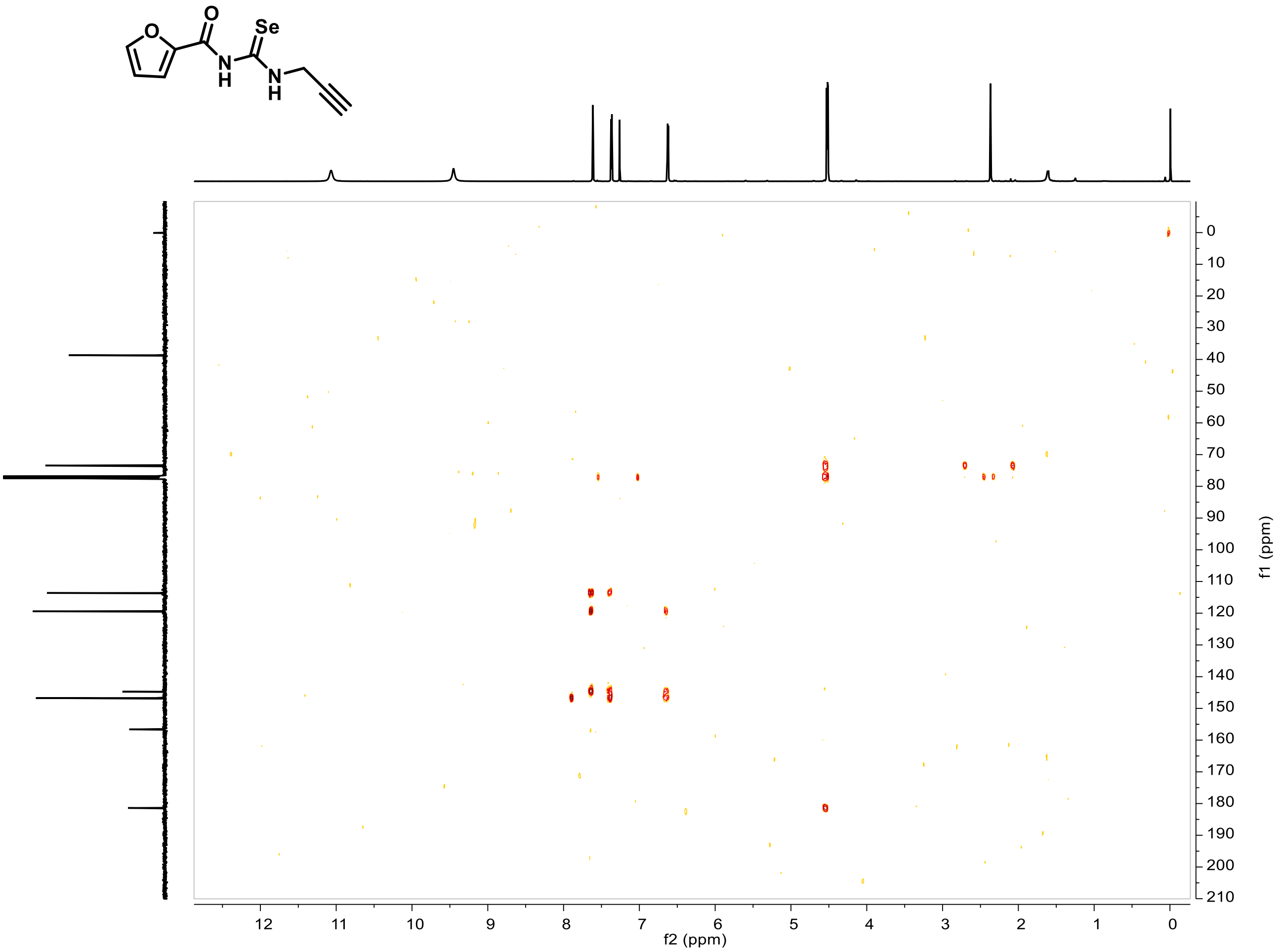

Figure S81. HMBC-NMR spectrum of compound **1.III**.

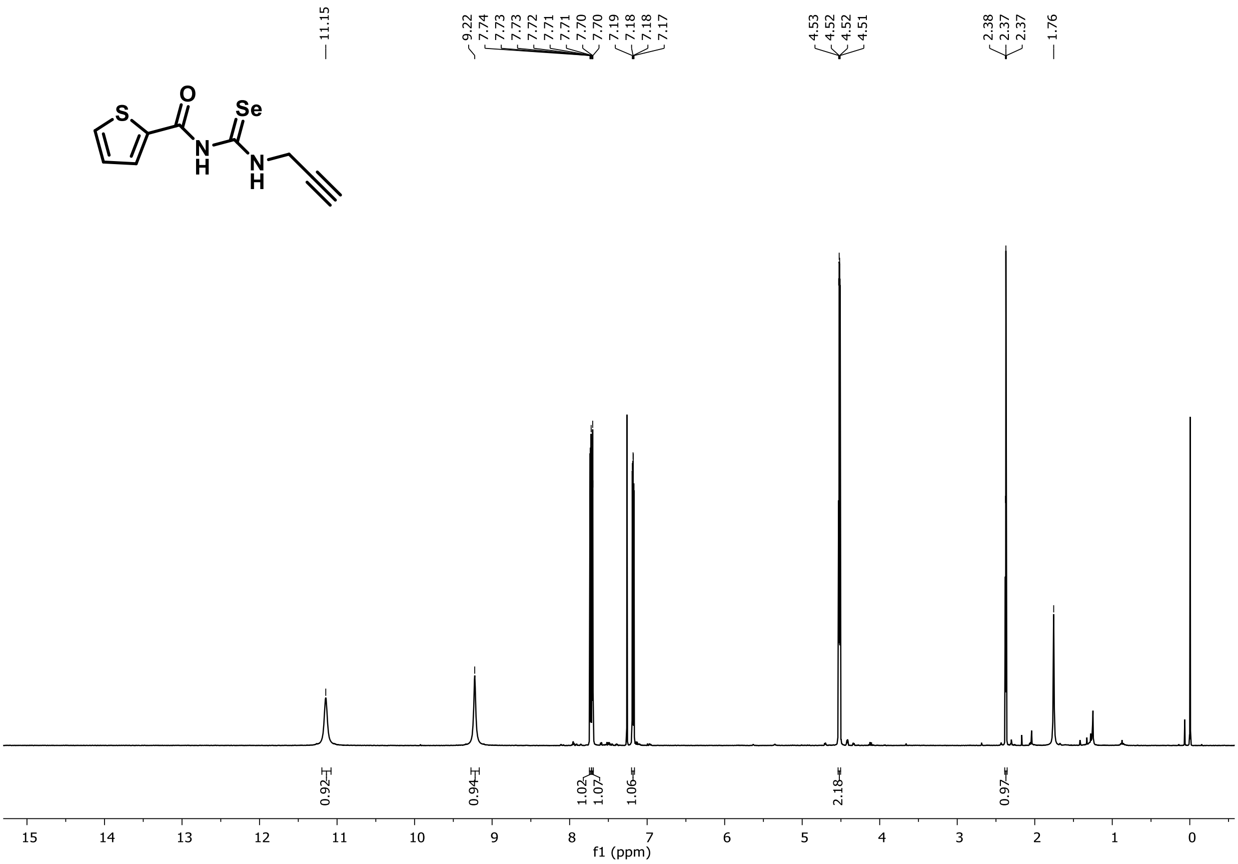

Figure S82. ^{1}H-NMR spectrum of compound **2.III**.

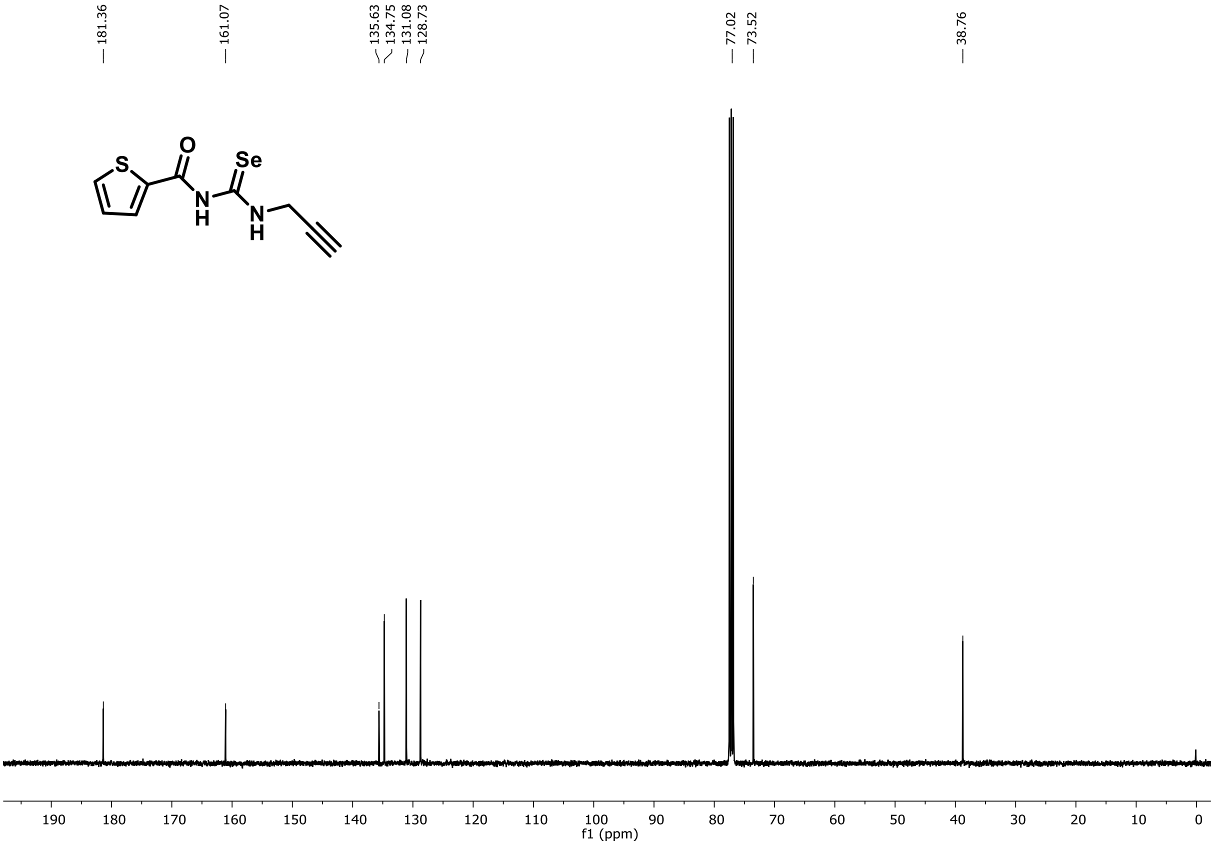

Figure S83. ^{13}C-NMR spectrum of compound **2.III**.

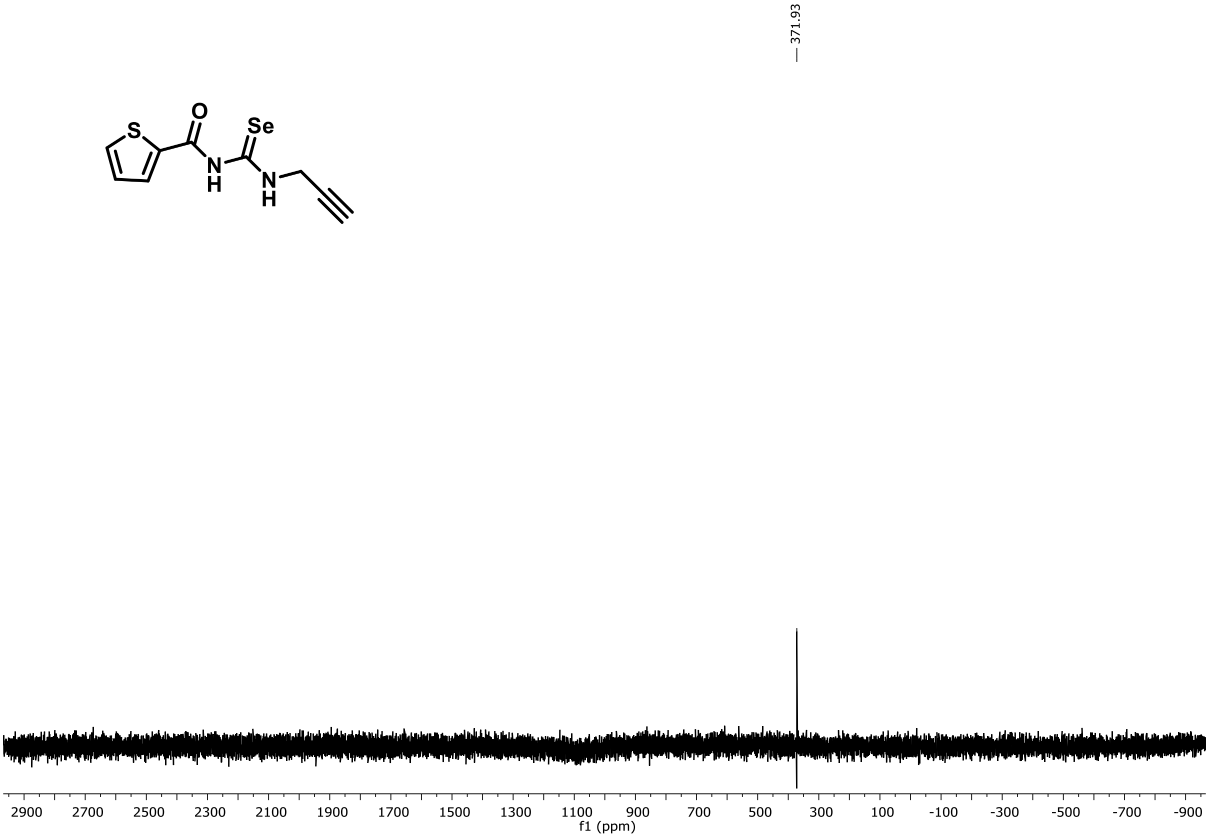

Figure S84.^{77}Se-NMR spectrum of compound **2.III**.

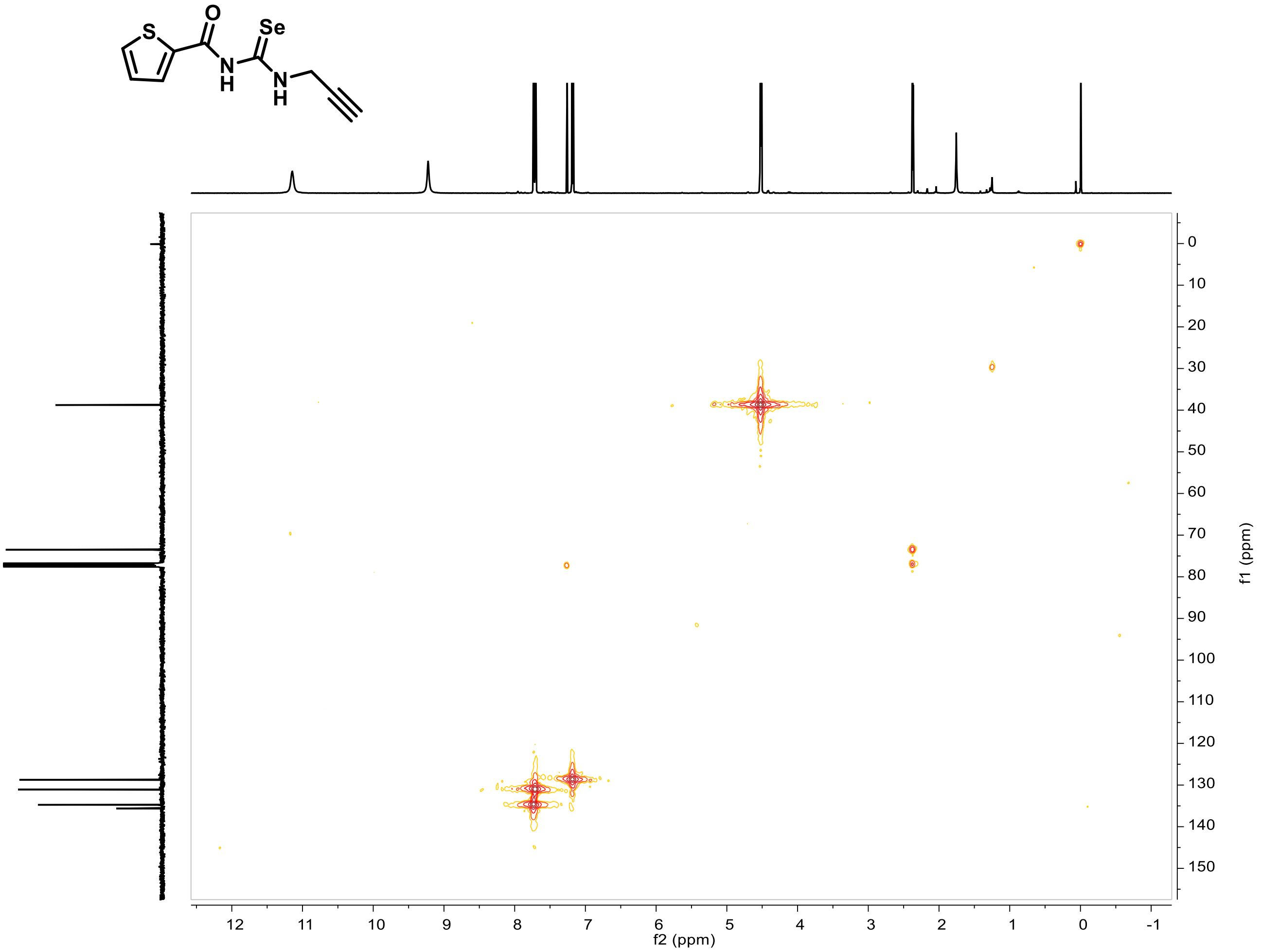

Figure S85. HMQC-NMR spectrum of compound **2.III**.

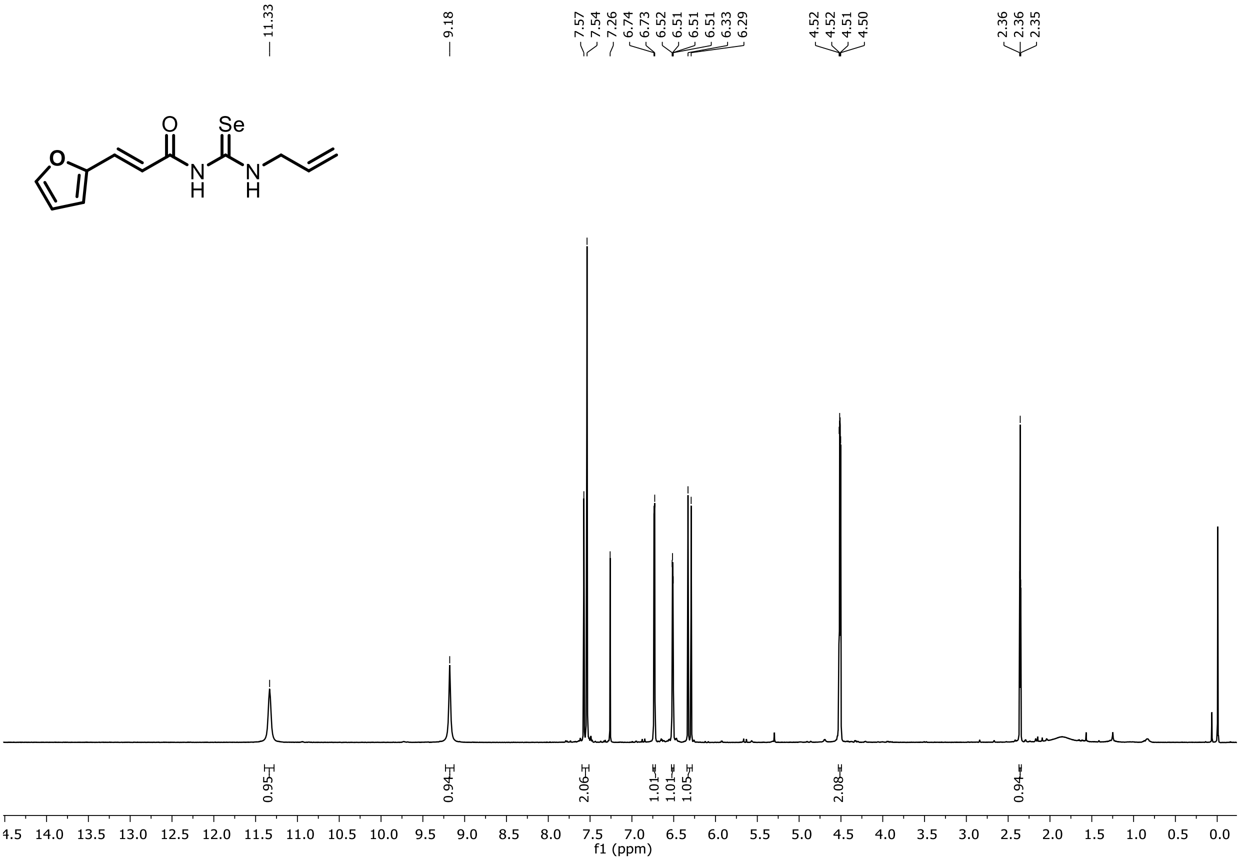

Figure S86. ^{1}H-NMR spectrum of compound **3.III**.

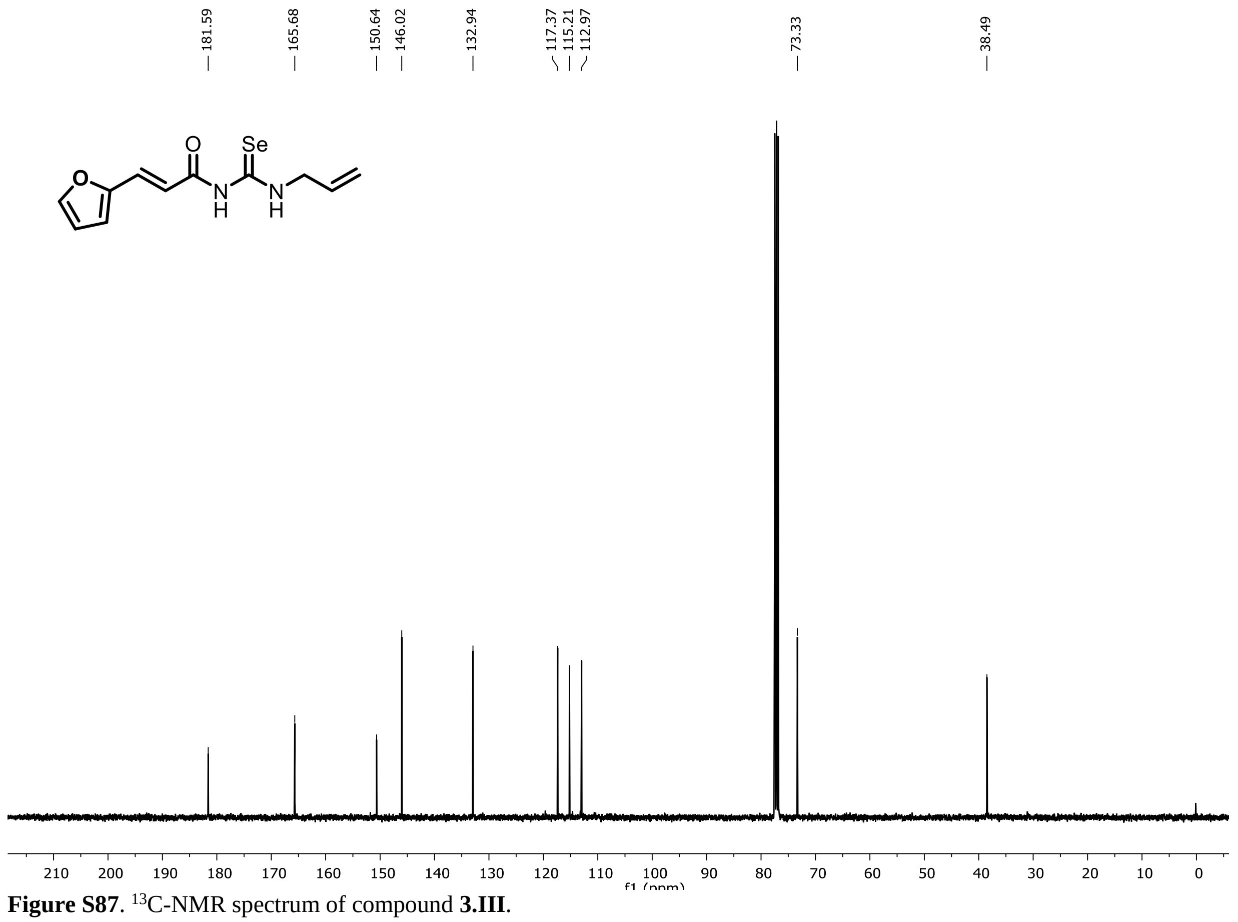

Figure S87. ^{13}C-NMR spectrum of compound **3.III**.

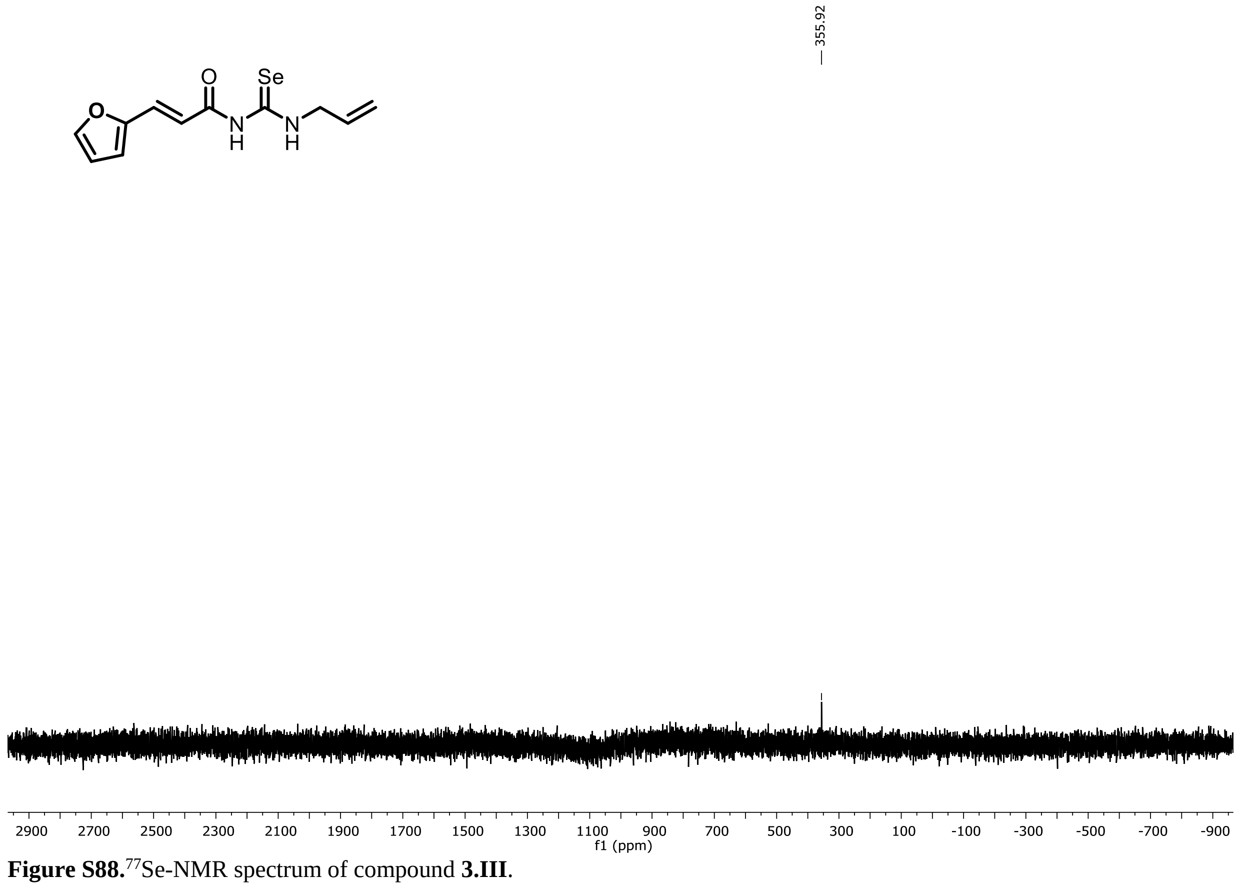

Figure S88.^{77}Se-NMR spectrum of compound **3.III**.

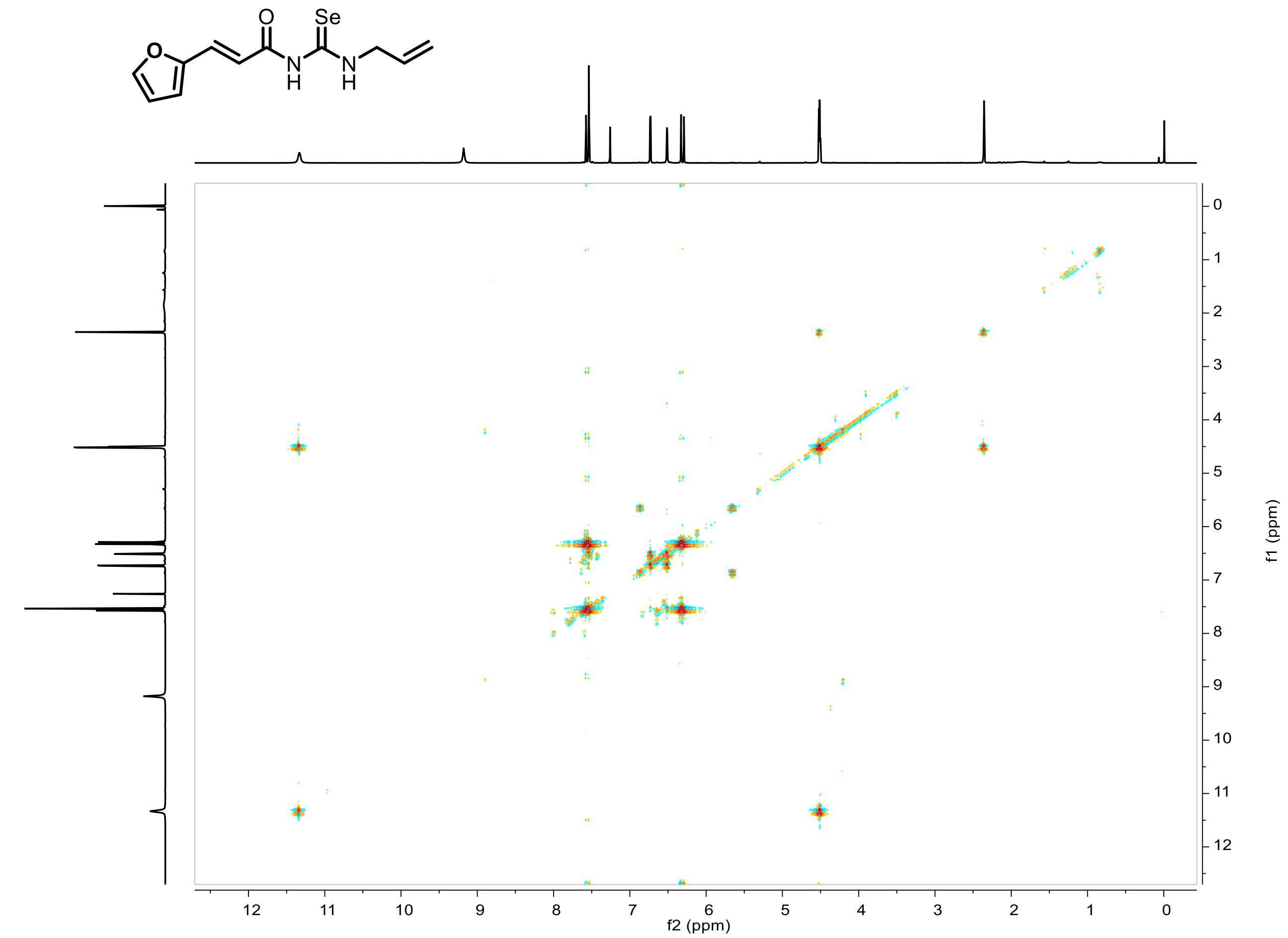

Figure S89.COSY-NMR spectrum of compound **3.III**.

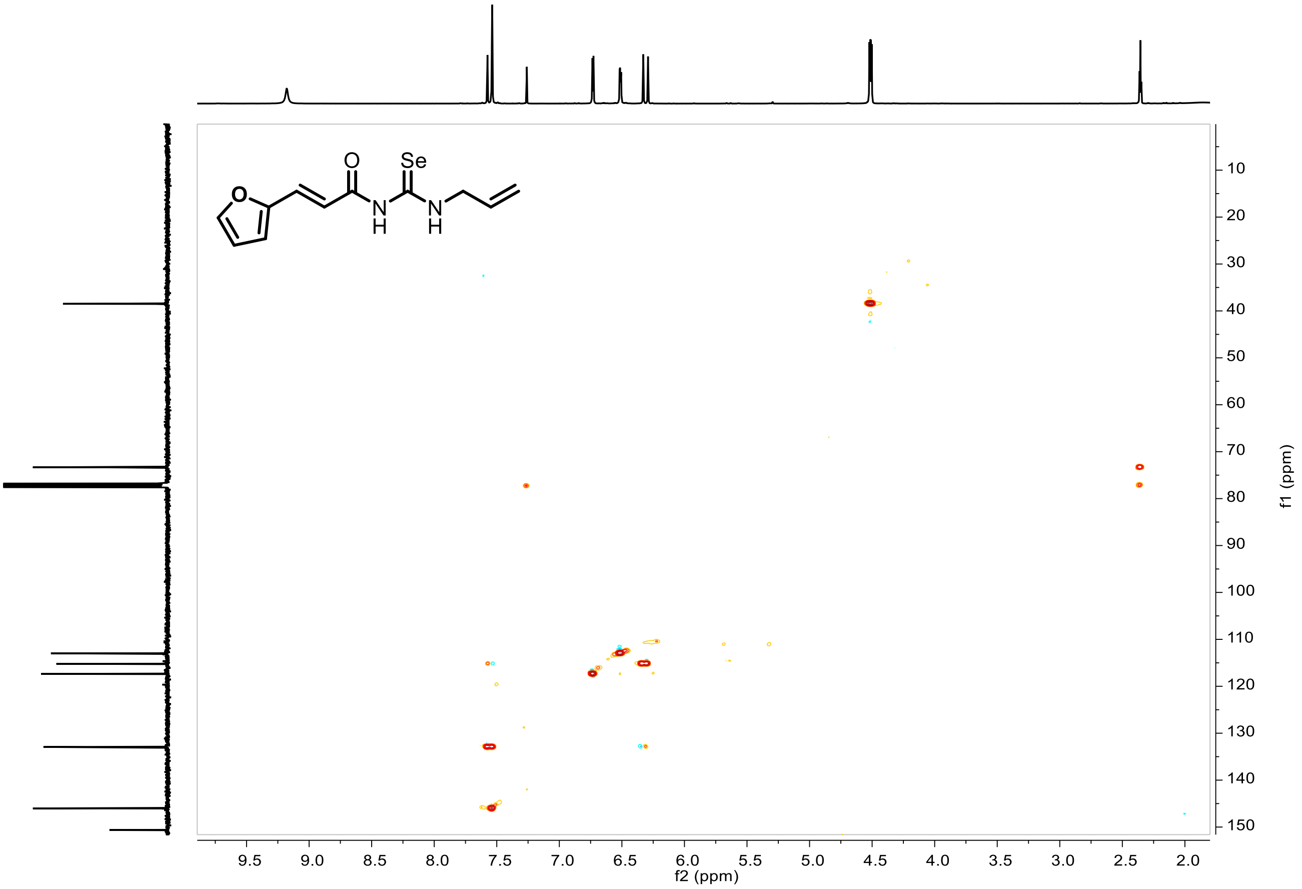

Figure S90.HSQC-NMR spectrum of compound **3.III**.

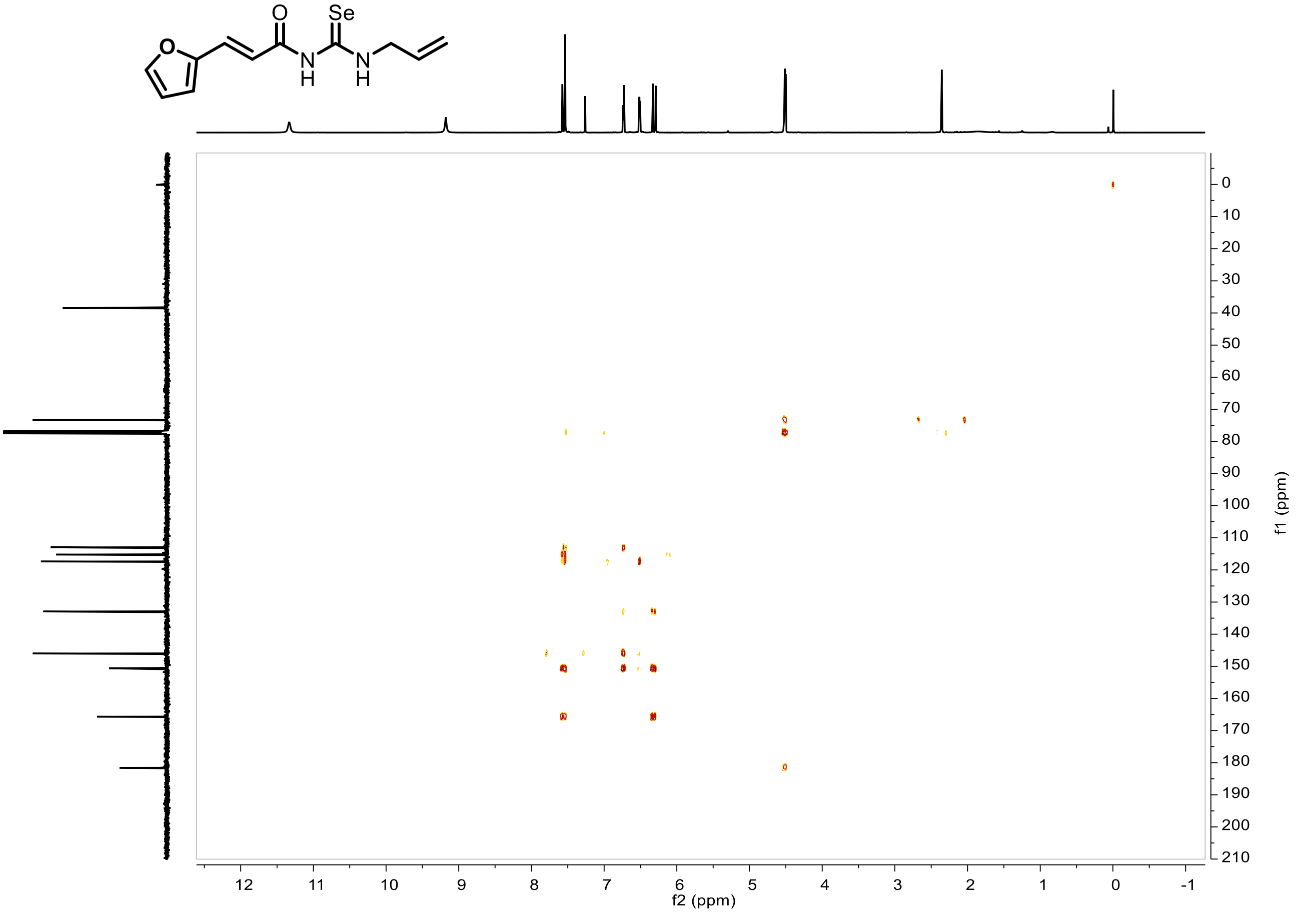

Figure S91.HMBC-NMR spectrum of compound **3.III**.

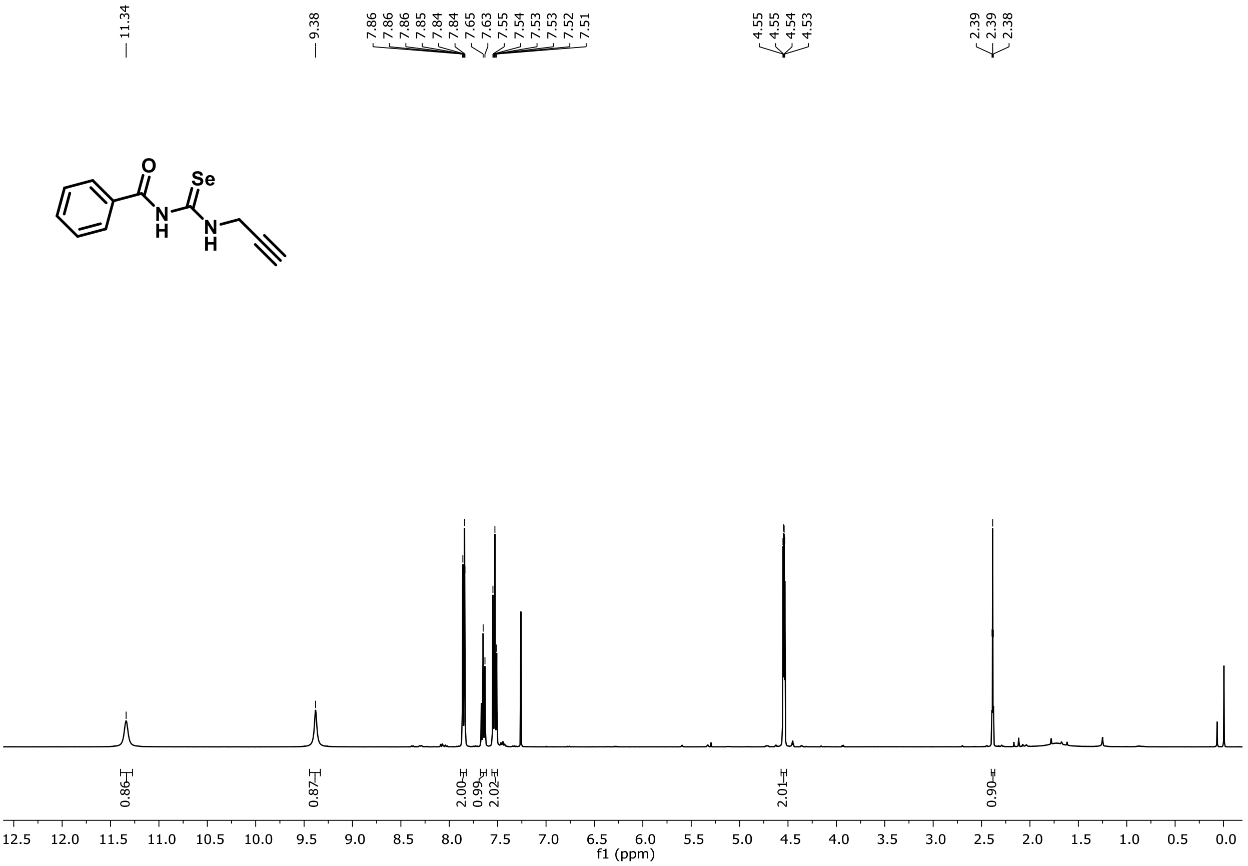

Figure S92. ^{1}H-NMR spectrum of compound **5.III**.

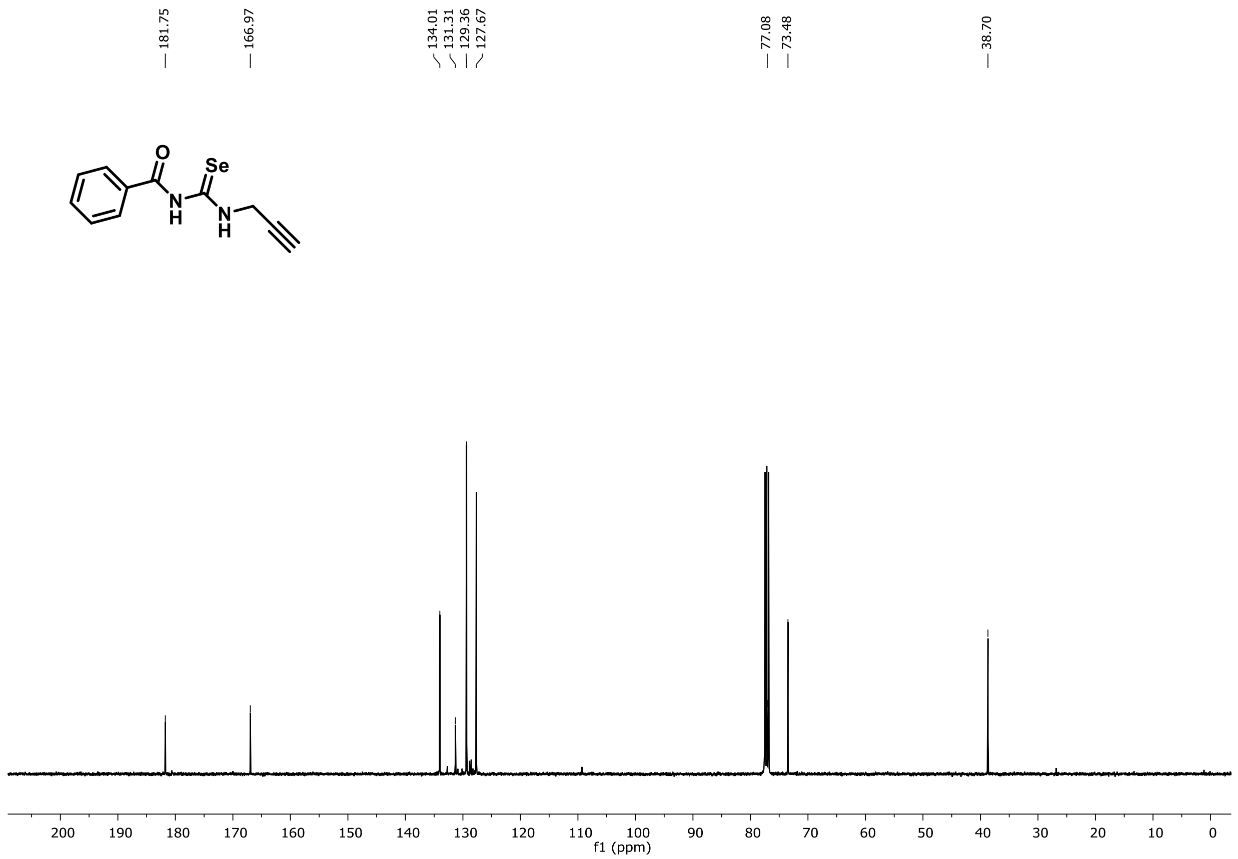

Figure S93. ^{13}C-NMR spectrum of compound **5.III**.

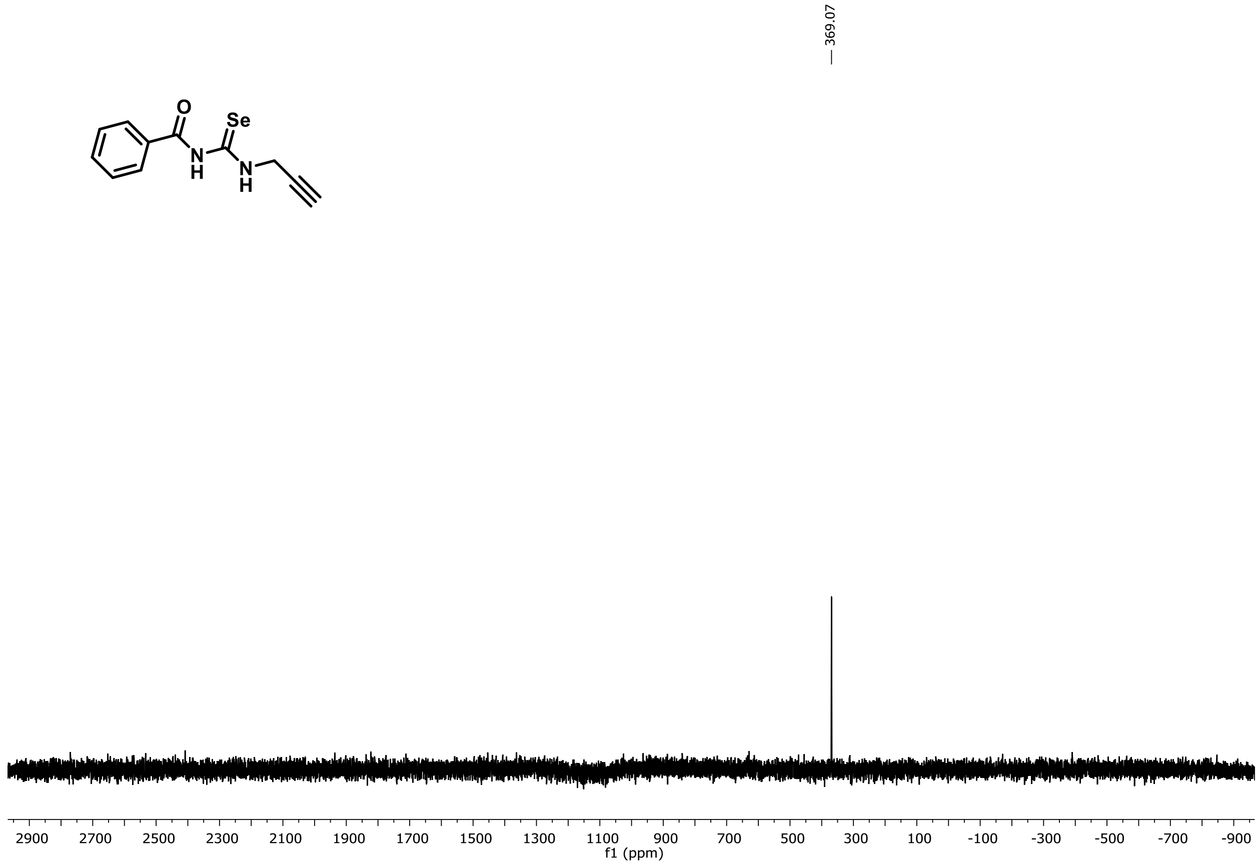

Figure S94.^{77}Se-NMR spectrum of compound **5.III**.

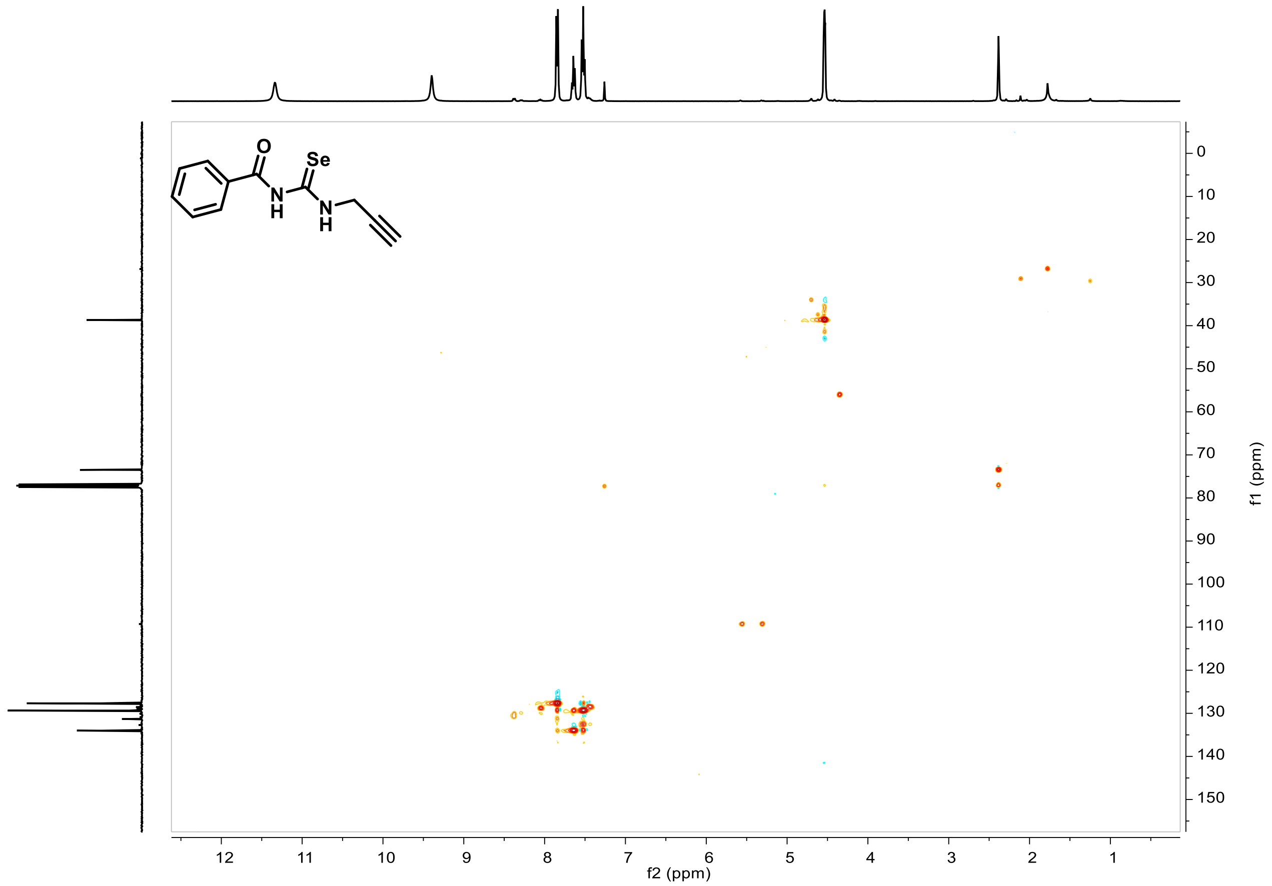

Figure S95. HSQC-NMR spectrum of compound **5.III**.

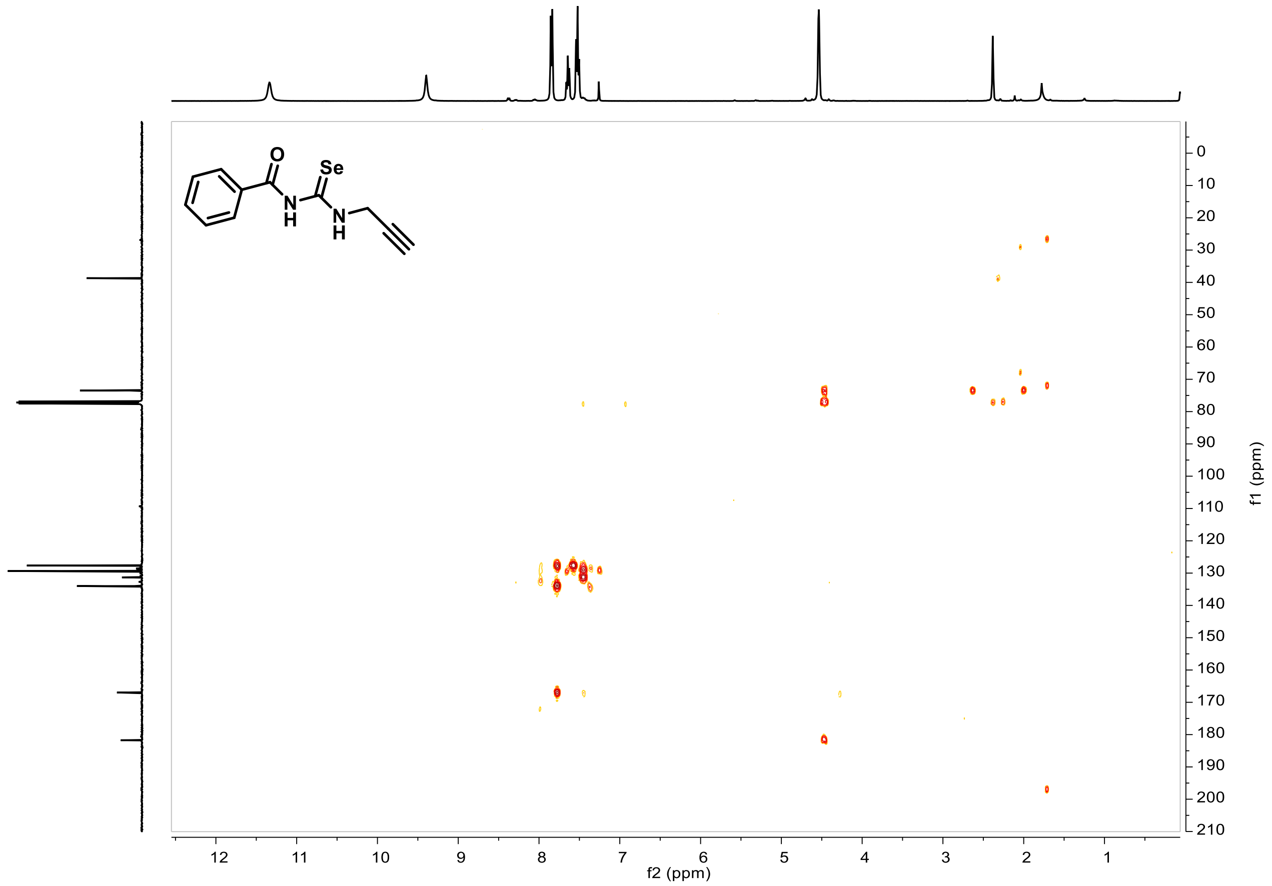

Figure S96. HMBC-NMR spectrum of compound **5.III**.

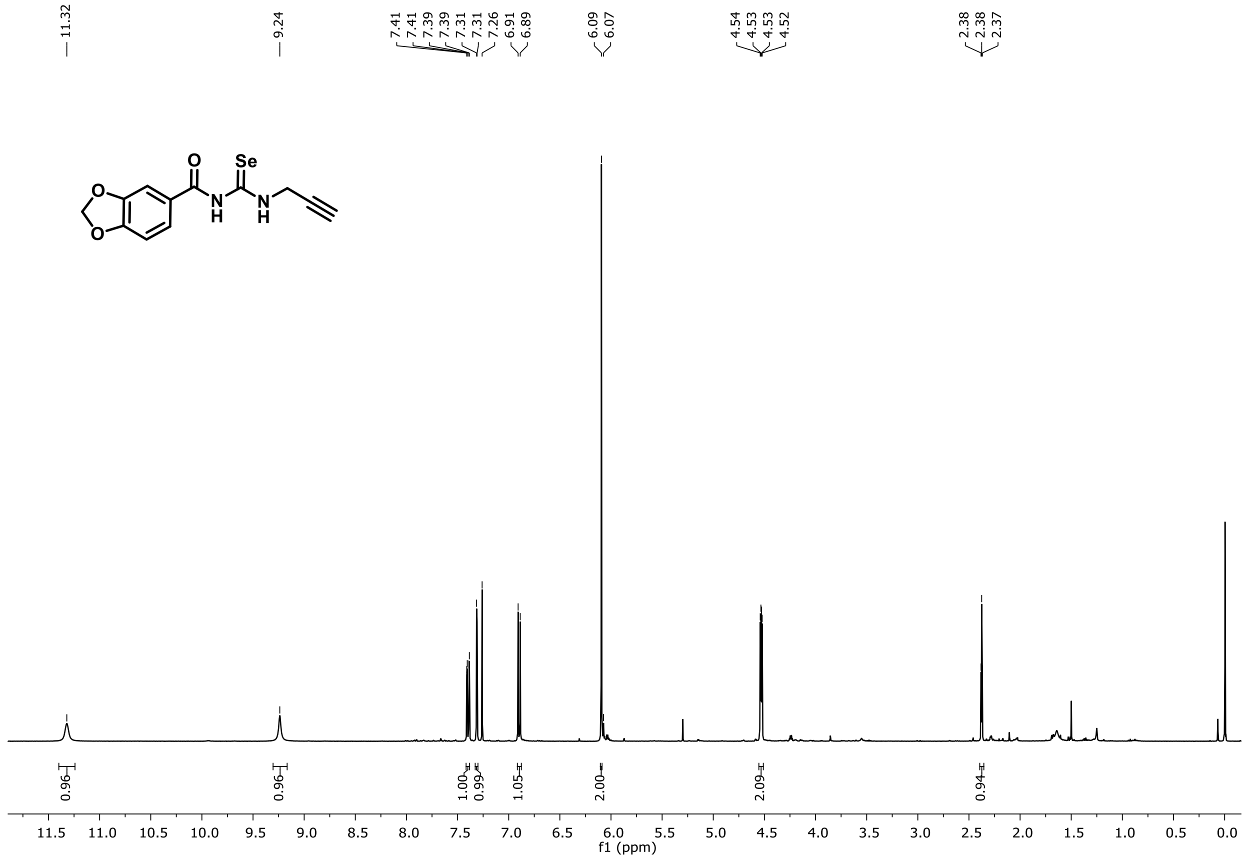

Figure S97. ^{1}H-NMR spectrum of compound **8.III**.

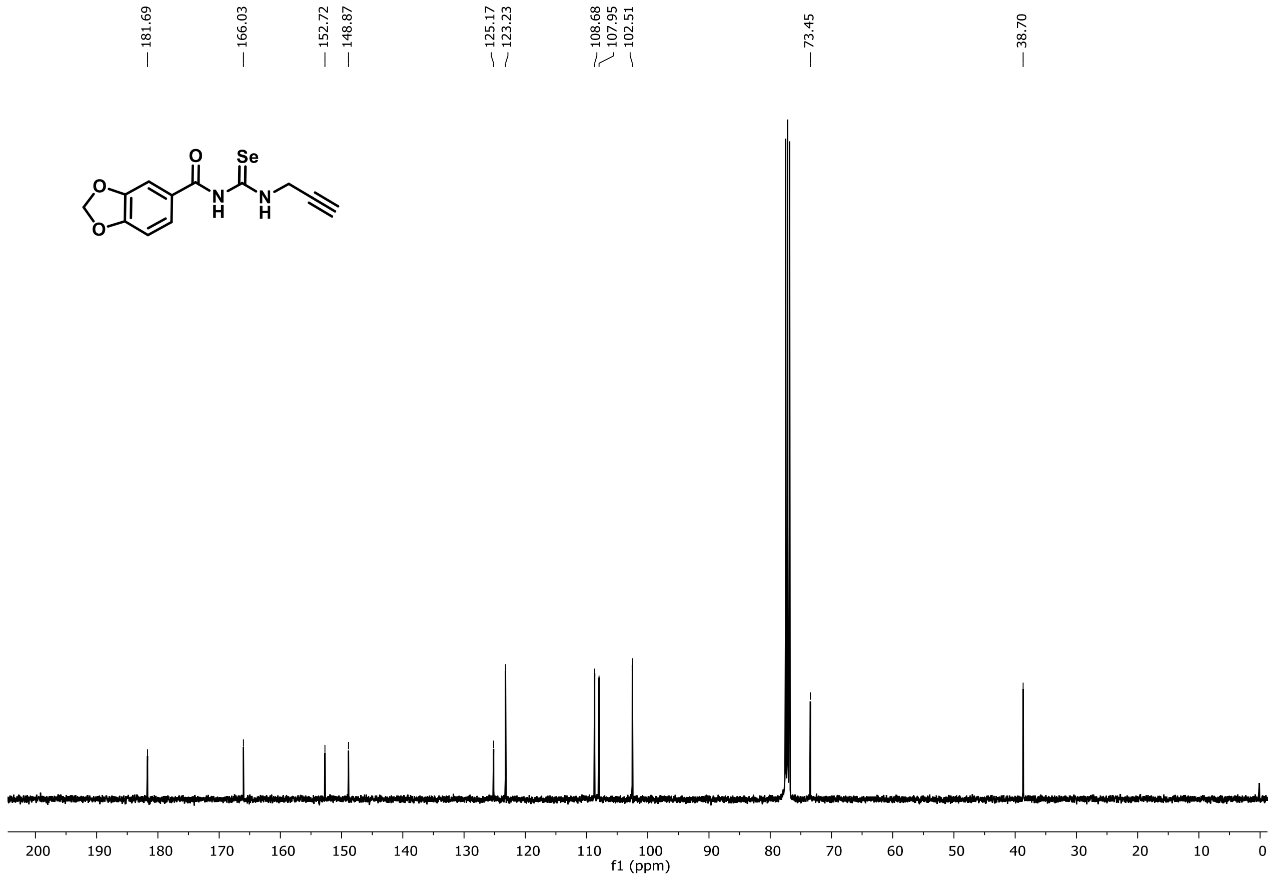

Figure S98. ^{13}C-NMR spectrum of compound **8.III**.

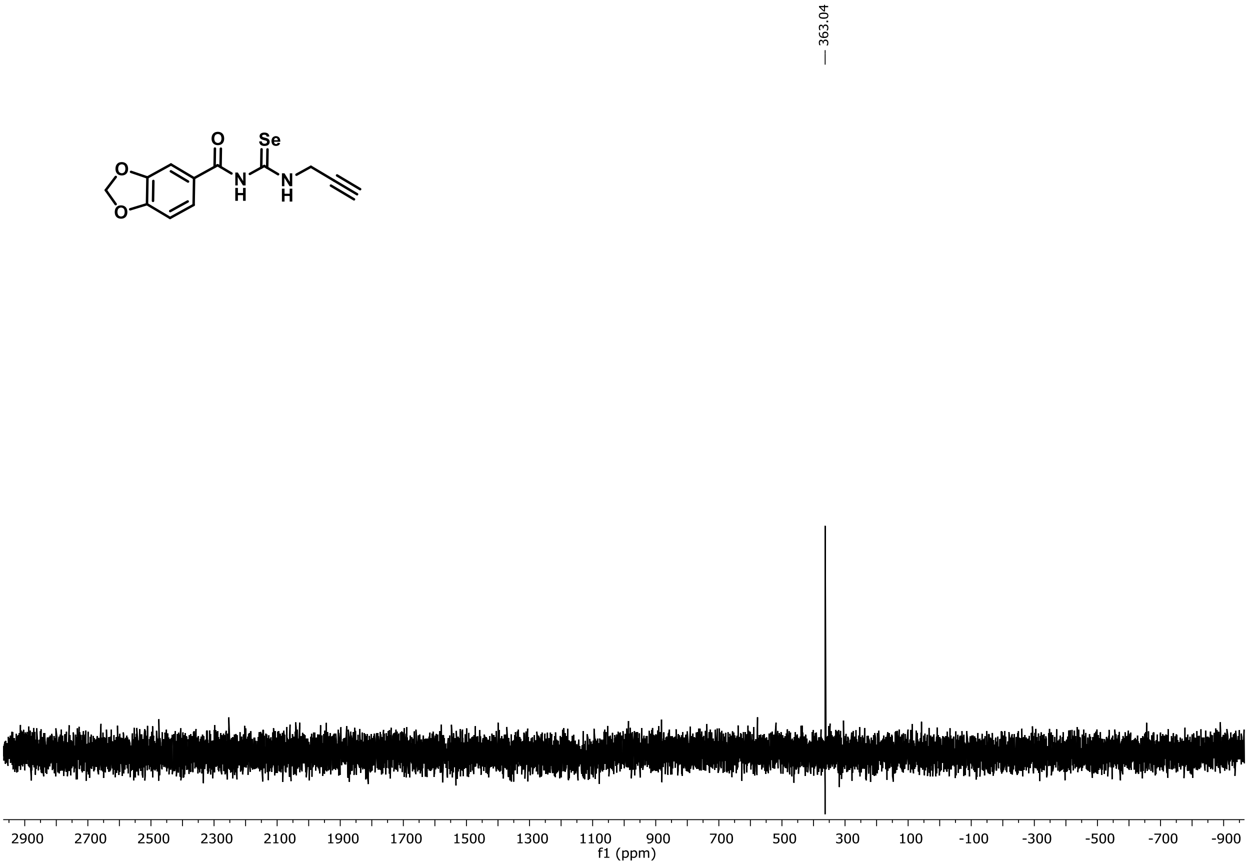

Figure S99. ^{77}Se-NMR spectrum of compound **8.III**.

DPPH inhibitory activity at 0.003 mg/mL

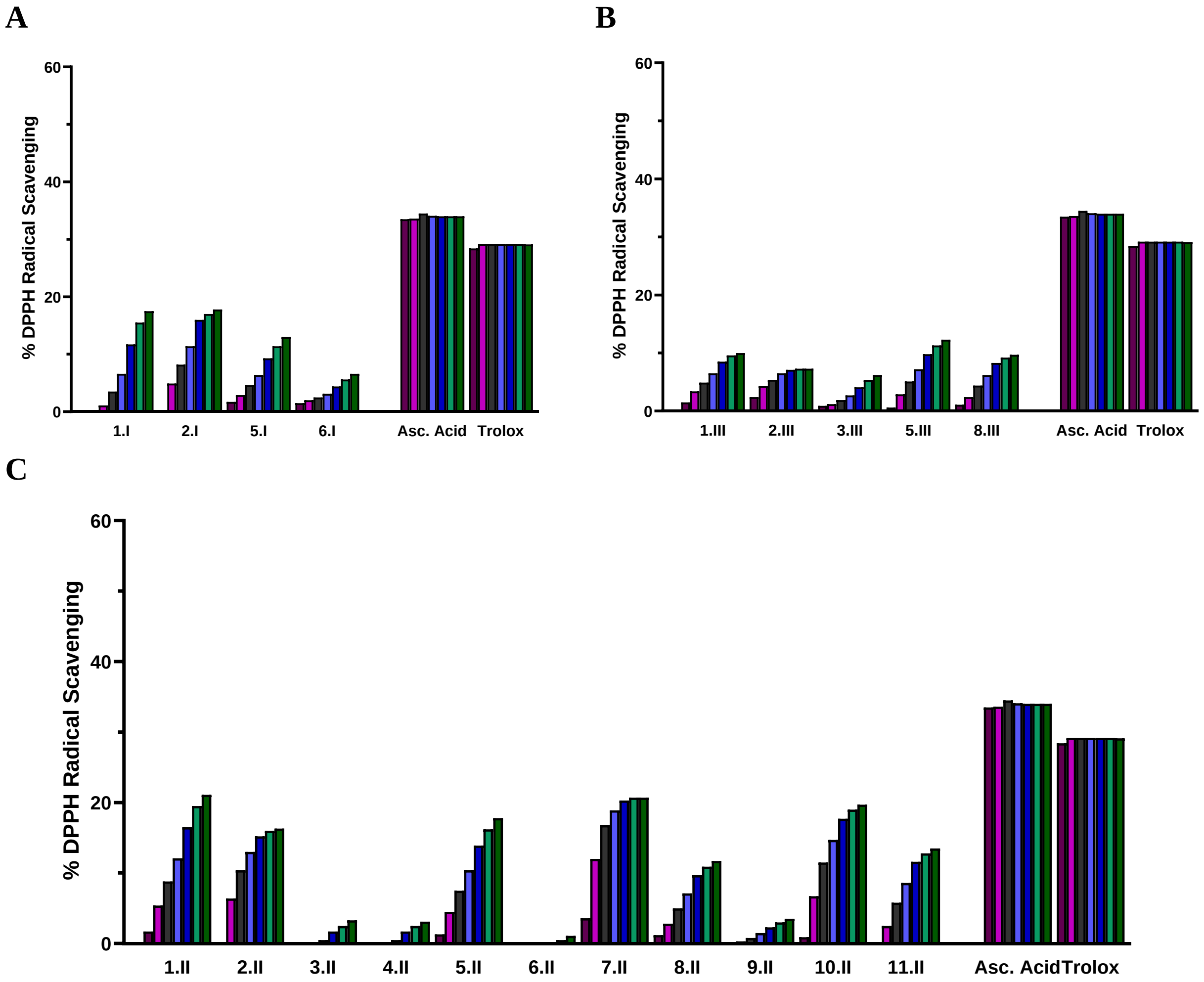

Figure S100. 2,2-diphenyl-1-picrylhydrazyl (DPPH) radical scavenging activity of the novel compounds at 0.003 mg/mL recorded at different points: 0 min (purple), 5 min (pink), 15 min (grey), 30 min (light blue), 60 min (dark blue), 90 min (light green), and 120 min (dark green). (A) Percentage of DPPH$^{\bullet}$ scavenging of compounds from series **I** (propyl); (B) percentage of DPPH$^{\bullet}$ scavenging of compounds from series **III** (propargyl); (C) percentage of DPPH$^{\bullet}$ scavenging of compounds from series **II** (allyl).
